# Supplementary material for: TMEM251 loss-induced autophagy dysfunction in the anterior cingulate cortex contributes to chronic postoperative pain
Source: EMBO Rep. 2025 Dec 3;27(1):186–207. doi: 10.1038/s44319-025-00646-8 (PMC12796400; doi:10.1038/s44319-025-00646-8)
Supplement: Supplementary file 1 — Appendix [file 44319_2025_646_MOESM1_ESM.pdf]

TMEM251 loss-induced autophagy dysfunction in the anterior cingulate cortex contributes to chronic postoperative pain

Authors

Yaowei Xu[1,3,†], Fei Xing[1,3,†], Xin Wei[1], Xiaoling Wang[3,4], Xiaoshan Shi[1,3], Zhongyu Wang[1], Na Xing[1], Jingjing Yuan[1], Zhisong Li[2,3,*] and Wei Zhang[1,3,*]

Affiliations

1. Department of Anesthesiology, Pain and Perioperative Medicine, The First Affiliated Hospital of Zhengzhou University, Zhengzhou, Henan, China

2. Department of Anesthesiology and Perioperative Medicine, The Second Affiliated Hospital of Zhengzhou University, Zhengzhou, Henan, China

3. Neuroscience Research Institute, Zhengzhou University Academy of Medical Sciences, Zhengzhou, Henan, China

4. Department of Anesthesiology and Perioperative Medicine, People's Hospital of Zhengzhou University, Henan Provincial People's Hospital, Zhengzhou, Henan, China

†These authors contributed equally: Yaowei Xu, Fei Xing

*Corresponding authors: Zhisong Li, lzszd@126.com; Wei Zhang, zhangw571012@126.com.

Table of content

[Appendix Figure S1……………………………………………………………………..3]

[Appendix Figure S2……………………………………………………………………..5]

[Appendix Figure S3……………………………………………………………………..7]

[Appendix Figure S4……………………………………………………………………10]

[Appendix Figure S5……………………………………………………………………12]

[Appendix Figure S6……………………………………………………………………14]

[Appendix Figure S7……………………………………………………………………16]

[Appendix Figure S8……………………………………………………………………19]

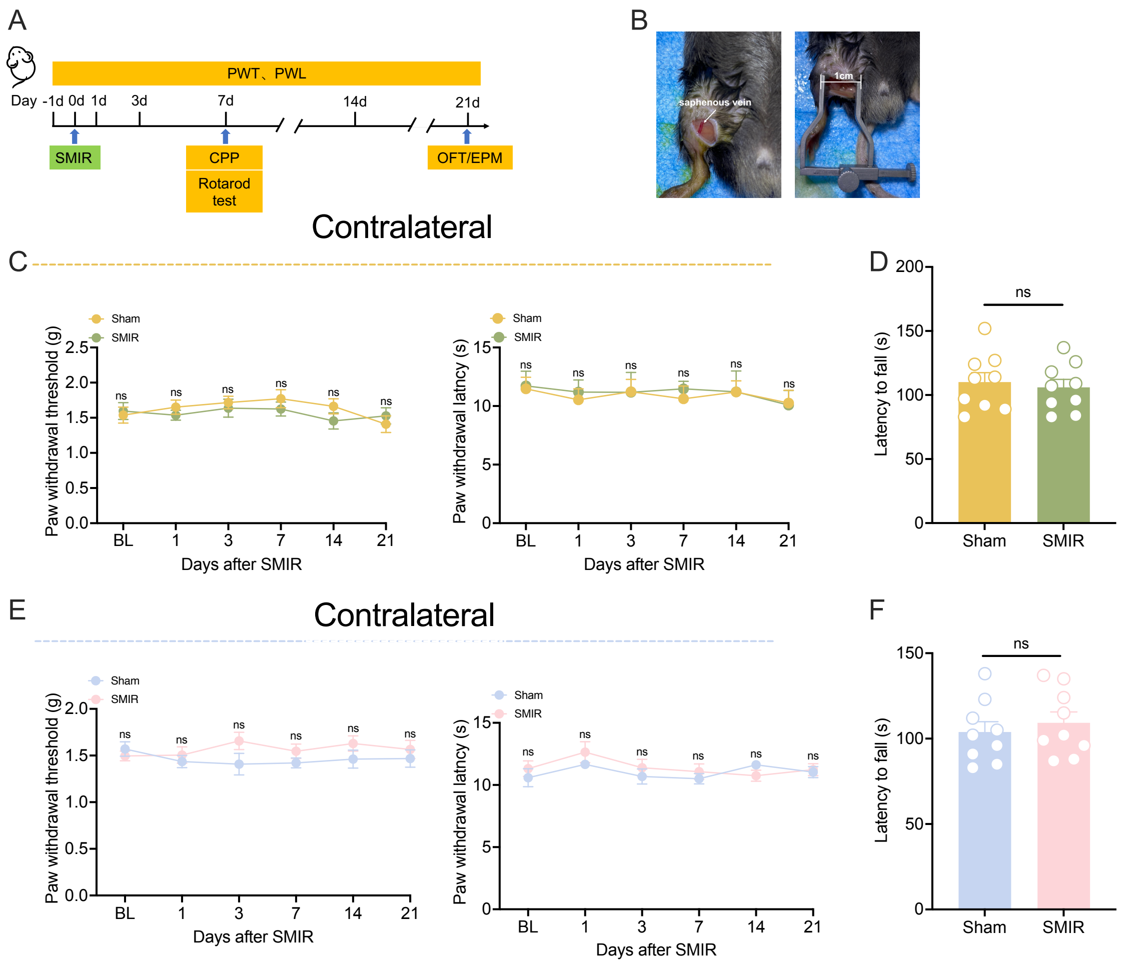

Appendix Figure S1. **Chronic postoperative pain in SMIR group of male and female mice.** **(A, B)** Experimental timeline (A) and schematic of the skin/muscle incision and retraction (SMIR) surgical procedure (B). PWT and PWL were assessed at baseline (day -1) and on POD1, 3, 7, 14, and 21. The CPP and rotarod test were implemented on POD7.The OFT and EPM were conducted on day 21. **(C)** PWT (left) and PWL (right) of the contralateral paws in the sham and SMIR groups of male mice. n = 12 per group; Two-way repeated-measures ANOVA with Bonferroni post hoc correction. **(D)** Latency to fall in the sham and SMIR groups of male mice. n = 9 per group; Two-tailed unpaired Student's t-tests. **(E)** PWT (left) and PWL (right) of the contralateral paws in the sham and SMIR groups of female mice. n = 8 per group; Two-way repeated-measures ANOVA

with Bonferroni post hoc correction. **(F)** Latency to fall in the sham and SMIR groups of female mice. n = 9 per group; Two-tailed unpaired Student's t-tests. All data are represented as mean ± SEM.

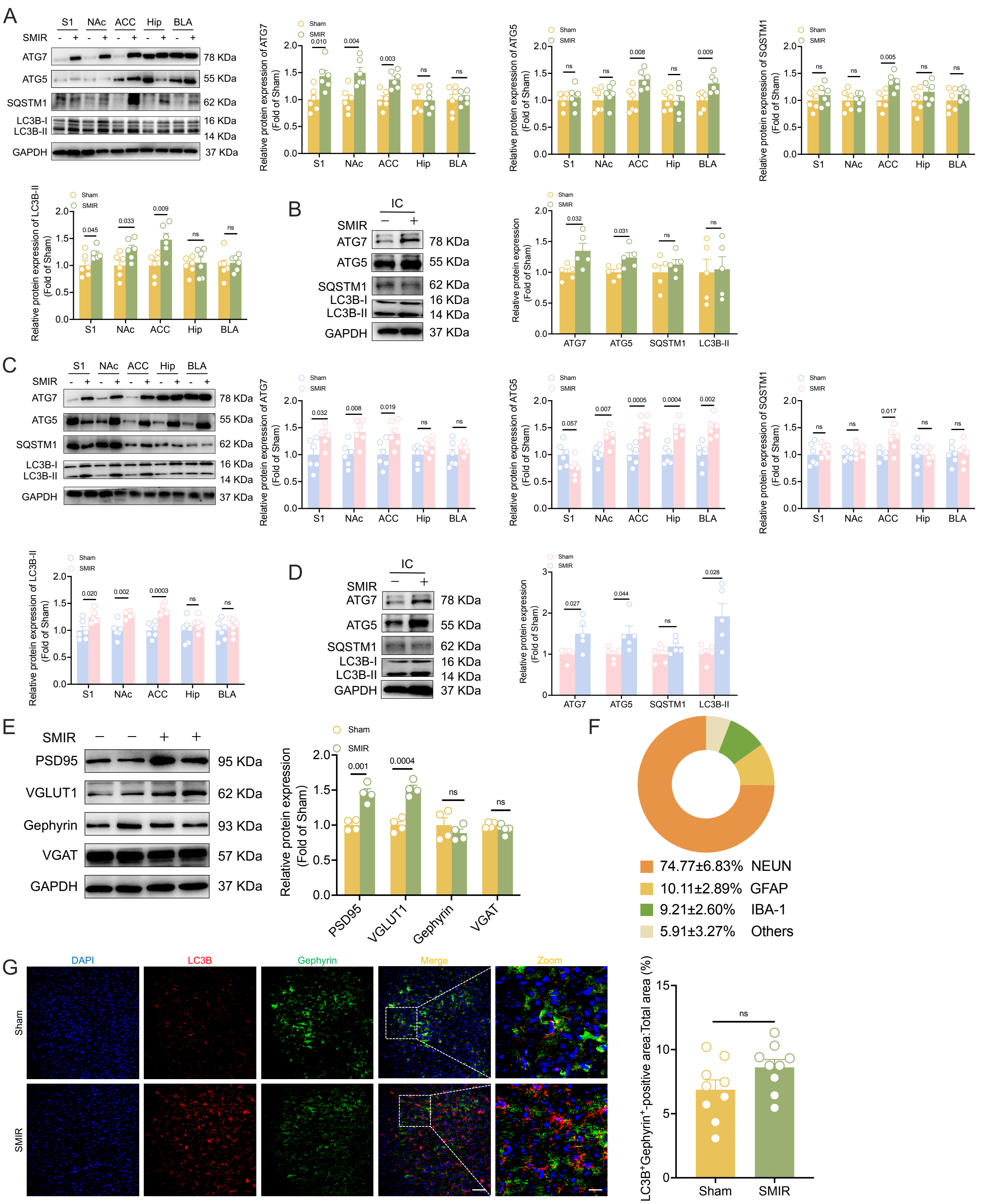

Appendix Figure S2. **The expression of autophagy-related proteins of male and female mice following SMIR surgery.**

(A) Expression levels of autophagy-related proteins (ATG7, ATG5, SQSTM1, and LC3B) in the S1, NAc, ACC, Hip, and BLA of male mice. n = 6 per group; Multiple unpaired t-tests with Bonferroni post hoc correction. **(B)** Expression levels of autophagy-related proteins (ATG7, ATG5, SQSTM1, and LC3B) in the IC of male mice. n = 5 per group; Multiple unpaired t-tests with Bonferroni post hoc correction. **(C)** Expression levels of autophagy-related proteins (ATG7, ATG5, SQSTM1, and LC3B) in the S1, NAc, ACC, Hip, and BLA of female mice. n = 6 per group; Multiple unpaired t-tests with Bonferroni post hoc correction. **(D)** Expression levels of autophagy-related proteins (ATG7, ATG5, SQSTM1, and LC3B) in the IC of female mice. n = 5 per group; Multiple unpaired t-tests with Bonferroni post hoc correction. **(E)** Expression levels of synapse-related proteins (PSD95, VGLUT1, Gephyrin, and VGAT) in the ACC after SMIR surgery. n = 4 per group; Multiple unpaired t-tests with Bonferroni post hoc correction. **(F)** Distribution of SQSTM1 in neurons, astrocytes, and microglia in the ACC of SMIR mice. **(G)** Representative immunofluorescence images (left) and statistical analysis (right) of LC3B colocalization with Gephyrin in the ACC. n = 9 per group; Two-tailed unpaired Student's t-tests. Scale bars: 100 μm and 20 μm. All data are represented as mean ± SEM.

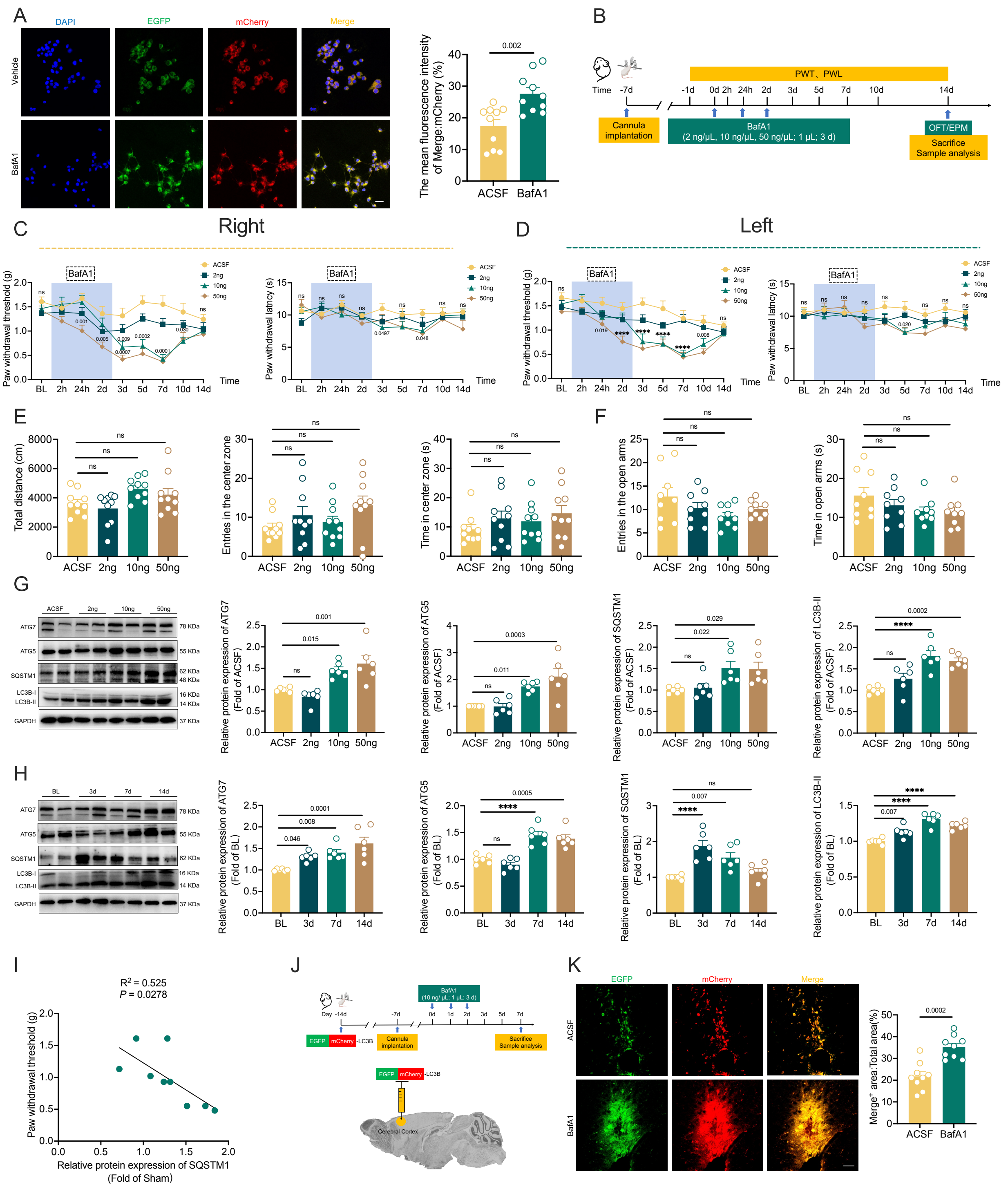

Appendix Figure S3. BafA1 disrupts autophagy in the ACC and induces chronic pain phenotypes in naïve mice.

(**A**) BafA1 elevated the ratio of intracellular merge signal to mCherry signal. Representative images (left) and statistical analysis (right) are shown. n = 10 per group; Two-tailed unpaired Student's t-tests; Scale bar: 20 μm. (**B**) Experimental timeline for ACC infusion of BafA1 (2 ng/μL, 10 ng/μL, and 50 ng/μL) over three consecutive days. PWT and PWL were assessed at baseline (day -1), 2 h and days 1, 3, 5, 7, 10, and 14 post-infusion. OFT and EPM were conducted on day 14. (**C**) Temporal changes in PWT (left) and PWL (right) of the right paws across treatment groups. n = 10 per group; Two-way repeated-measures ANOVA with Bonferroni correction. (**D**) Temporal changes in PWT (left) and PWL (right) of the left paws across treatment groups. n = 10 per group; ****$P < 0.0001$ vs. the ACSF group; Two-way repeated-measures ANOVA with Bonferroni correction. (**E**) OFT behavioral results across treatment groups. n = 10 per group; One-way repeated-measures ANOVA with Bonferroni correction. **(F)** EPM behavioral results across treatment groups. n = 9 per group. One-way repeated measure ANOVA with Bonferroni's post hoc test. (**G**) Different doses (2 ng/μL, 10 ng/μL, and 50 ng/μL) of BafA1 increase the expression of autophagy-related proteins (ATG7, ATG5, SQSTM1 and LC3B) on day 7. Representative western blotting images (left) and data analysis (right). n = 6 per group; ****$P < 0.0001$ vs. the ACSF group; One-way repeated measure ANOVA with Bonferroni's post hoc test. (**H**) Time-dependent expression of autophagy-related proteins (ATG7, ATG5, SQSTM1, and LC3B) in the 10ng group. Representative western blot images (left) and quantification (right). n = 6 per group; ****$P < 0.0001$ vs. the ACSF group; One-way repeated-measures ANOVA with Bonferroni correction. (**I**) Correlation between SQSTM1 expression levels and left PWT in the 10ng group. n = 9 per group; $R^2 = 0.525$; $P = 0.0278$; Simple linear regression analysis. (**J**) Experimental design for pLenti-EGFP-mCherry-LC3B injection (top) and schematic of microinjection procedure (bottom). (**K**) BafA1-induced changes in pLenti-EGFP-mCherry-LC3B. Representative images

(left) and quantification (right) are shown. n = 9 per group; Two-tailed unpaired Student's t-tests.
Scale bar: 50 µm. All data are presented as mean ± SEM.

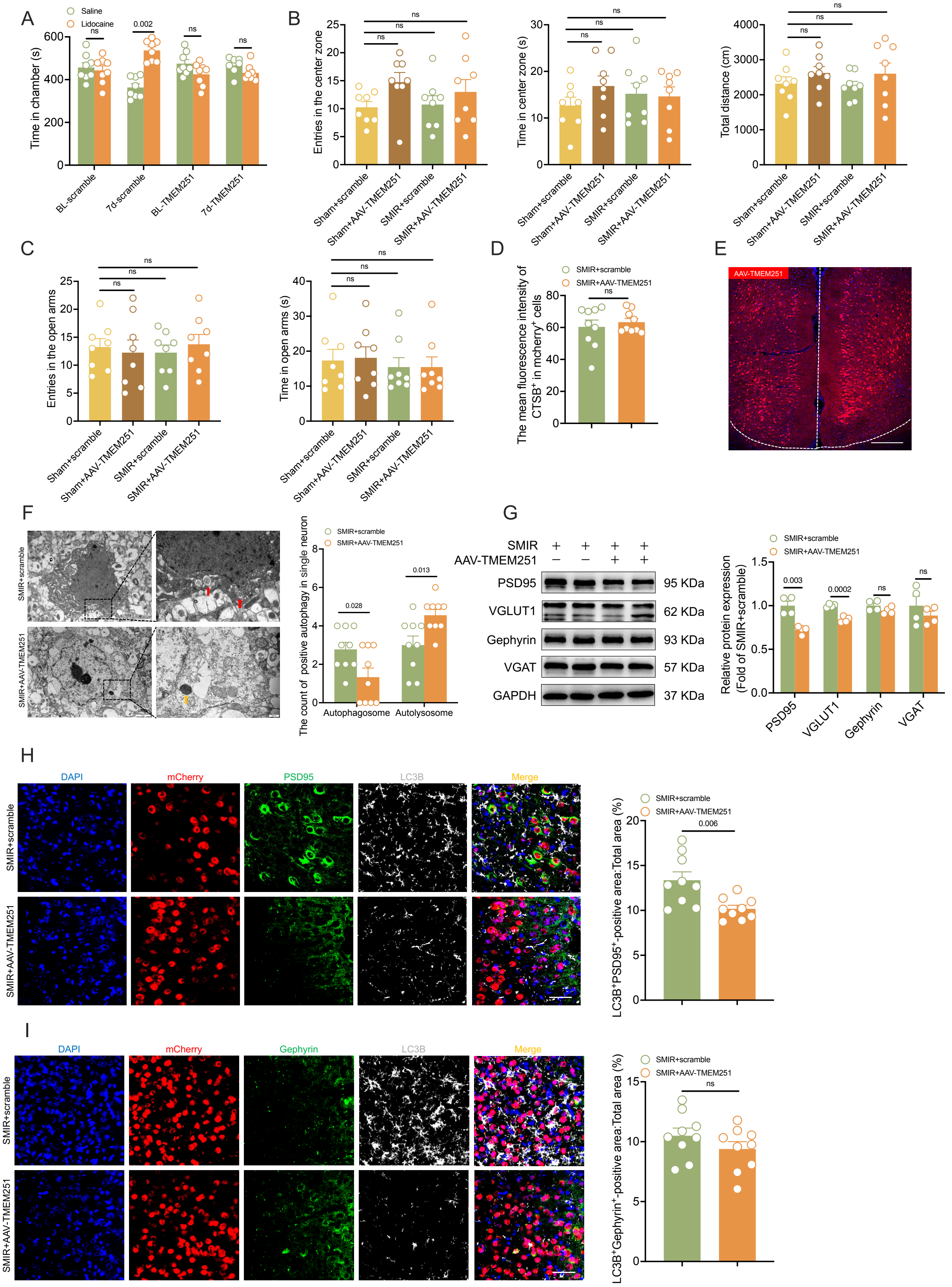

A
Saline
Lidocaine
Time in chamber (s)
BL-scramble
7d-scramble
BL-TMEM251
7d-TMEM251
0.002
ns
B
Entries in the center zone
Time in the center zone (s)
Total distance (cm)
Sham+scramble
Sham+AAV-TMEM251
SMIR+scramble
SMIR+AAV-TMEM251
C
Entries in the open arms
Time in open arms (s)
D
The mean fluorescence intensity of CTSB+ in mcherry+ cells
E
AAV-TMEM251
F
The count of positive autophagy in single neuron
Autophagosome
Autolysosome
0.028
0.013
G
SMIR
AAV-TMEM251
PSD95
95 KDa
VGLUT1
62 KDa
Gephyrin
93 KDa
VGAT
57 KDa
GAPDH
37 KDa
Relative protein expression (Fold of SMIR+scramble)
0.003
0.0002
H
DAPI
mCherry
PSD95
LC3B
Merge
LC3B+PSD95+-positive area:Total area (%)
0.006
I
Gephyrin
LC3B+Gephyrin+-positive area:Total area (%)

Appendix Figure S4. Effect of TMEM251 overexpression on behavioral data and autophagy in mice.

(**A**) The CPP analysis showing increased lidocaine-paired chamber residence time in SMIR+scramble group, not the SMIR+AAV-TMEM251 group at POD7. n = 8 per group; Multiple paired t-tests with Bonferroni post hoc correction. (**B**) Behavioral results of the mice in the OFT. n = 8 per group; One-way repeated measure ANOVA with Bonferroni's post hoc test. (**C**) Behavioral results of the mice in the EPM. n = 8 per group; One-way repeated measure ANOVA with Bonferroni's post hoc test. (**D**) Overexpression of TMEM251 did not affect the expression of CTSB. n = 9 per group; Two-tailed unpaired Student's t-tests. (**E**) Representative images of ACC transfected AAV-TMEM251. The white dashed lines are the boundary of the ACC brain region. Scale bar: 200 μm. (**F**) TEM analysis of ACC neurons. Representative images (left) and quantification (right) show reduced numbers of autophagosomes (red arrows) and increased numbers of autolysosomes (yellow arrows) in SMIR+AAV-TMEM251 mice. n = 9 per group; Multiple unpaired t-tests with Bonferroni post hoc correction; Scale bar: 0.5 μm. (**G**) Expression levels of synapse-related proteins (PSD95, VGLUT1, Gephyrin, and VGAT) in the ACC after TMEM251 overexpression. n = 4 per group; Multiple unpaired t-tests with Bonferroni post hoc correction. (**H**) Representative immunofluorescence images (left) and statistical analysis (right) of LC3B colocalization with PSD95 in the ACC with TMEM251 overexpression. n = 9 per group; Two-tailed unpaired Student's t-tests. Scale bars: 50 μm. **(I)** Representative immunofluorescence images (left) and statistical analysis (right) of LC3B colocalization with Gephyrin in the ACC with TMEM251 overexpression. n = 9 per group; Two-tailed unpaired Student's t-tests. Scale bars: 50 μm. All data are represented as mean ± SEM.

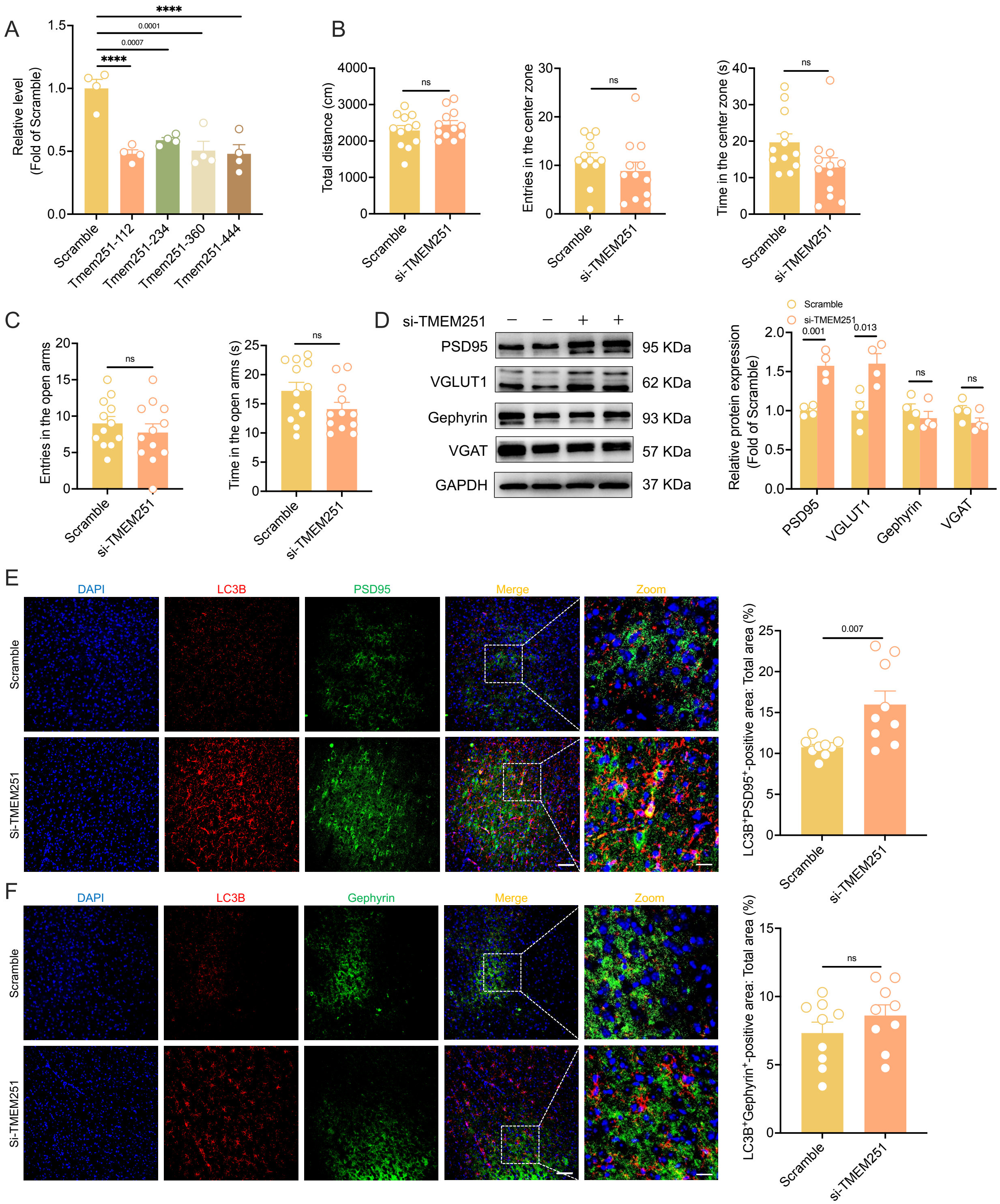

Appendix Figure S5. Effect of TMEM251 knockdown on behavioral data and autophagy in mice.

(**A**) Knockdown levels of four *Tmem251* siRNAs. n = 4 per group; ****P < 0.0001 vs. the scramble group; One-way repeated measure ANOVA with Bonferroni's post hoc test. (**B**) The behavioral OFT results of the mice after knockdown of TMEM251 in the ACC. n = 12 per group; Two-tailed unpaired Student's t-tests. (**C**) The behavioral EPM results of the mice after knockdown of TMEM251 in the ACC. n = 12 per group; Two-tailed unpaired Student's t-tests. (**D**) Expression levels of synapse-related proteins (PSD95, VGLUT1, Gephyrin, and VGAT) in the ACC after TMEM251 knockdown. n = 4 per group; Multiple unpaired t-tests with Bonferroni post hoc correction. (**E**) Representative immunofluorescence images (left) and statistical analysis (right) of LC3B colocalization with PSD95 in the ACC with TMEM251 knockdown. n = 9 per group; Two-tailed unpaired Student's t-tests. Scale bars: 100 μm and 20 μm. **(F)** Representative immunofluorescence images (left) and statistical analysis (right) of LC3B colocalization with Gephyrin in the ACC with TMEM251 knockdown. n = 9 per group; Two-tailed unpaired Student's t-tests. Scale bars: 100 μm and 20 μm. All data are represented as mean ± SEM.

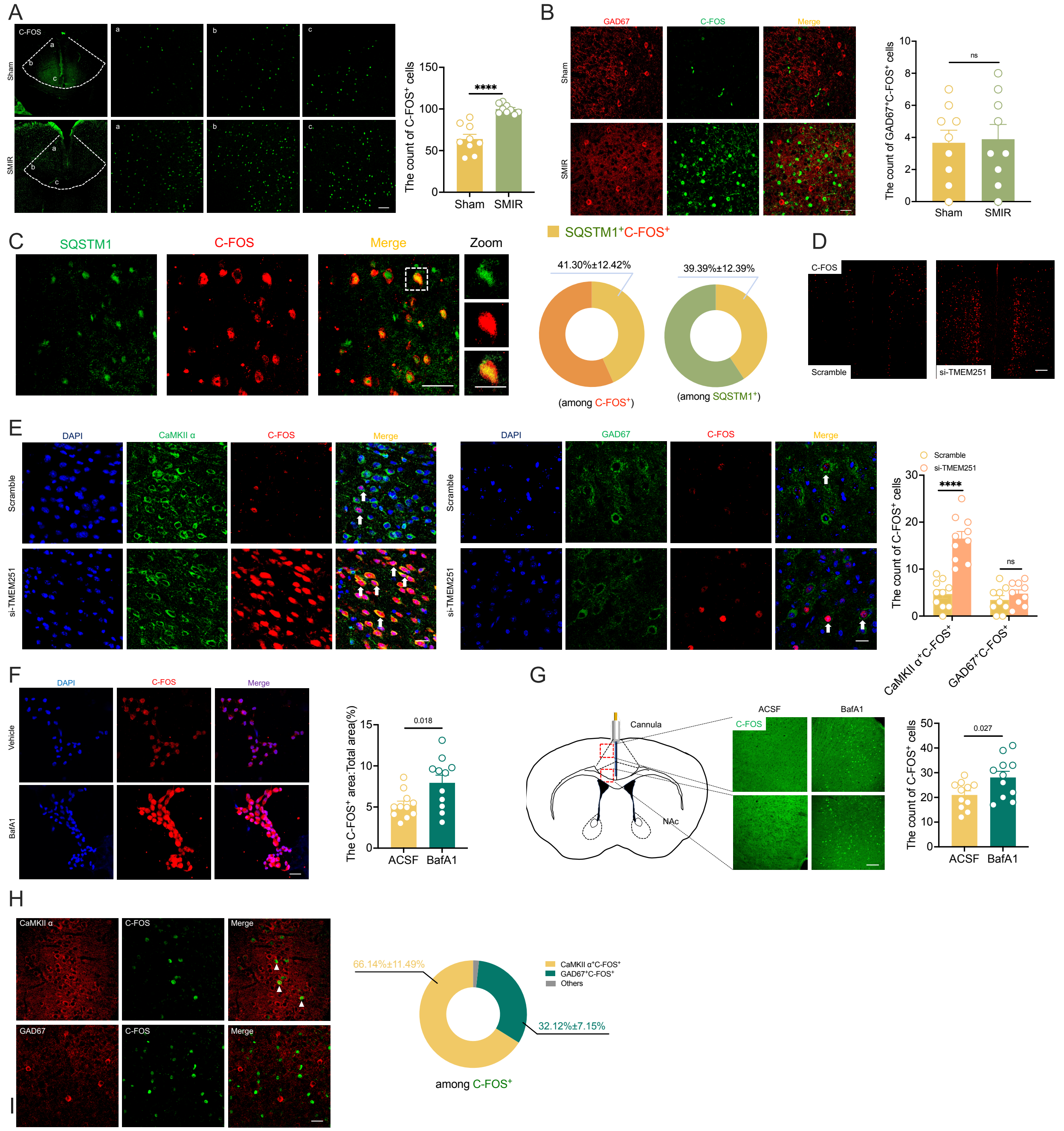

Appendix Figure S6. Impaired autophagy induces significant activation of CaMKIIα-positive neurons in the ACC.

(**A**) Increased number of C-FOS-positive cells in the ACC of SMIR mice. Representative fluorescence images (left) and statistical analysis (right). The white dashed lines are the boundary of the ACC brain region. n = 9 per group; *****P* < 0.0001 vs. the sham group; Two-tailed unpaired

Student's t-tests. Scale bar: 100 μm. (**B**) Representative images (left) and statistical analysis (right) of immunofluorescence colocalization of GAD67 and C-FOS in the sham and SMIR groups. n = 9 per group; Two-tailed unpaired Student's t-tests. Scale bar: 50 μm. (**C**) Representative images (left) and statistical analysis (right) of immunofluorescence colocalization of SQSTM1 and C-FOS. Scale bar: 20 μm and 10 μm. (**D**) TMEM251 knockdown increased the expression of C-FOS in the ACC. Scale bar: 50 μm. (**E**) Knockdown of TMEM251 predominantly activated CaMKIIα-positive cells (white arrows) but not GAD67-positive cells (white arrows). Representative images (left and middle) and statistical analysis (right) are shown. n = 9-10 per group; $^{****}P < 0.0001$ vs. the scramble group; Multiple unpaired t-tests with Bonferroni post hoc correction. (**F**) BafA1 induced intracellular C-FOS expression. Representative fluorescent images (left) and statistical analysis (right). n = 11 per group; Two-tailed unpaired Student's t-tests. Scale bar: 20 μm. (**G**) BafA1 increased the number of C-FOS-positive cells in the ACC. Representative images (left) and statistical analysis (right) are shown. n = 11 per group; Two-tailed unpaired Student's t-tests. Scale bar: 50 μm. (**H**) The C-FOS in the ACC of BafA1-treated mice was mainly concentrated in CaMKIIα-positive cells (white arrows), rather than GAD67-positive cells. Representative fluorescence images (left) and pie charts (right) are shown. Scale bar: 20 μm. All data are represented as mean ± SEM.

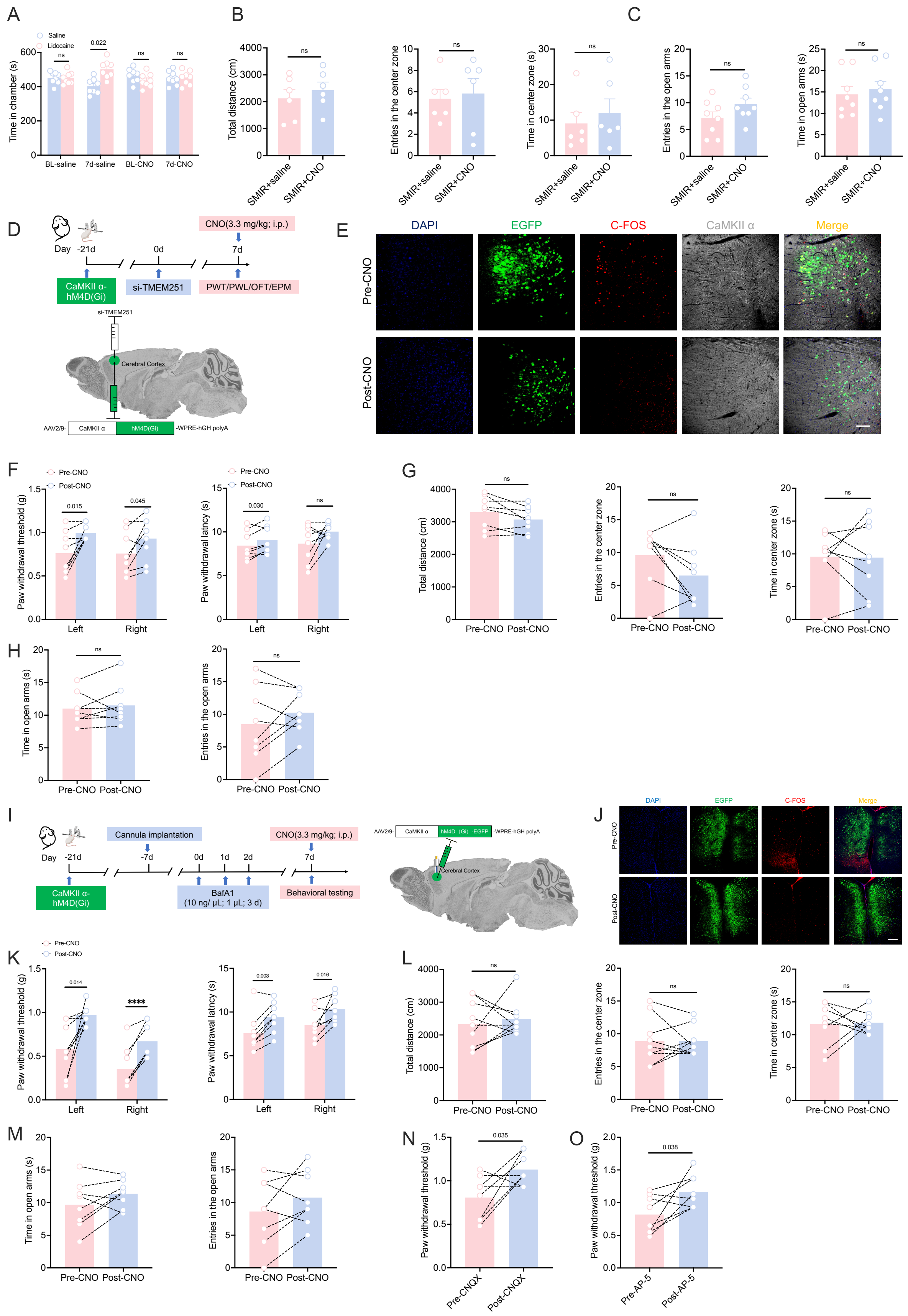
A
B
C
D
E
F
G
H
I
J
K
L
M
N
O
Saline
Lidocaine
Time in chamber (s)
BL-saline
7d-saline
BL-CNO
7d-CNO
Total distance (cm)
Entries in the center zone
Time in center zone (s)
Entries in the open arms
Time in open arms (s)
SMIR+saline
SMIR+CNO
CNO(3.3 mg/kg; i.p.)
Day -21d
0d
7d
CaMKII α-hM4D(Gi)
si-TMEM251
PWT/PWL/OFT/EPM
Cerebral Cortex
AAV2/9-
CaMKII α
hM4D(Gi)
-WPRE-hGH polyA
DAPI
EGFP
C-FOS
CaMKII α
Merge
Pre-CNO
Post-CNO
Paw withdrawal threshold (g)
Paw withdrawal latncy (s)
Left
Right
Cannula implantation
-7d
1d
2d
BafA1
(10 ng/ μL; 1 μL; 3 d)
Behavioral testing
hM4D (Gi) -EGFP
Pre-CNQX
Post-CNQX
Pre-AP-5
Post-AP-5

Appendix Figure S7. Chemogenetic inhibition of CaMKIIα-positive neurons in the ACC attenuates CPOP associated with impaired autophagy in mice.

(**A**) The CPP analysis showing increased lidocaine-paired chamber residence time in male SMIR+saline group, not the SMIR+CNO group at POD7. n = 8 per group; Multiple paired t-tests with Bonferroni post hoc correction. (**B**) Behavioral data of the OFT after inhibition of activated CaMKIIα-positive neurons. n = 6 per group; Two-tailed unpaired Student's t-tests. (**C**) Behavioral data of the EPM after inhibition of activated CaMKIIα-positive neurons in SMIR mice. n = 8 per group; Two-tailed unpaired Student's t-tests. (**D**) Flowchart (top) and schematic diagram (bottom) of the chemogenetic inhibition for CaMKIIα-positive neurons in the ACC. We injected the AAV-CaMKIIα-hM4D(Gi) virus into the bilateral ACC of the siTMEM251 group. CNO (3.3 mg/kg) was injected intraperitoneally, and behavioral tests were performed on day 7 after the microinjection of siTMEM251. (**E**) CNO inhibited the expression of C-FOS in the $EGFP^+$ region. Scale bar: 100 μm. (**F**) Inhibition of activated CaMKIIα-positive neurons in the ACC increased the PWT (left) and PWL (right) in the bilateral paws of siTMEM251-treated mice. n = 8 per group; Multiple paired t-tests with Bonferroni post hoc correction. (**G**) Behavioral data of the OFT after the inhibition of activated CaMKIIα-positive neurons in siTMEM251-treated mice. n = 8 per group; Two-tailed paired Student's t-tests. (**H**) Behavioral results of the EPM after the inhibition of activated CaMKIIα-positive neurons in siTMEM251-treated mice. n = 8 per group; Multiple paired t-tests with Bonferroni post hoc correction. (**I**) Flowchart (left) and schematic diagram (right) of the chemogenetic experiment for CaMKIIα-positive neurons in the ACC. We injected the AAV-CaMKIIα-hM4D (Gi) virus into the ACC and implanted cannulas into the viral transfection site two weeks later. CNO (3.3 mg/kg) was injected intraperitoneally, and behavioral tests were performed on day 7 after BafA1 infusion. (**J**) CNO inhibited the expression of C-FOS in the $EGFP^+$

region. Scale bar: 200 μm. (**K**) Inhibition of activated CaMKIIα-positive neurons in the ACC increased the PWT (left) and PWL (right) in the bilateral paws of BafA1-treated mice. n = 8 per group; ****P < 0.0001 vs. the pre-CNO group; Multiple paired t-tests with Bonferroni post hoc correction. (**L**) Behavioral data of the OFT after the inhibition of CaMKIIα-positive neurons in BafA1-treated mice. n = 8 per group; Two-tailed paired Student's t-tests. (**M**) Behavioral data of the EPM after the inhibition of CaMKIIα-positive neurons in BafA1-treated mice. n = 8 per group; Two-tailed paired Student's t-tests. (**N**) Administration of CNQX in the ACC significantly increased ipsilateral PWT in SMIR mice on POD7. n = 8 per group; Two-tailed paired Student's t-tests. (**O**) Administration of AP-5 in the ACC significantly increased ipsilateral PWT in SMIR mice on POD7. n = 8 per group; Two-tailed paired Student's t-tests. All data are represented as mean ± SEM.

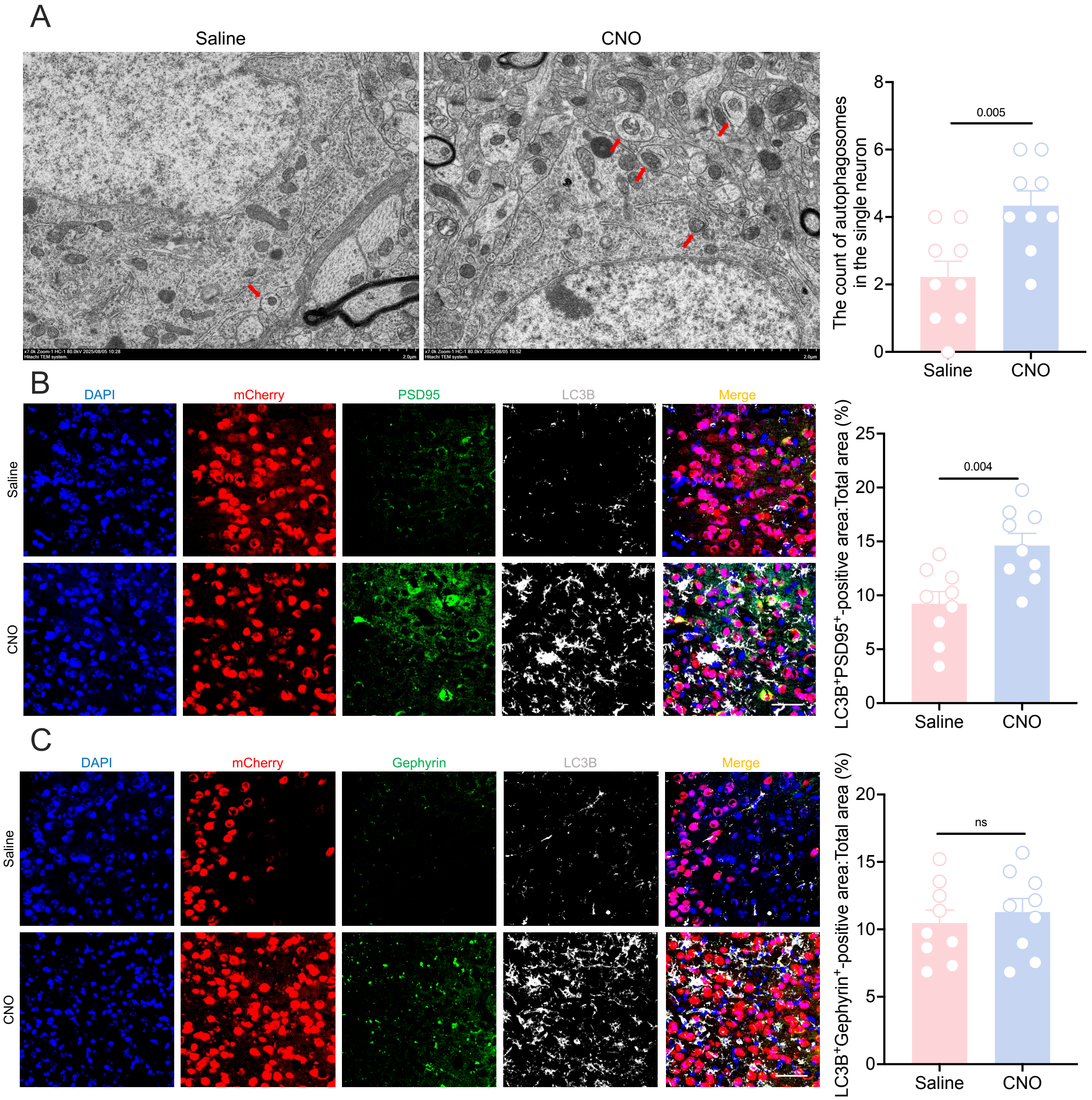

Appendix Figure S8. Chemogenetic activation of CaMKIIα-positive neurons in the ACC induces the accumulation of autophagy substrates.

(**A**) TEM analysis showed a significant increase in the number of autophagosomes (red arrows) in the ACC neurons of CNO group. n = 9 per group; Two-tailed unpaired Student's t-tests. Scale bar: 2 μm. (**B**) The application of CNO significantly increased the colocalization signal of LC3B and PSD95 in the ACC. n = 9 per group; Two-tailed unpaired Student's t-tests. Scale bar: 50 μm. (**C**)

The application of CNO has no effect on the colocalization signal of LC3B and Gephyrin in the ACC. n = 9 per group; Two-tailed unpaired Student's t-tests. Scale bar: 50 μm. All data are represented as mean ± SEM.
