# Supplementary figures and images for: TMEM251 loss-induced autophagy dysfunction in the anterior cingulate cortex contributes to chronic postoperative pain (part 1 of 2)
Source: EMBO Rep. 2025 Dec 3;27(1):186–207. doi: 10.1038/s44319-025-00646-8 (PMC12796400; doi:10.1038/s44319-025-00646-8)

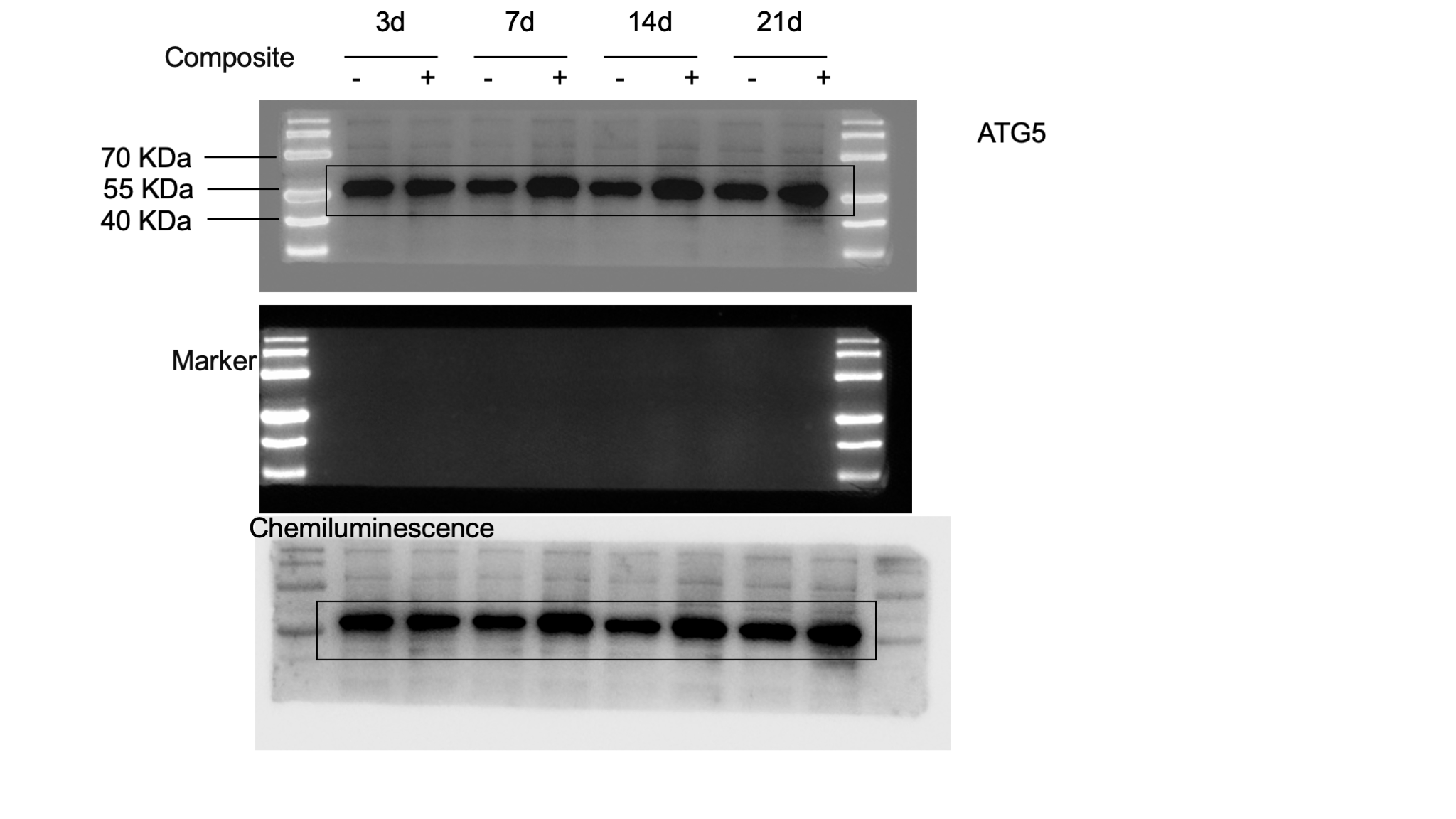

Supplement: Supplementary file 4 — Source data Fig. 2 [file 44319_2025_646_MOESM4_ESM.zip › Figure 2/2A/2A-ATG5.tiff]

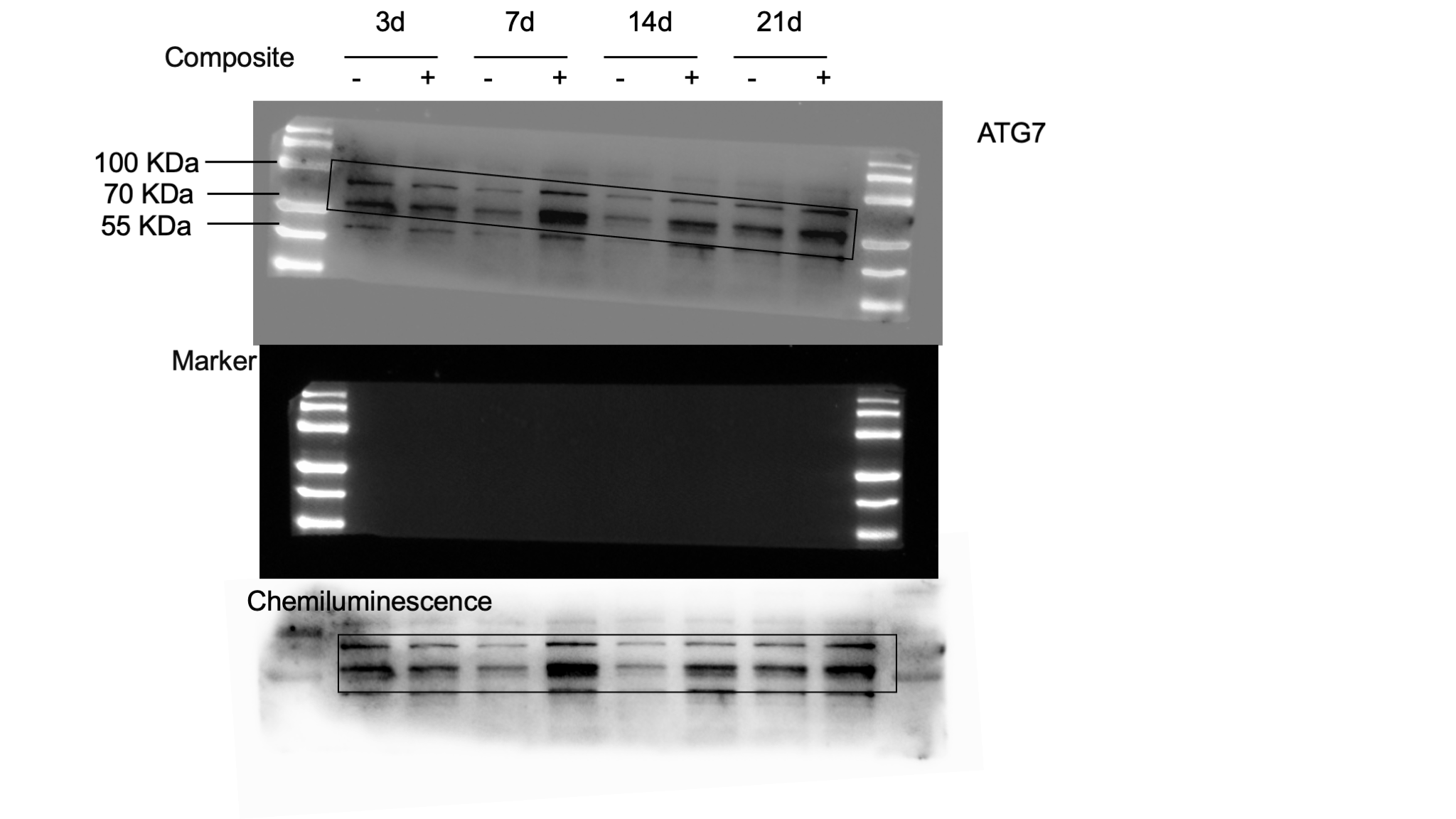

Supplement: Supplementary file 4 — Source data Fig. 2 [file 44319_2025_646_MOESM4_ESM.zip › Figure 2/2A/2A-ATG7.tiff]

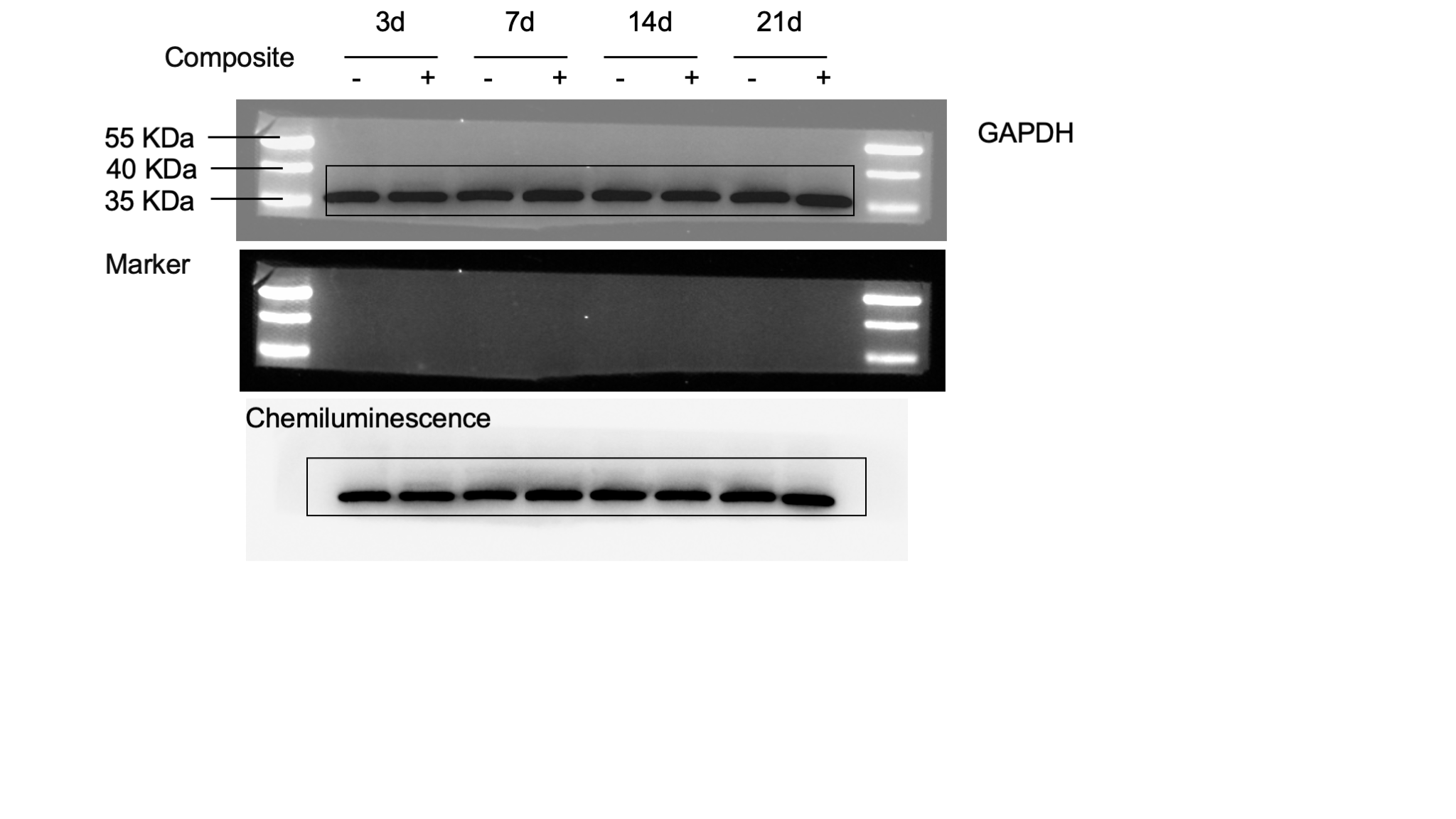

Supplement: Supplementary file 4 — Source data Fig. 2 [file 44319_2025_646_MOESM4_ESM.zip › Figure 2/2A/2A-GAPDH.tiff]

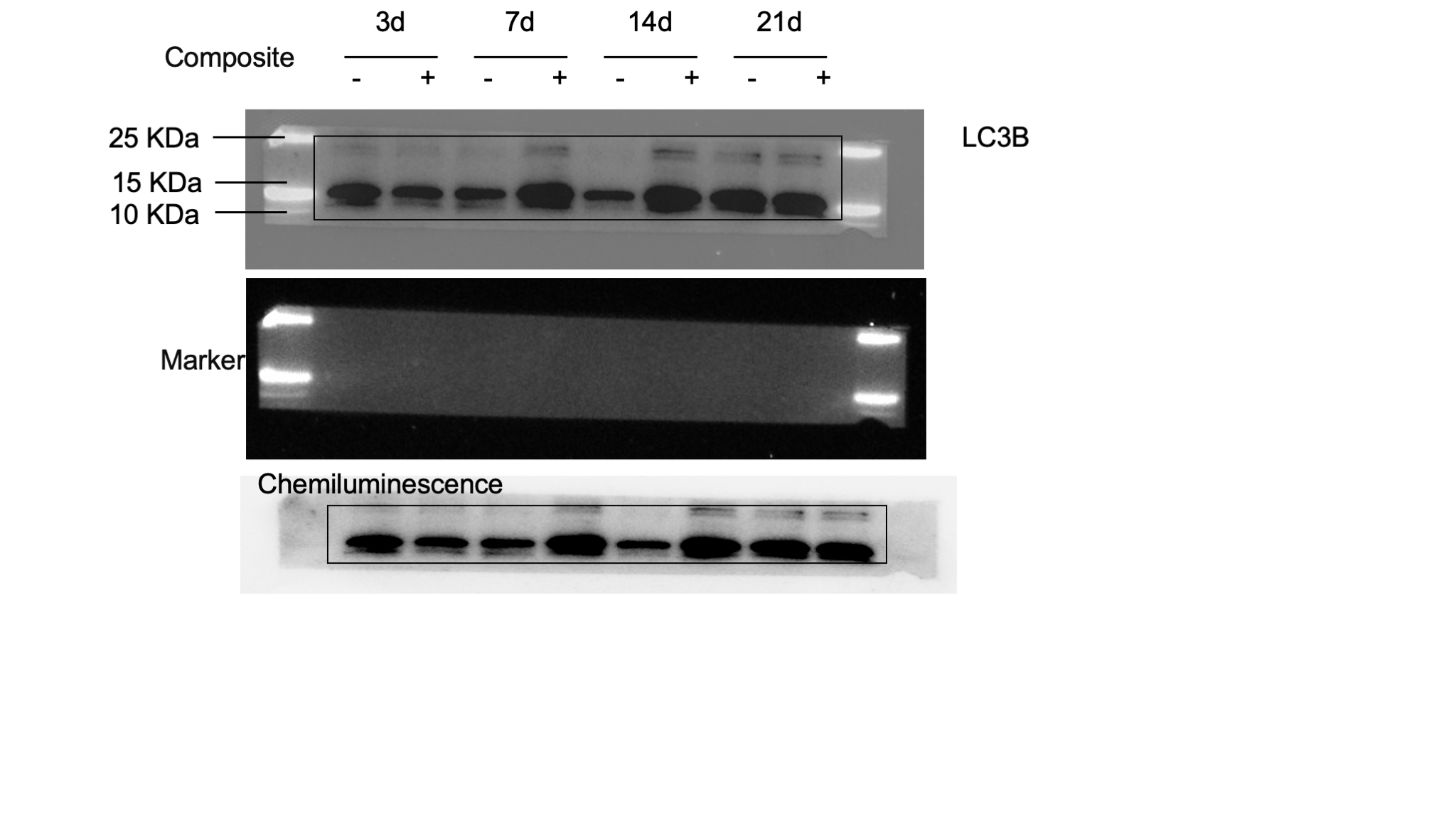

Supplement: Supplementary file 4 — Source data Fig. 2 [file 44319_2025_646_MOESM4_ESM.zip › Figure 2/2A/2A-LC3B.tiff]

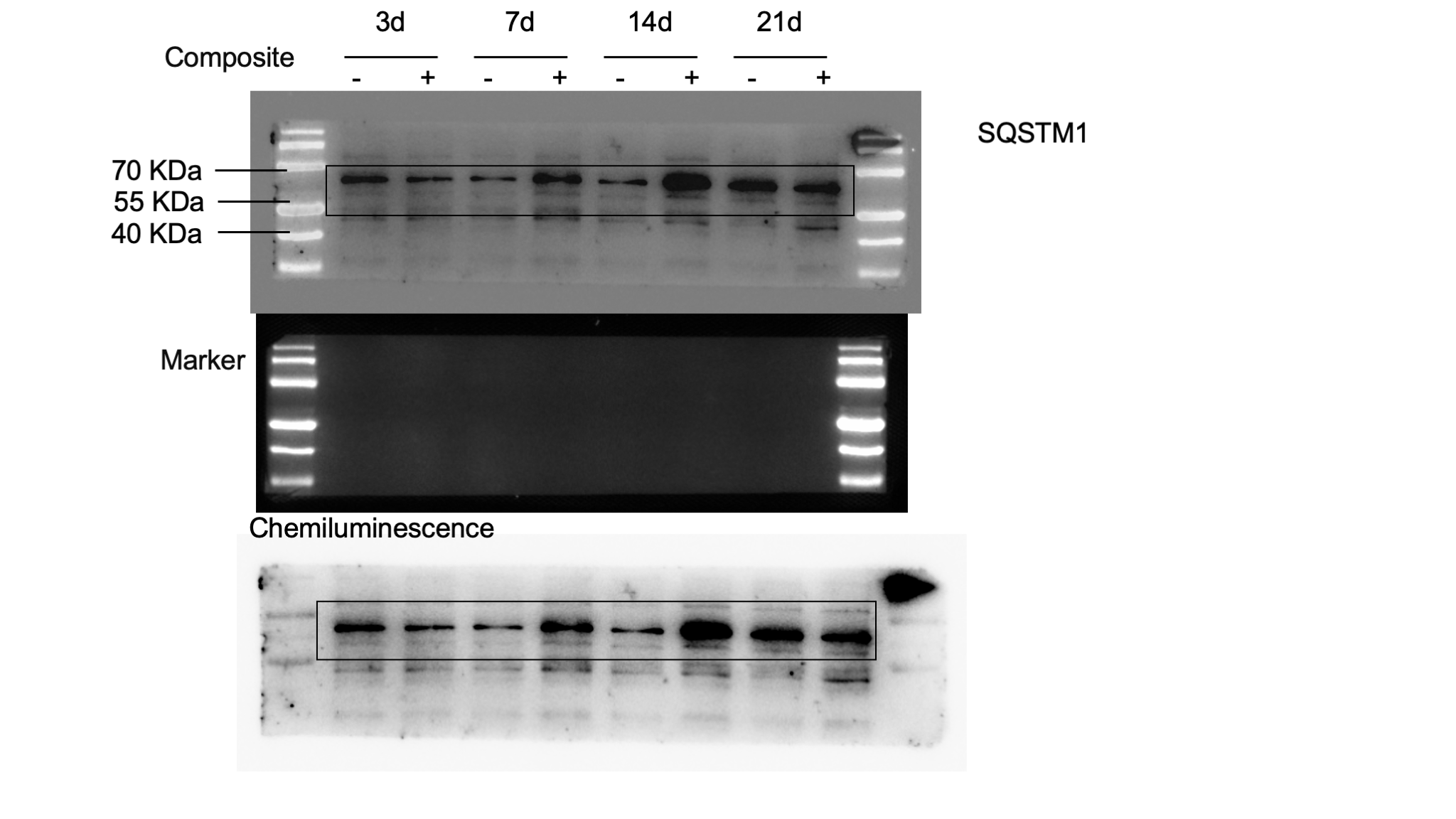

Supplement: Supplementary file 4 — Source data Fig. 2 [file 44319_2025_646_MOESM4_ESM.zip › Figure 2/2A/2A-SQSTM1.tiff]

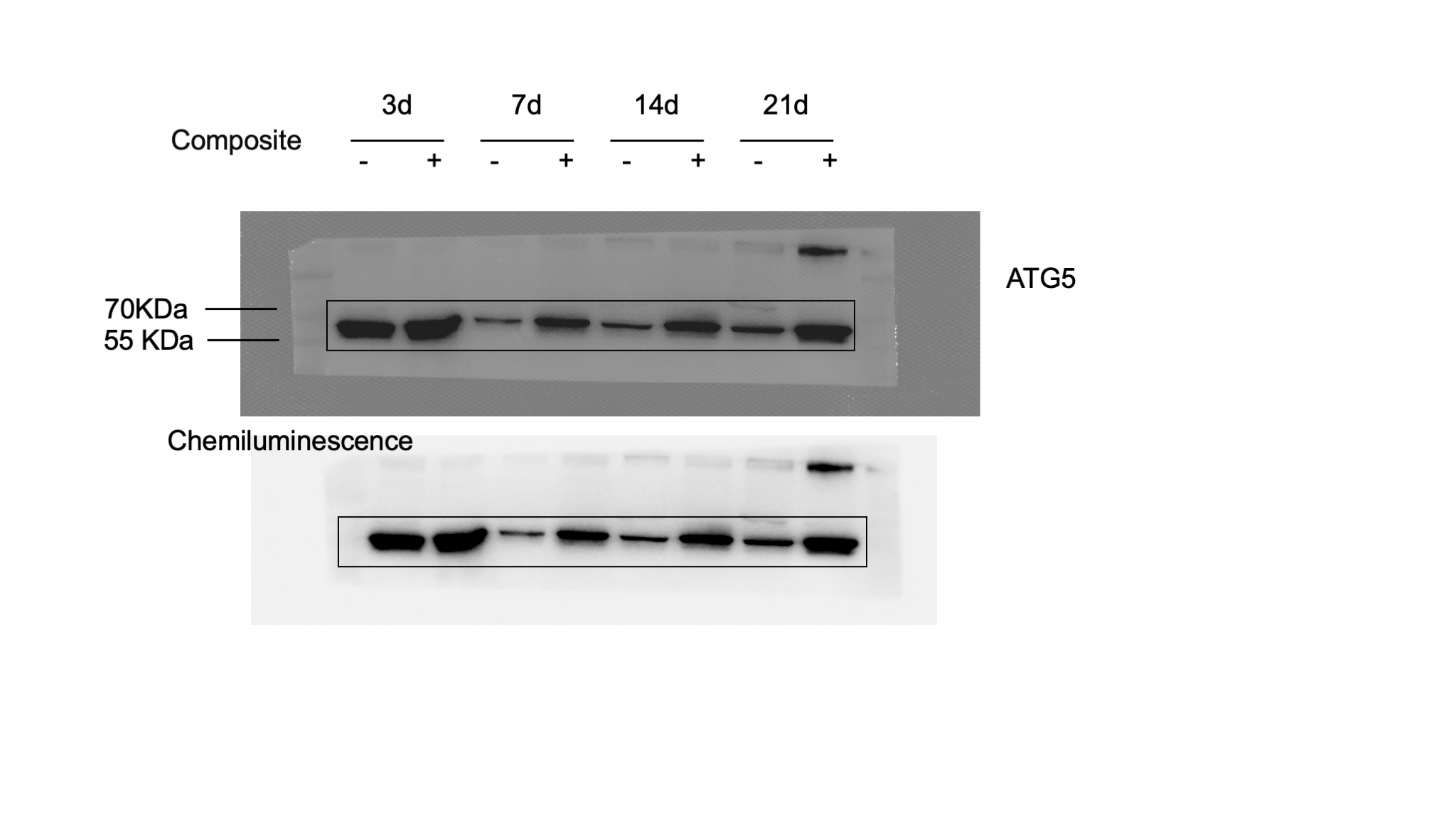

Supplement: Supplementary file 4 — Source data Fig. 2 [file 44319_2025_646_MOESM4_ESM.zip › Figure 2/2B/2B-ATG5.tiff]

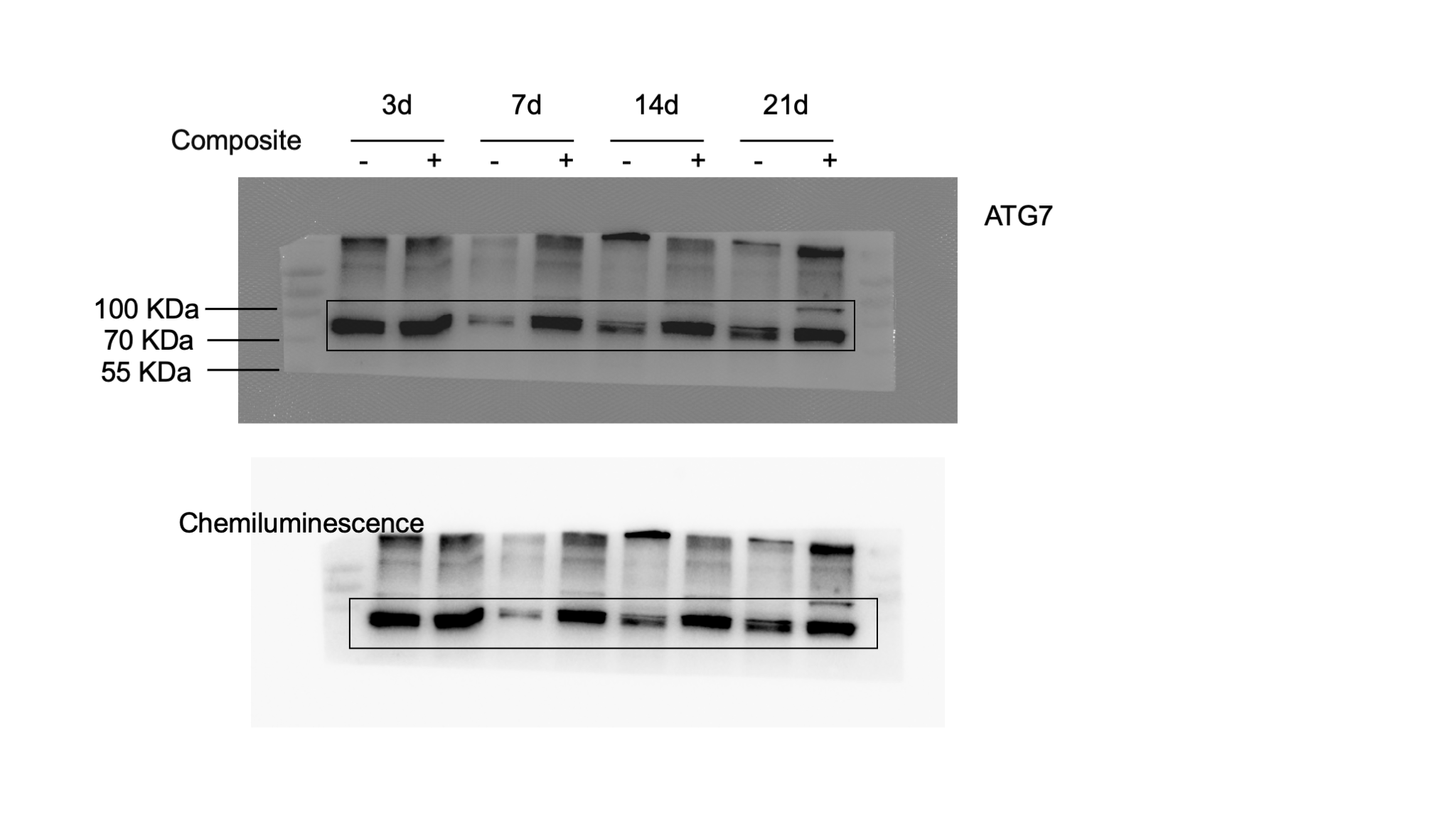

Supplement: Supplementary file 4 — Source data Fig. 2 [file 44319_2025_646_MOESM4_ESM.zip › Figure 2/2B/2B-ATG7.tiff]

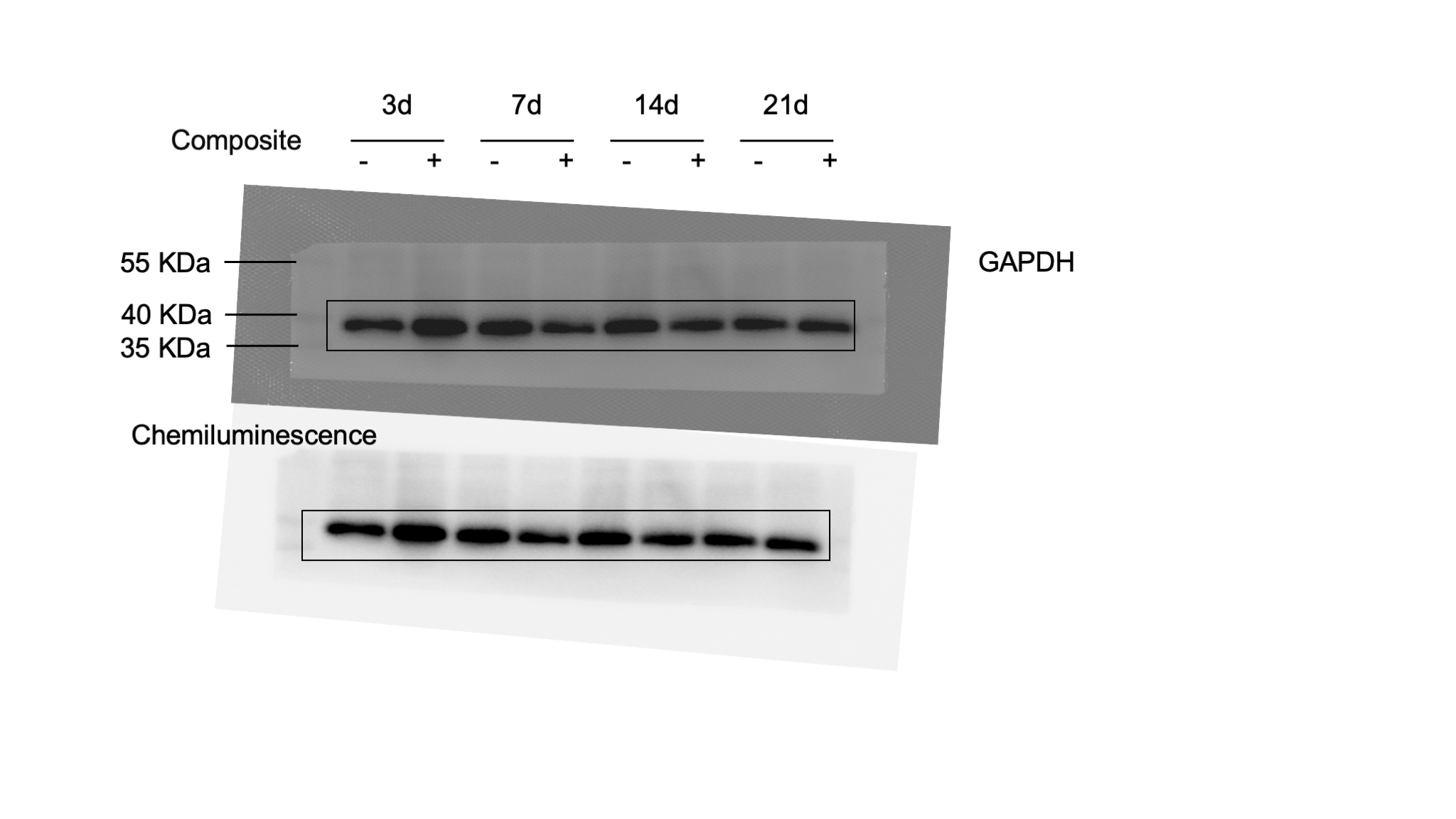

Supplement: Supplementary file 4 — Source data Fig. 2 [file 44319_2025_646_MOESM4_ESM.zip › Figure 2/2B/2B-GAPDH.tiff]

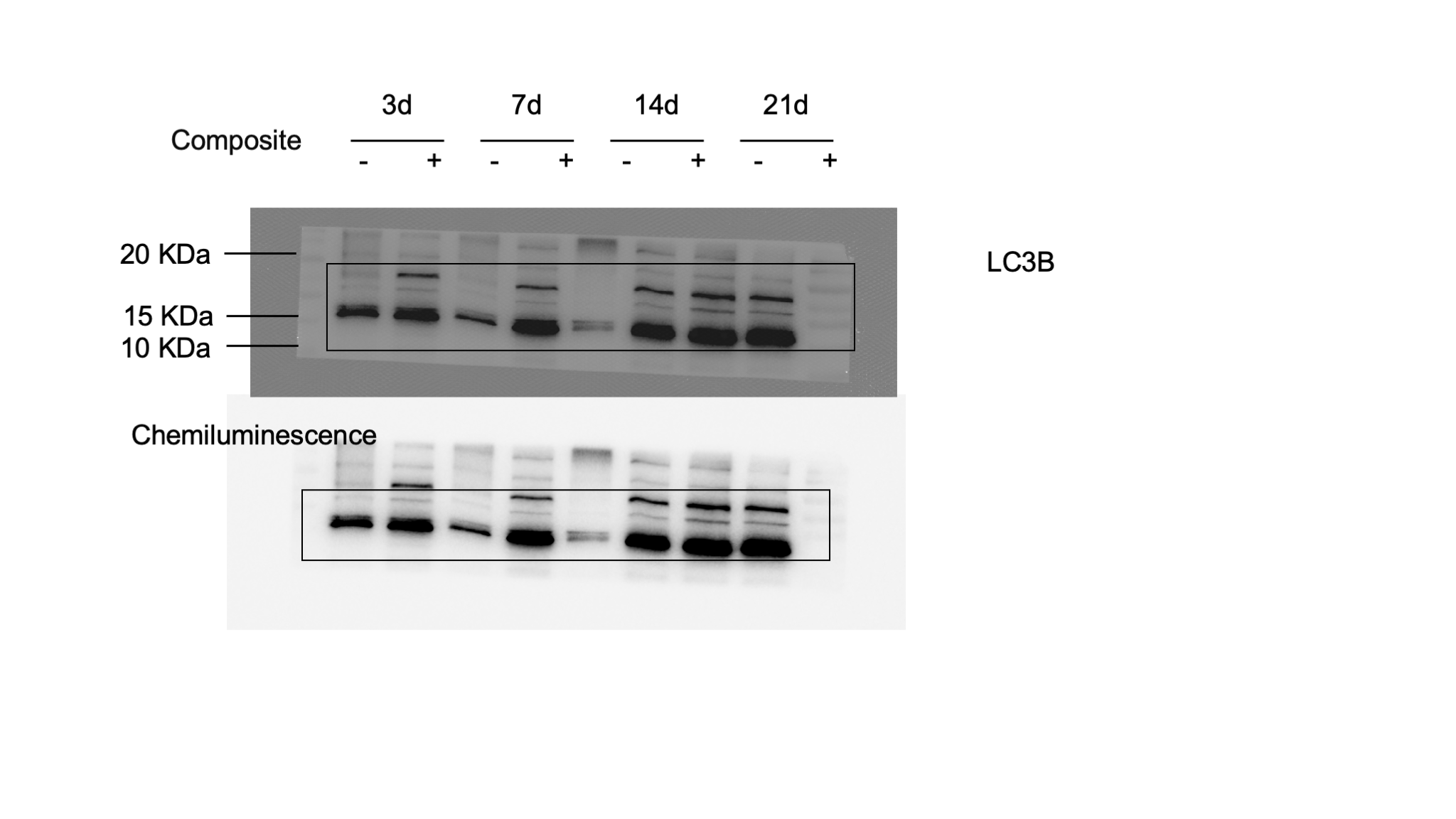

Supplement: Supplementary file 4 — Source data Fig. 2 [file 44319_2025_646_MOESM4_ESM.zip › Figure 2/2B/2B-LC3B.tiff]

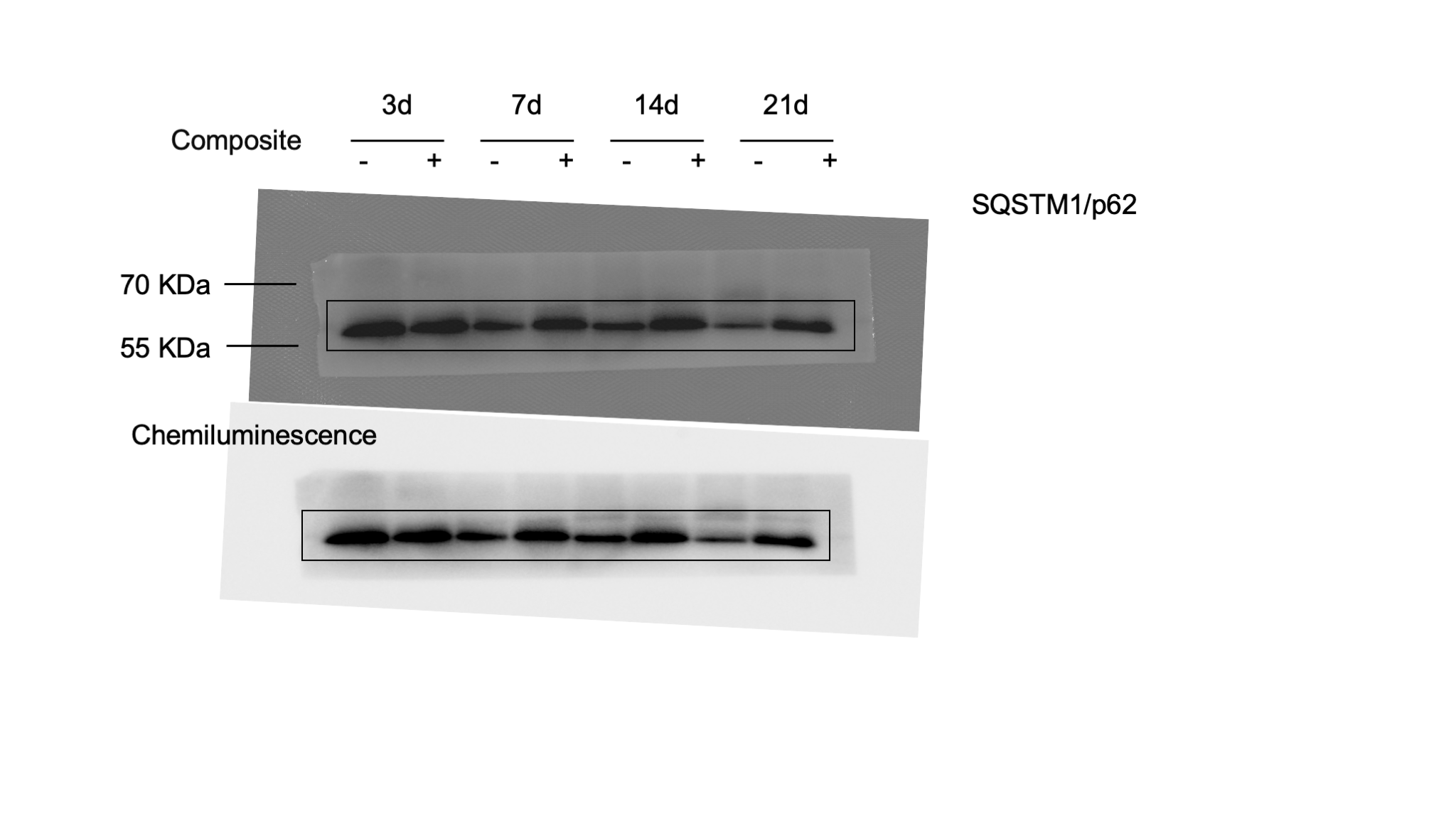

Supplement: Supplementary file 4 — Source data Fig. 2 [file 44319_2025_646_MOESM4_ESM.zip › Figure 2/2B/2B-SQSTM1.tiff]

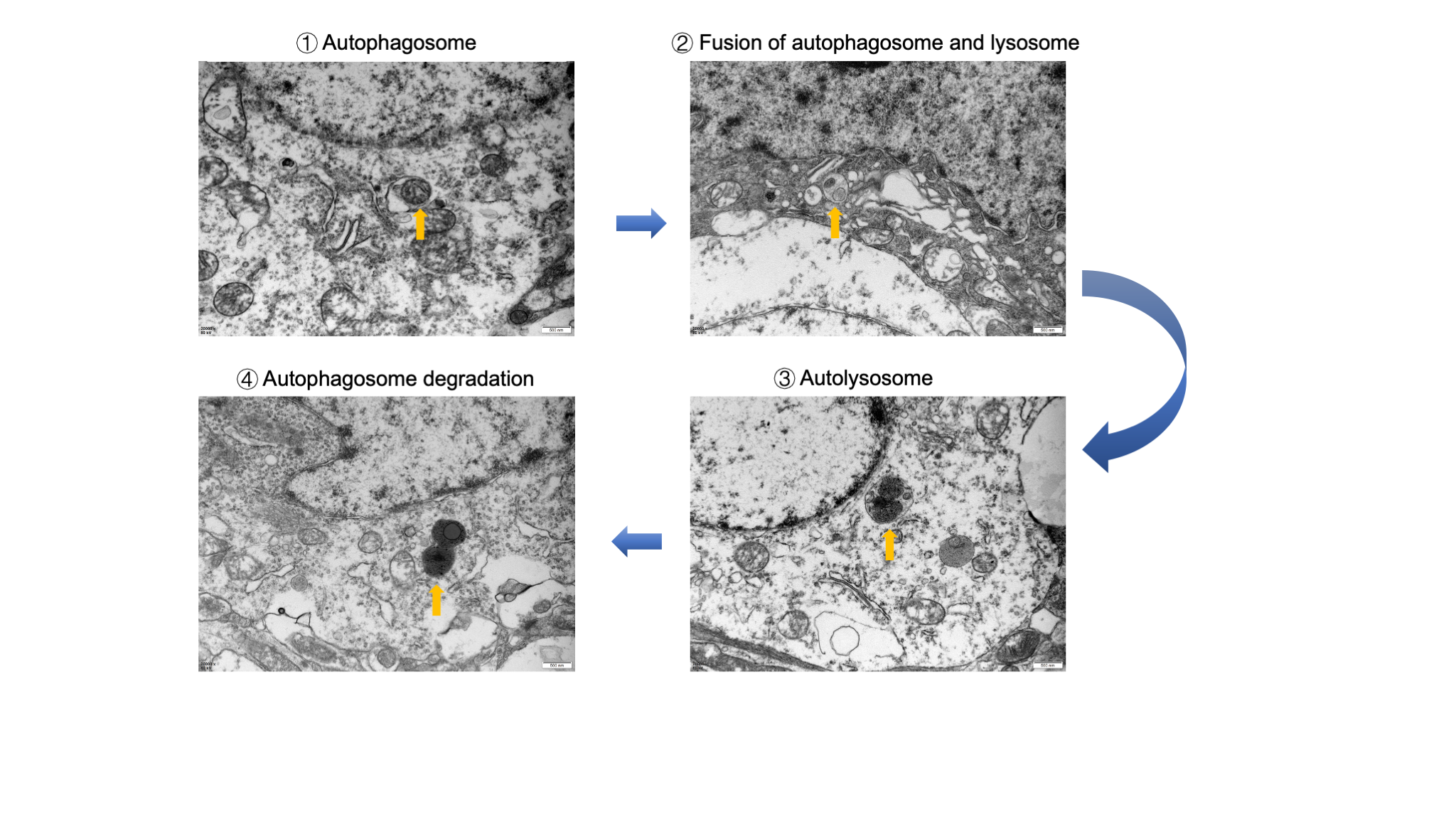

Supplement: Supplementary file 4 — Source data Fig. 2 [file 44319_2025_646_MOESM4_ESM.zip › Figure 2/2C/2C.tiff]

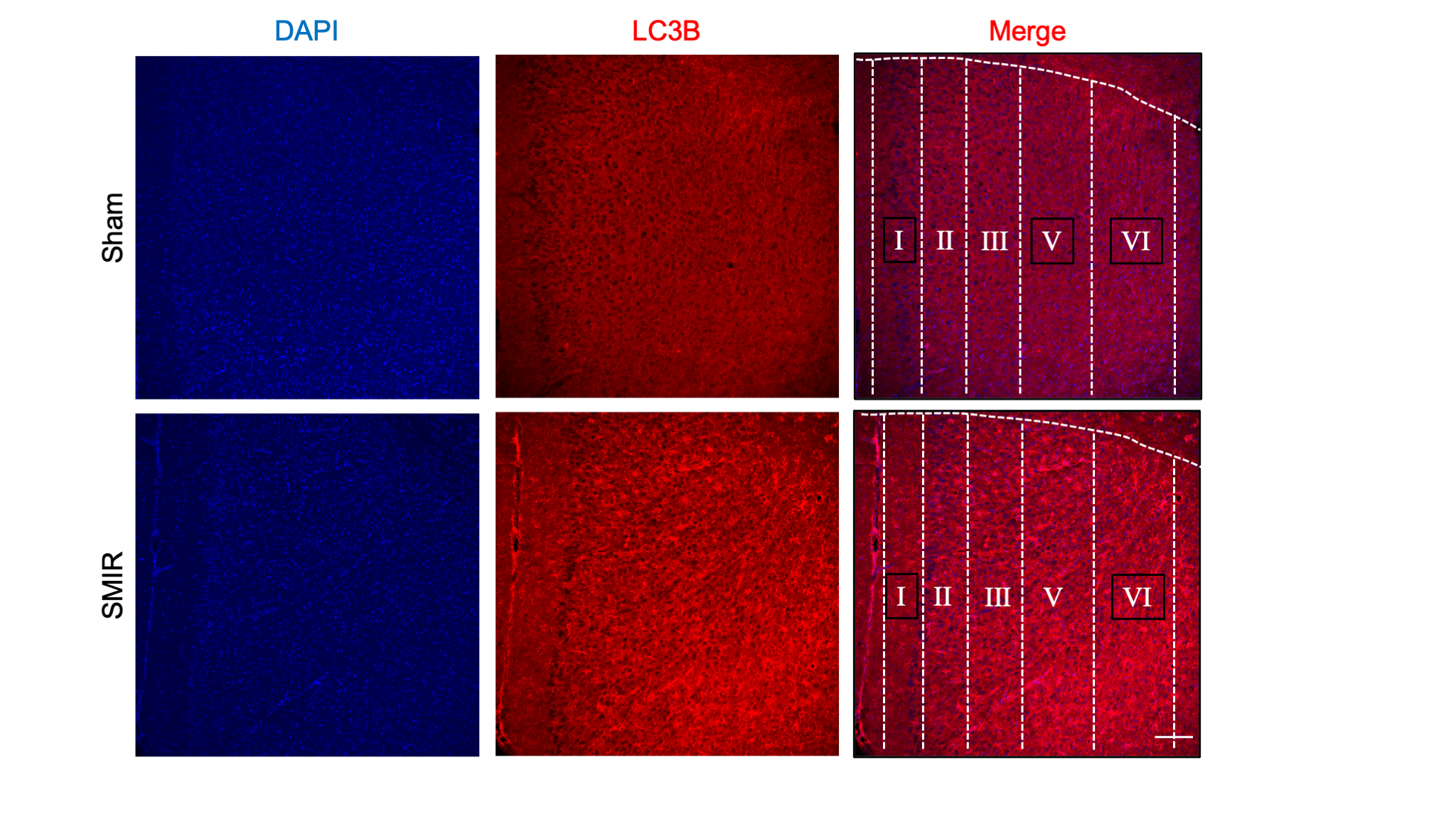

Supplement: Supplementary file 4 — Source data Fig. 2 [file 44319_2025_646_MOESM4_ESM.zip › Figure 2/2D/2D.tiff]

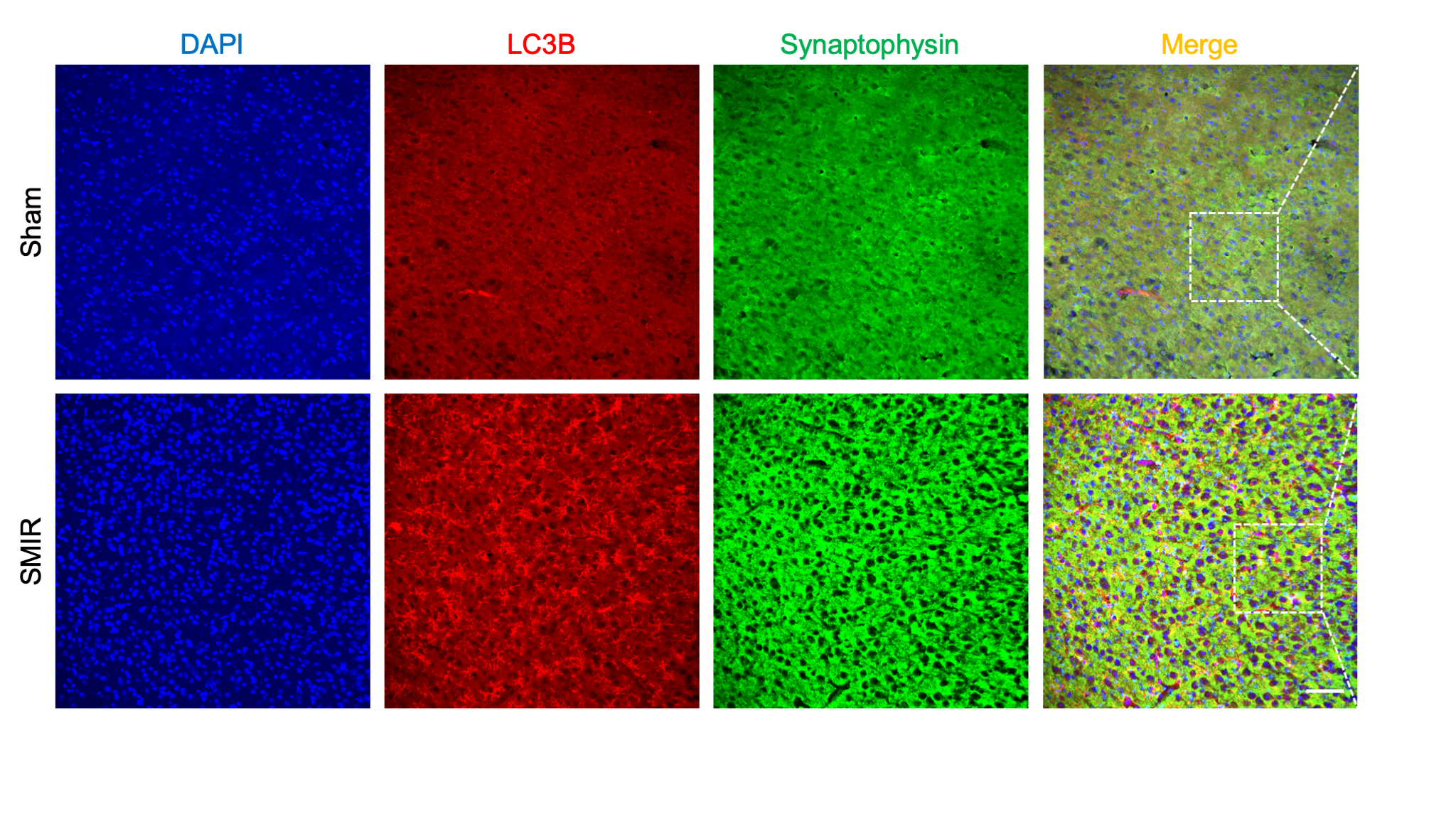

Supplement: Supplementary file 4 — Source data Fig. 2 [file 44319_2025_646_MOESM4_ESM.zip › Figure 2/2E/2E.tiff]

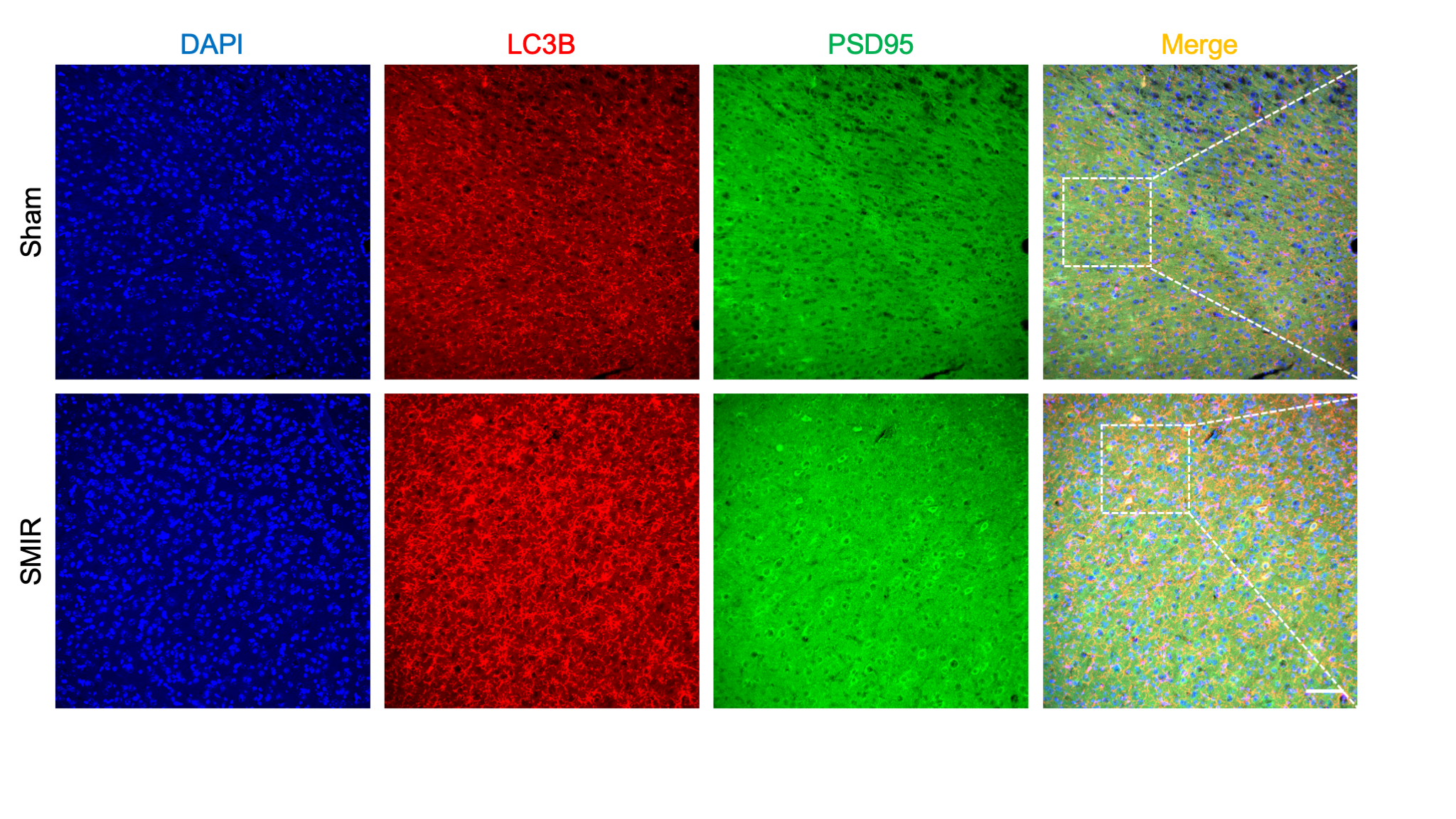

Supplement: Supplementary file 4 — Source data Fig. 2 [file 44319_2025_646_MOESM4_ESM.zip › Figure 2/2F/2F.tiff]

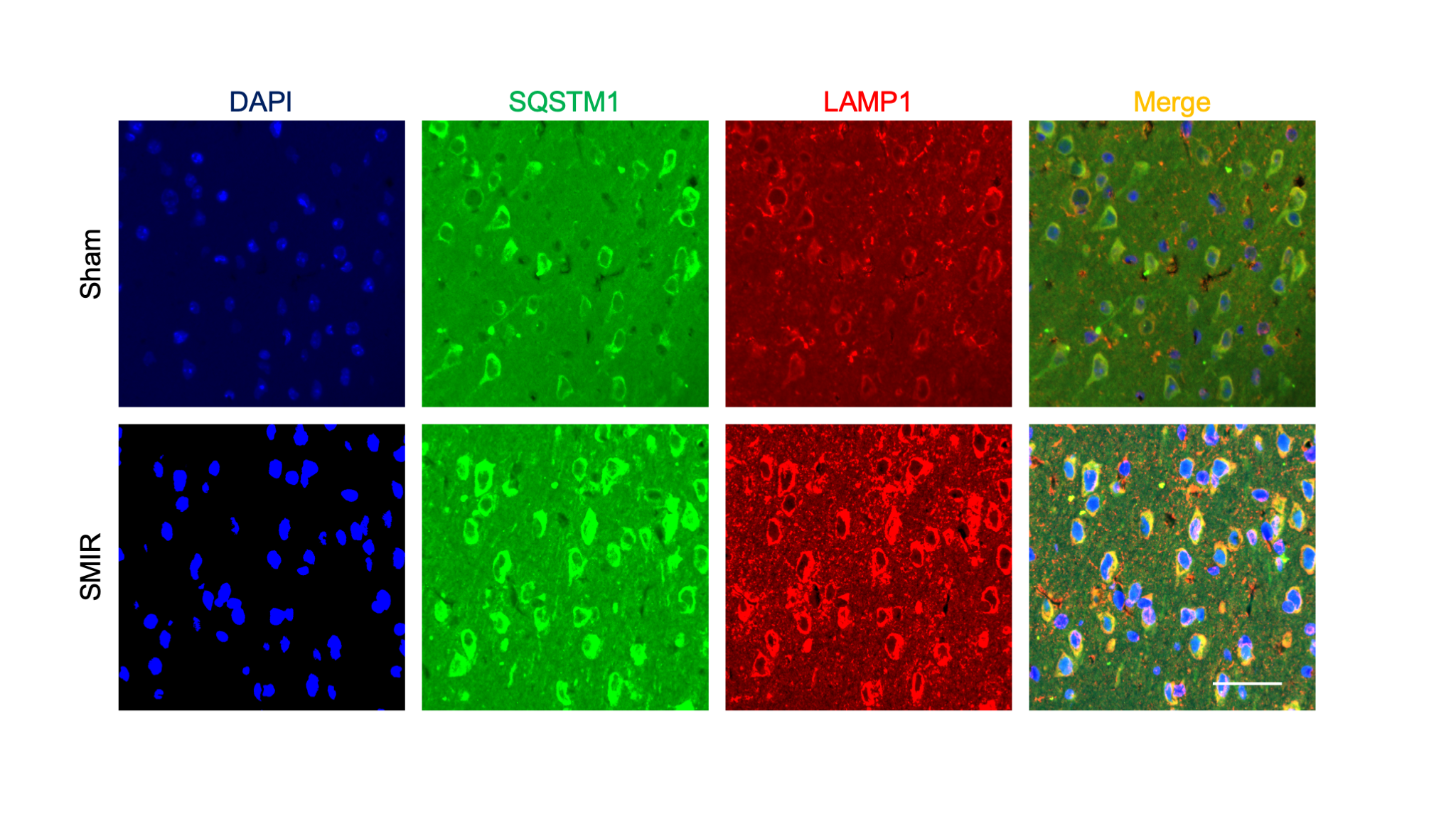

Supplement: Supplementary file 5 — Source data Fig. 3 [file 44319_2025_646_MOESM5_ESM.zip › Figure 3/3A/3A.tiff]

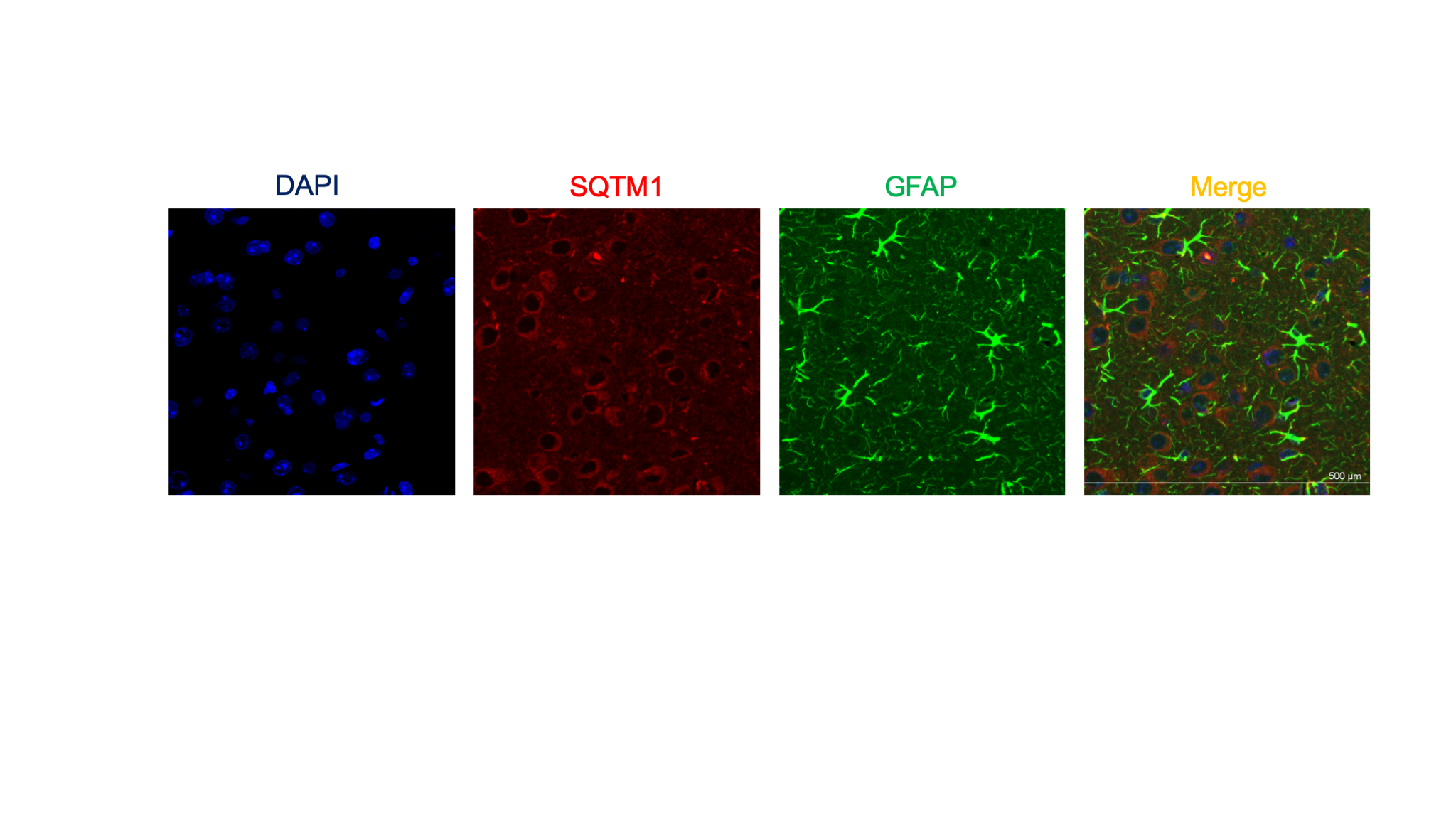

Supplement: Supplementary file 5 — Source data Fig. 3 [file 44319_2025_646_MOESM5_ESM.zip › Figure 3/3B/3B-GFAP.tiff]

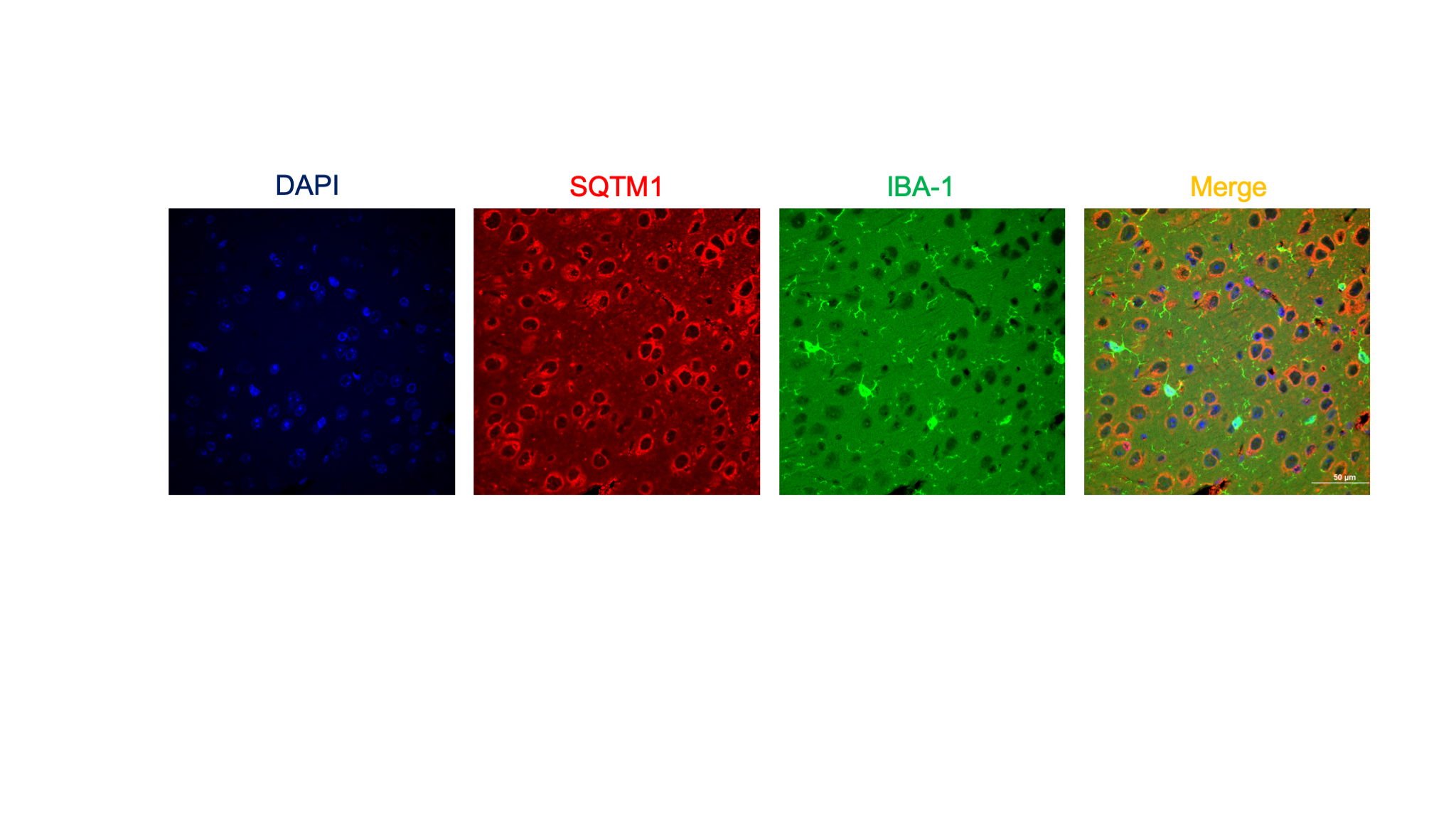

Supplement: Supplementary file 5 — Source data Fig. 3 [file 44319_2025_646_MOESM5_ESM.zip › Figure 3/3B/3B-IBA-1.tiff]

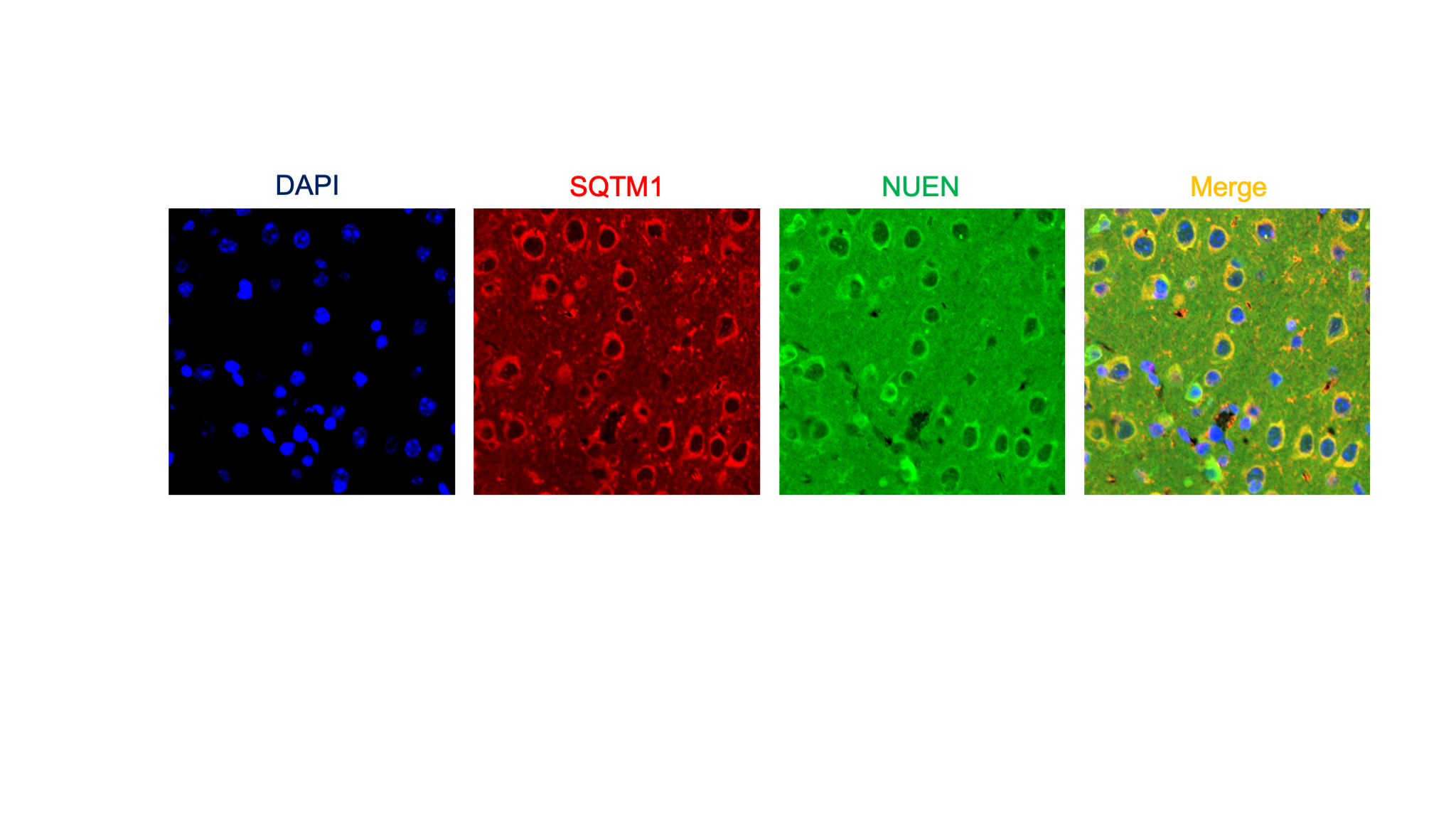

Supplement: Supplementary file 5 — Source data Fig. 3 [file 44319_2025_646_MOESM5_ESM.zip › Figure 3/3B/3B-NEUN.tiff]

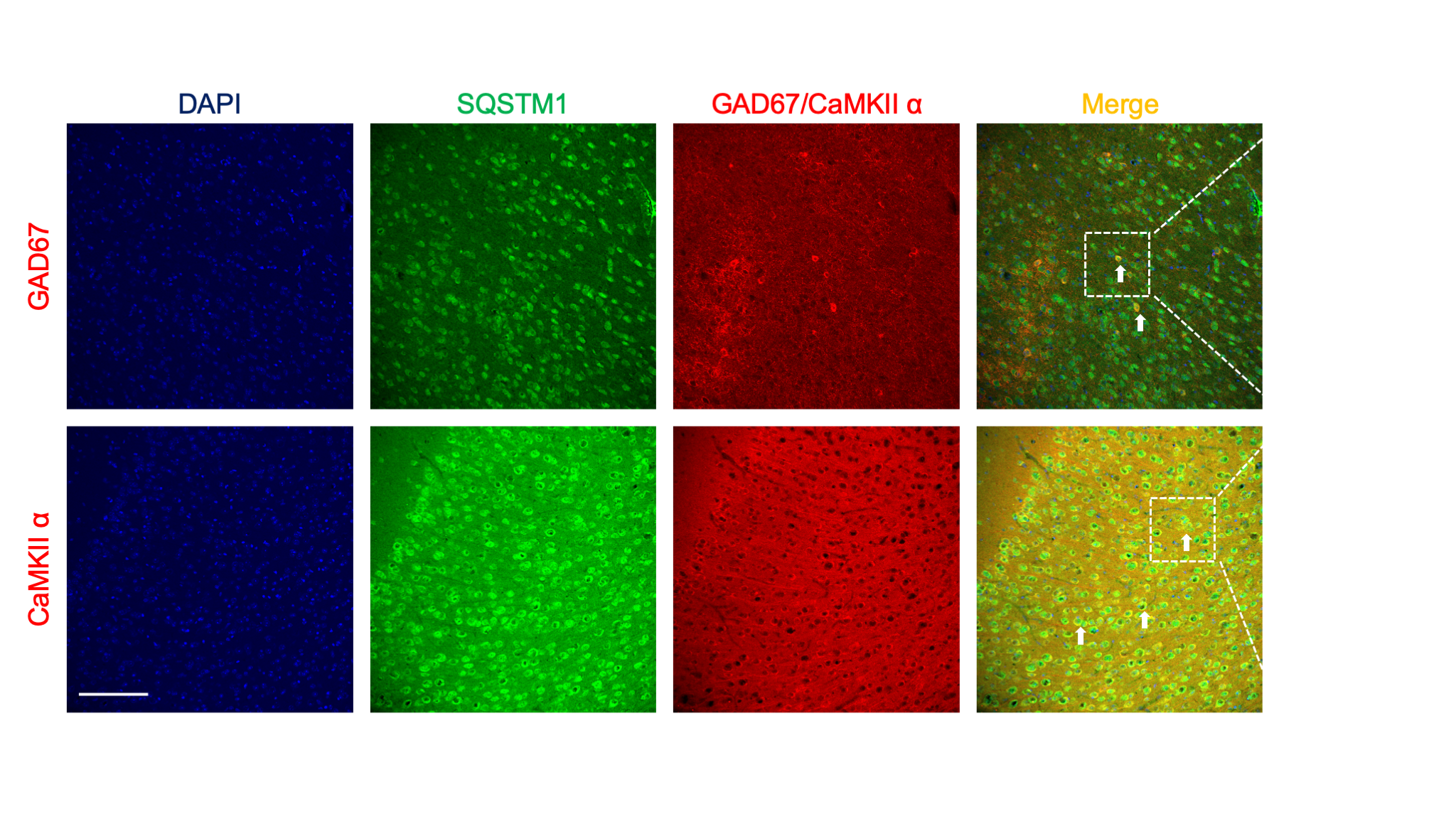

Supplement: Supplementary file 5 — Source data Fig. 3 [file 44319_2025_646_MOESM5_ESM.zip › Figure 3/3C/3C.tiff]

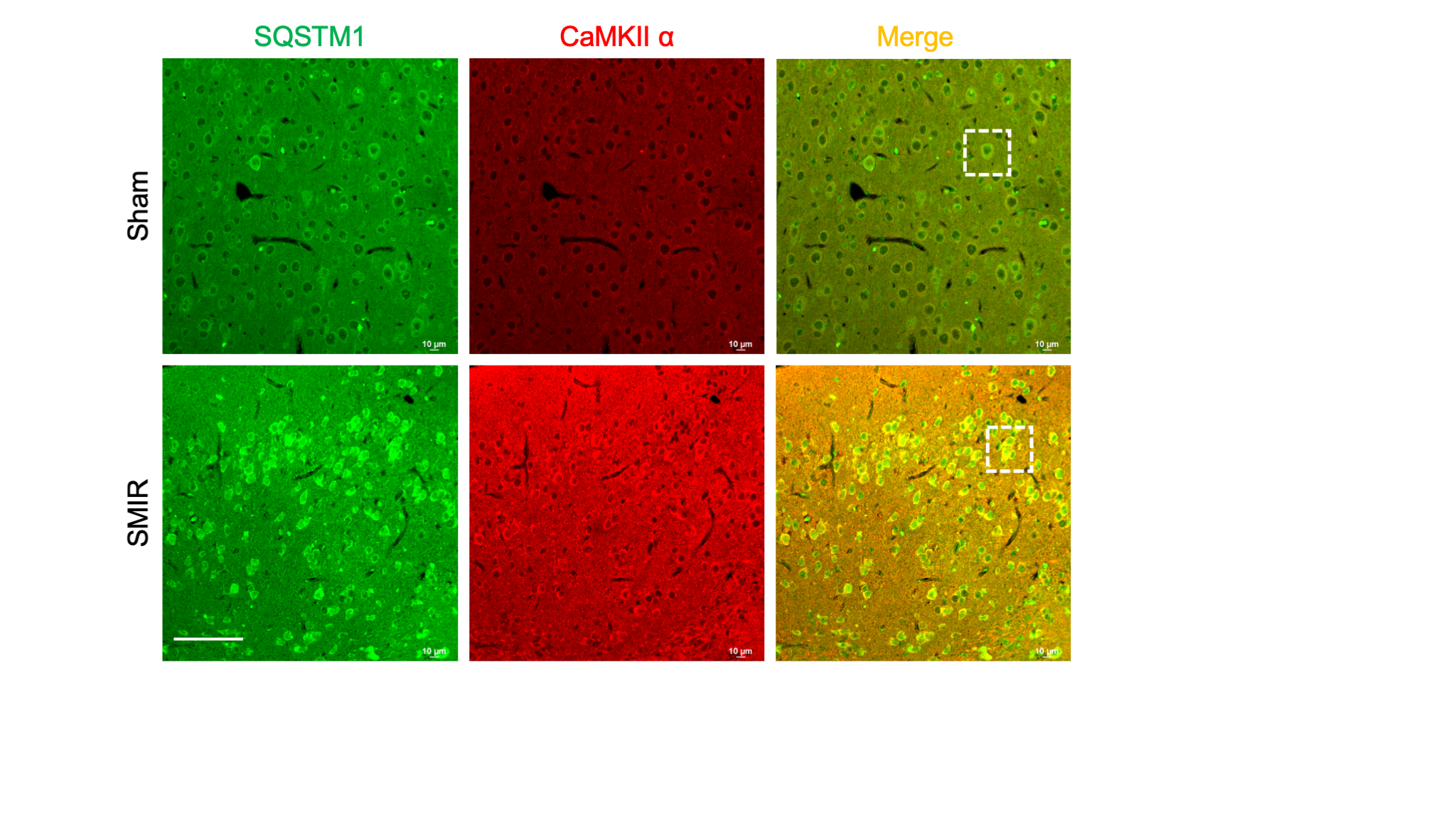

Supplement: Supplementary file 5 — Source data Fig. 3 [file 44319_2025_646_MOESM5_ESM.zip › Figure 3/3D/3D.tiff]

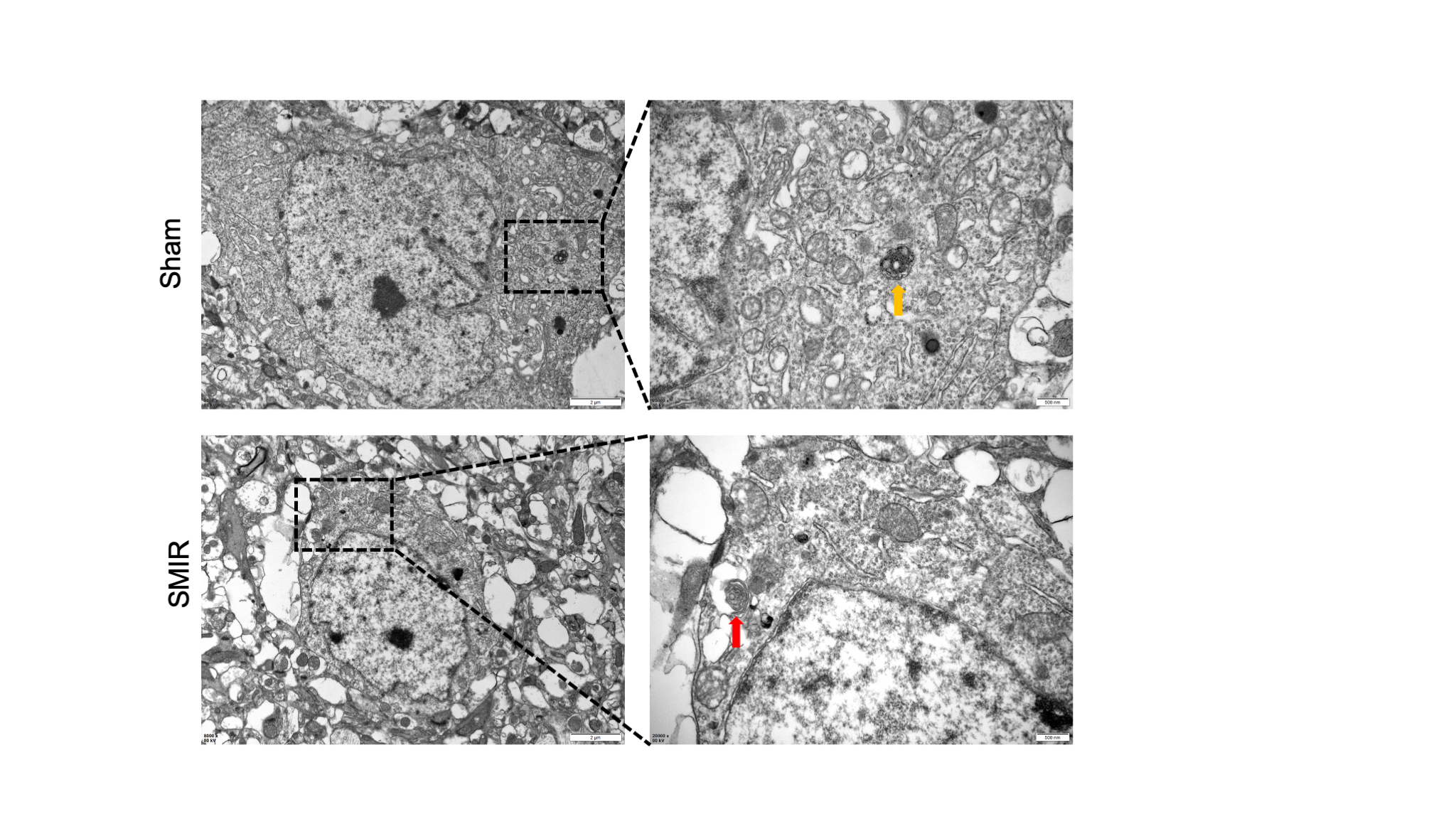

Supplement: Supplementary file 5 — Source data Fig. 3 [file 44319_2025_646_MOESM5_ESM.zip › Figure 3/3E/3E.tiff]

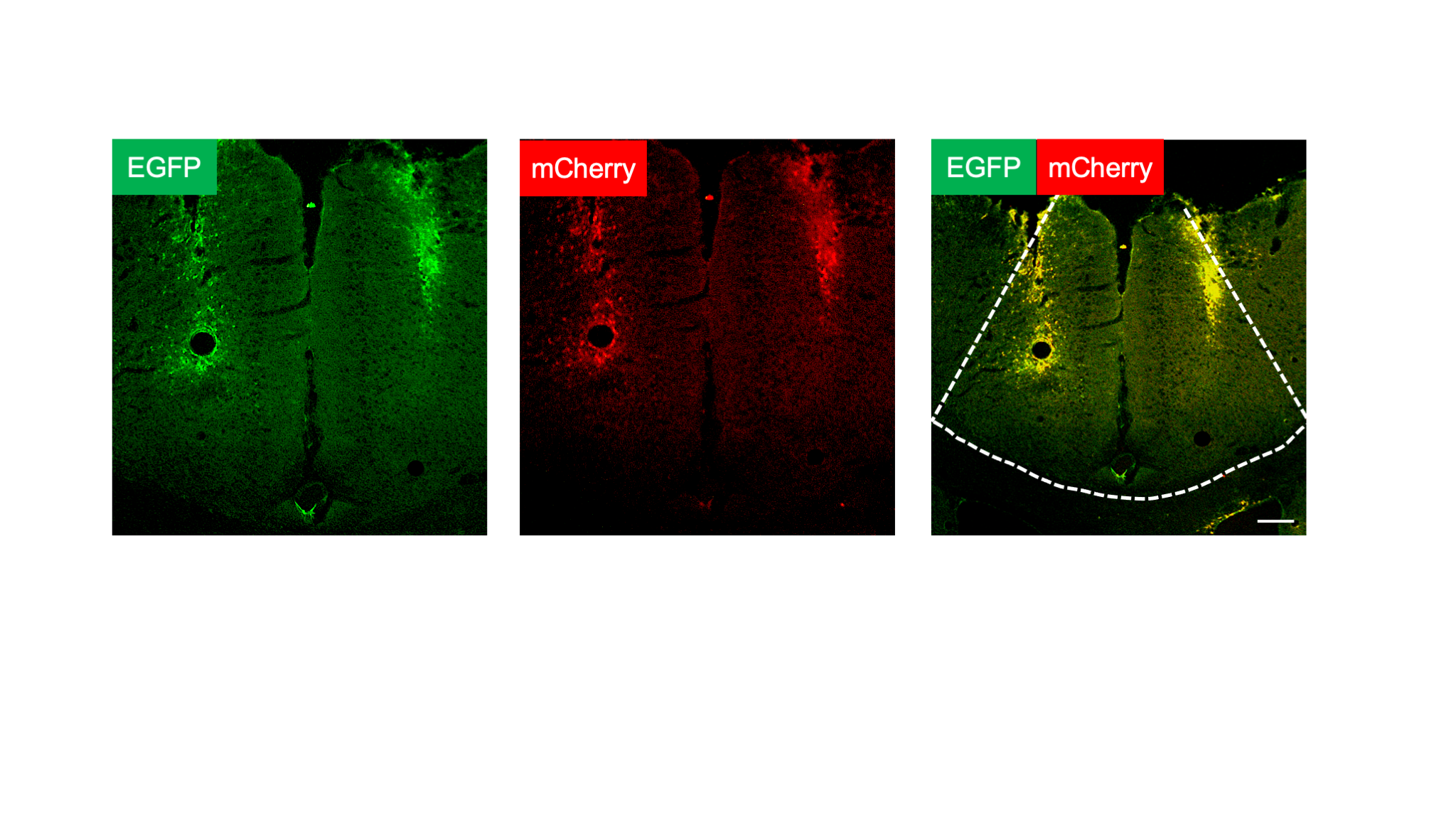

Supplement: Supplementary file 5 — Source data Fig. 3 [file 44319_2025_646_MOESM5_ESM.zip › Figure 3/3G/3G.tiff]

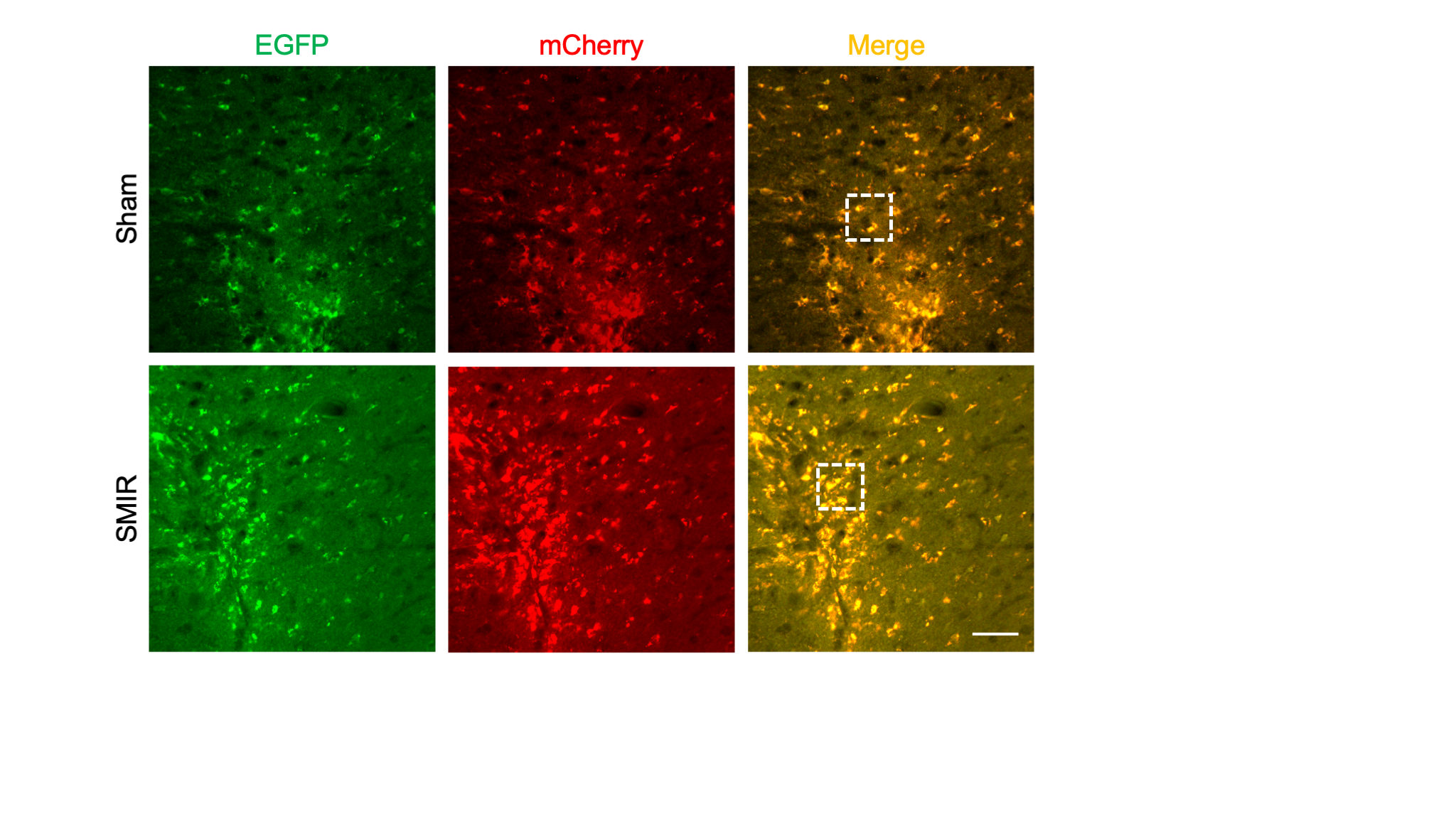

Supplement: Supplementary file 5 — Source data Fig. 3 [file 44319_2025_646_MOESM5_ESM.zip › Figure 3/3H/3H.tiff]

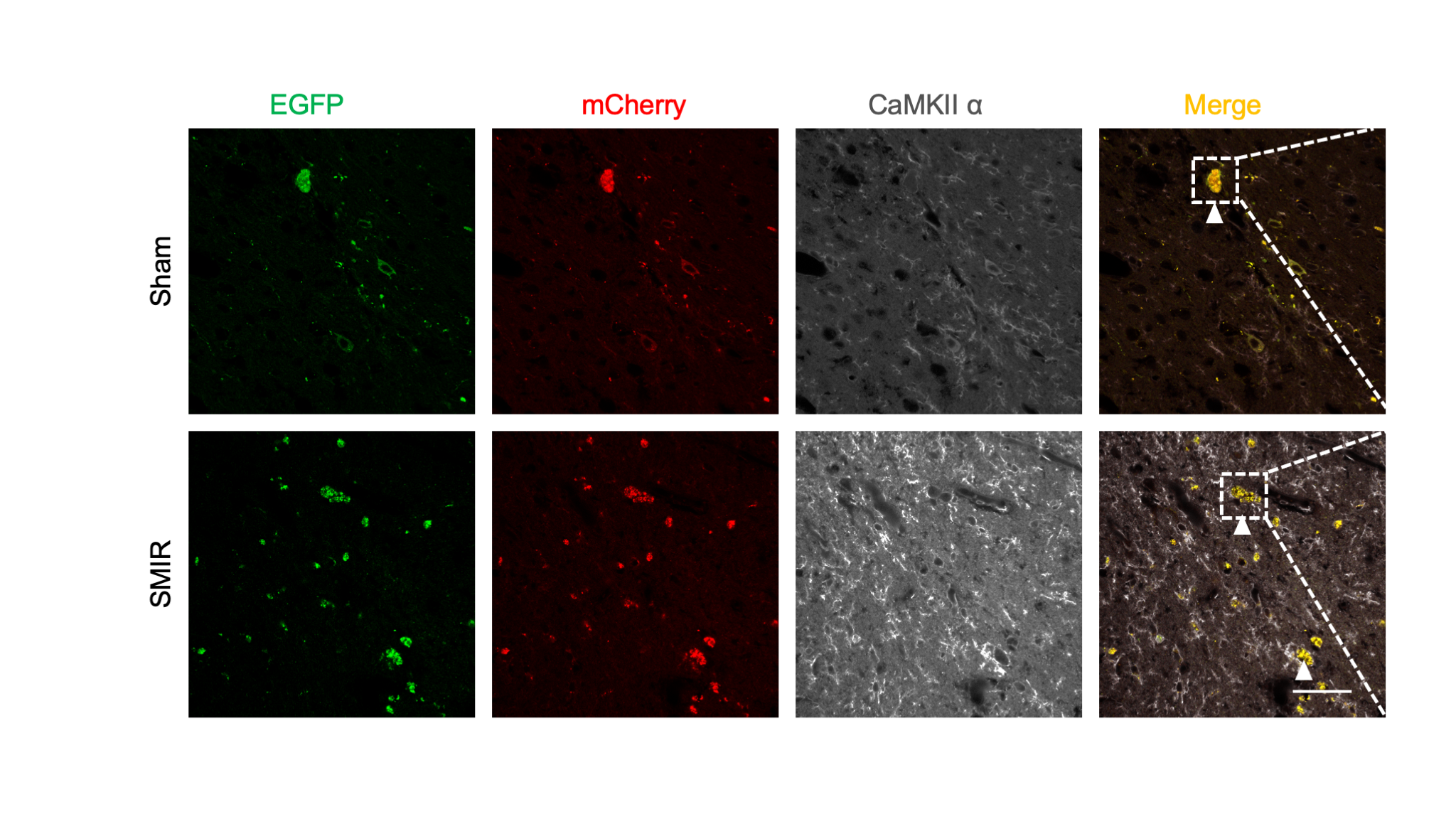

Supplement: Supplementary file 5 — Source data Fig. 3 [file 44319_2025_646_MOESM5_ESM.zip › Figure 3/3J/3J-left.tiff]

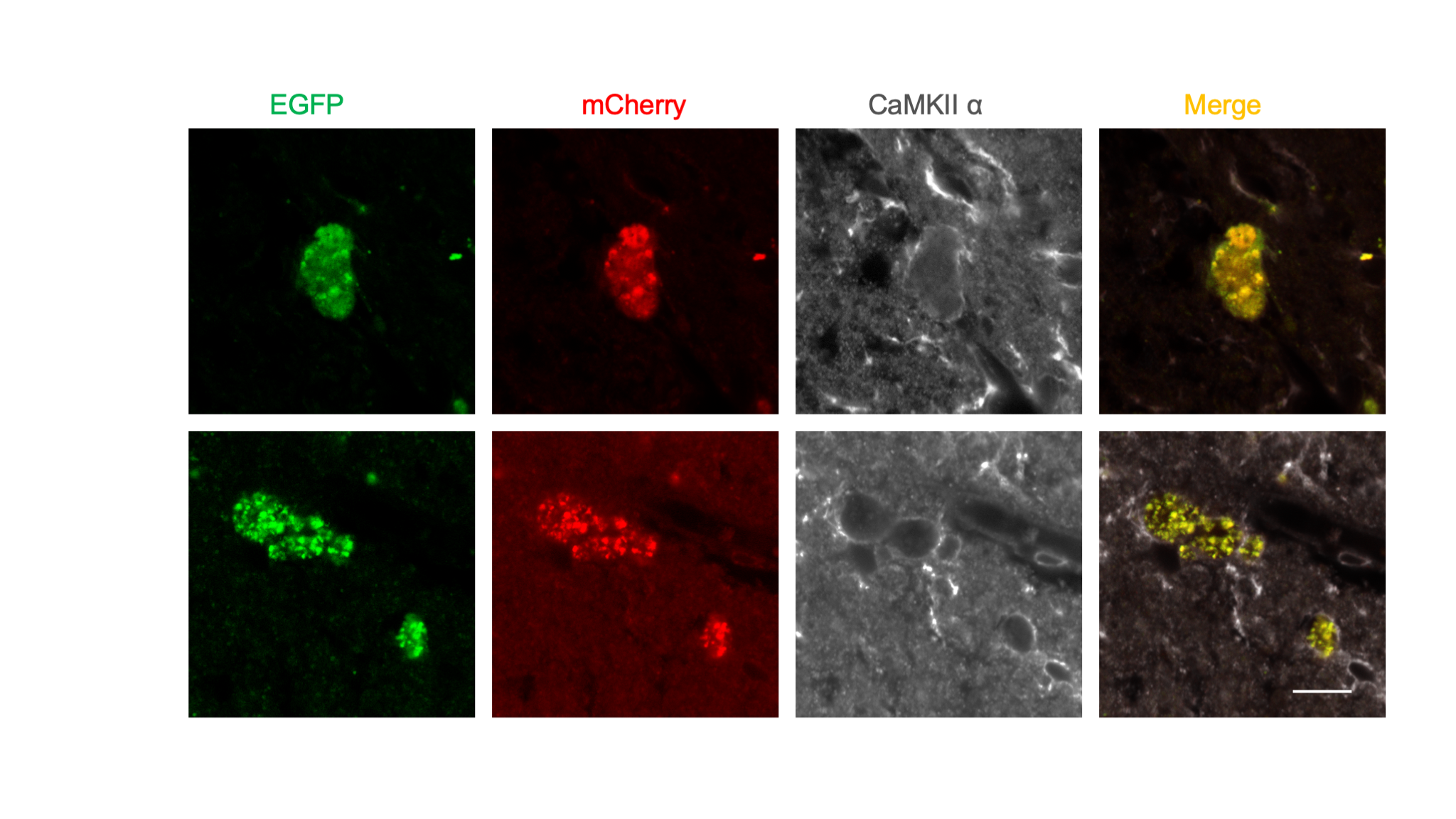

Supplement: Supplementary file 5 — Source data Fig. 3 [file 44319_2025_646_MOESM5_ESM.zip › Figure 3/3J/3J-right.tiff]

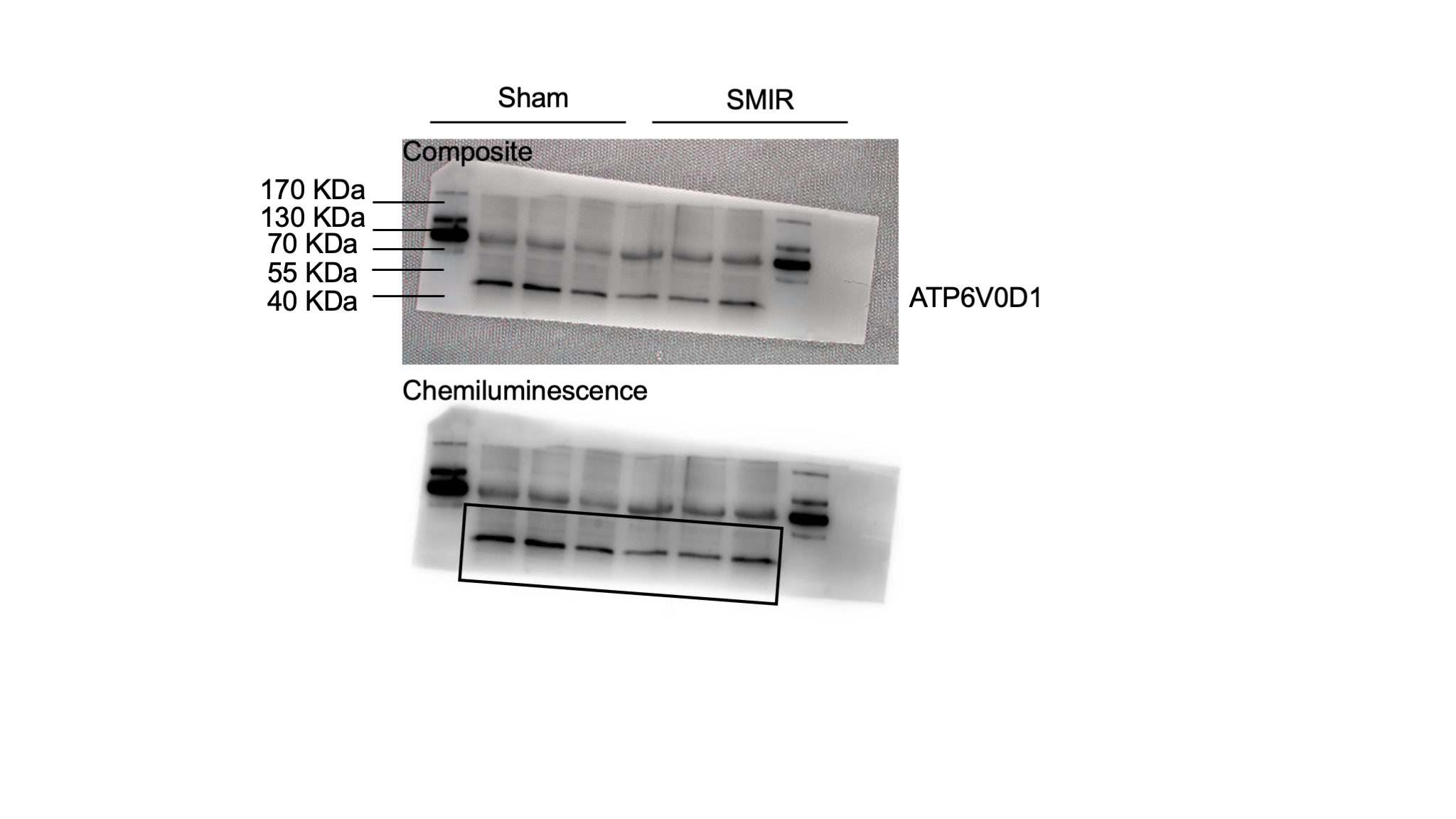

Supplement: Supplementary file 6 — Source data Fig. 4 [file 44319_2025_646_MOESM6_ESM.zip › Figure 4/4A/4A-ATP6V0D1.tiff]

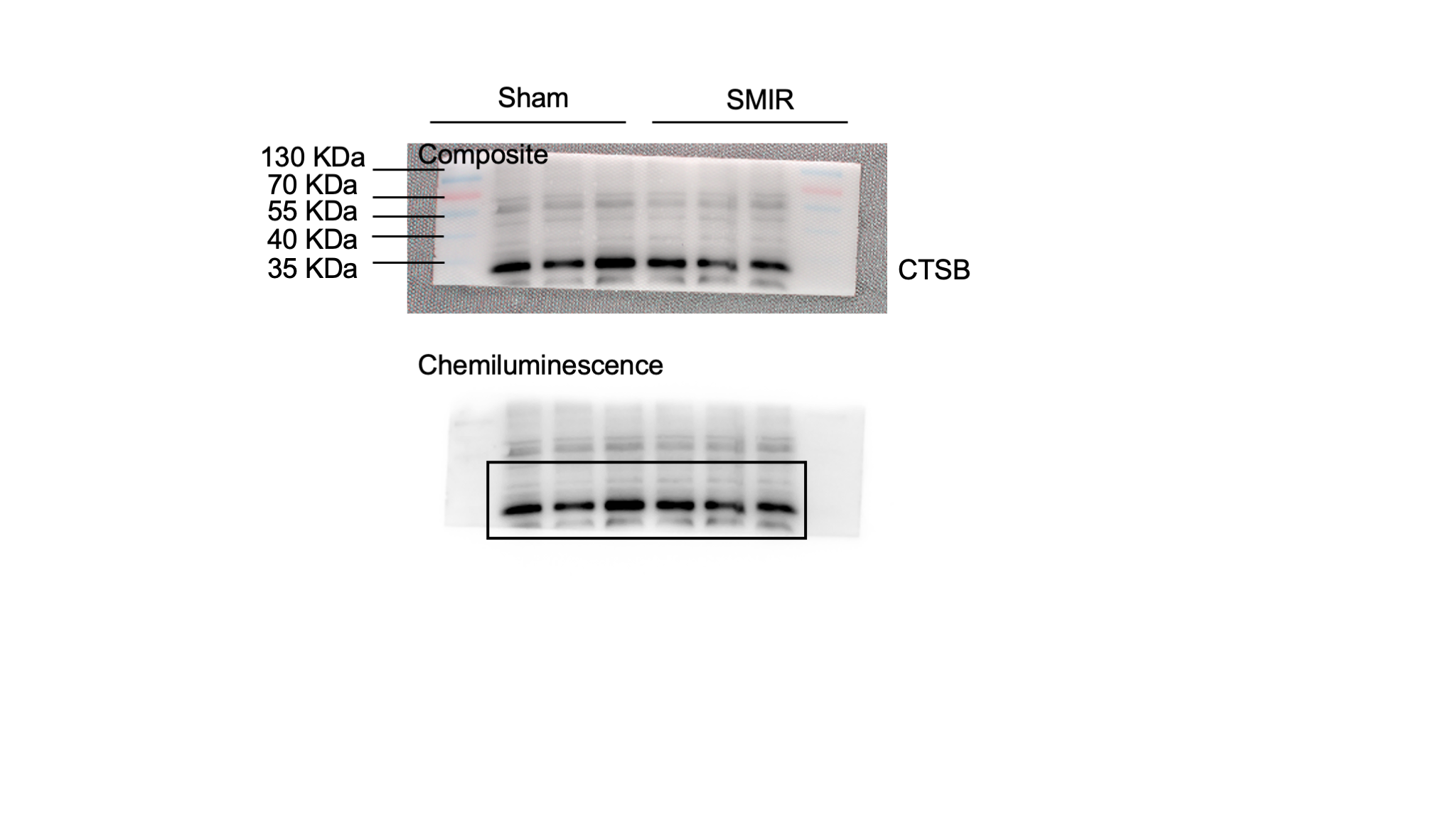

Supplement: Supplementary file 6 — Source data Fig. 4 [file 44319_2025_646_MOESM6_ESM.zip › Figure 4/4A/4A-CTSB.tiff]

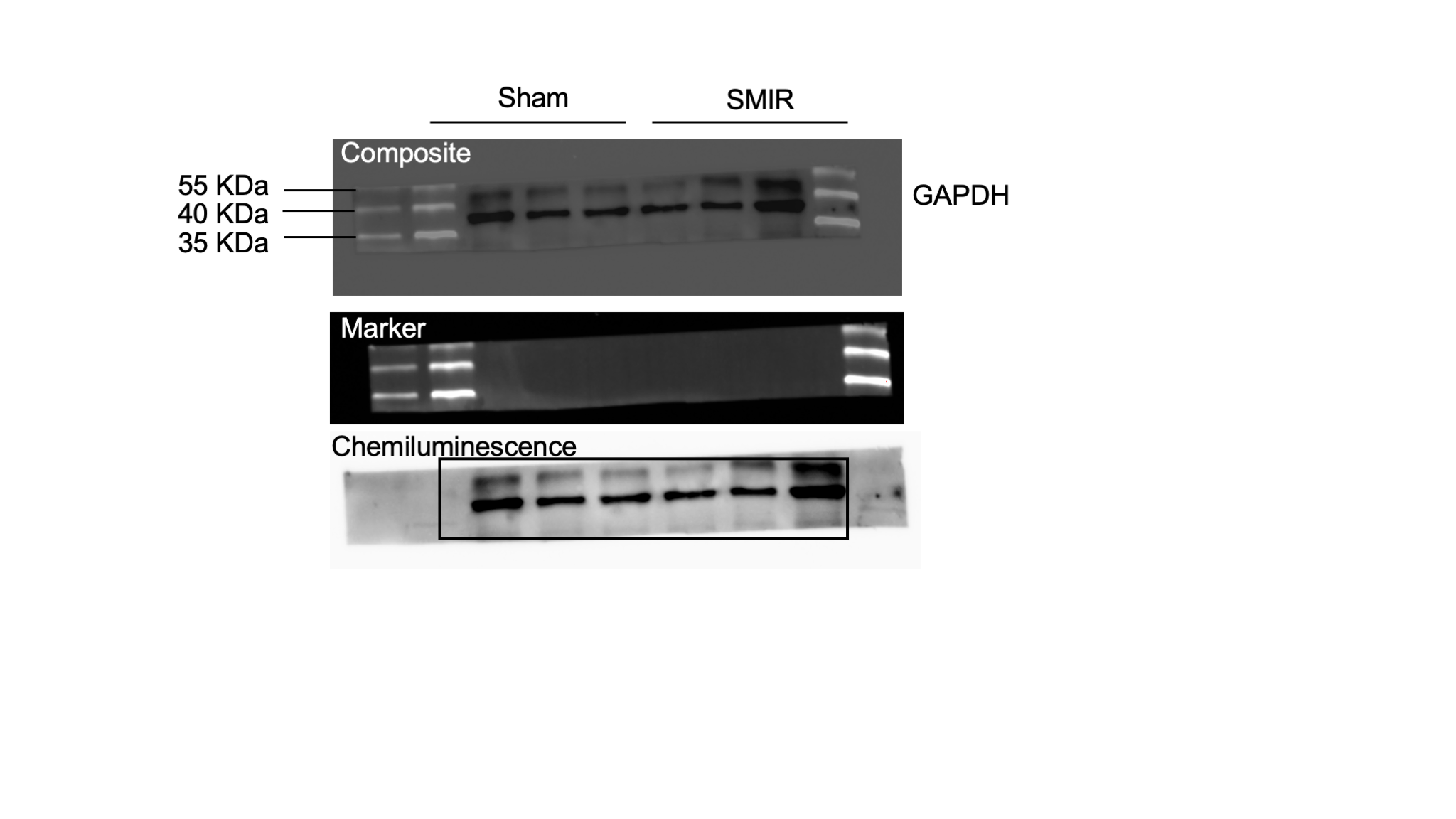

Supplement: Supplementary file 6 — Source data Fig. 4 [file 44319_2025_646_MOESM6_ESM.zip › Figure 4/4A/4A-GAPDH.tiff]

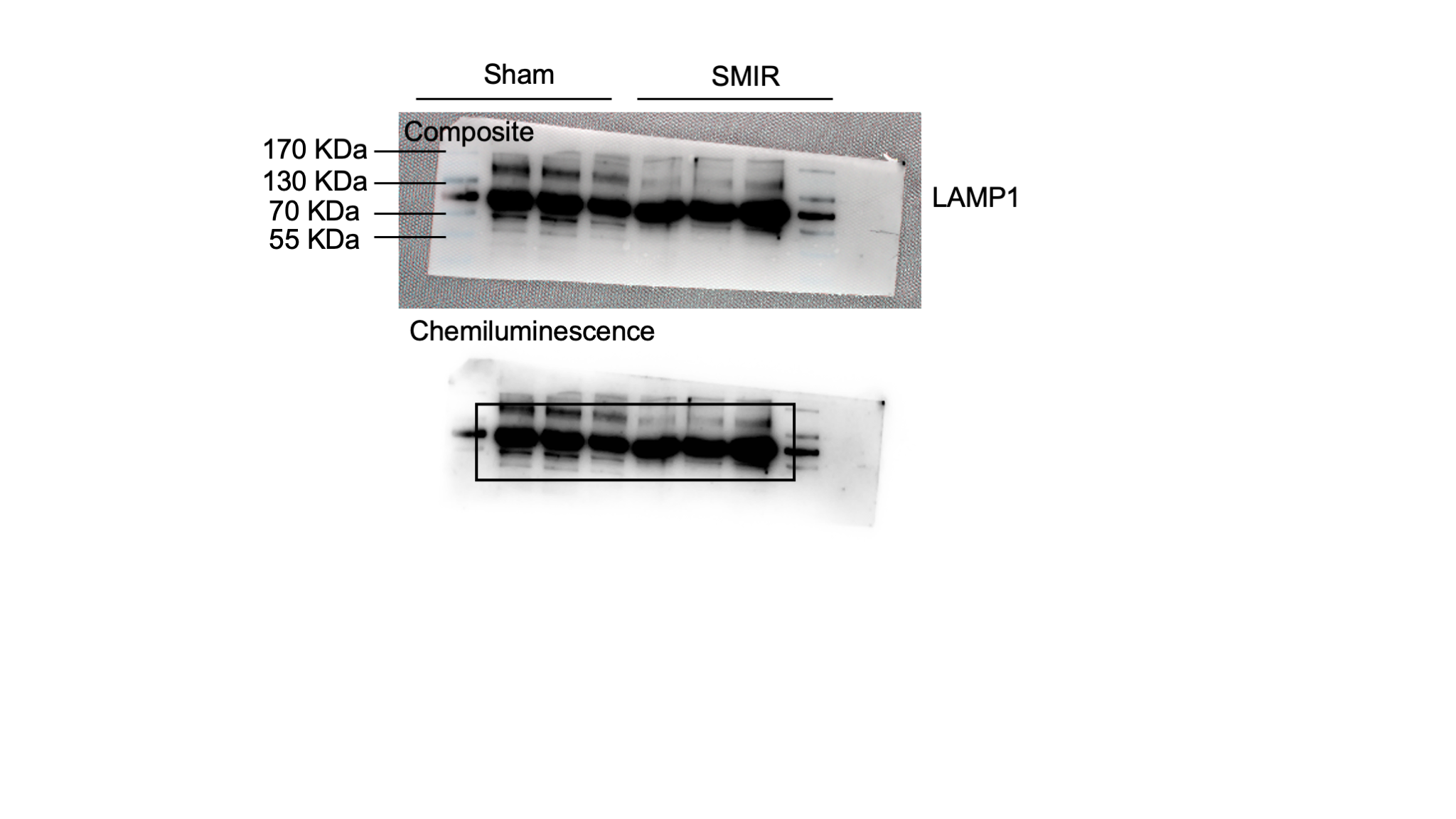

Supplement: Supplementary file 6 — Source data Fig. 4 [file 44319_2025_646_MOESM6_ESM.zip › Figure 4/4A/4A-LAMP1.tiff]

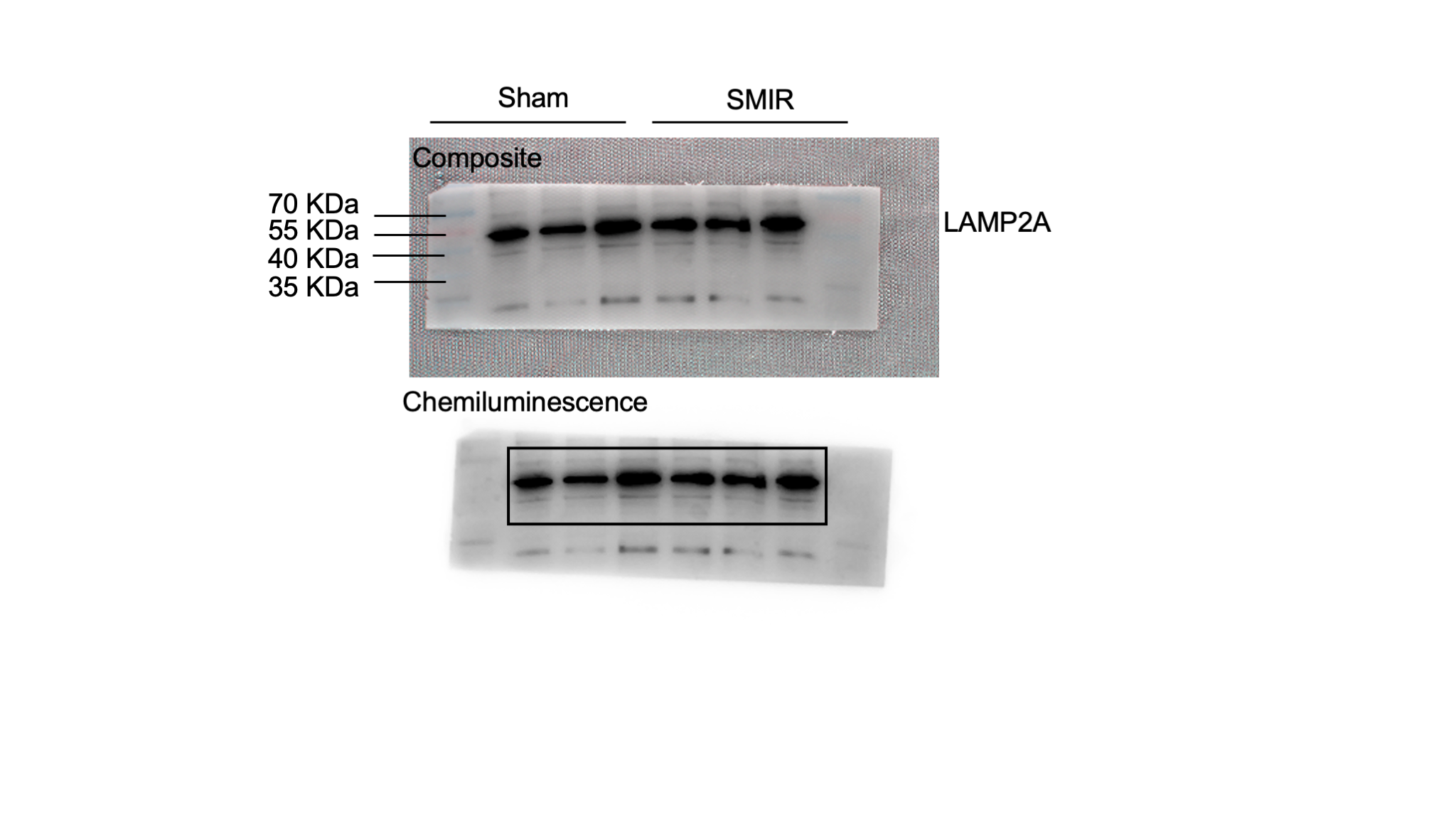

Supplement: Supplementary file 6 — Source data Fig. 4 [file 44319_2025_646_MOESM6_ESM.zip › Figure 4/4A/4A-LAMP2A.tiff]

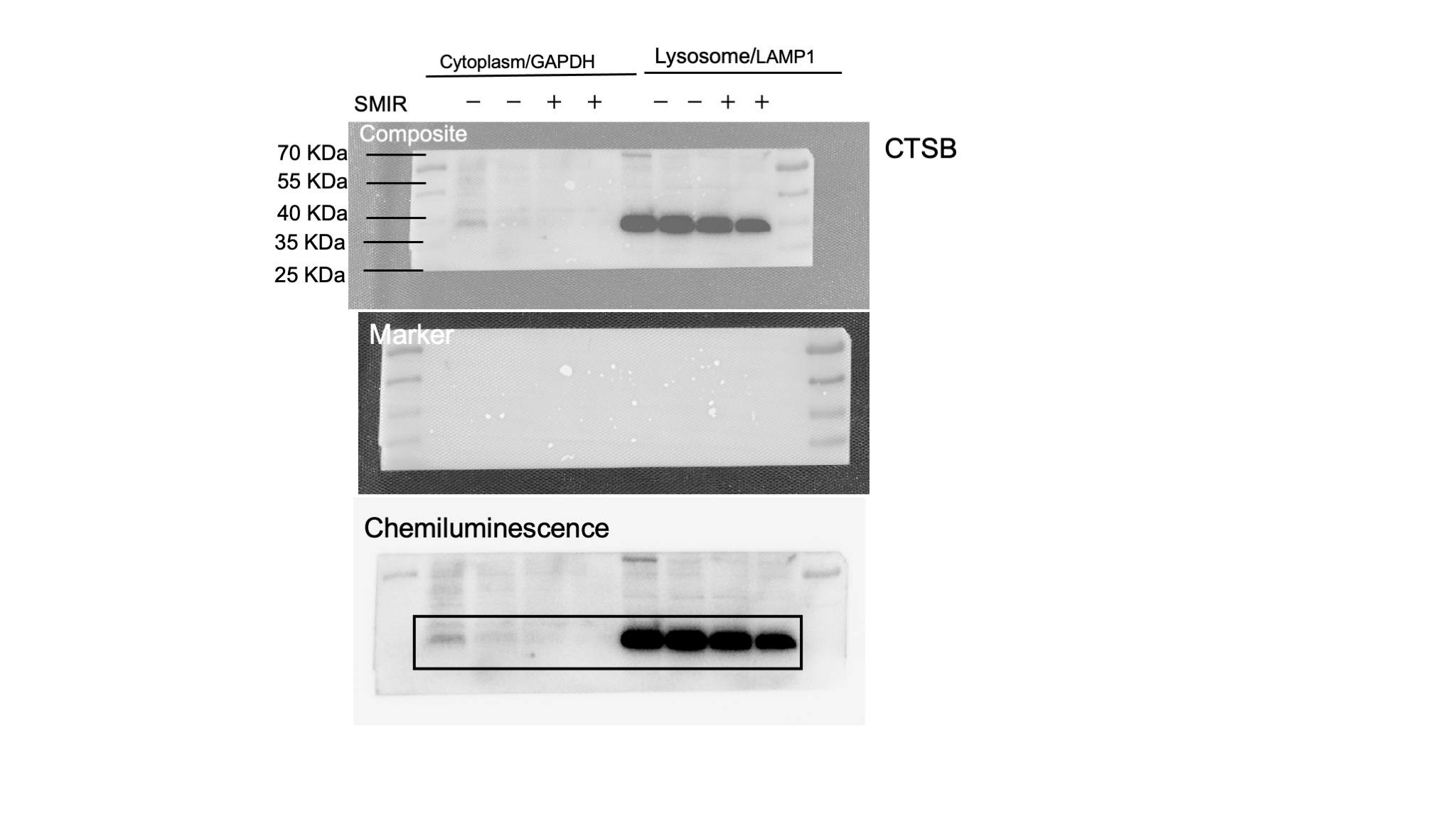

Supplement: Supplementary file 6 — Source data Fig. 4 [file 44319_2025_646_MOESM6_ESM.zip › Figure 4/4B/4B-CTSB.tiff]

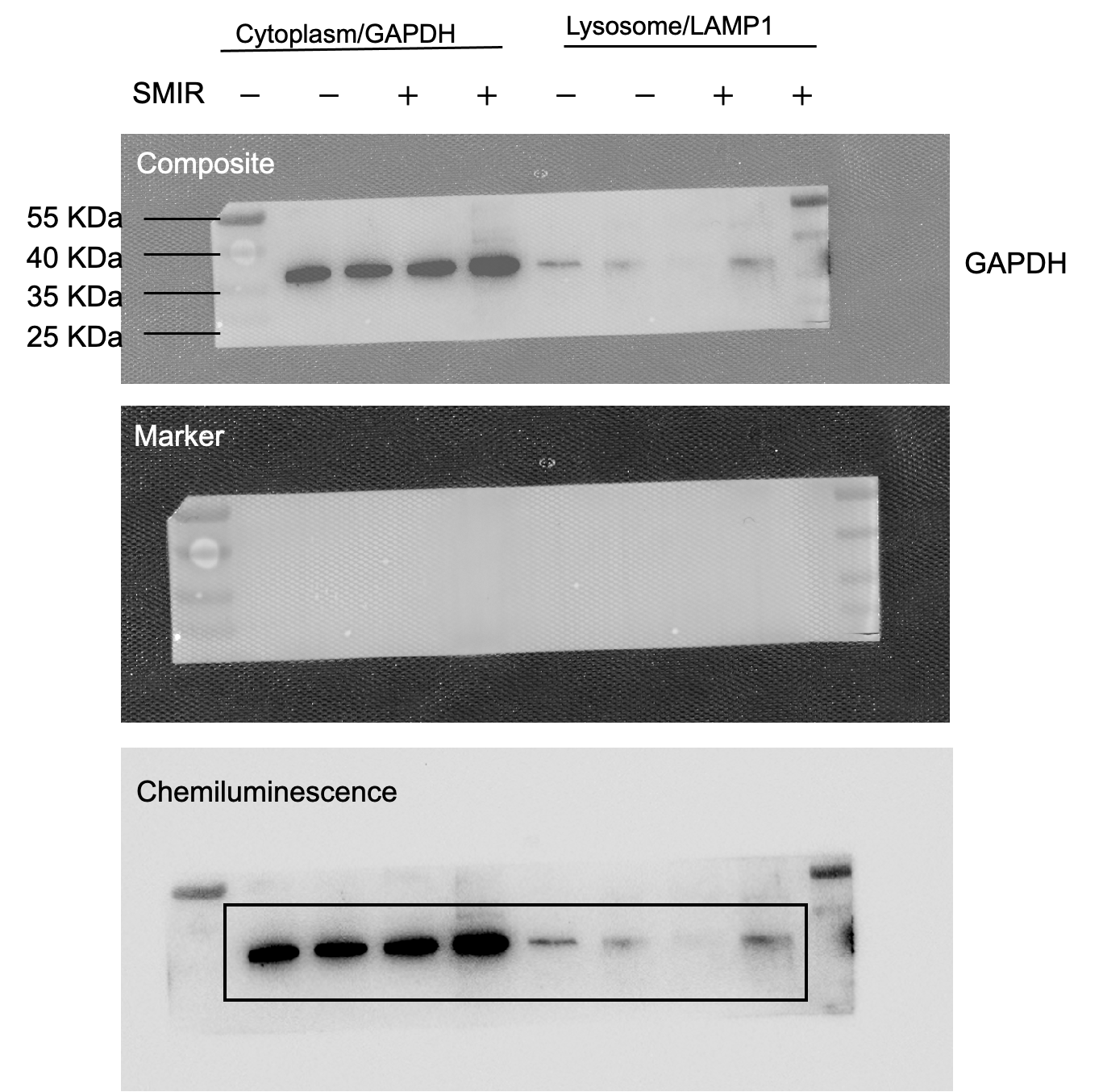

Supplement: Supplementary file 6 — Source data Fig. 4 [file 44319_2025_646_MOESM6_ESM.zip › Figure 4/4B/4B-GAPDH.png]

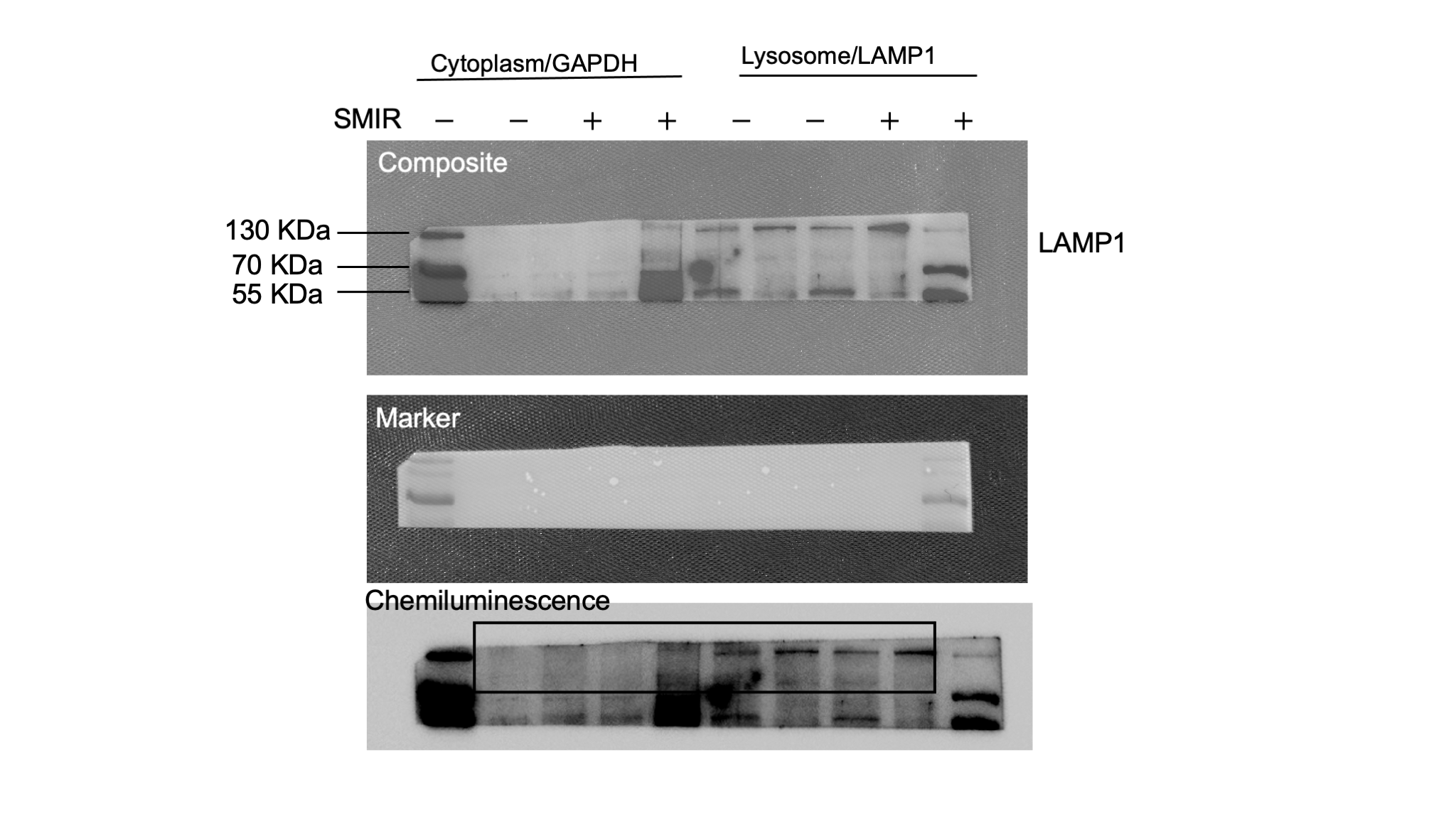

Supplement: Supplementary file 6 — Source data Fig. 4 [file 44319_2025_646_MOESM6_ESM.zip › Figure 4/4B/4B-LAMP1.tiff]

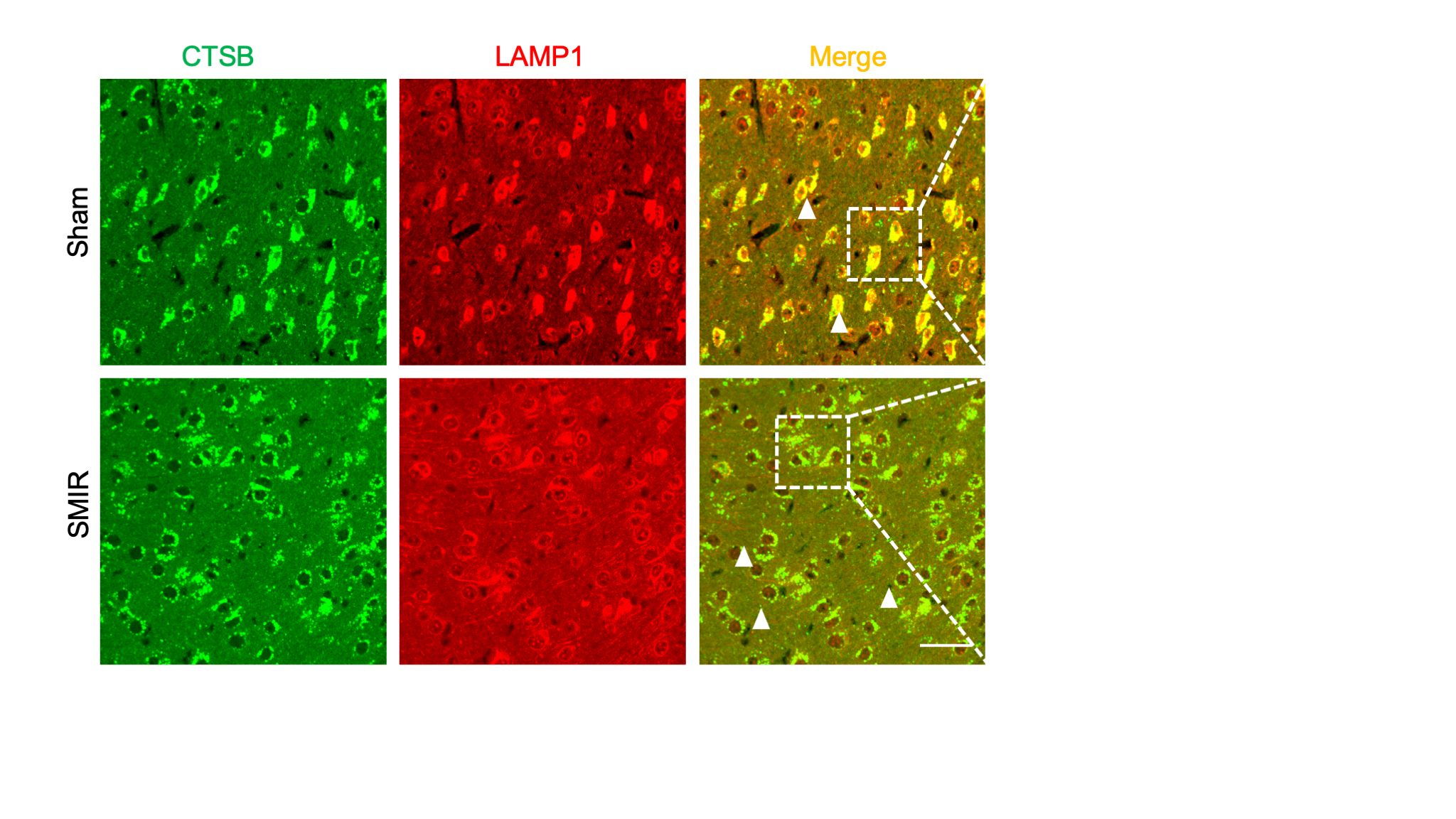

Supplement: Supplementary file 6 — Source data Fig. 4 [file 44319_2025_646_MOESM6_ESM.zip › Figure 4/4C/4C.tiff]

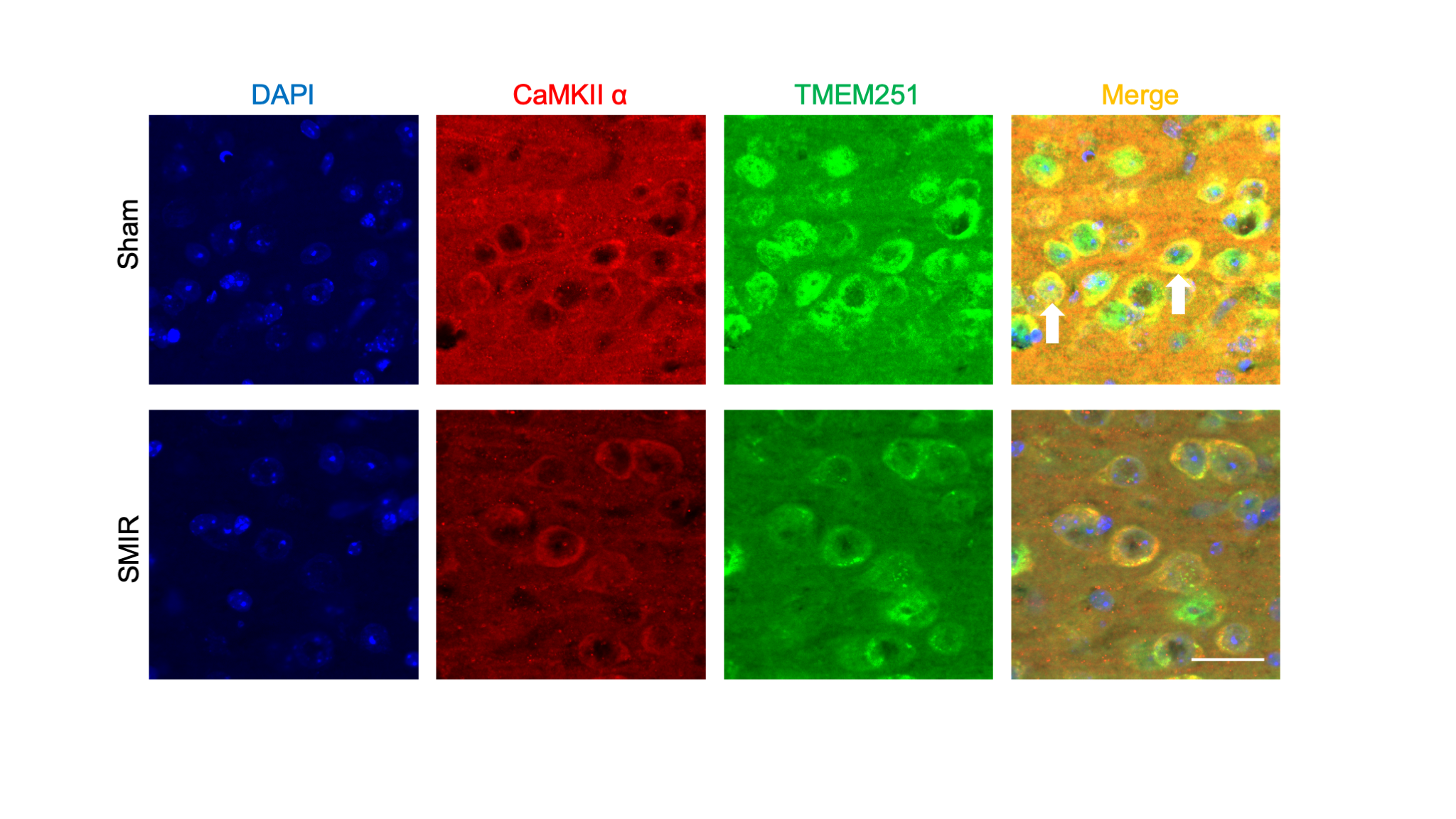

Supplement: Supplementary file 6 — Source data Fig. 4 [file 44319_2025_646_MOESM6_ESM.zip › Figure 4/4E/4E.tiff]

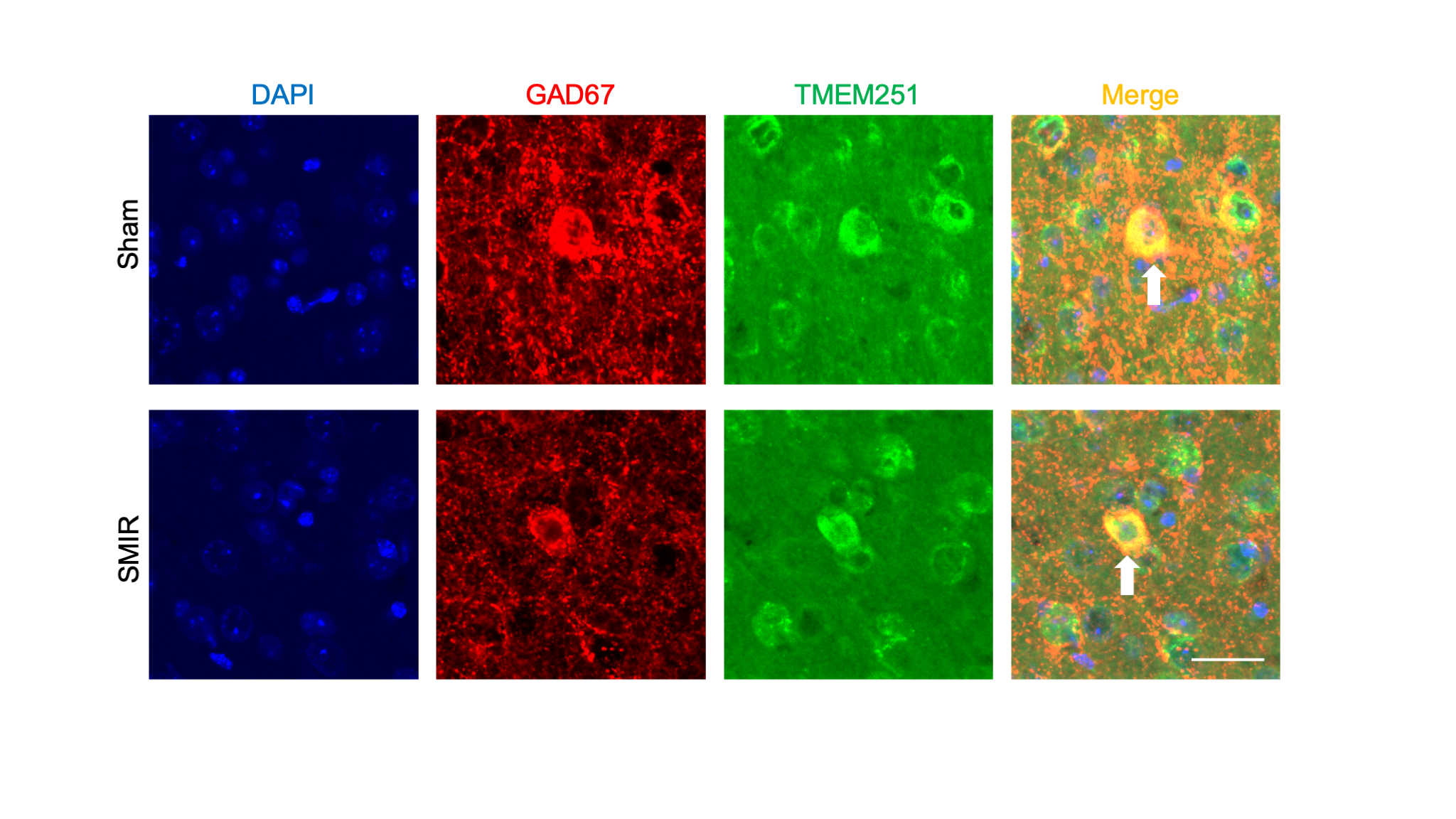

Supplement: Supplementary file 6 — Source data Fig. 4 [file 44319_2025_646_MOESM6_ESM.zip › Figure 4/4F/4F.tiff]

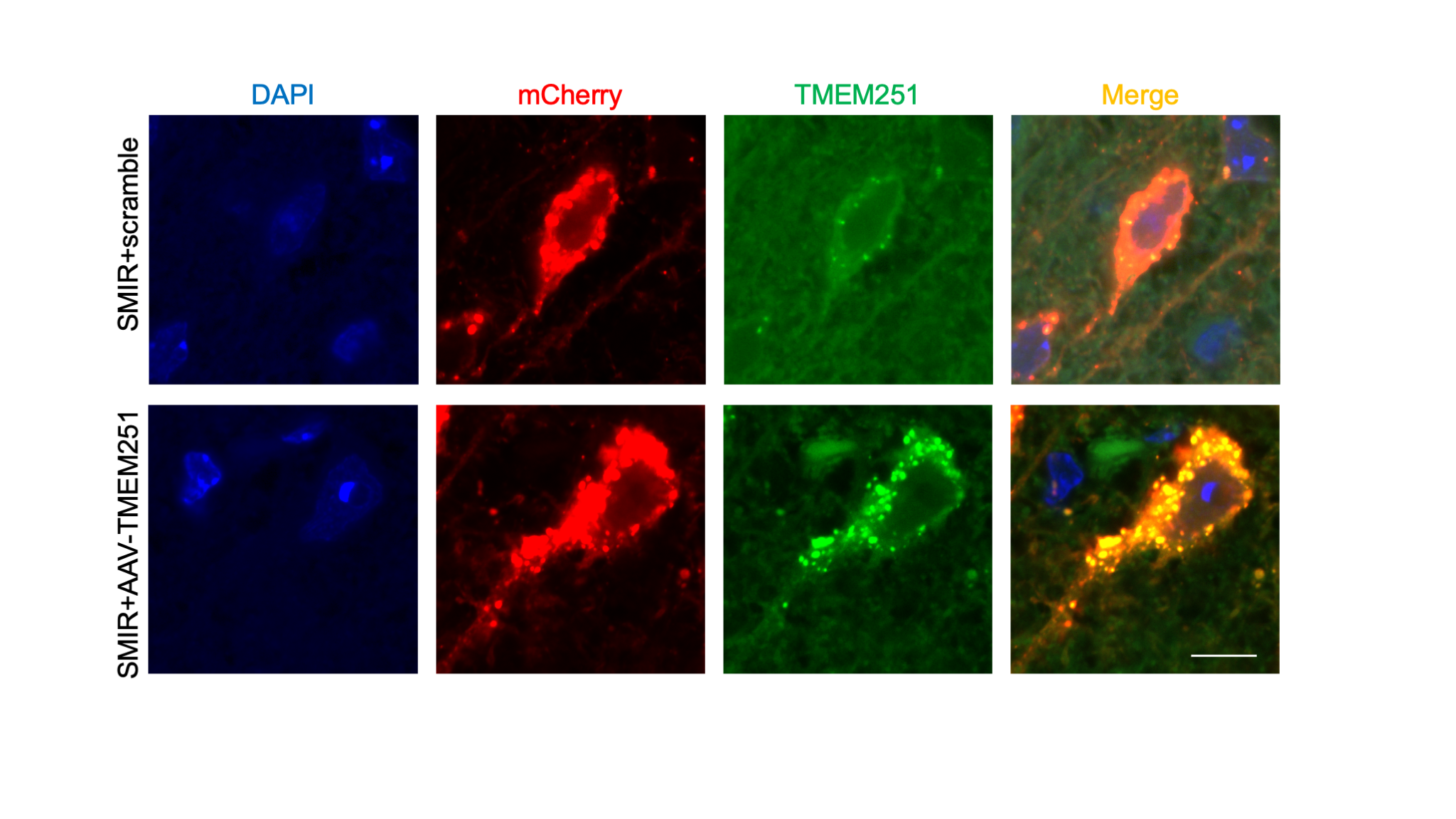

Supplement: Supplementary file 7 — Source data Fig. 5 [file 44319_2025_646_MOESM7_ESM.zip › Figure 5/5B/5B.tiff]

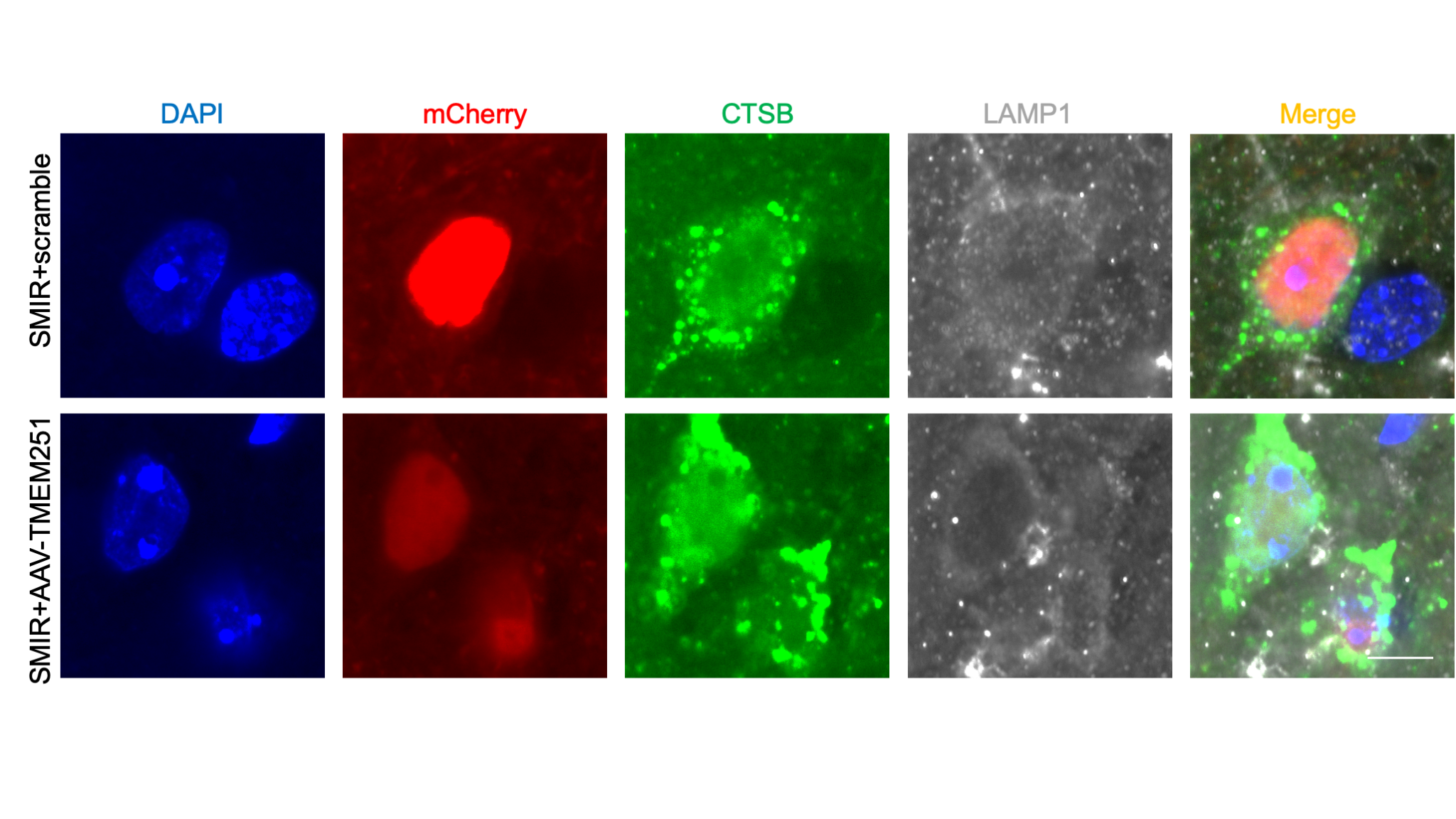

Supplement: Supplementary file 7 — Source data Fig. 5 [file 44319_2025_646_MOESM7_ESM.zip › Figure 5/5D/5D.tiff]

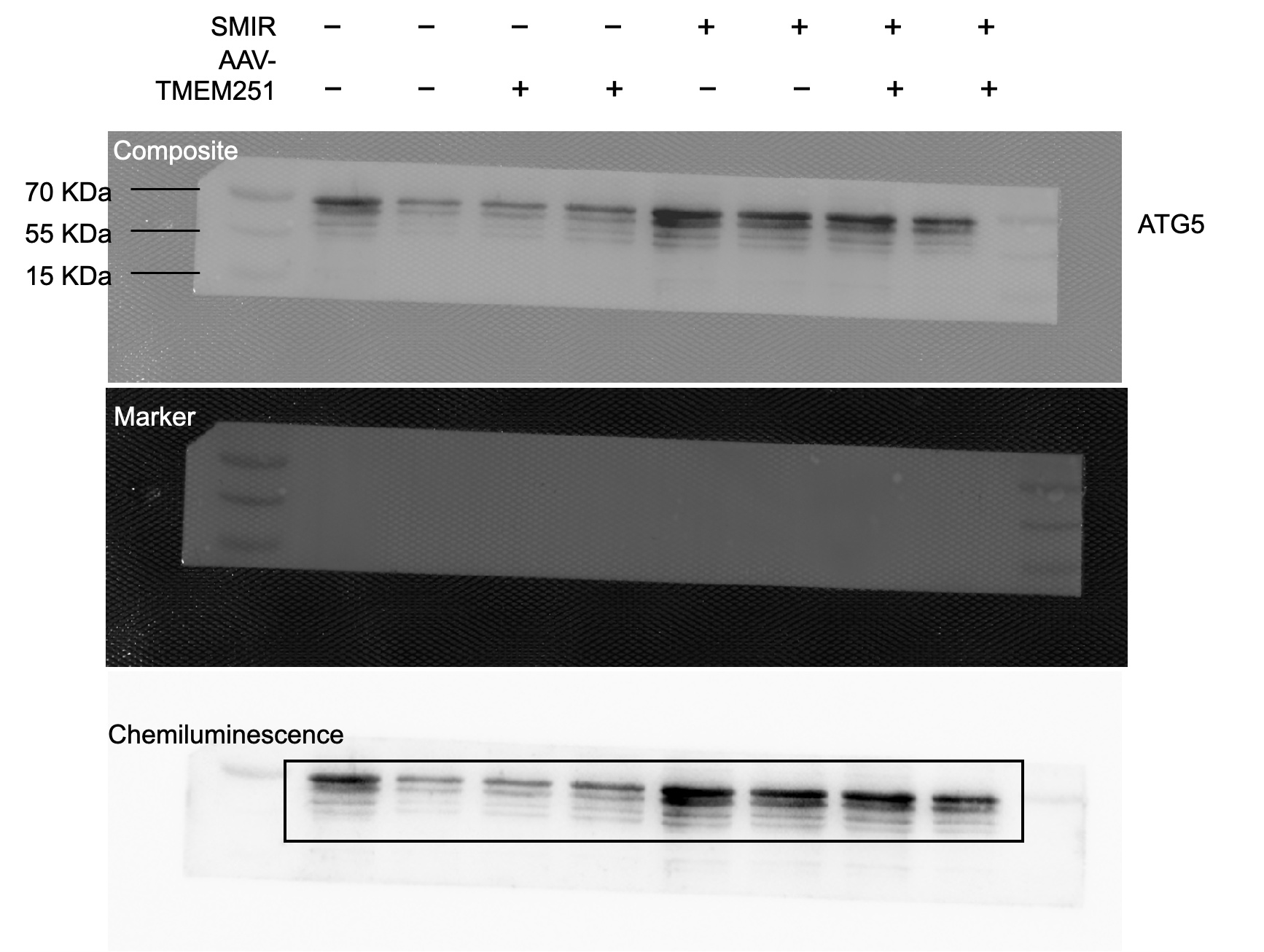

Supplement: Supplementary file 7 — Source data Fig. 5 [file 44319_2025_646_MOESM7_ESM.zip › Figure 5/5G/5G-ATG5.png]

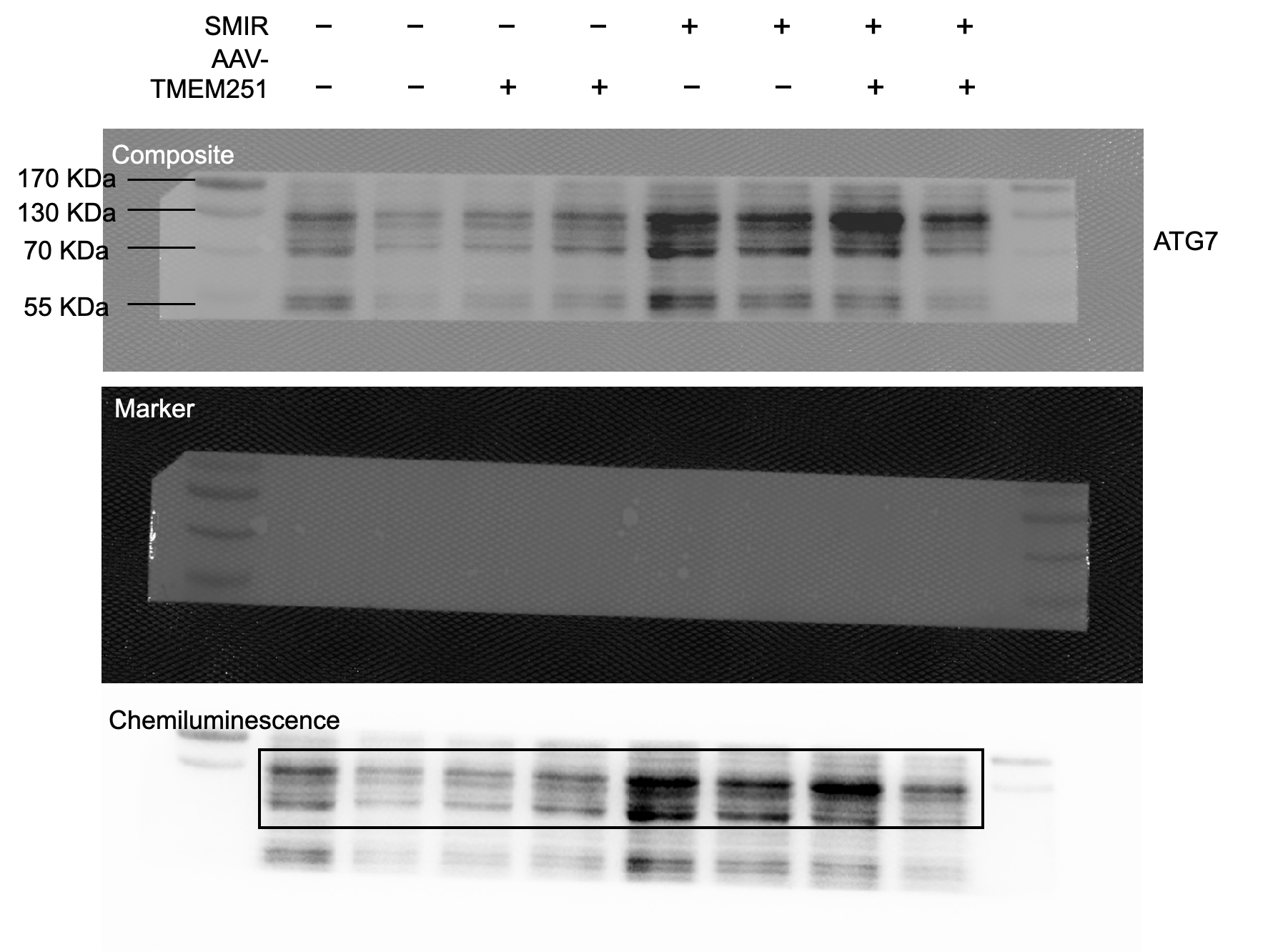

Supplement: Supplementary file 7 — Source data Fig. 5 [file 44319_2025_646_MOESM7_ESM.zip › Figure 5/5G/5G-ATG7.png]

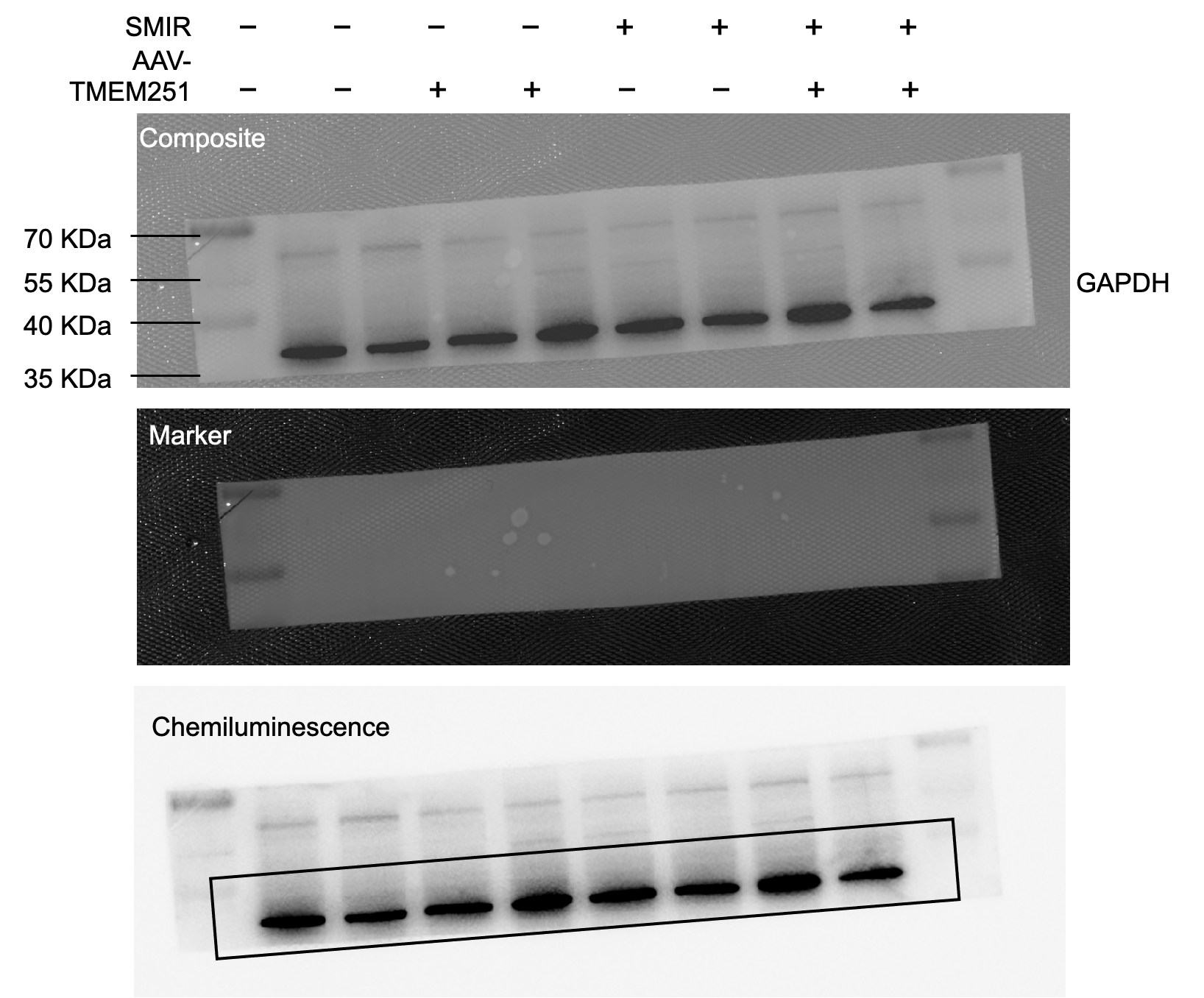

Supplement: Supplementary file 7 — Source data Fig. 5 [file 44319_2025_646_MOESM7_ESM.zip › Figure 5/5G/5G-GAPDH.png]

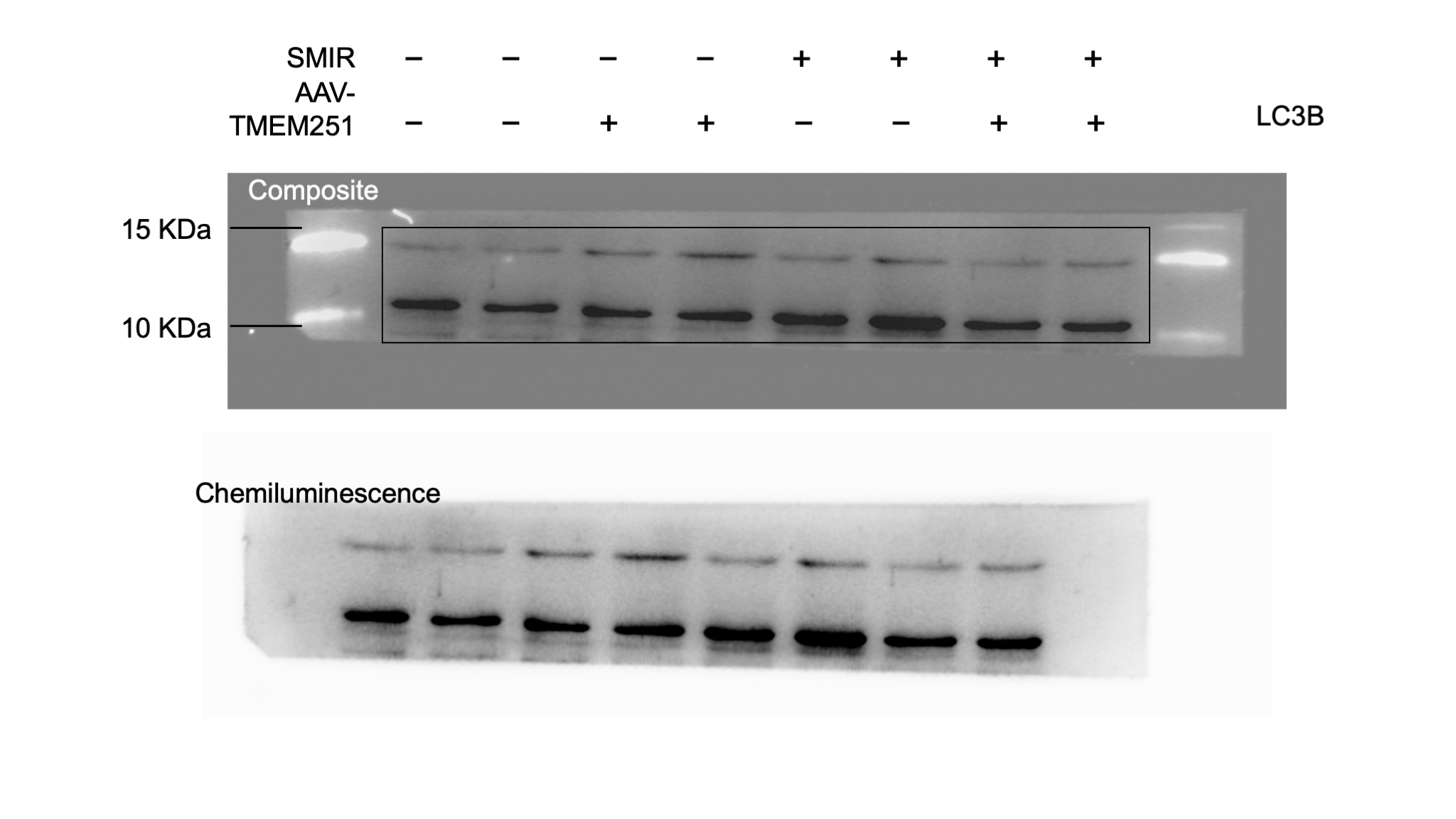

Supplement: Supplementary file 7 — Source data Fig. 5 [file 44319_2025_646_MOESM7_ESM.zip › Figure 5/5G/5G-LC3B.tiff]

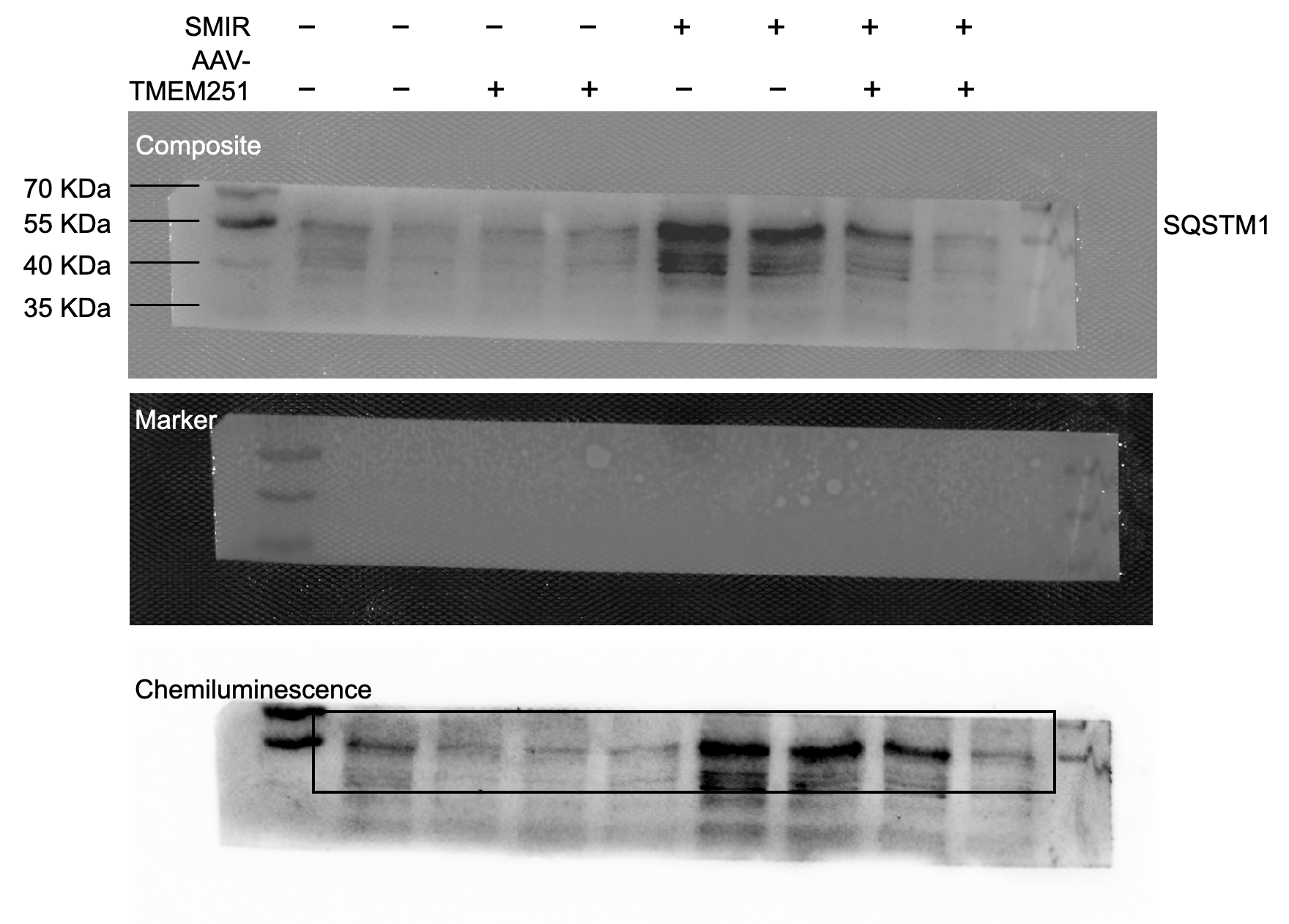

Supplement: Supplementary file 7 — Source data Fig. 5 [file 44319_2025_646_MOESM7_ESM.zip › Figure 5/5G/5G-SQSTM1.png]

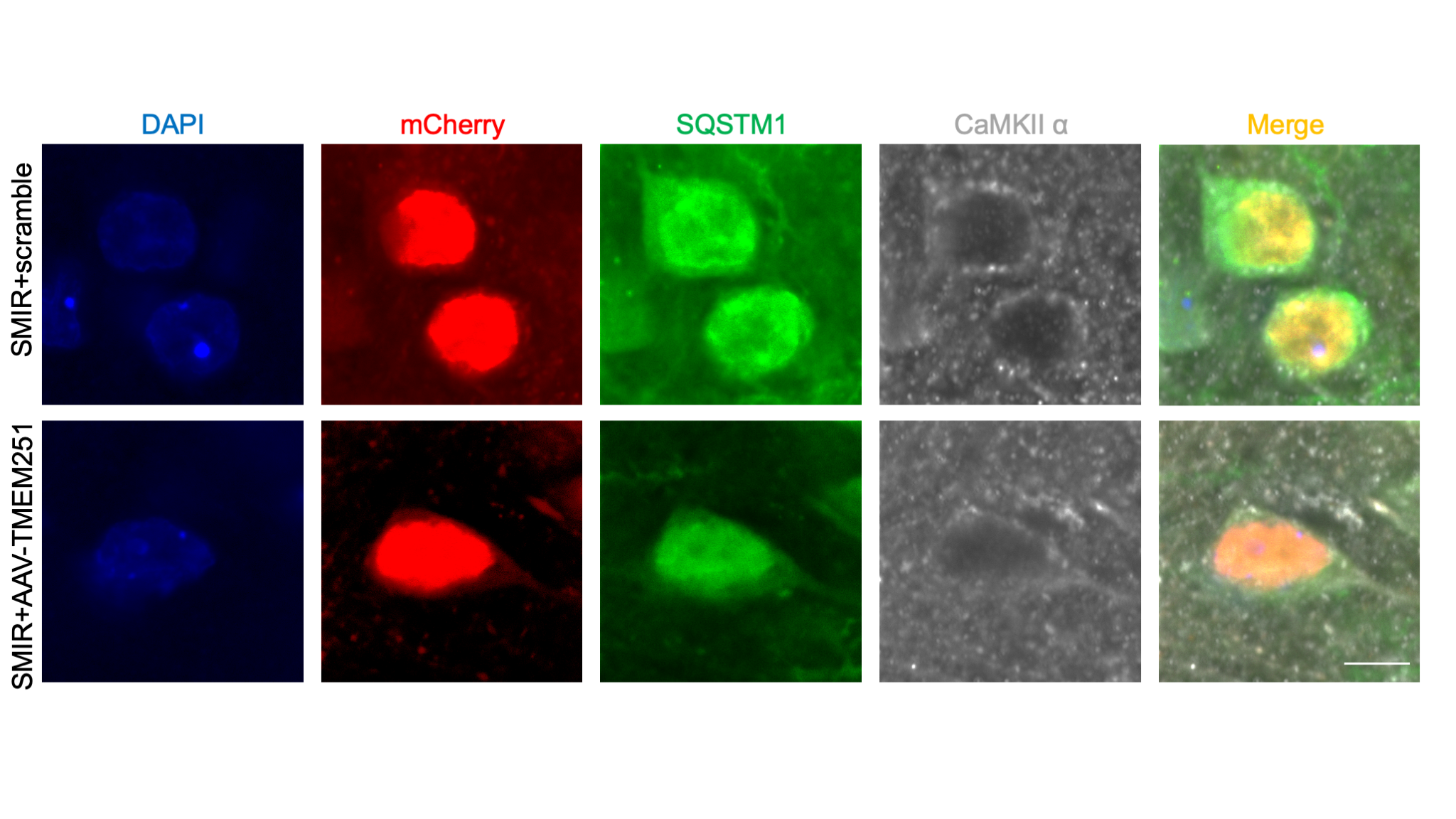

Supplement: Supplementary file 7 — Source data Fig. 5 [file 44319_2025_646_MOESM7_ESM.zip › Figure 5/5H/5H.tiff]

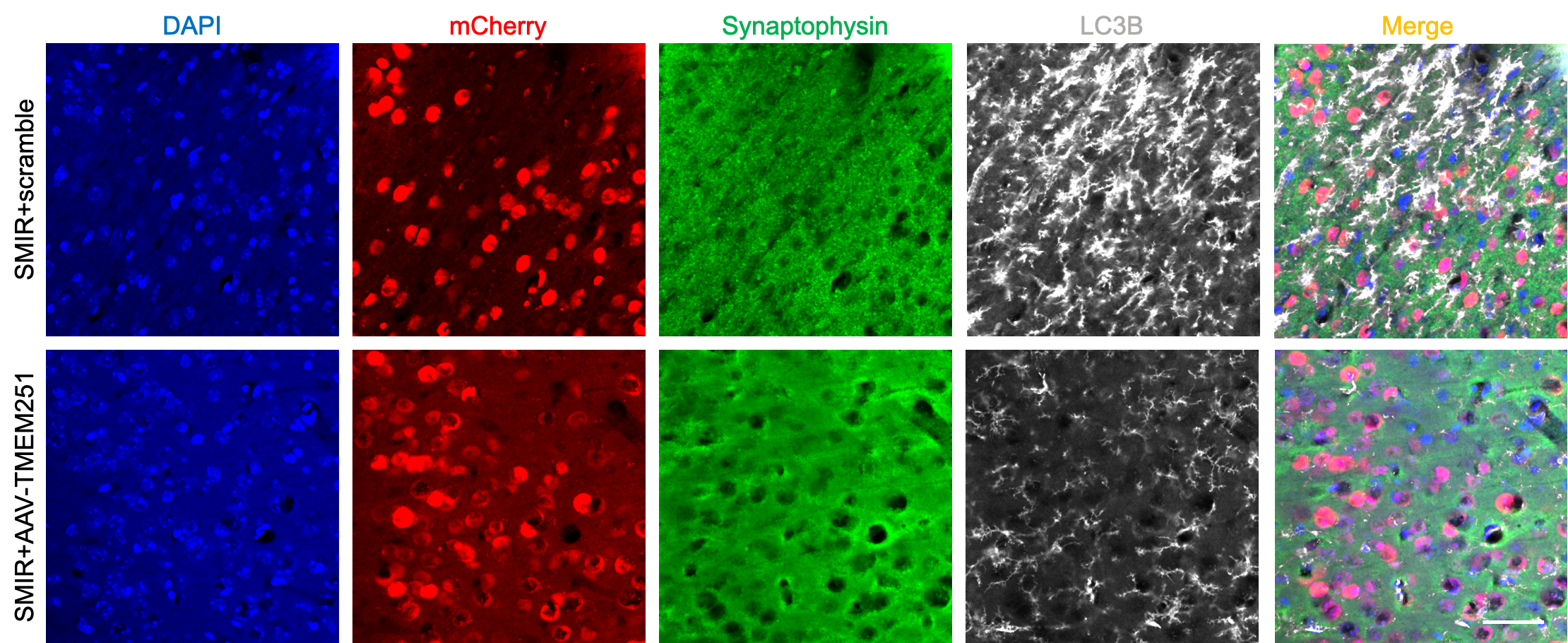

Supplement: Supplementary file 7 — Source data Fig. 5 [file 44319_2025_646_MOESM7_ESM.zip › Figure 5/5I/5I.png]

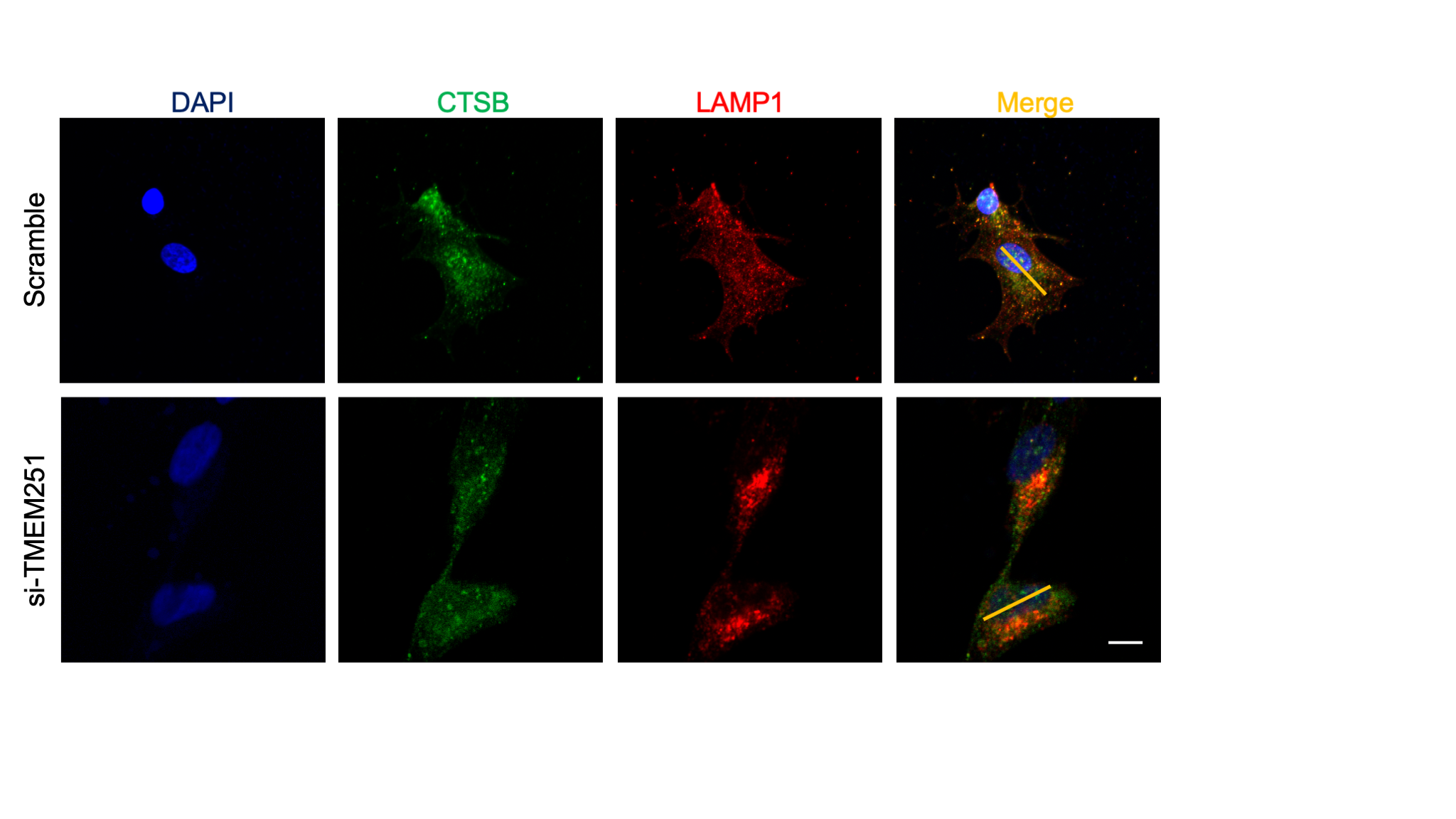

Supplement: Supplementary file 8 — Source data Fig. 6 [file 44319_2025_646_MOESM8_ESM.zip › Figure 6/6A/6A.tiff]

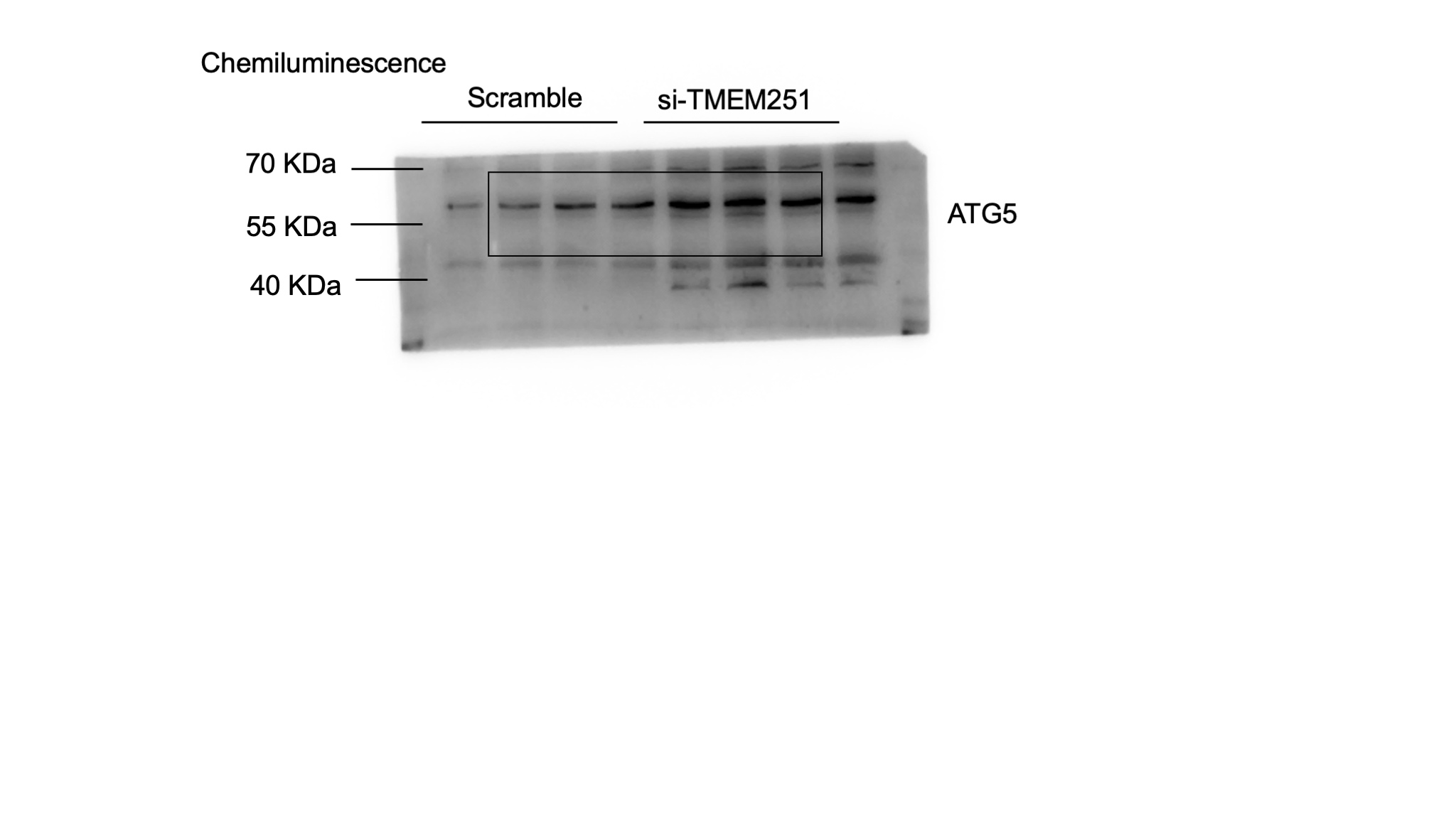

Supplement: Supplementary file 8 — Source data Fig. 6 [file 44319_2025_646_MOESM8_ESM.zip › Figure 6/6B/6B-ATG5.tiff]

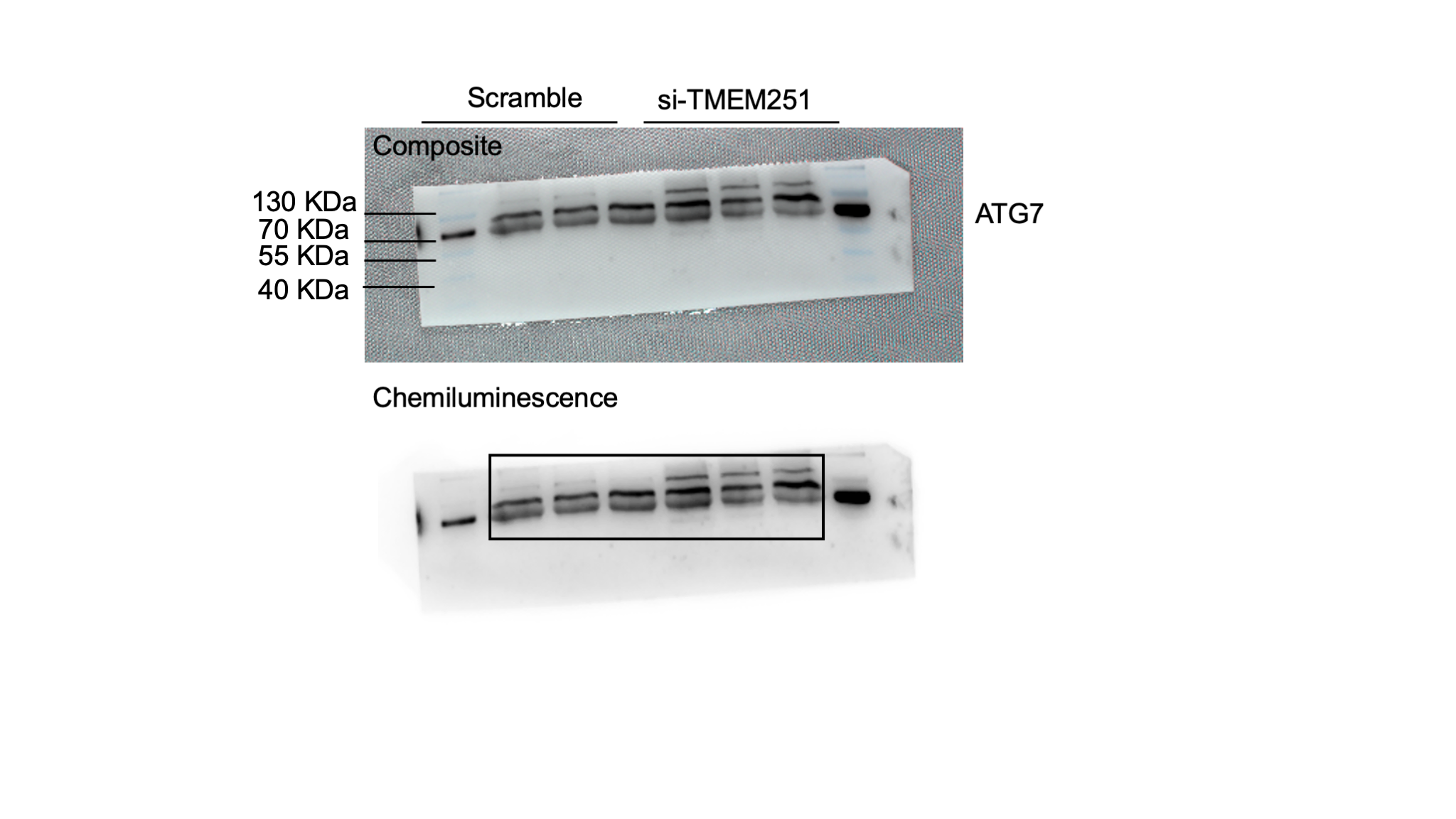

Supplement: Supplementary file 8 — Source data Fig. 6 [file 44319_2025_646_MOESM8_ESM.zip › Figure 6/6B/6B-ATG7.tiff]

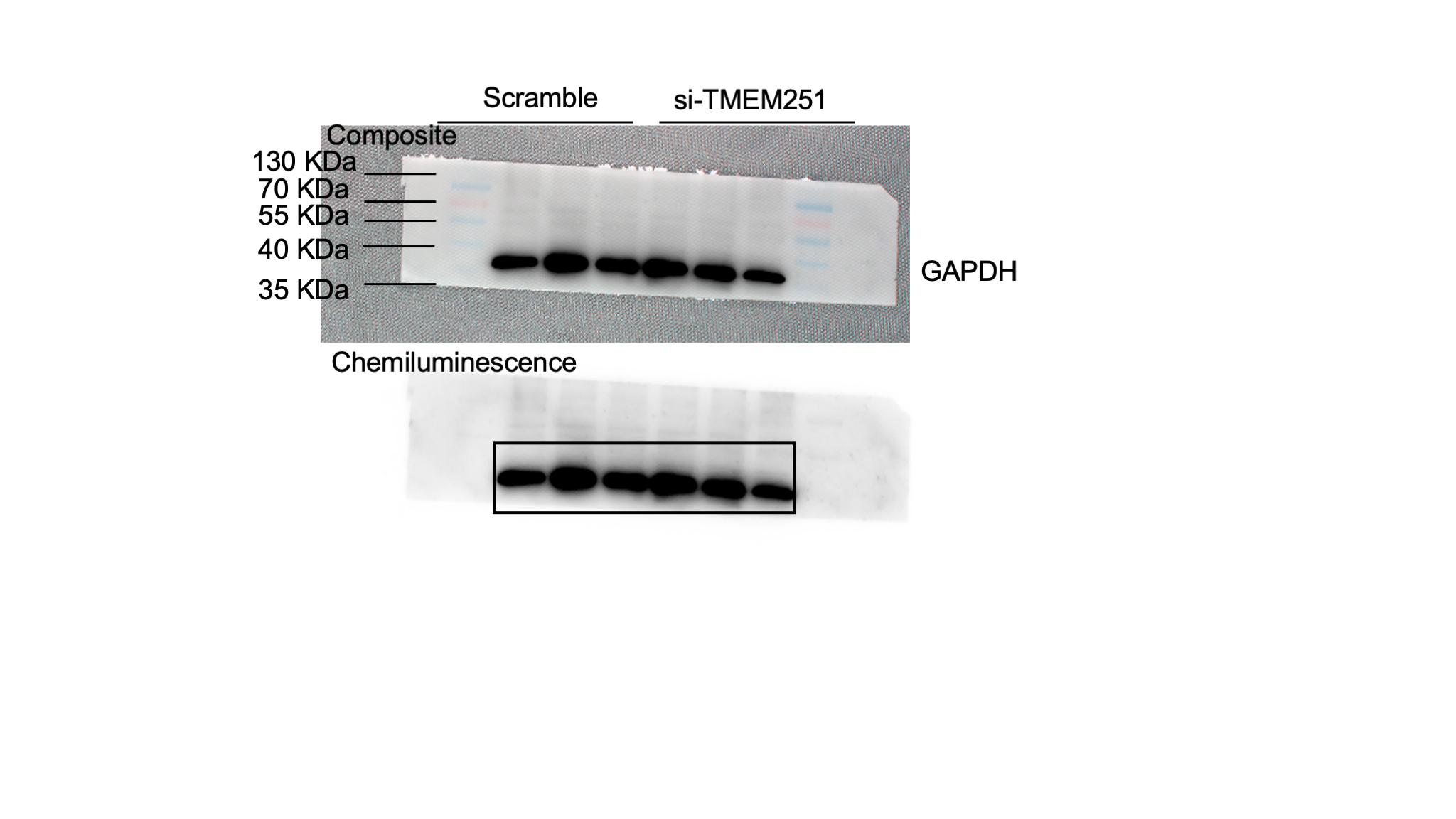

Supplement: Supplementary file 8 — Source data Fig. 6 [file 44319_2025_646_MOESM8_ESM.zip › Figure 6/6B/6B-GAPDH.tiff]

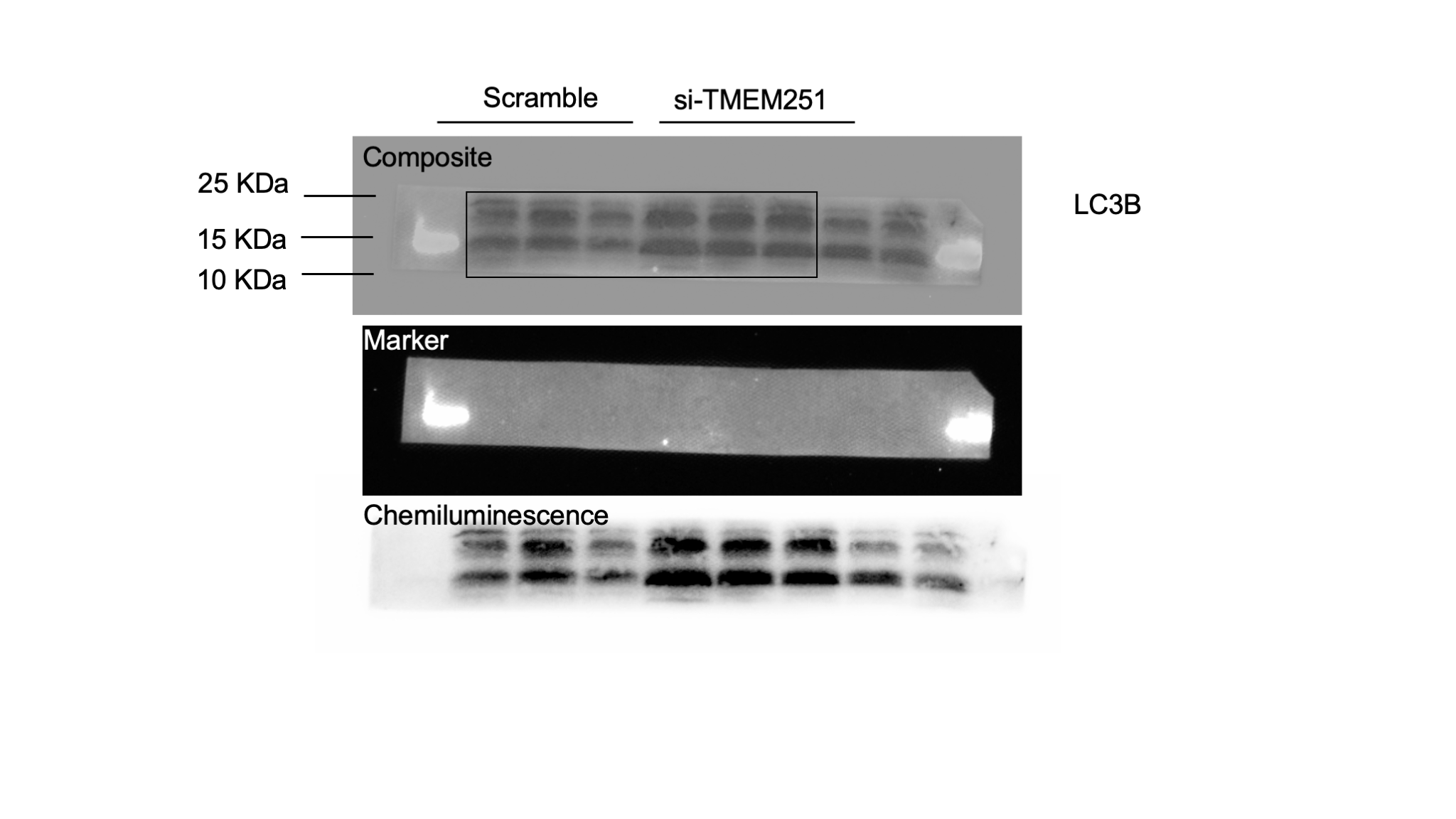

Supplement: Supplementary file 8 — Source data Fig. 6 [file 44319_2025_646_MOESM8_ESM.zip › Figure 6/6B/6B-LC3B.tiff]

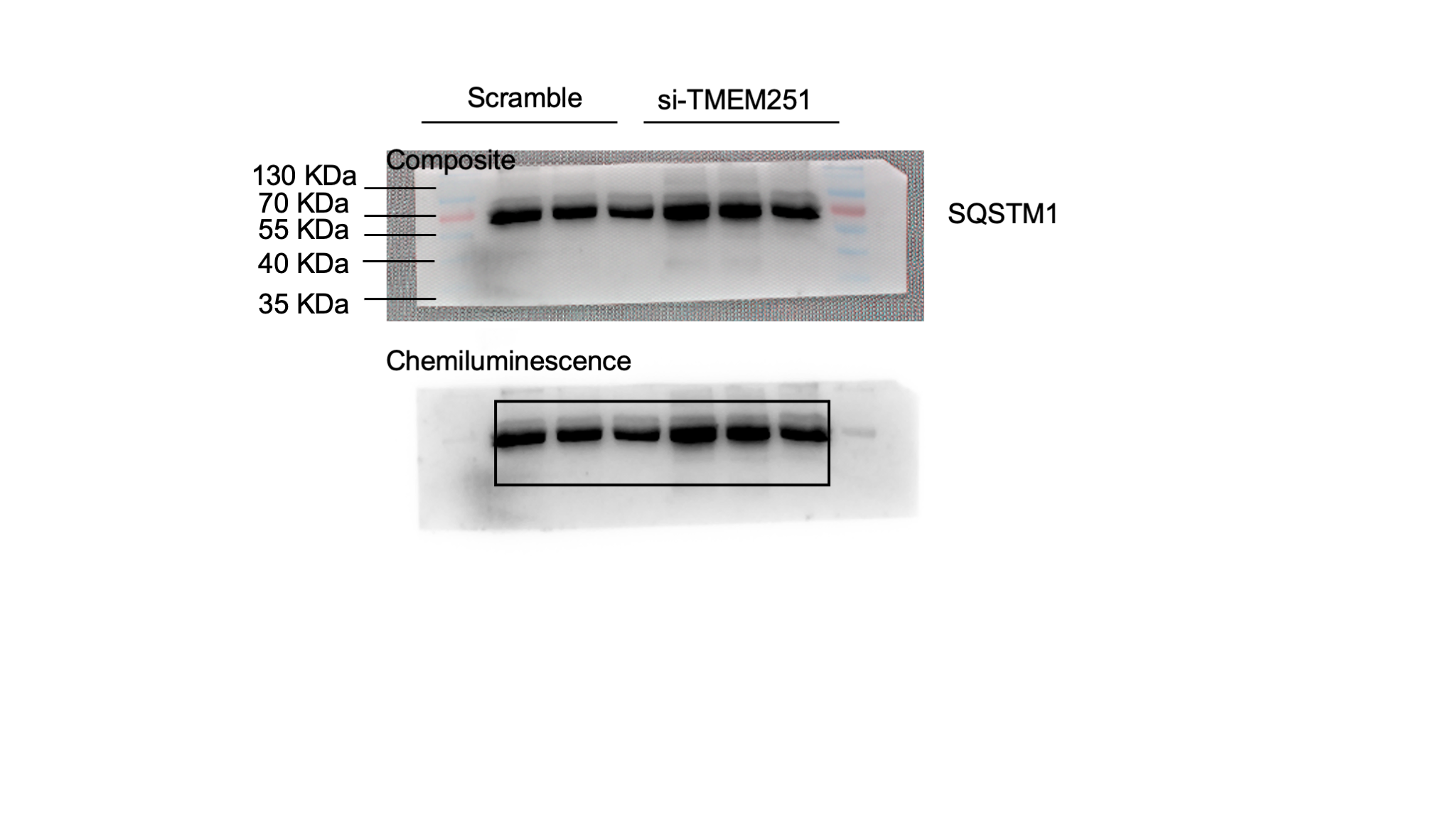

Supplement: Supplementary file 8 — Source data Fig. 6 [file 44319_2025_646_MOESM8_ESM.zip › Figure 6/6B/6B-SQSTM1.tiff]

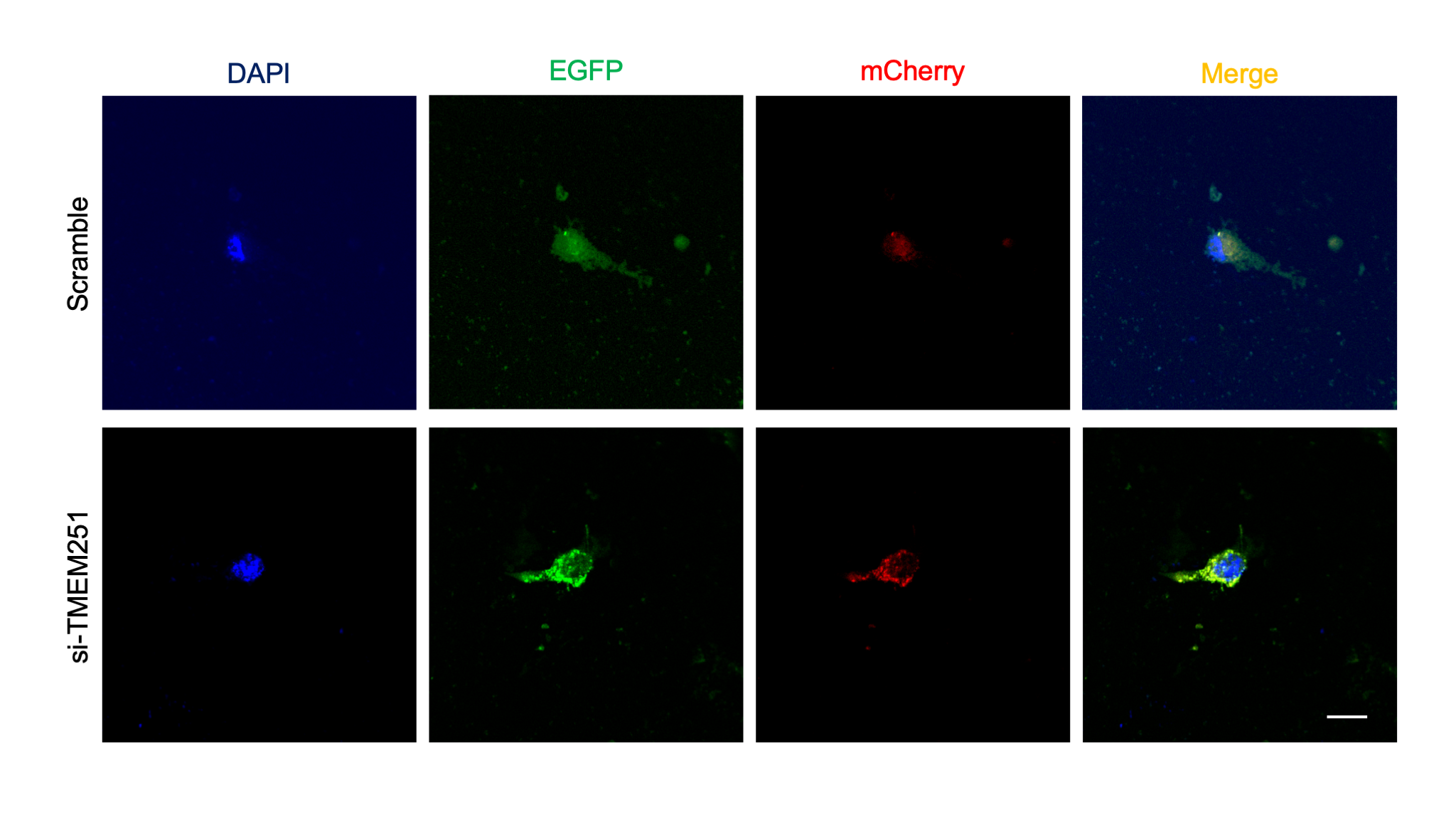

Supplement: Supplementary file 8 — Source data Fig. 6 [file 44319_2025_646_MOESM8_ESM.zip › Figure 6/6D/6D.tiff]

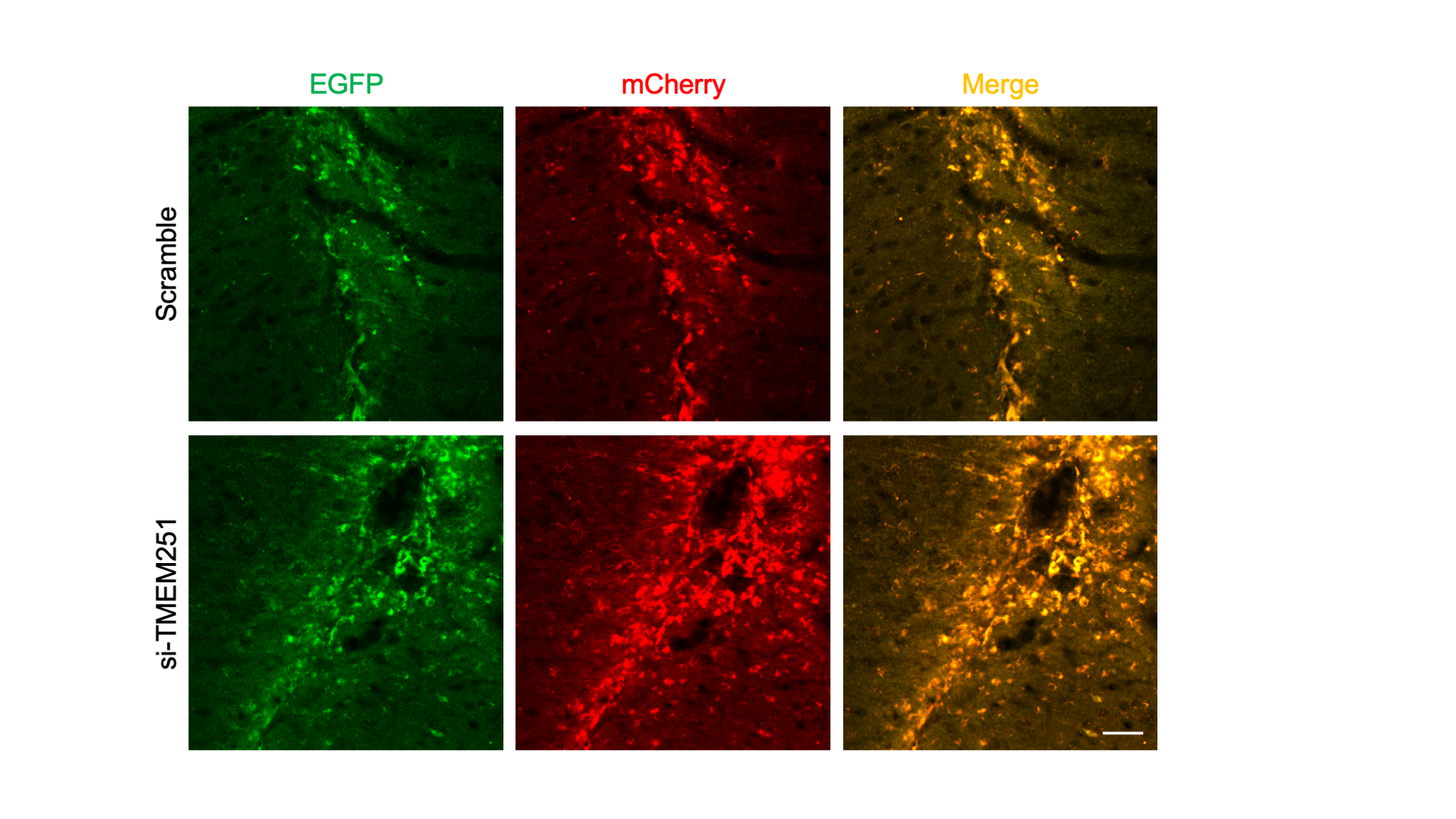

Supplement: Supplementary file 8 — Source data Fig. 6 [file 44319_2025_646_MOESM8_ESM.zip › Figure 6/6G/6G.tiff]

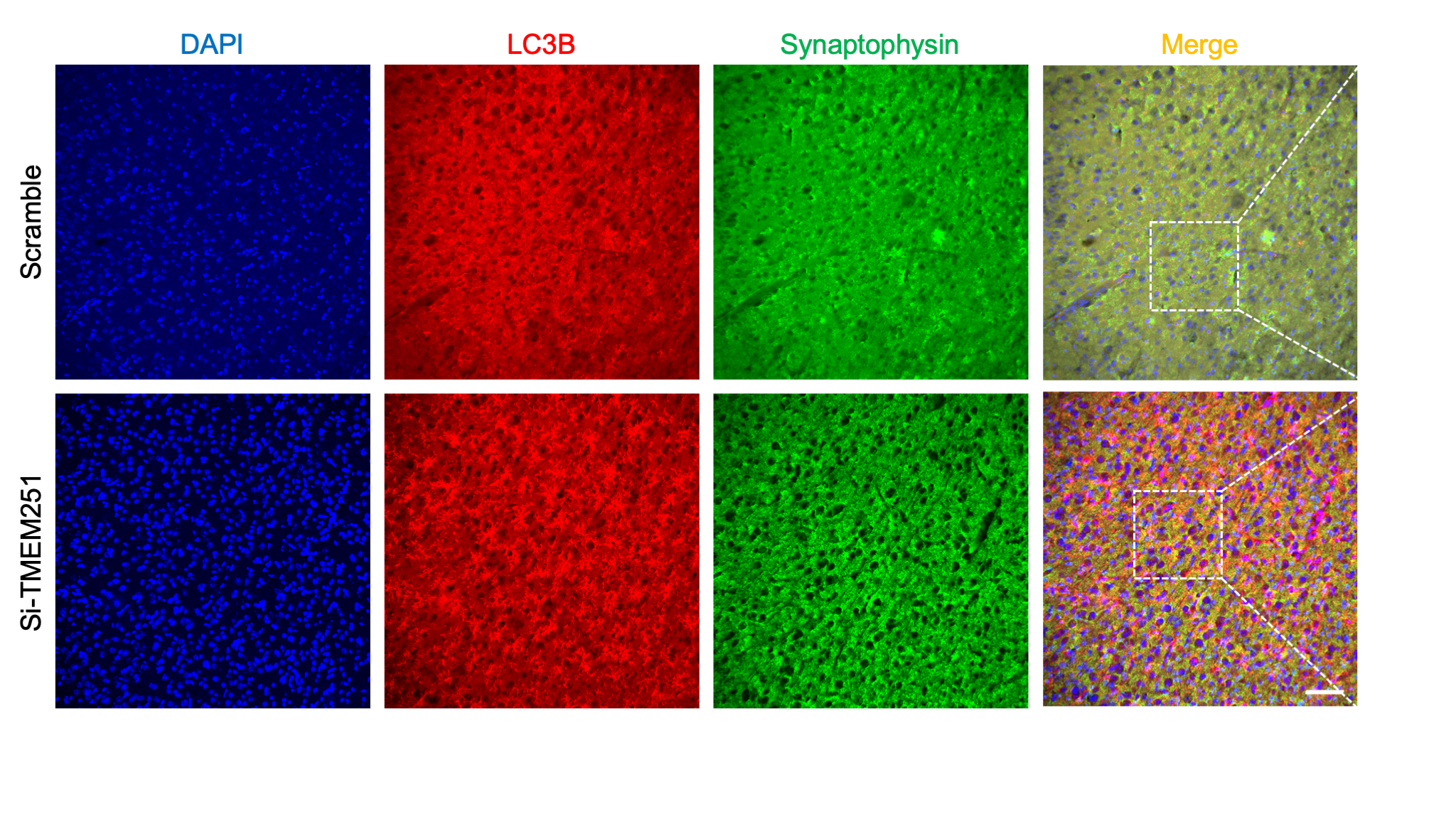

Supplement: Supplementary file 8 — Source data Fig. 6 [file 44319_2025_646_MOESM8_ESM.zip › Figure 6/6J/6J.tiff]

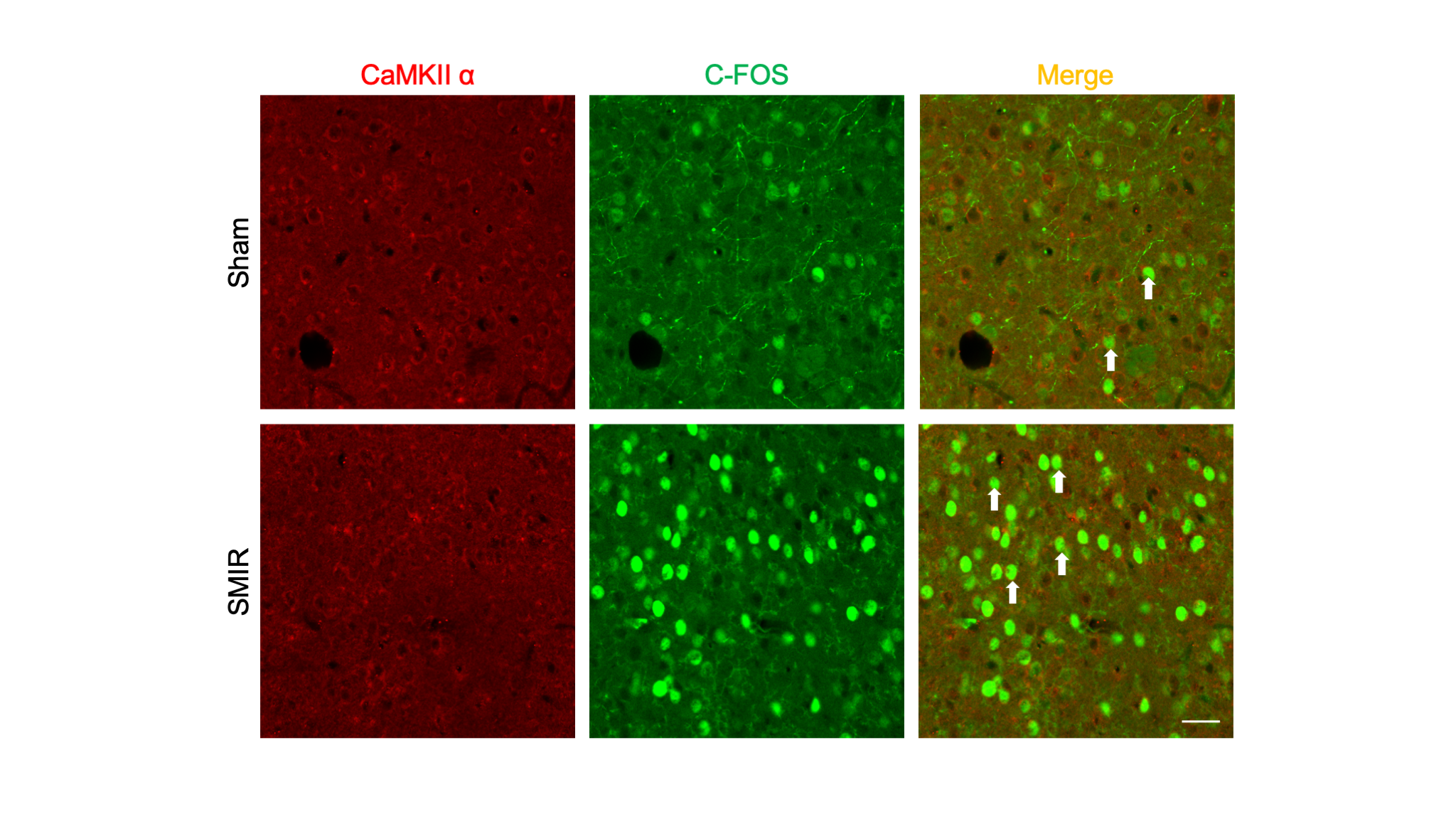

Supplement: Supplementary file 9 — Source data Fig. 7 [file 44319_2025_646_MOESM9_ESM.zip › Figure 7/7A/7A.tiff]

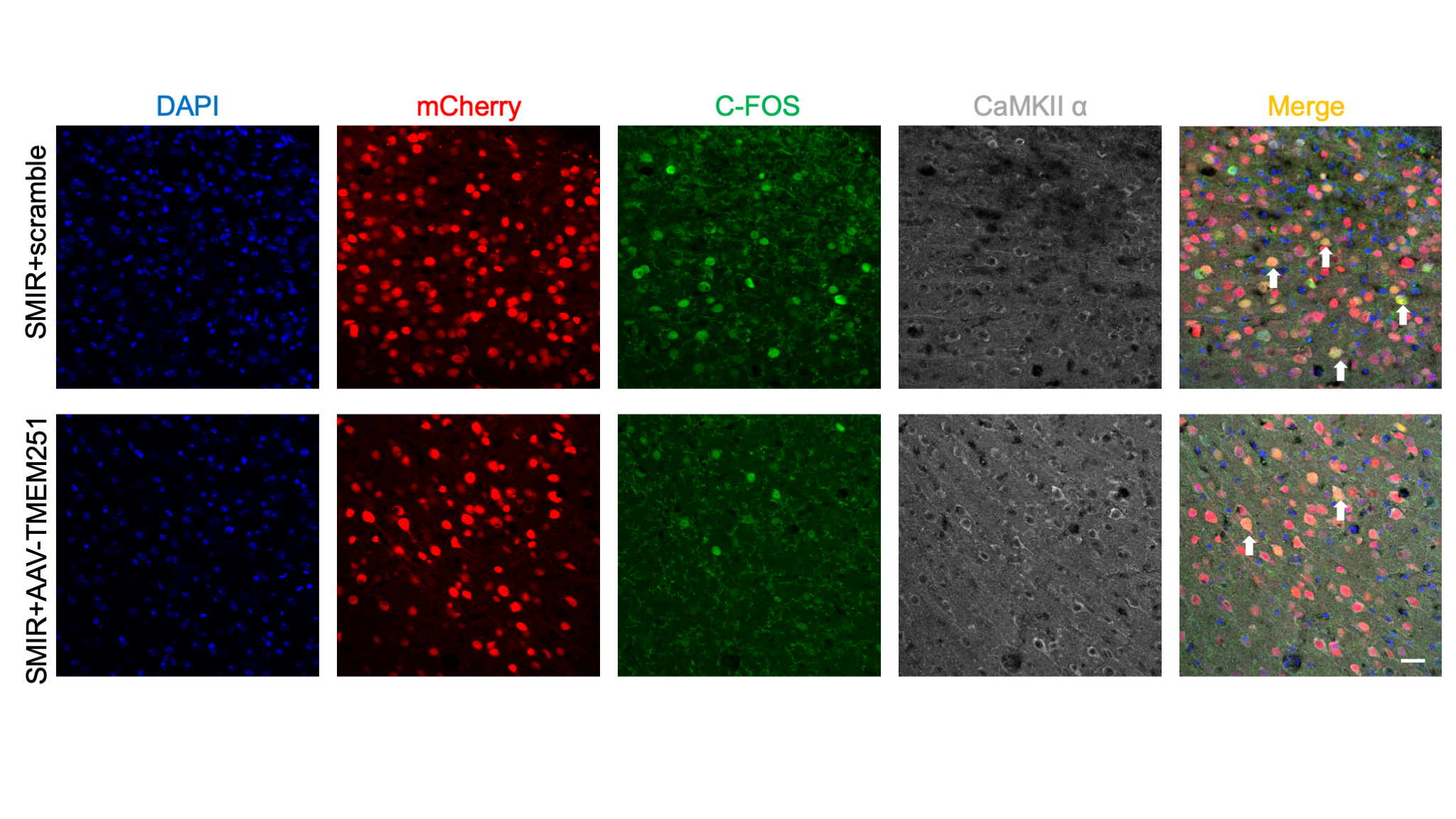

Supplement: Supplementary file 9 — Source data Fig. 7 [file 44319_2025_646_MOESM9_ESM.zip › Figure 7/7B/7B.tiff]

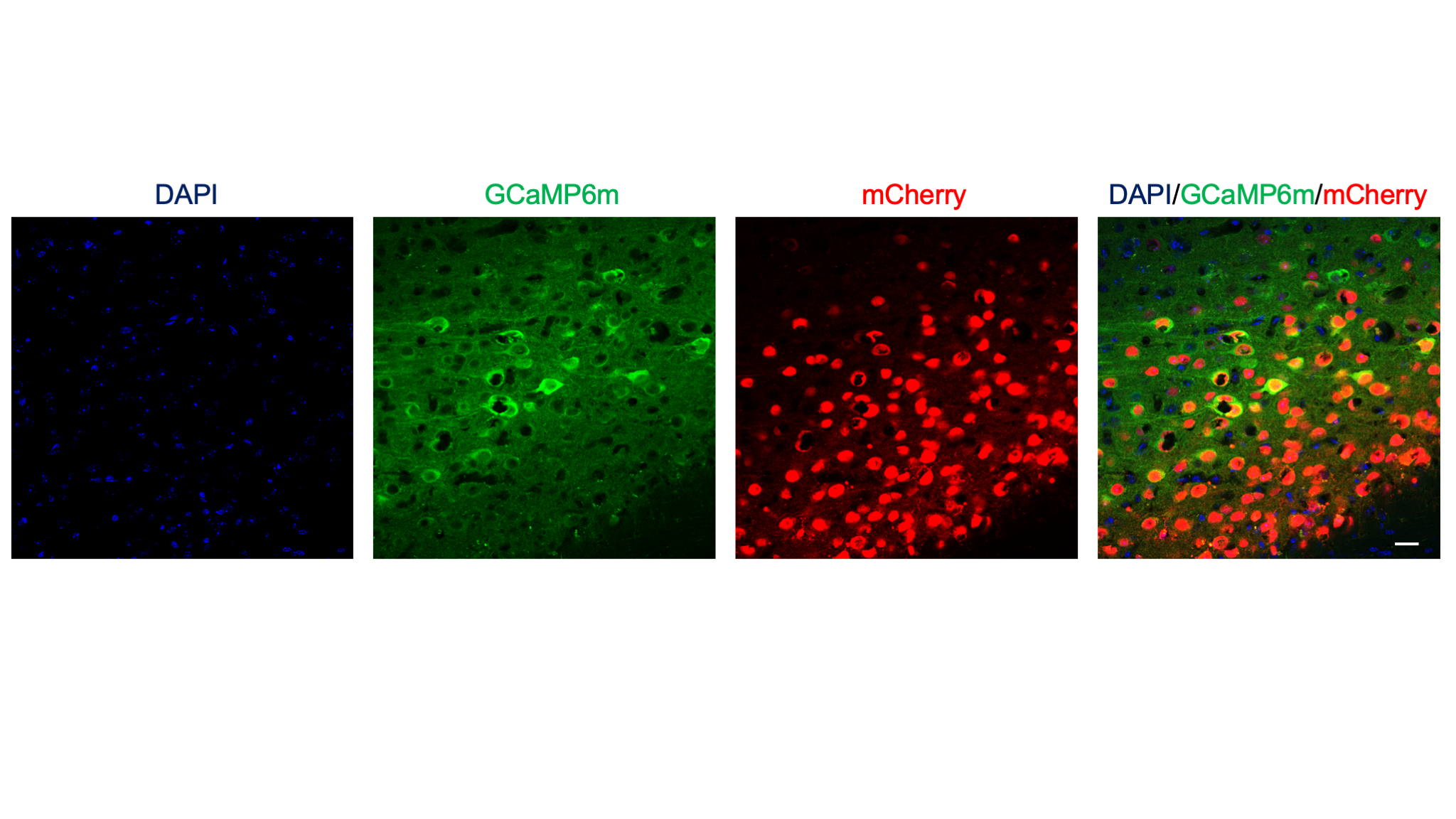

Supplement: Supplementary file 9 — Source data Fig. 7 [file 44319_2025_646_MOESM9_ESM.zip › Figure 7/7D/7D.tiff]

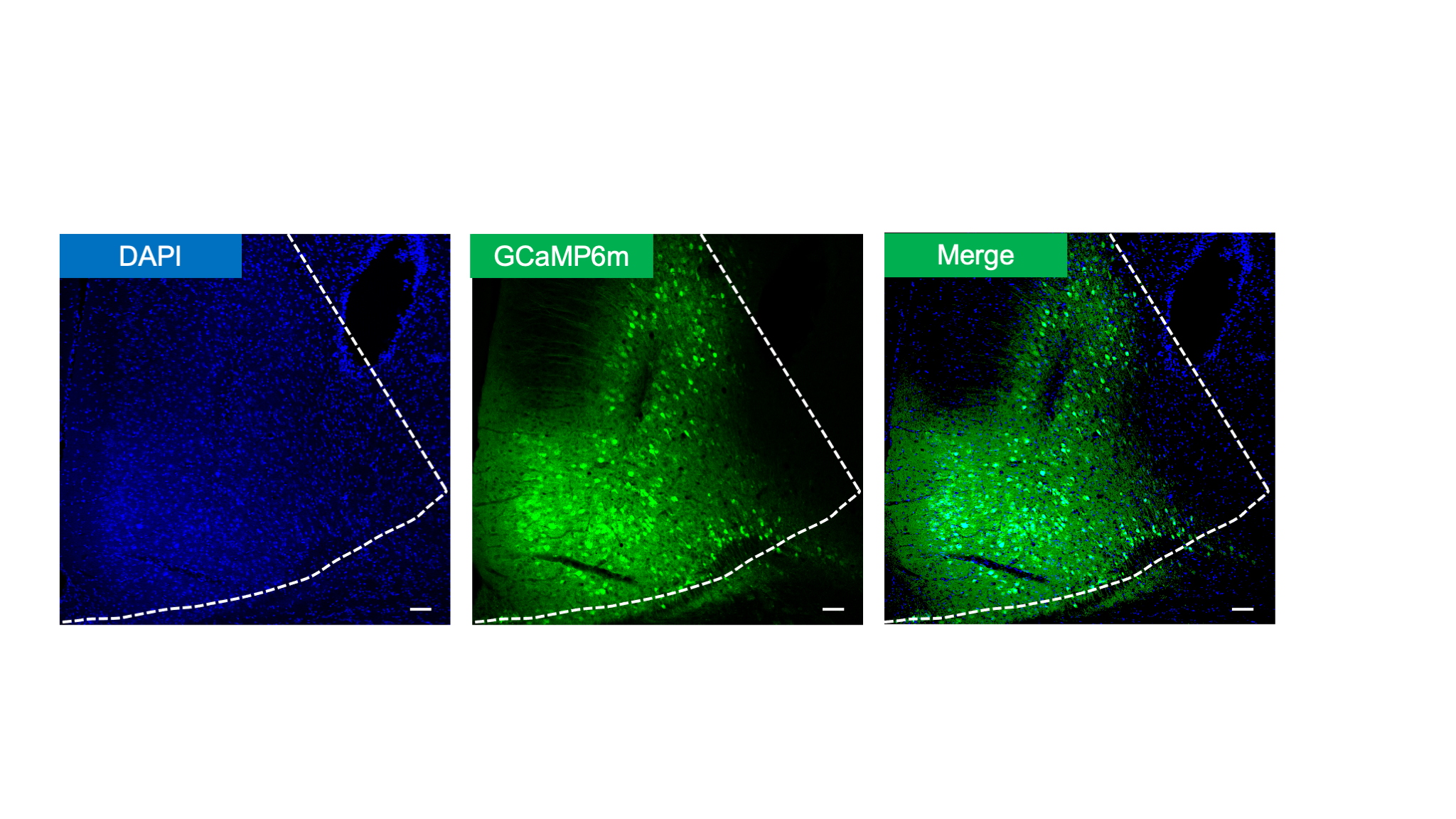

Supplement: Supplementary file 9 — Source data Fig. 7 [file 44319_2025_646_MOESM9_ESM.zip › Figure 7/7L/7L.tiff]

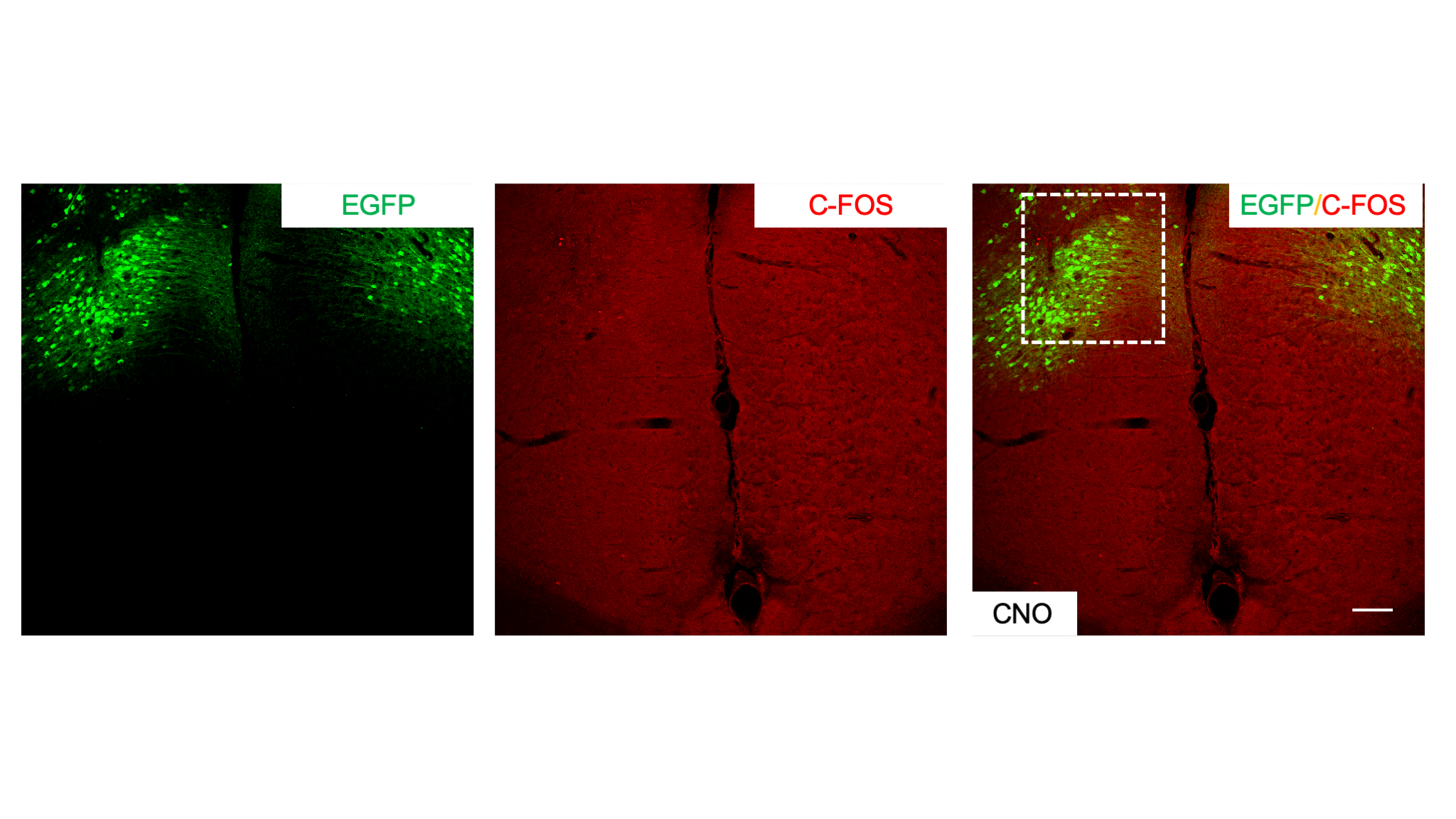

Supplement: Supplementary file 10 — Source data Fig. 8 [file 44319_2025_646_MOESM10_ESM.zip › Figure 8/8B/8B-CNO.tiff]

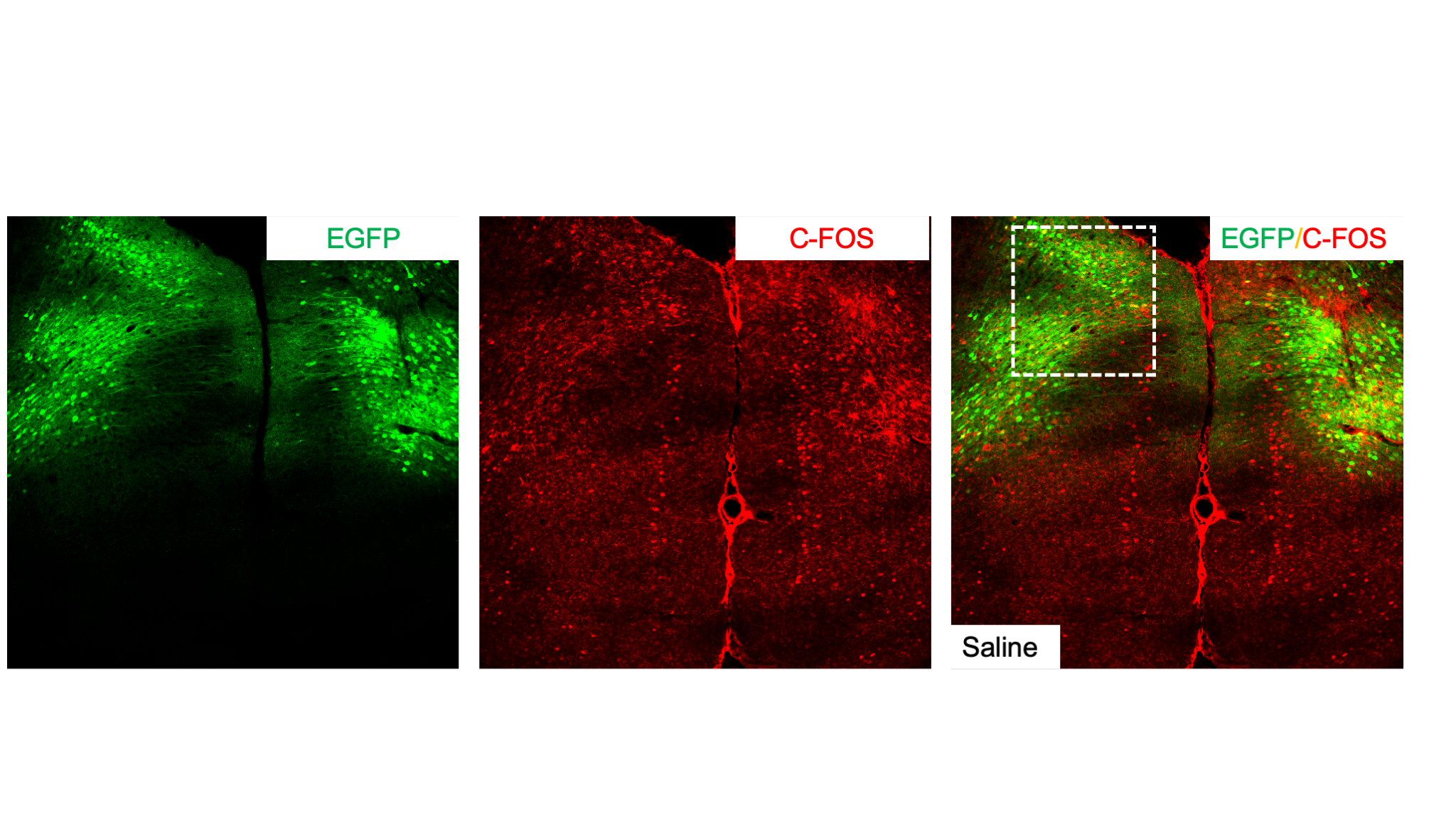

Supplement: Supplementary file 10 — Source data Fig. 8 [file 44319_2025_646_MOESM10_ESM.zip › Figure 8/8B/8B-Saline.tiff]

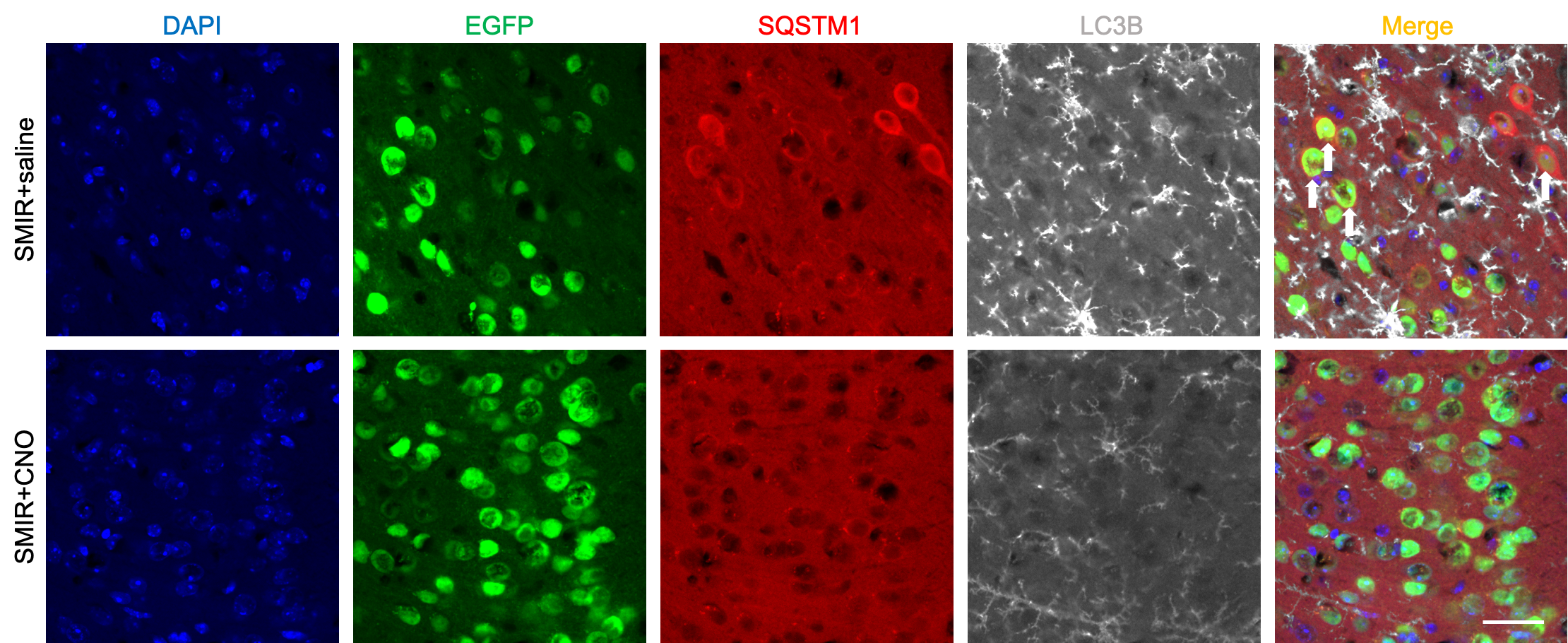

Supplement: Supplementary file 10 — Source data Fig. 8 [file 44319_2025_646_MOESM10_ESM.zip › Figure 8/8E/8E.png]

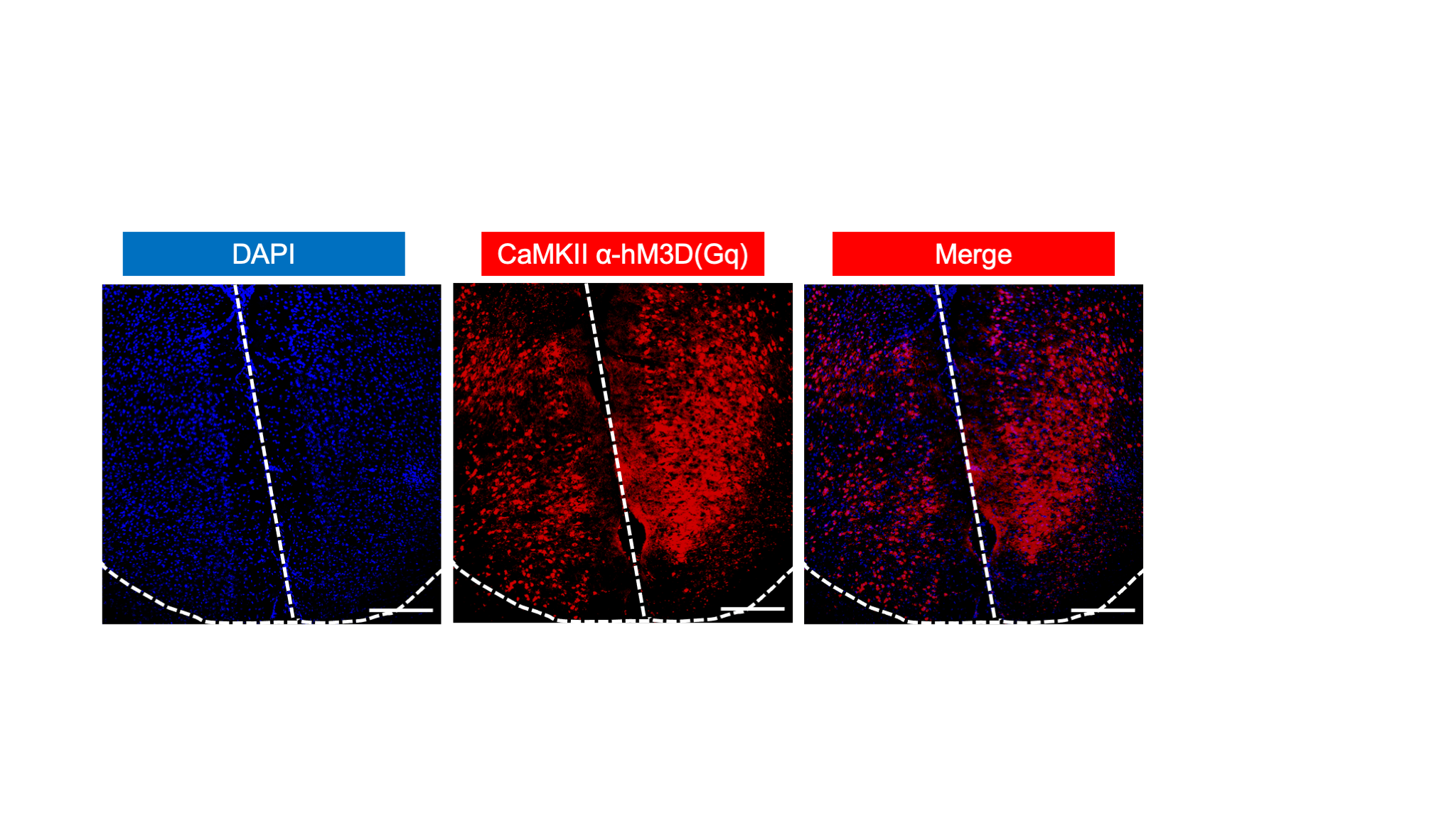

Supplement: Supplementary file 10 — Source data Fig. 8 [file 44319_2025_646_MOESM10_ESM.zip › Figure 8/8G/8G.tiff]

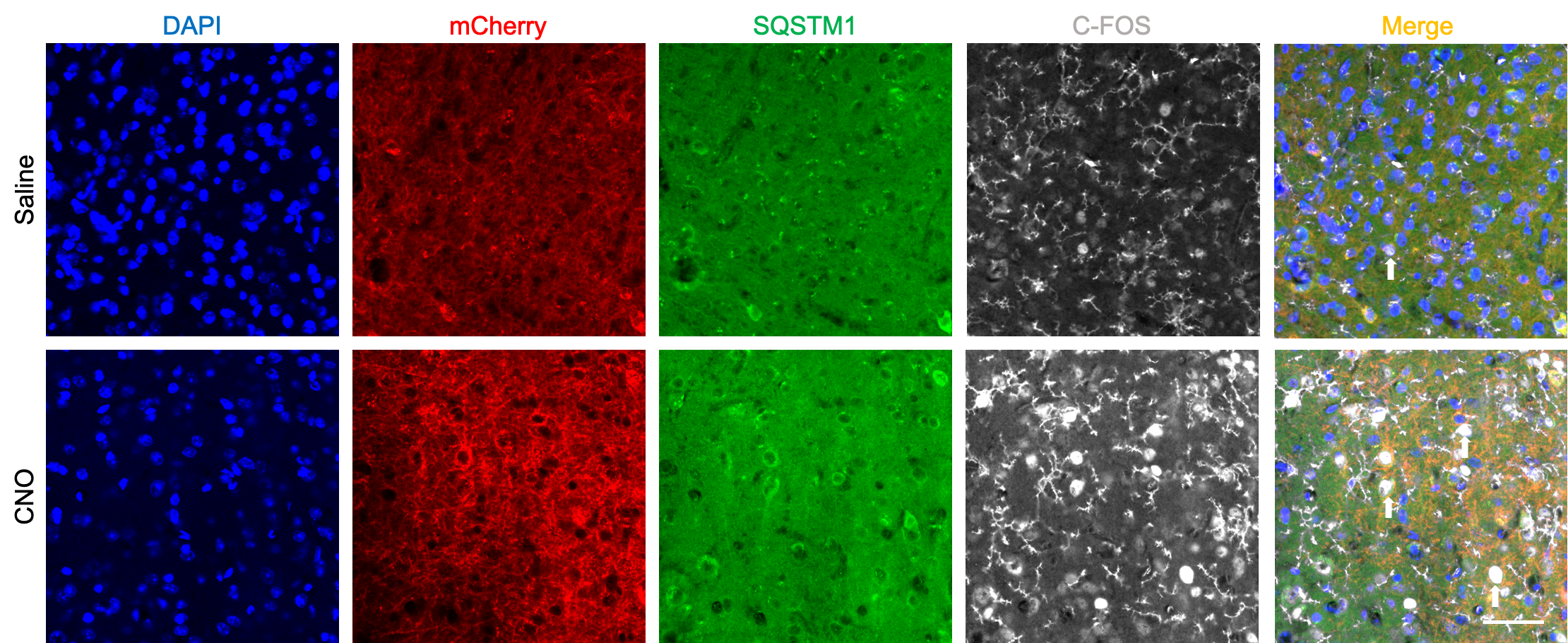

Supplement: Supplementary file 10 — Source data Fig. 8 [file 44319_2025_646_MOESM10_ESM.zip › Figure 8/8H/8H.png]

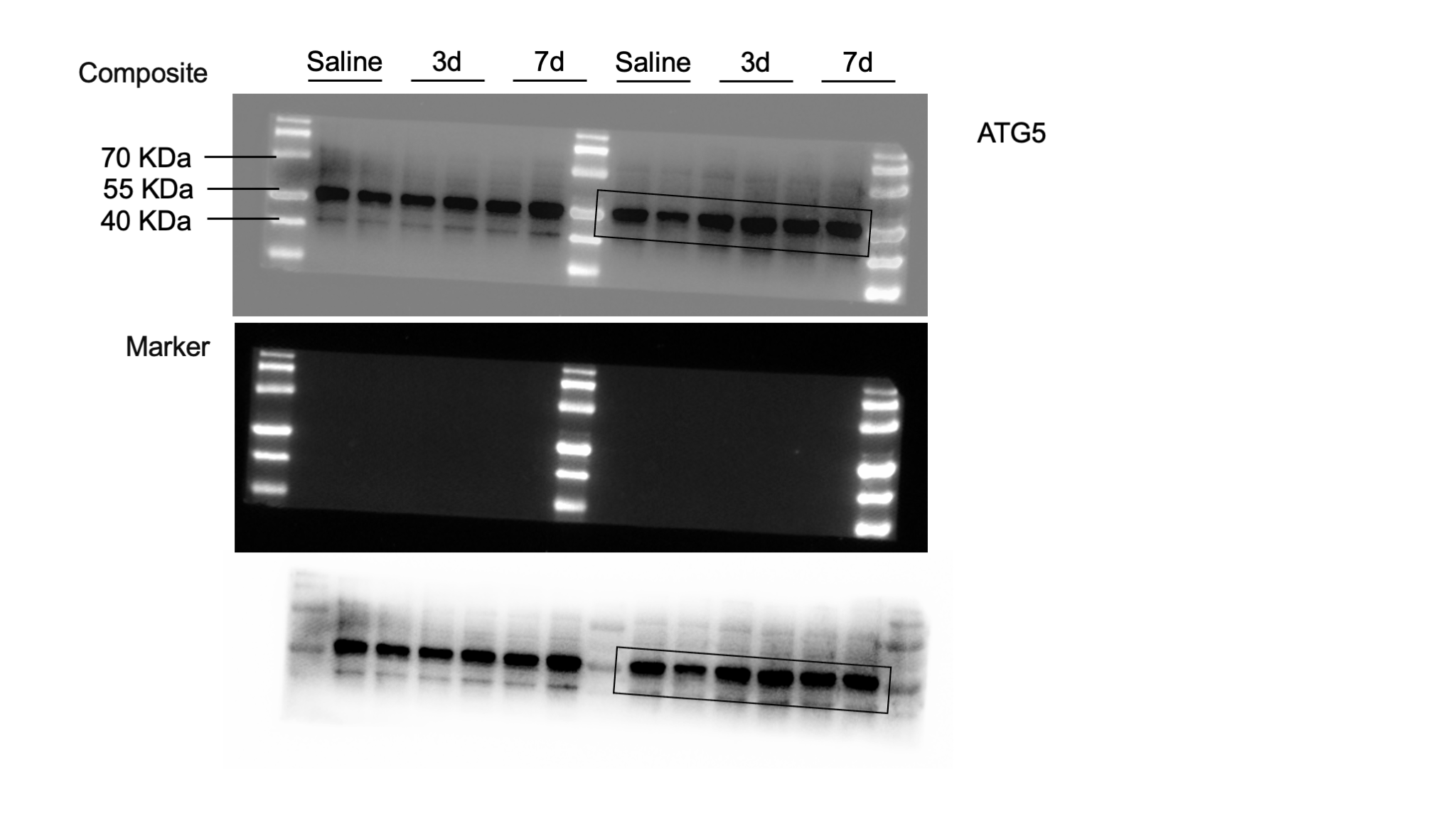

Supplement: Supplementary file 10 — Source data Fig. 8 [file 44319_2025_646_MOESM10_ESM.zip › Figure 8/8I/8I-ATG5.tiff]

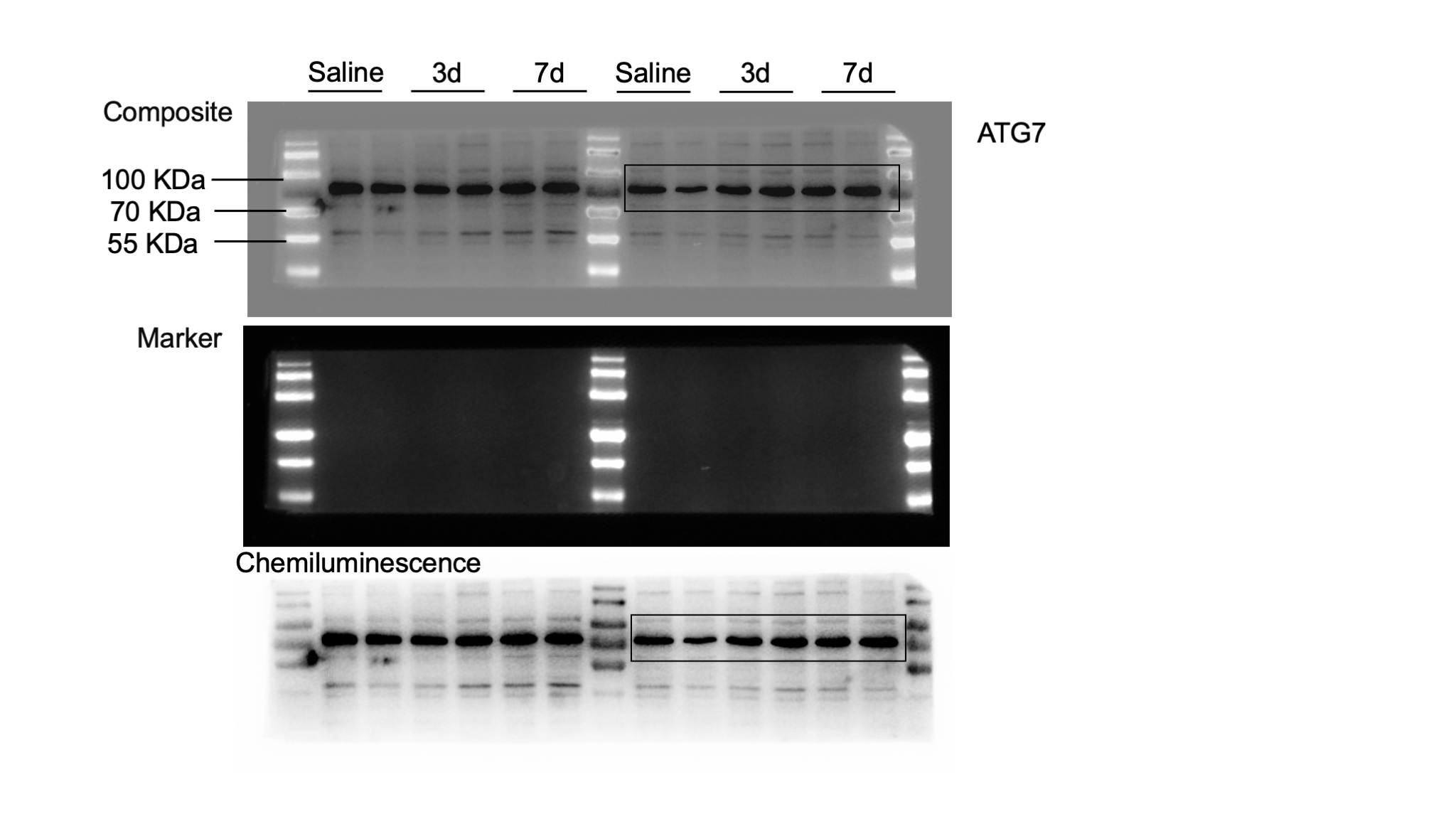

Supplement: Supplementary file 10 — Source data Fig. 8 [file 44319_2025_646_MOESM10_ESM.zip › Figure 8/8I/8I-ATG7.tiff]

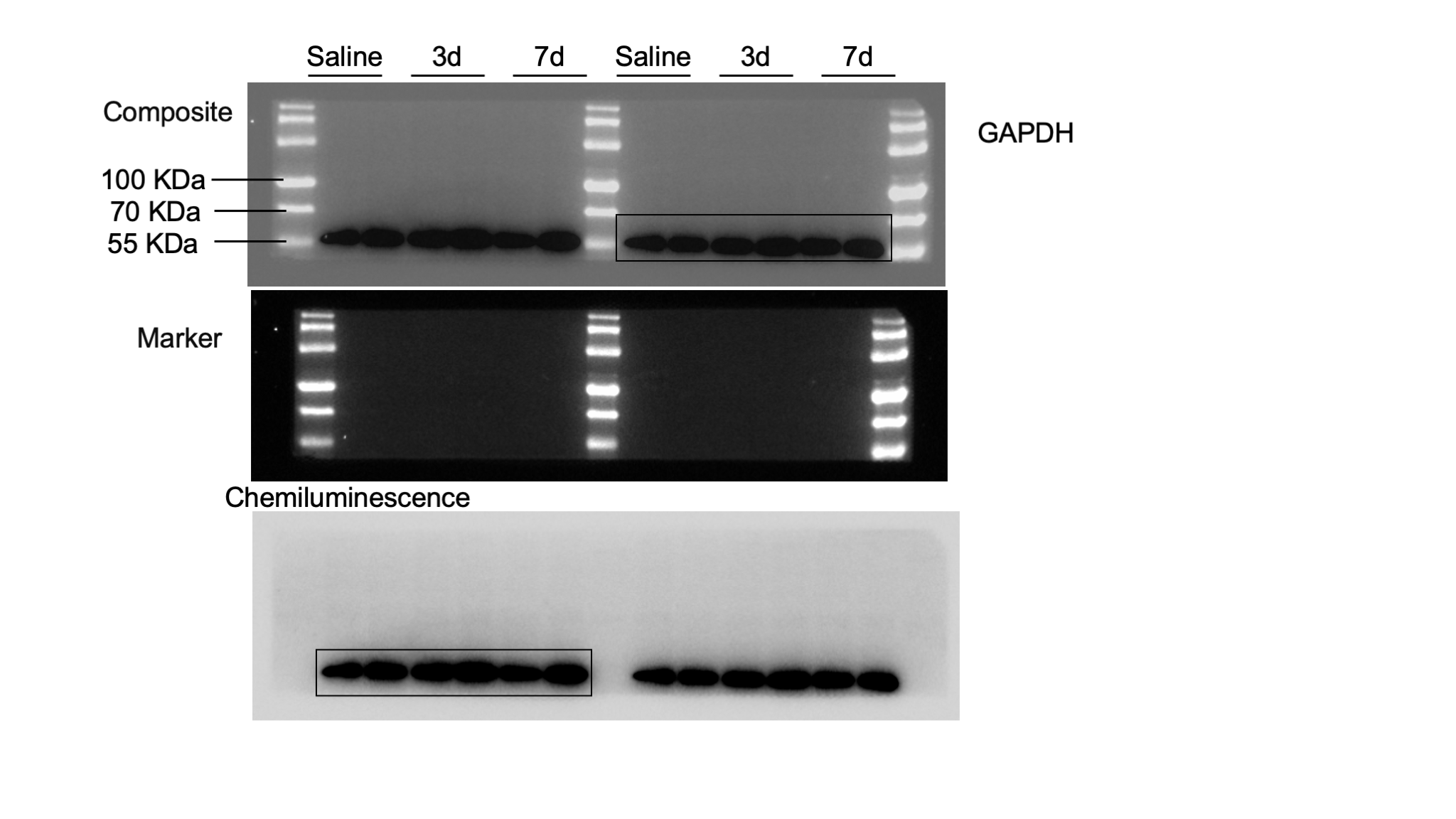

Supplement: Supplementary file 10 — Source data Fig. 8 [file 44319_2025_646_MOESM10_ESM.zip › Figure 8/8I/8I-GAPDH.tiff]

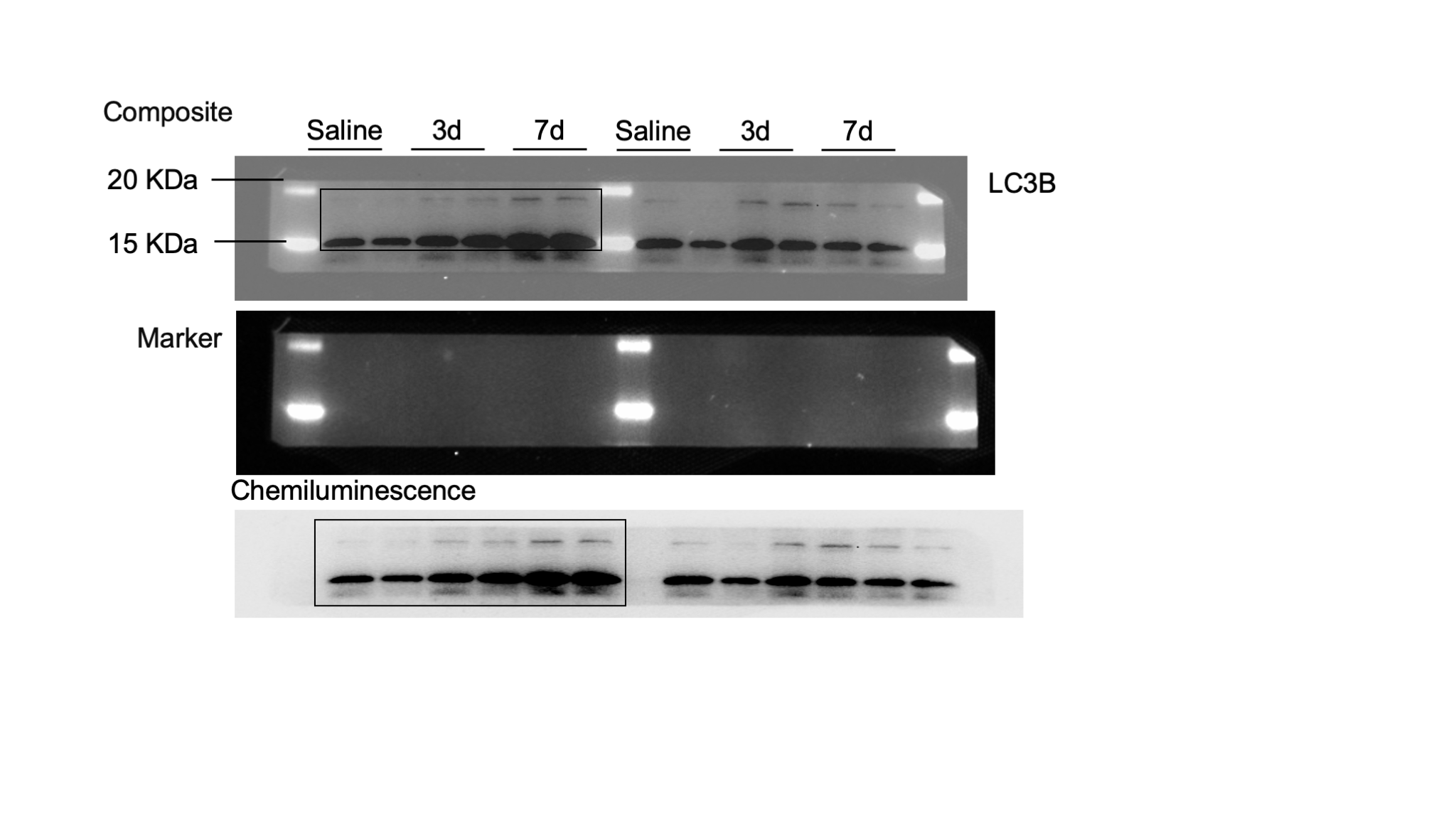

Supplement: Supplementary file 10 — Source data Fig. 8 [file 44319_2025_646_MOESM10_ESM.zip › Figure 8/8I/8I-LC3B.tiff]

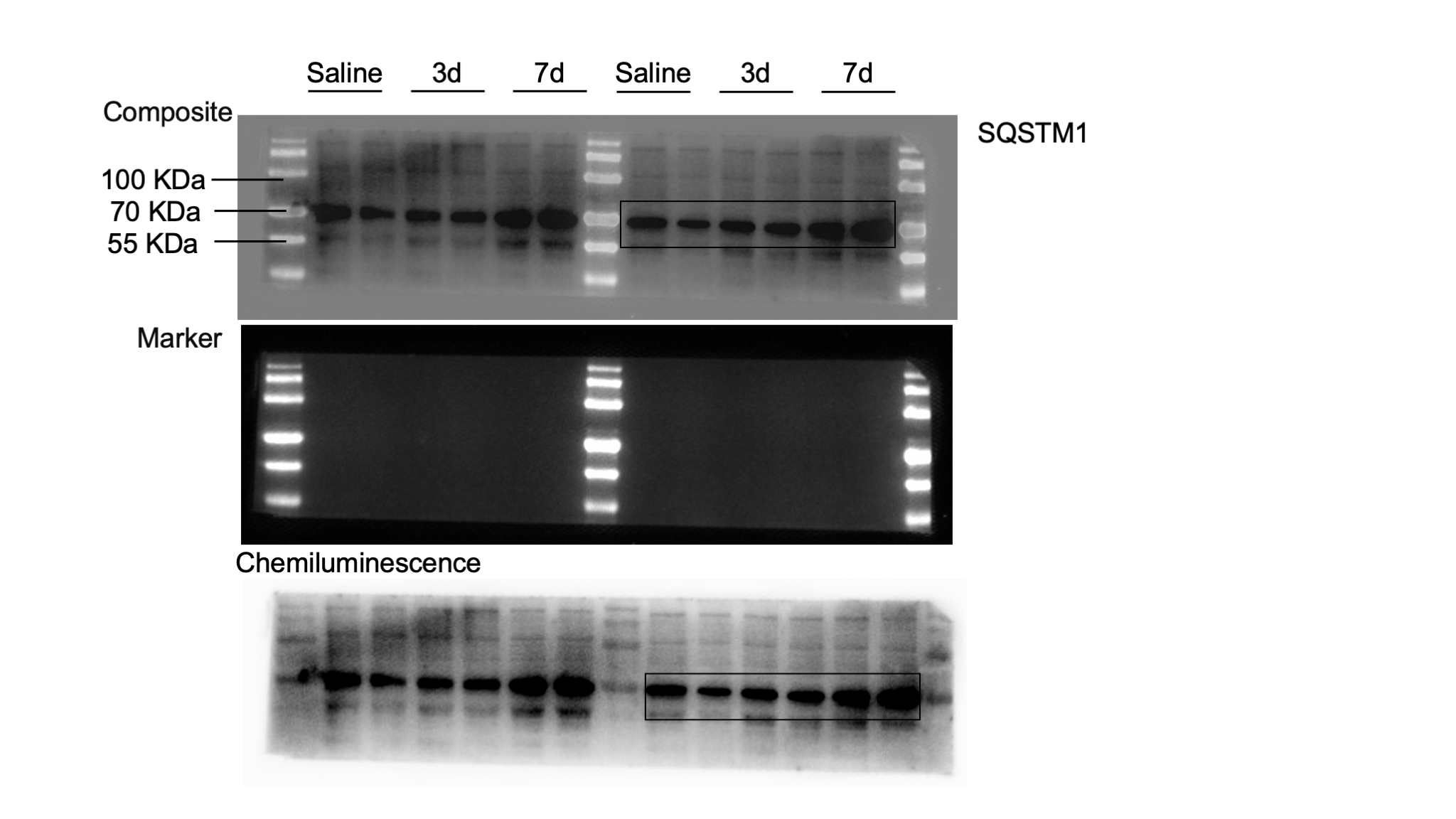

Supplement: Supplementary file 10 — Source data Fig. 8 [file 44319_2025_646_MOESM10_ESM.zip › Figure 8/8I/8I-SQSTM1.tiff]

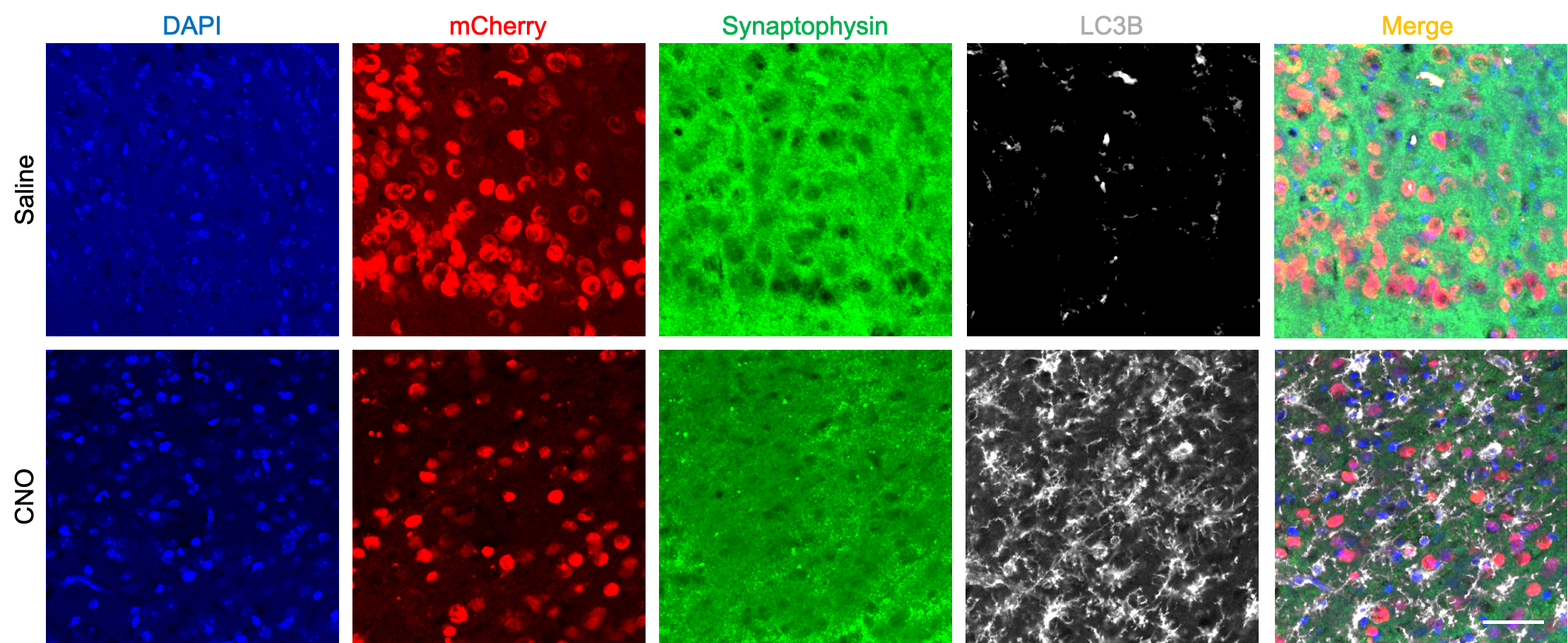

Supplement: Supplementary file 10 — Source data Fig. 8 [file 44319_2025_646_MOESM10_ESM.zip › Figure 8/8J/8J.png]

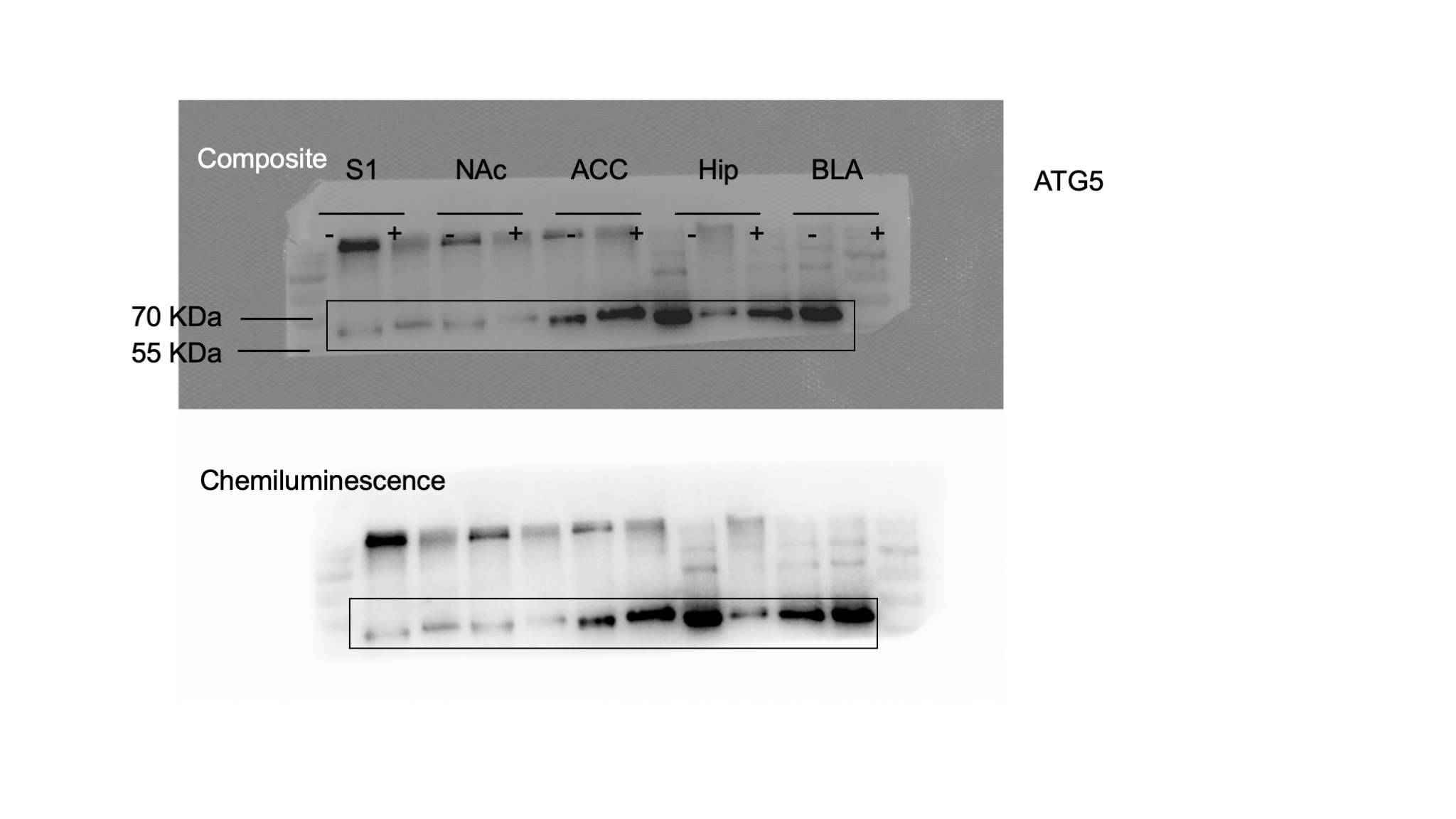

Supplement: Supplementary file 12 — Appendix Figure S2 Source Data [file 44319_2025_646_MOESM12_ESM.zip › Appendix Figure S2/S2A/S2A-ATG5.tiff]

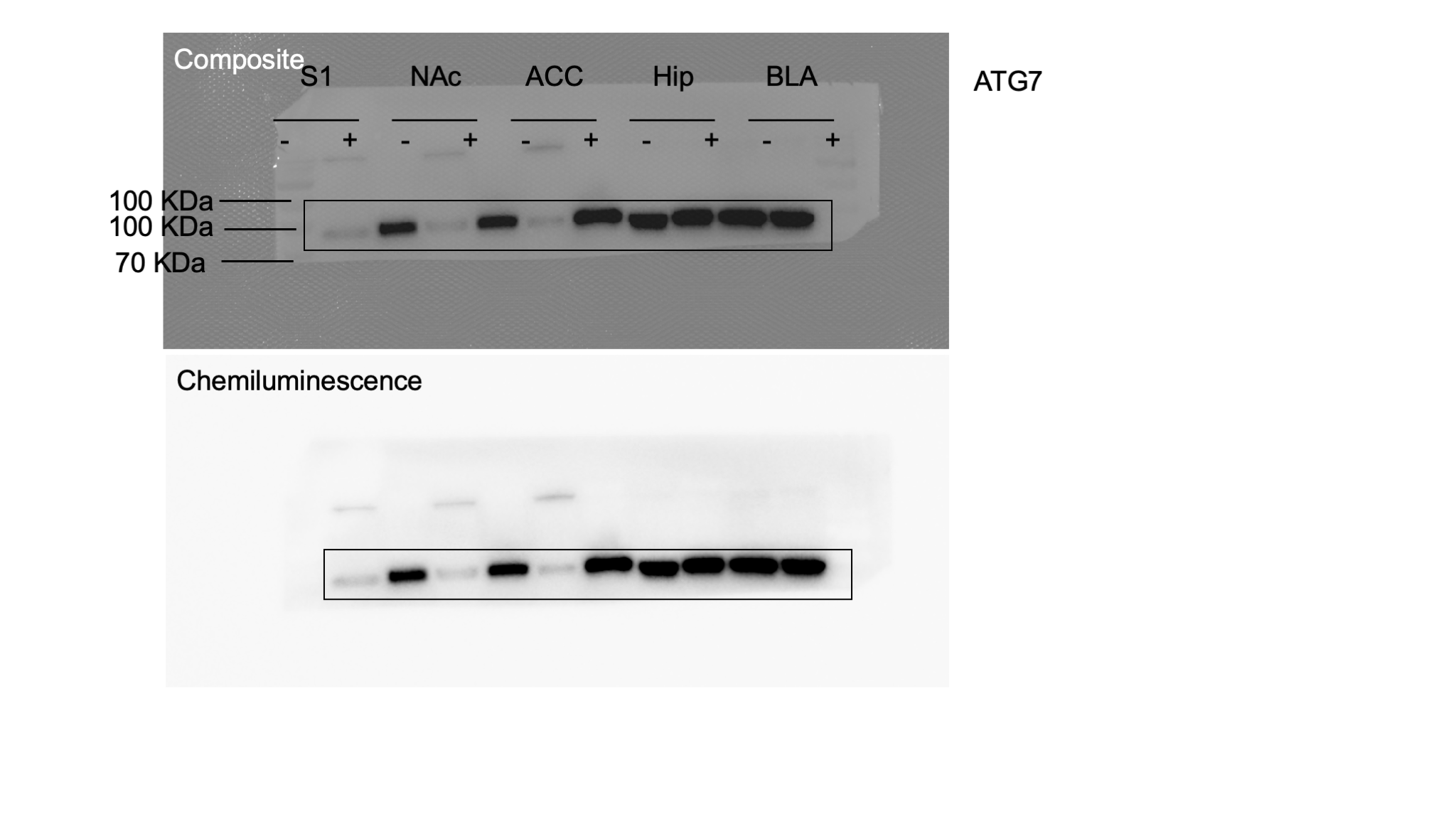

Supplement: Supplementary file 12 — Appendix Figure S2 Source Data [file 44319_2025_646_MOESM12_ESM.zip › Appendix Figure S2/S2A/S2A-ATG7.tiff]

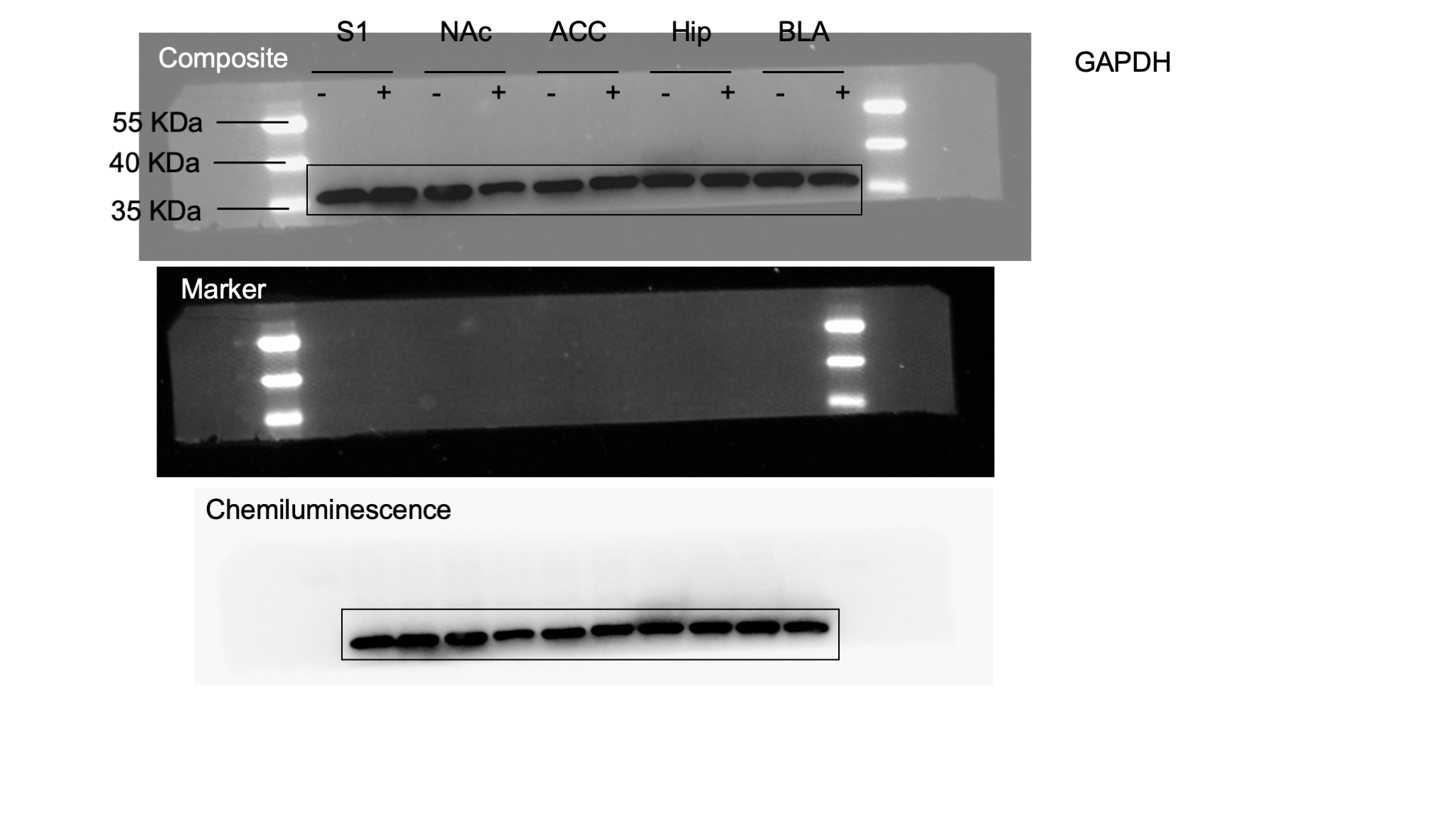

Supplement: Supplementary file 12 — Appendix Figure S2 Source Data [file 44319_2025_646_MOESM12_ESM.zip › Appendix Figure S2/S2A/S2A-GAPDH.tiff]

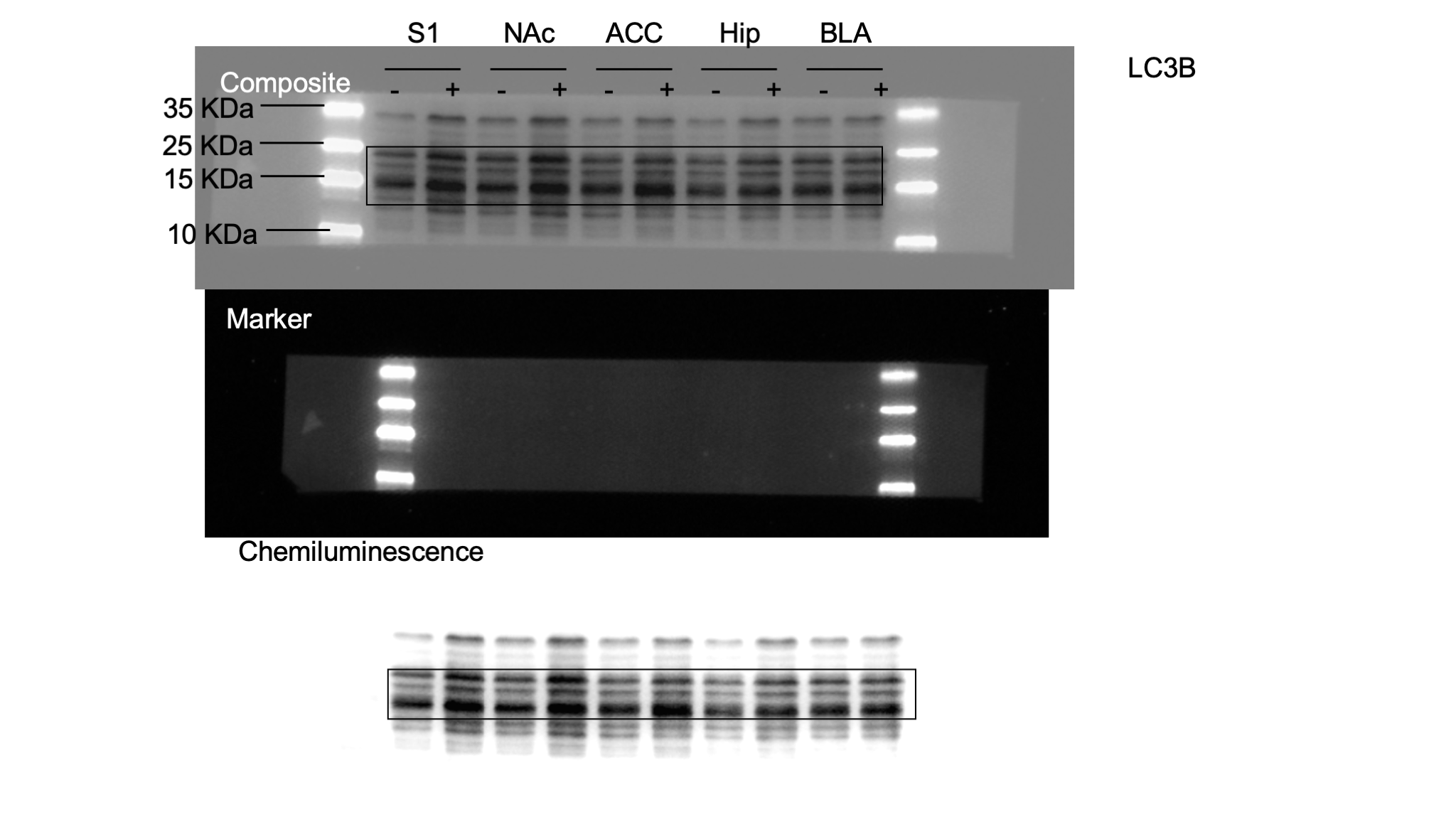

Supplement: Supplementary file 12 — Appendix Figure S2 Source Data [file 44319_2025_646_MOESM12_ESM.zip › Appendix Figure S2/S2A/S2A-LC3B.tiff]

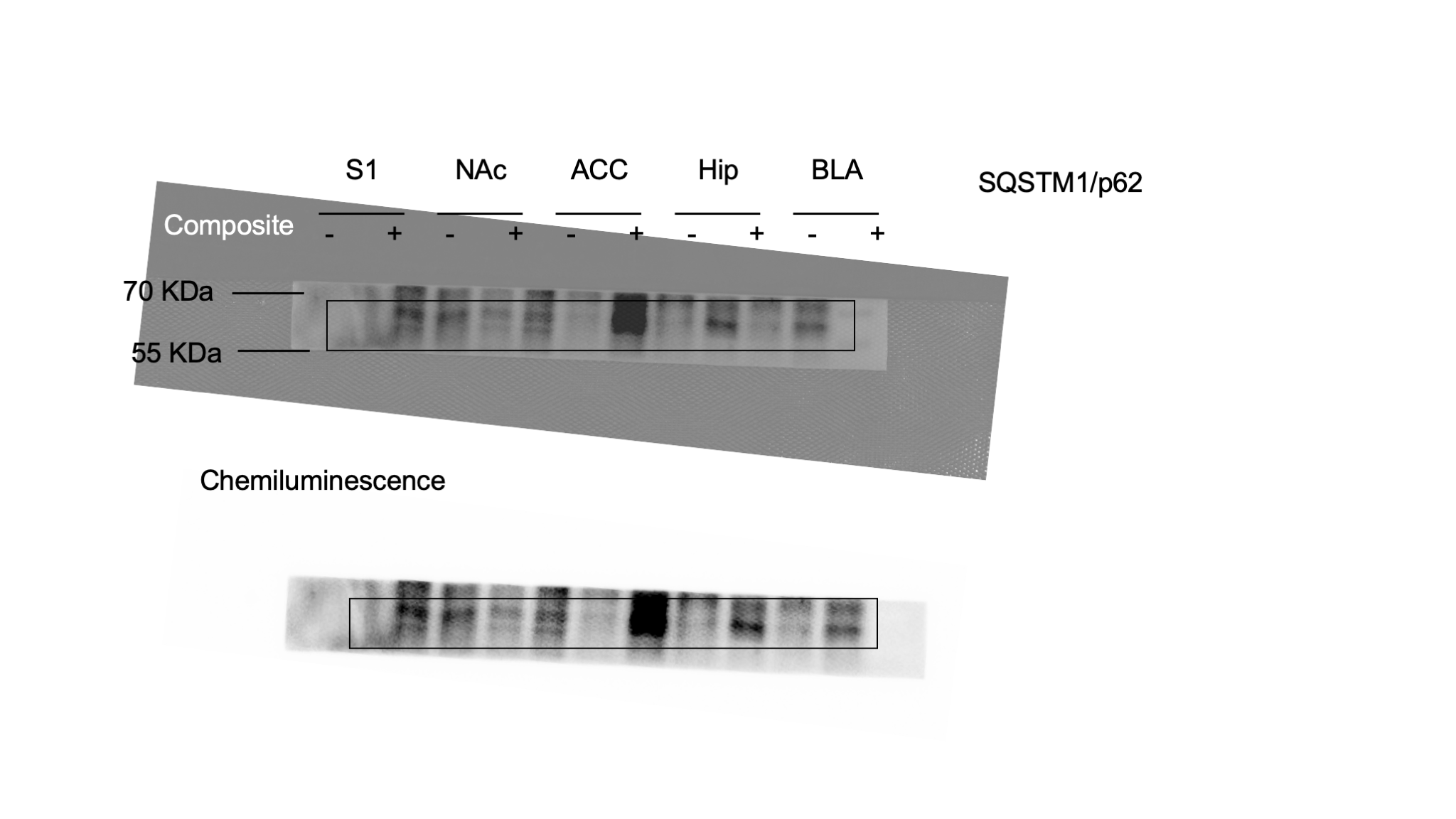

Supplement: Supplementary file 12 — Appendix Figure S2 Source Data [file 44319_2025_646_MOESM12_ESM.zip › Appendix Figure S2/S2A/S2A-SQSTM1.tiff]

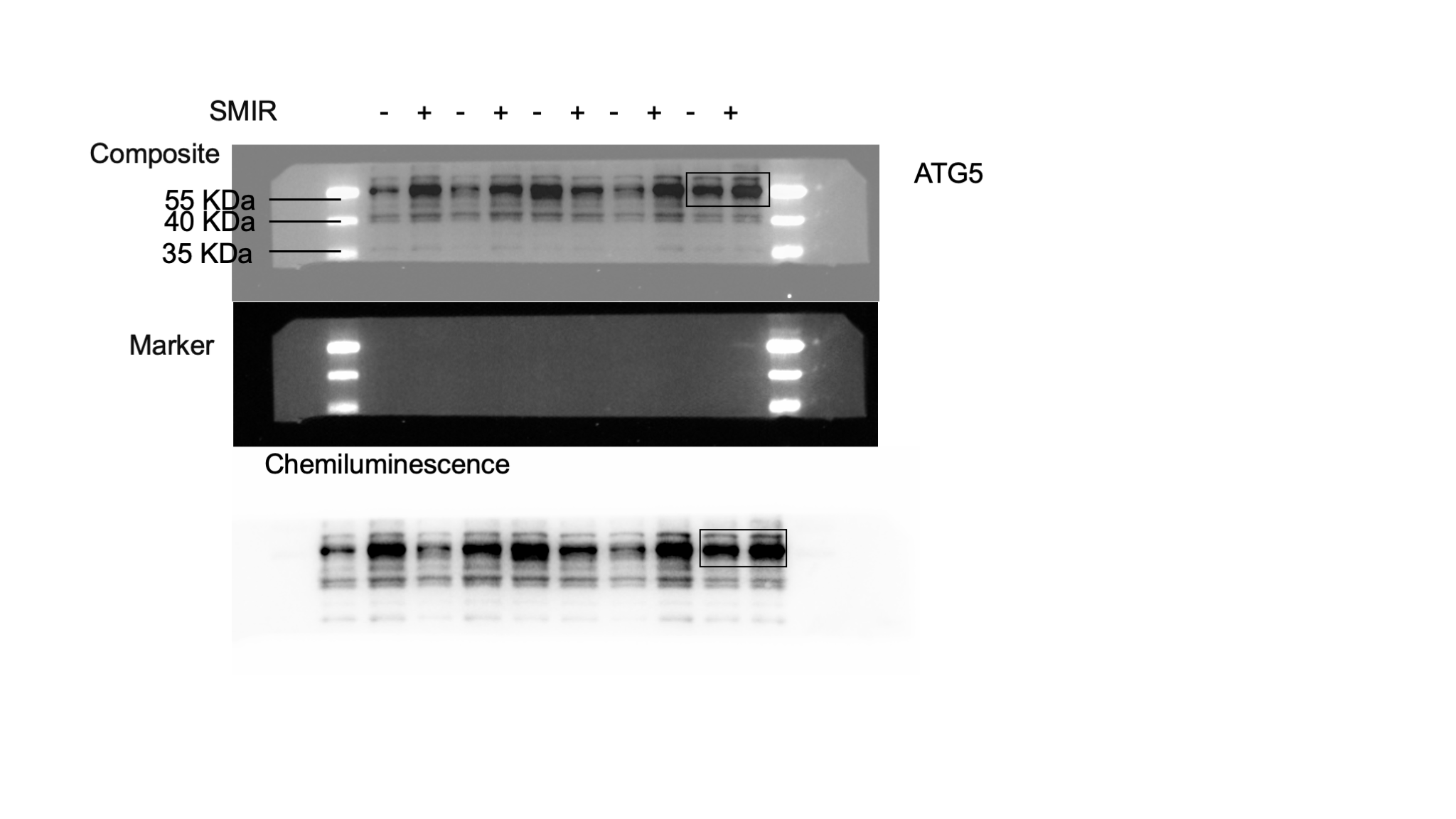

Supplement: Supplementary file 12 — Appendix Figure S2 Source Data [file 44319_2025_646_MOESM12_ESM.zip › Appendix Figure S2/S2B/S2B-ATG5.tiff]

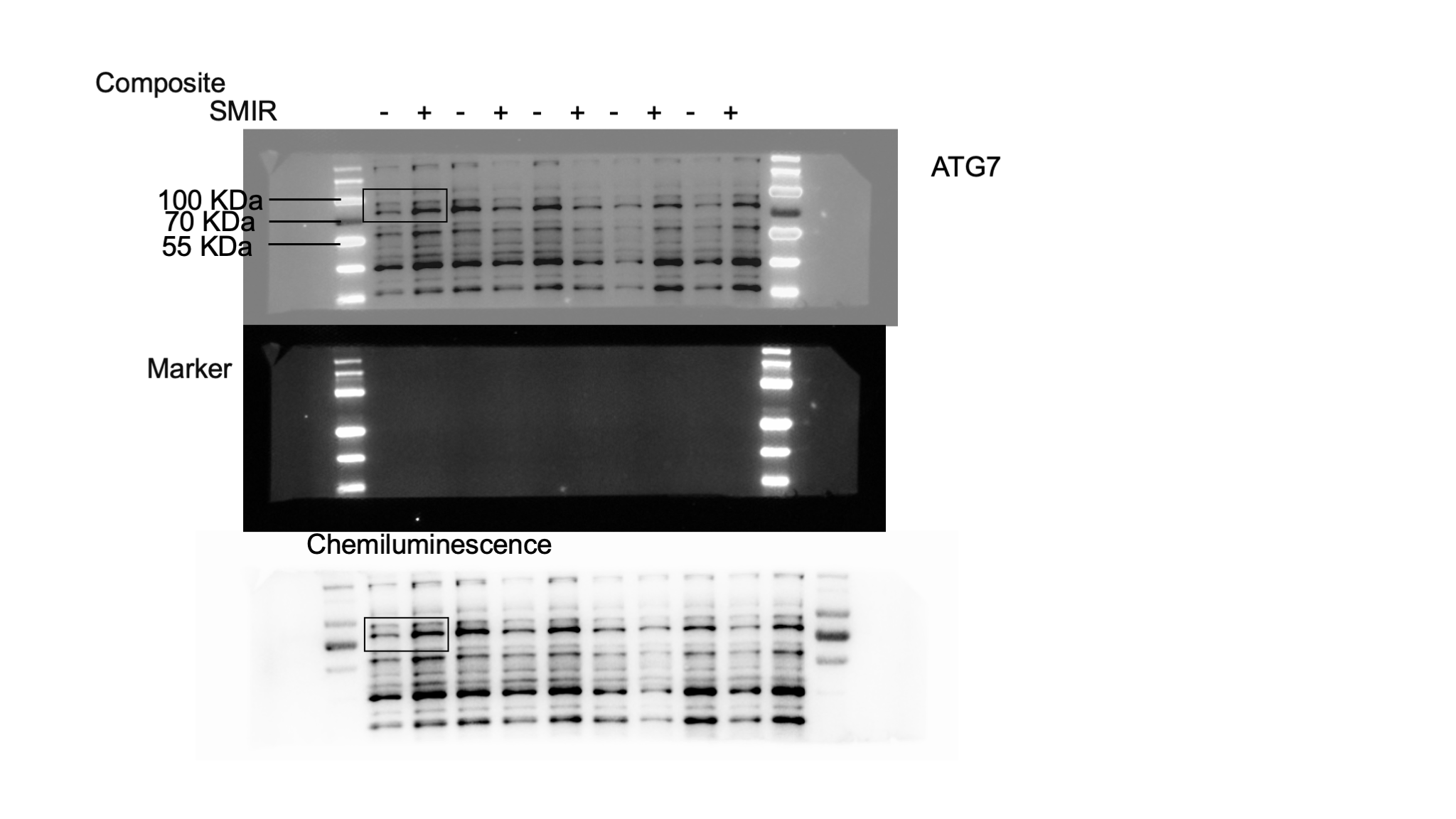

Supplement: Supplementary file 12 — Appendix Figure S2 Source Data [file 44319_2025_646_MOESM12_ESM.zip › Appendix Figure S2/S2B/S2B-ATG7.tiff]

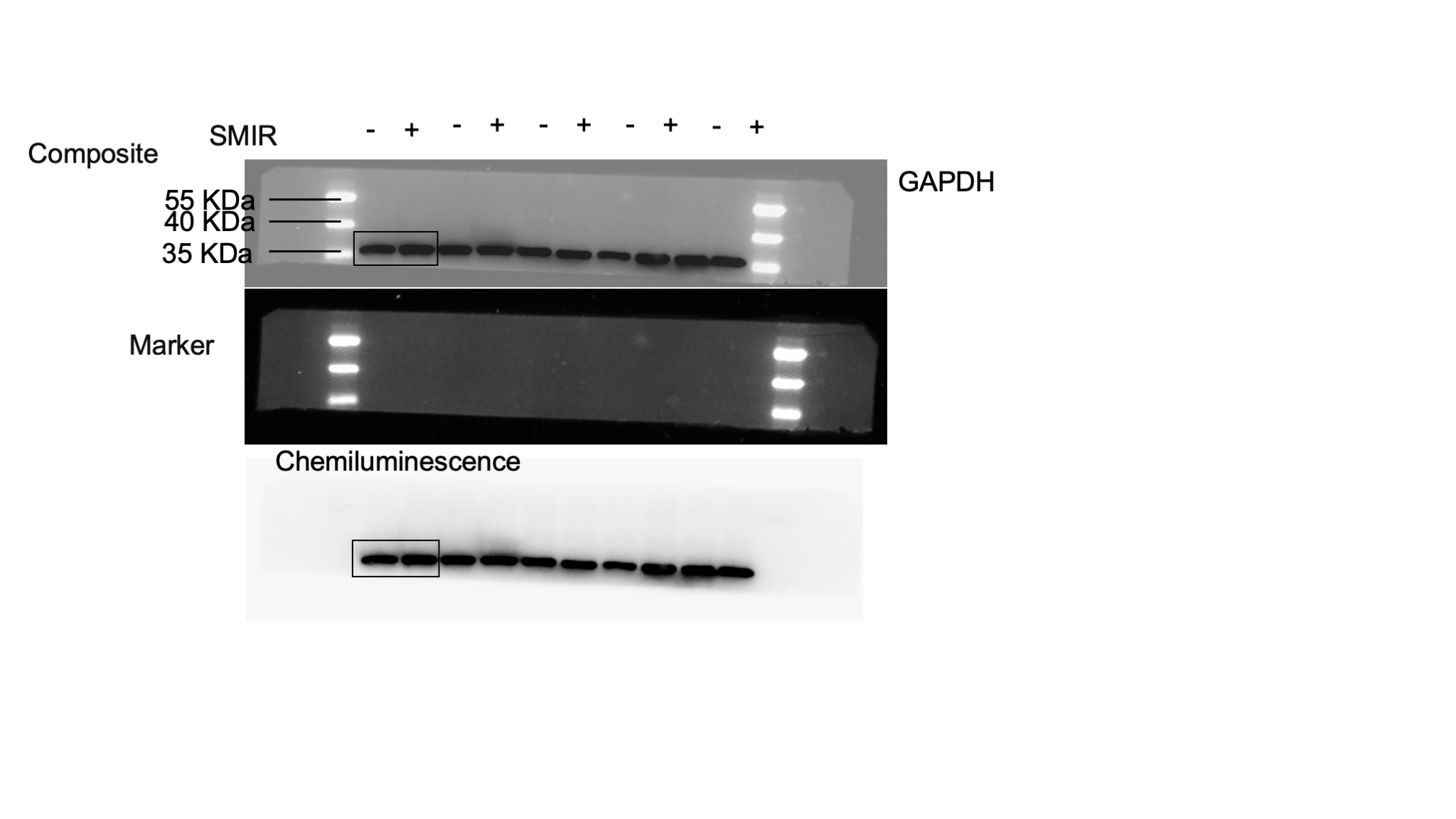

Supplement: Supplementary file 12 — Appendix Figure S2 Source Data [file 44319_2025_646_MOESM12_ESM.zip › Appendix Figure S2/S2B/S2B-GAPDH.tiff]

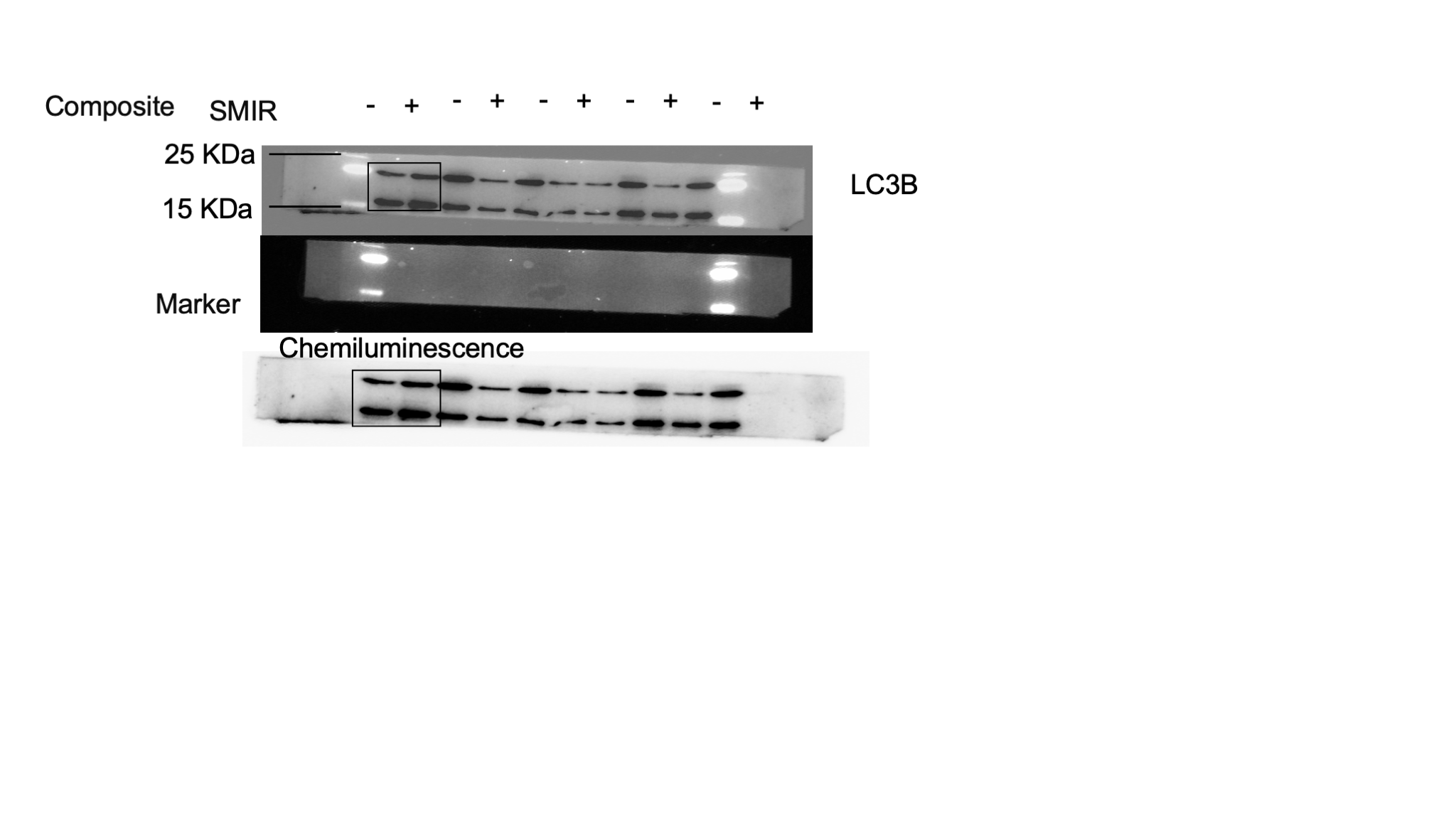

Supplement: Supplementary file 12 — Appendix Figure S2 Source Data [file 44319_2025_646_MOESM12_ESM.zip › Appendix Figure S2/S2B/S2B-LC3B.tiff]

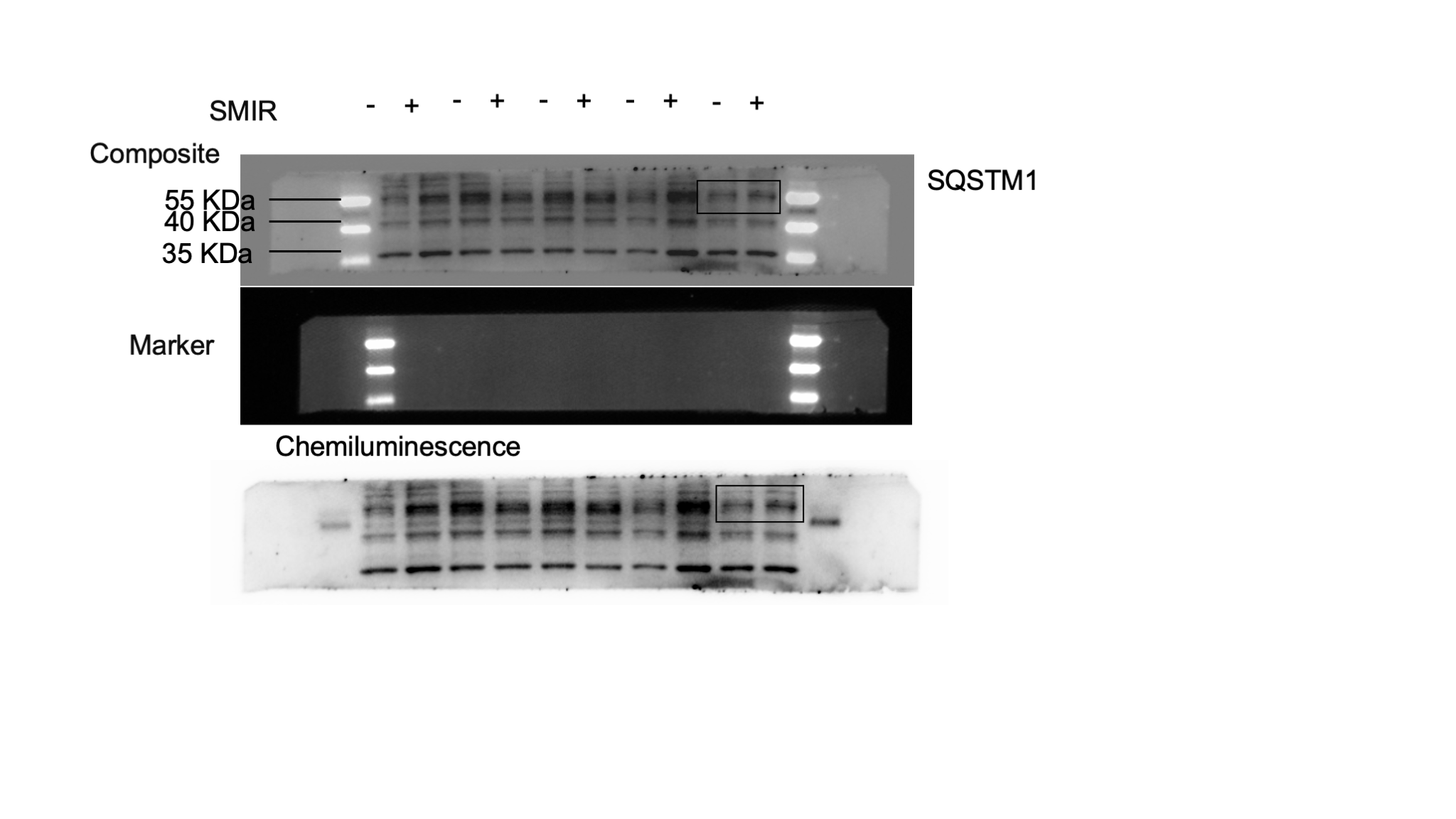

Supplement: Supplementary file 12 — Appendix Figure S2 Source Data [file 44319_2025_646_MOESM12_ESM.zip › Appendix Figure S2/S2B/S2B-SQSTM1.tiff]

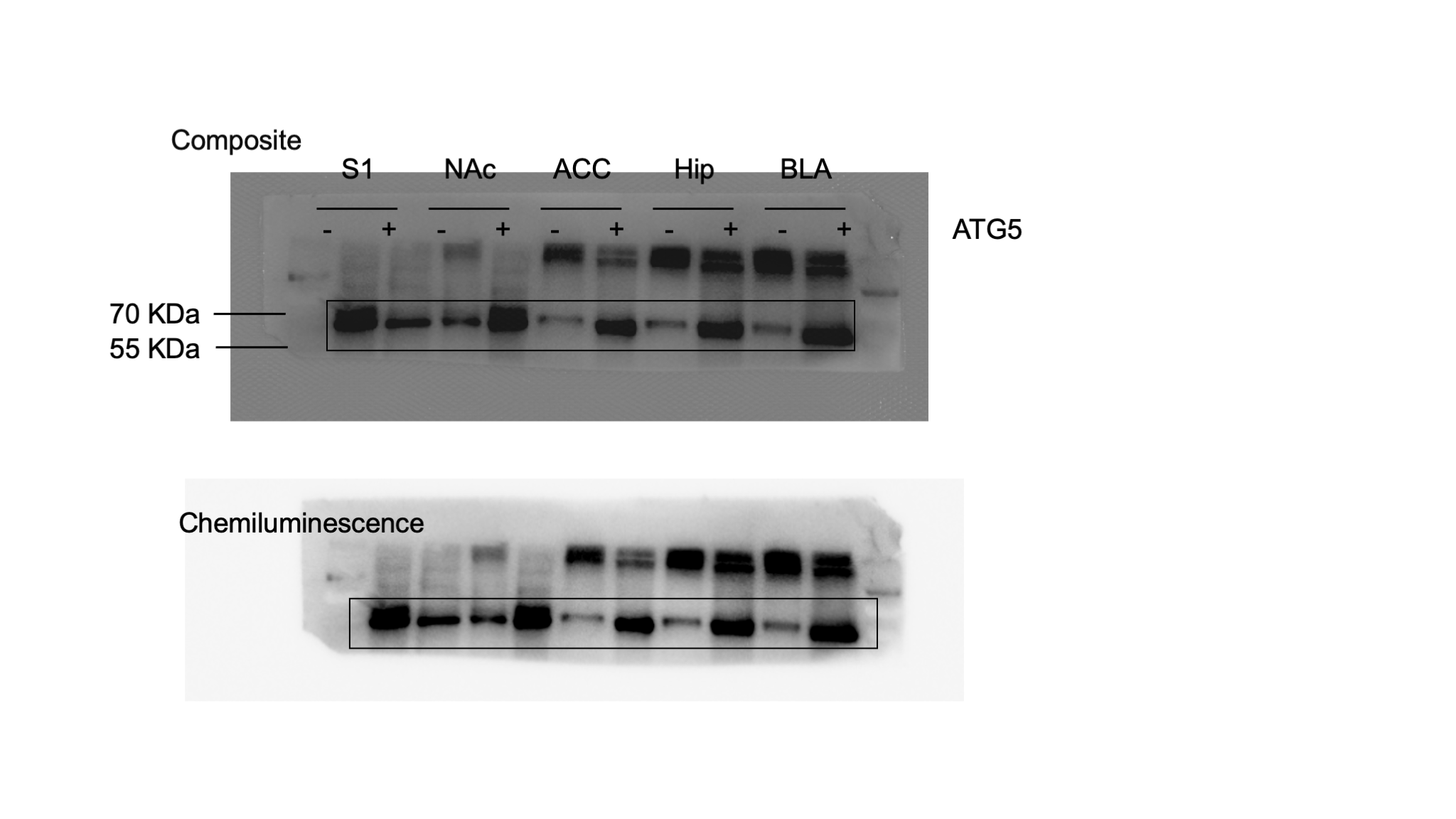

Supplement: Supplementary file 12 — Appendix Figure S2 Source Data [file 44319_2025_646_MOESM12_ESM.zip › Appendix Figure S2/S2C/S2C-ATG5.tiff]

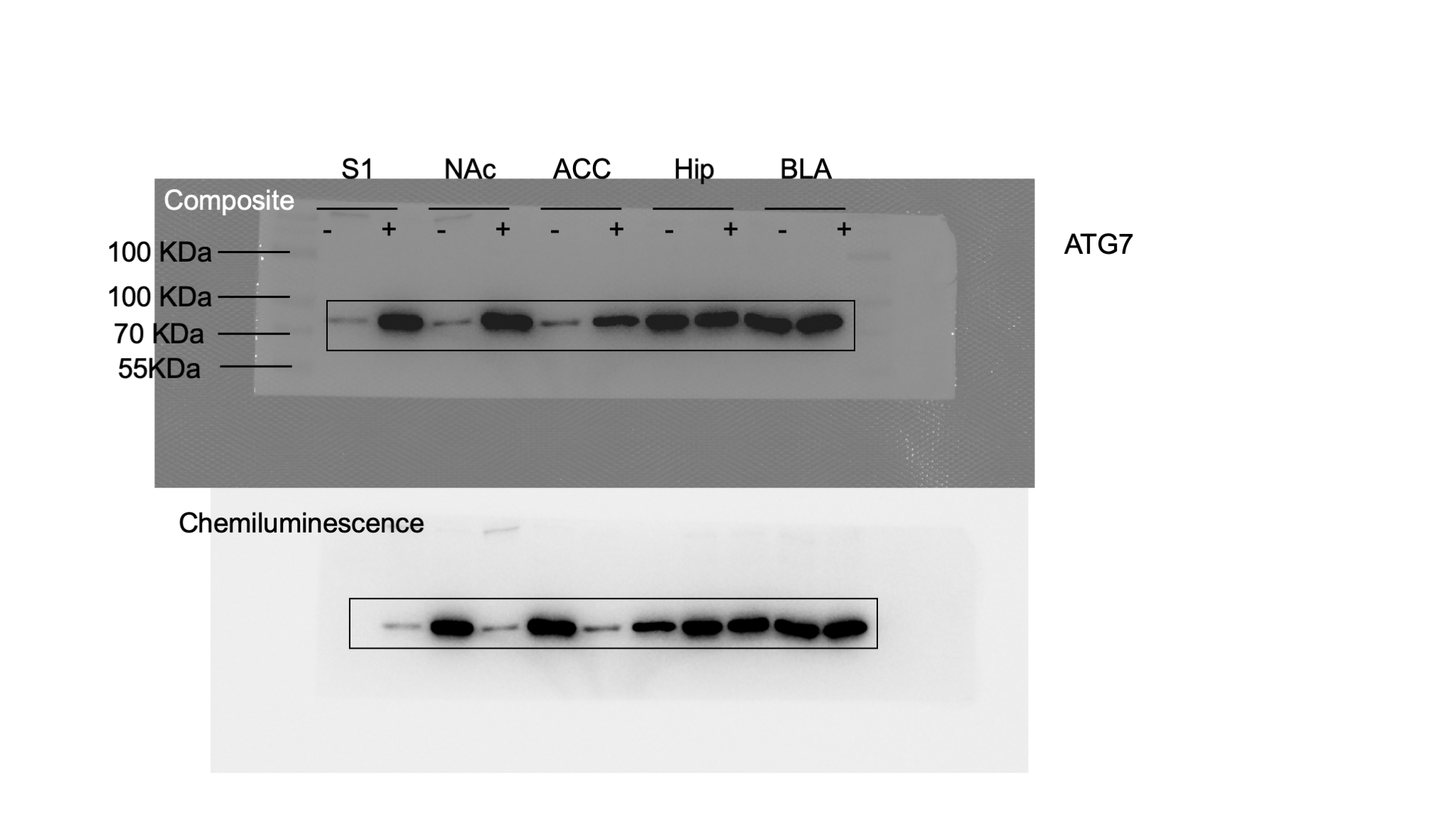

Supplement: Supplementary file 12 — Appendix Figure S2 Source Data [file 44319_2025_646_MOESM12_ESM.zip › Appendix Figure S2/S2C/S2C-ATG7.tiff]

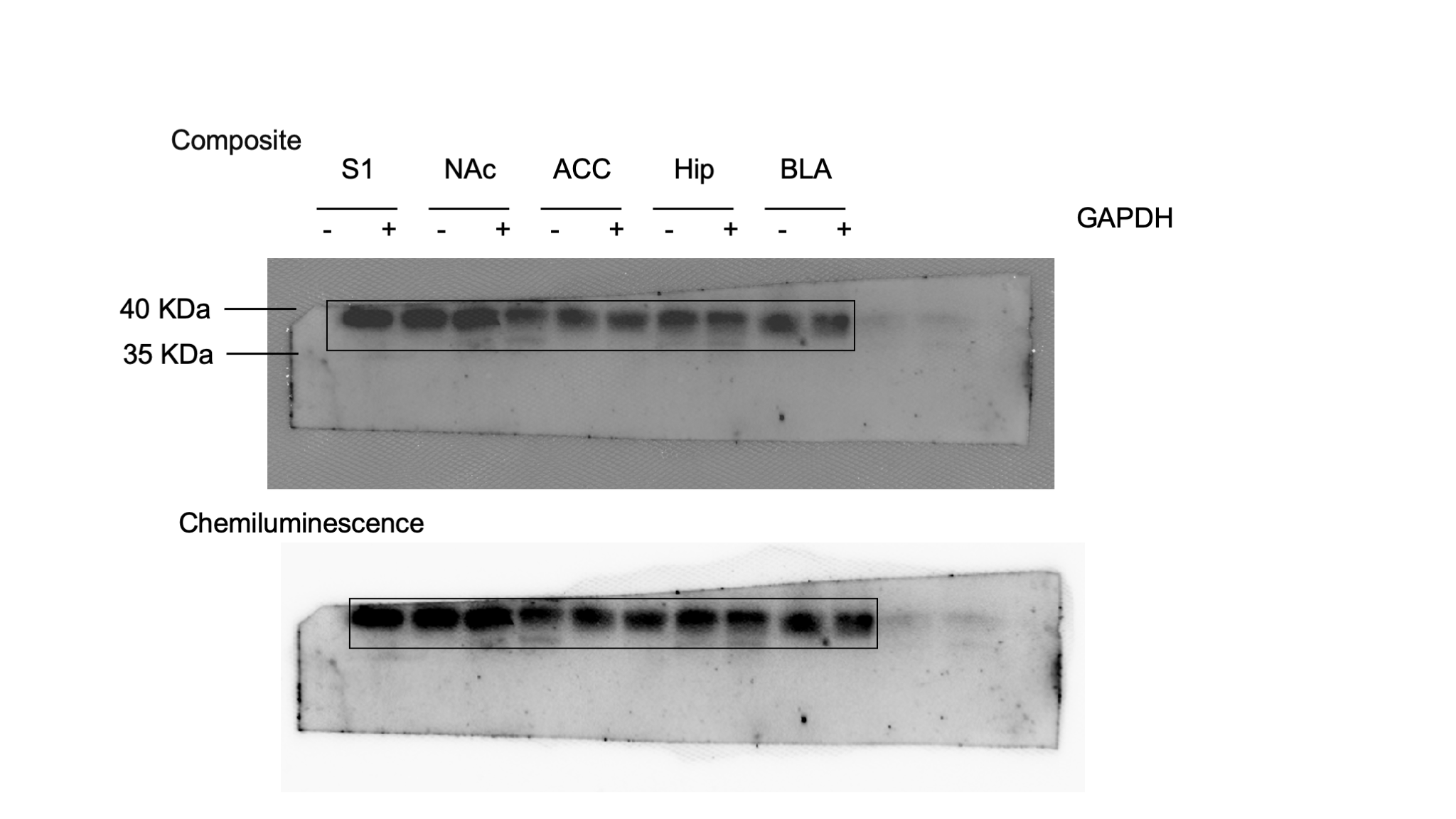

Supplement: Supplementary file 12 — Appendix Figure S2 Source Data [file 44319_2025_646_MOESM12_ESM.zip › Appendix Figure S2/S2C/S2C-GAPDH.tiff]

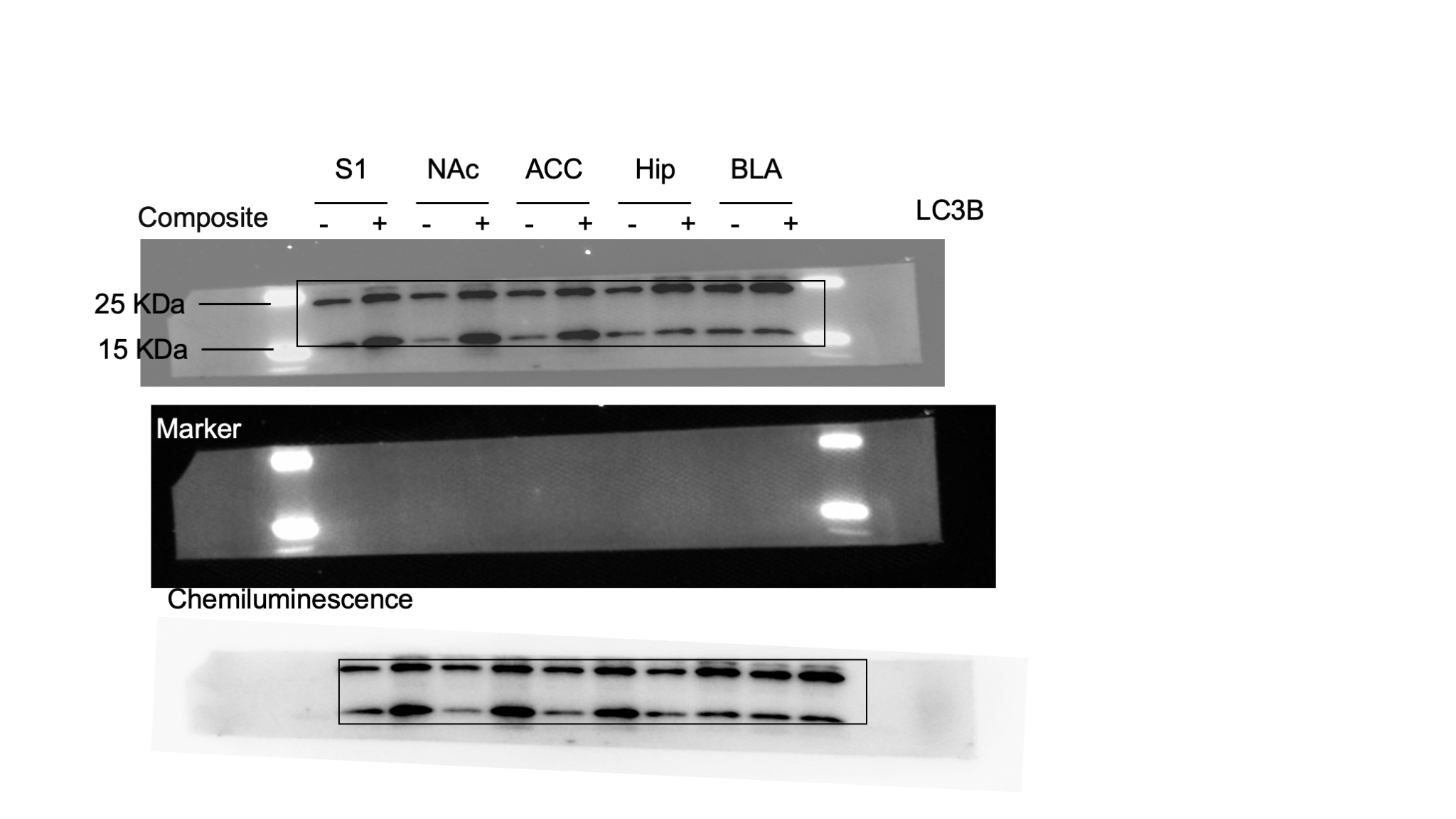

Supplement: Supplementary file 12 — Appendix Figure S2 Source Data [file 44319_2025_646_MOESM12_ESM.zip › Appendix Figure S2/S2C/S2C-LC3B.tiff]

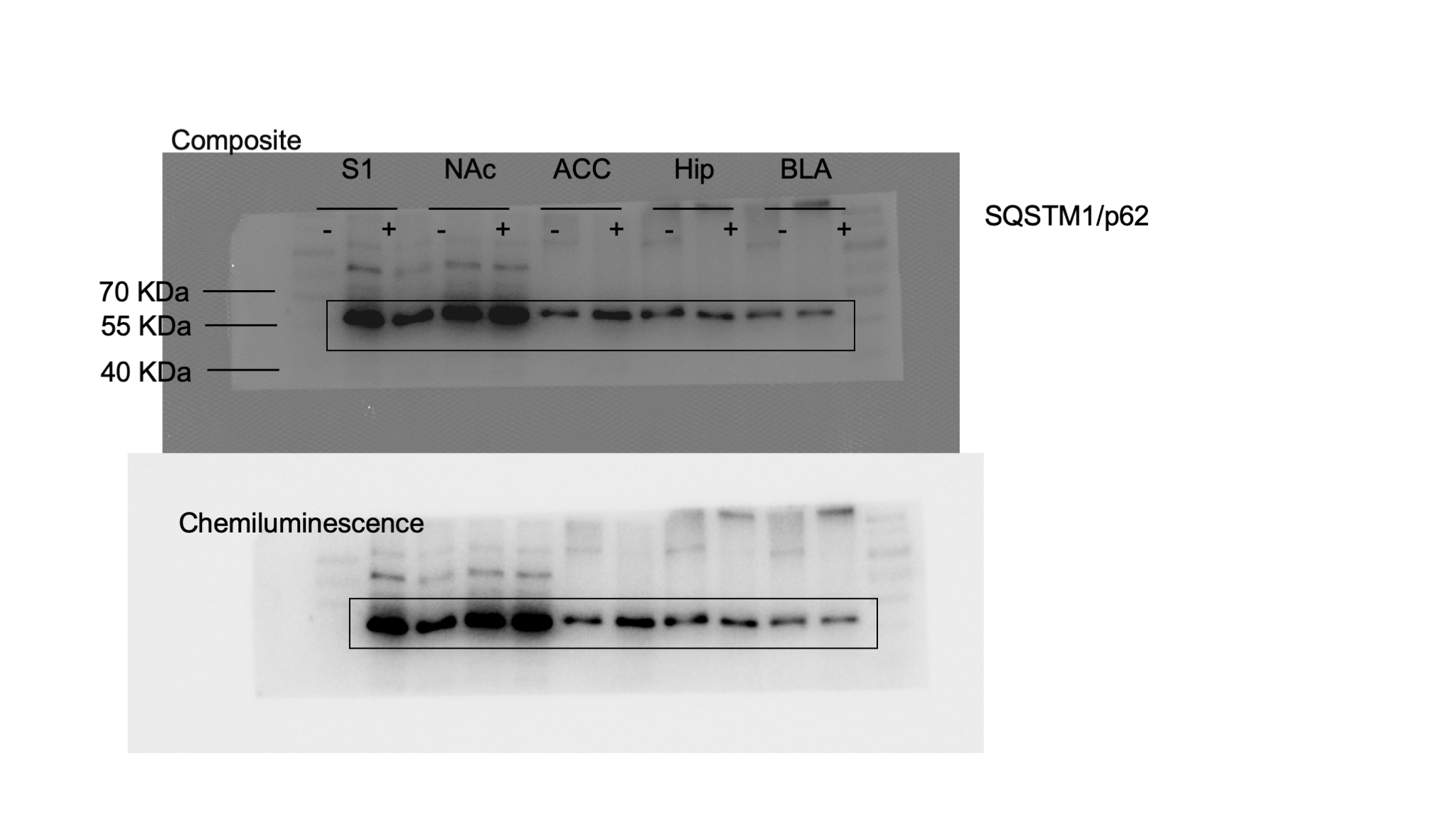

Supplement: Supplementary file 12 — Appendix Figure S2 Source Data [file 44319_2025_646_MOESM12_ESM.zip › Appendix Figure S2/S2C/S2C-SQSTM1.tiff]

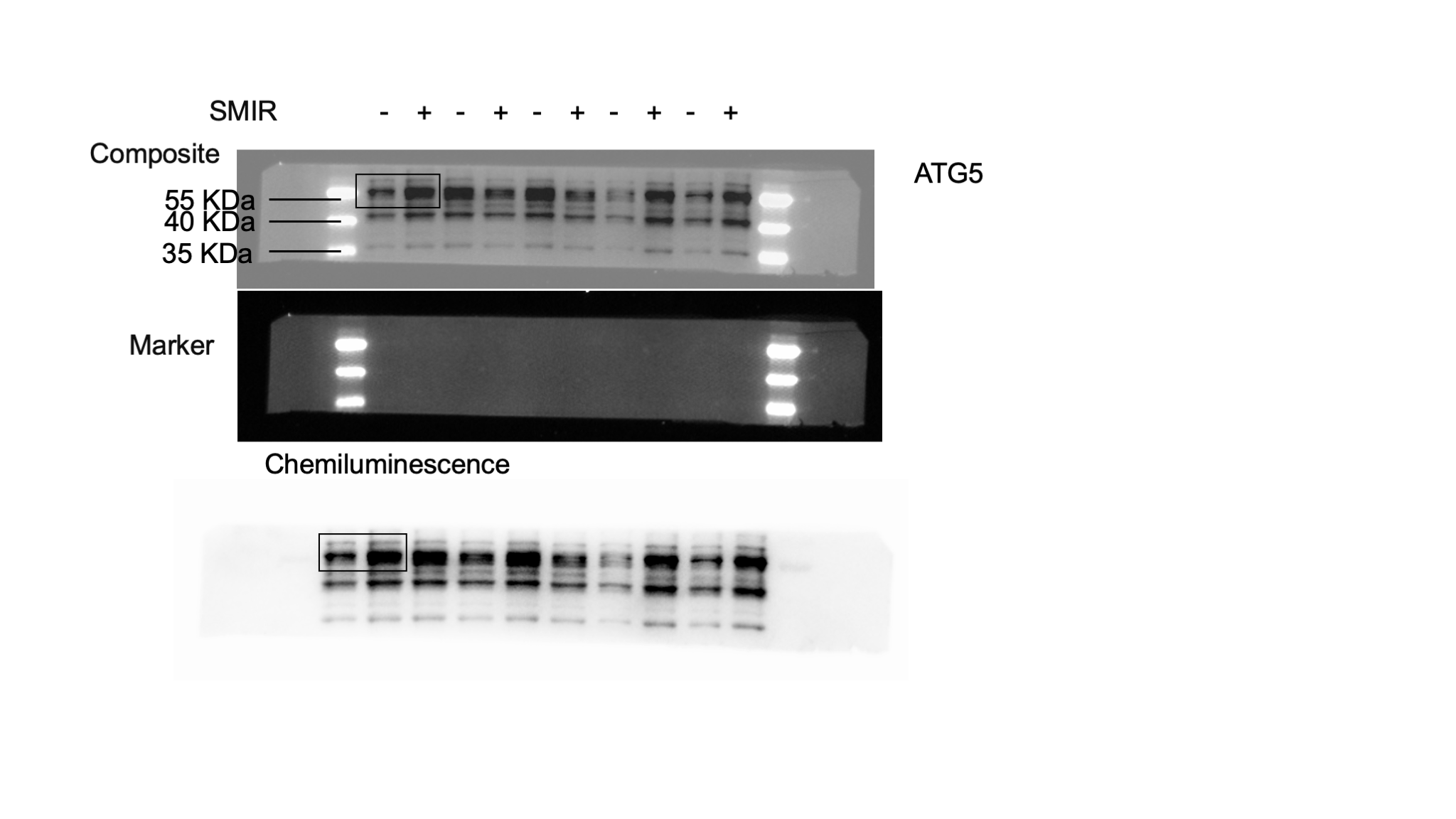

Supplement: Supplementary file 12 — Appendix Figure S2 Source Data [file 44319_2025_646_MOESM12_ESM.zip › Appendix Figure S2/S2D/S2D-ATG5.tiff]

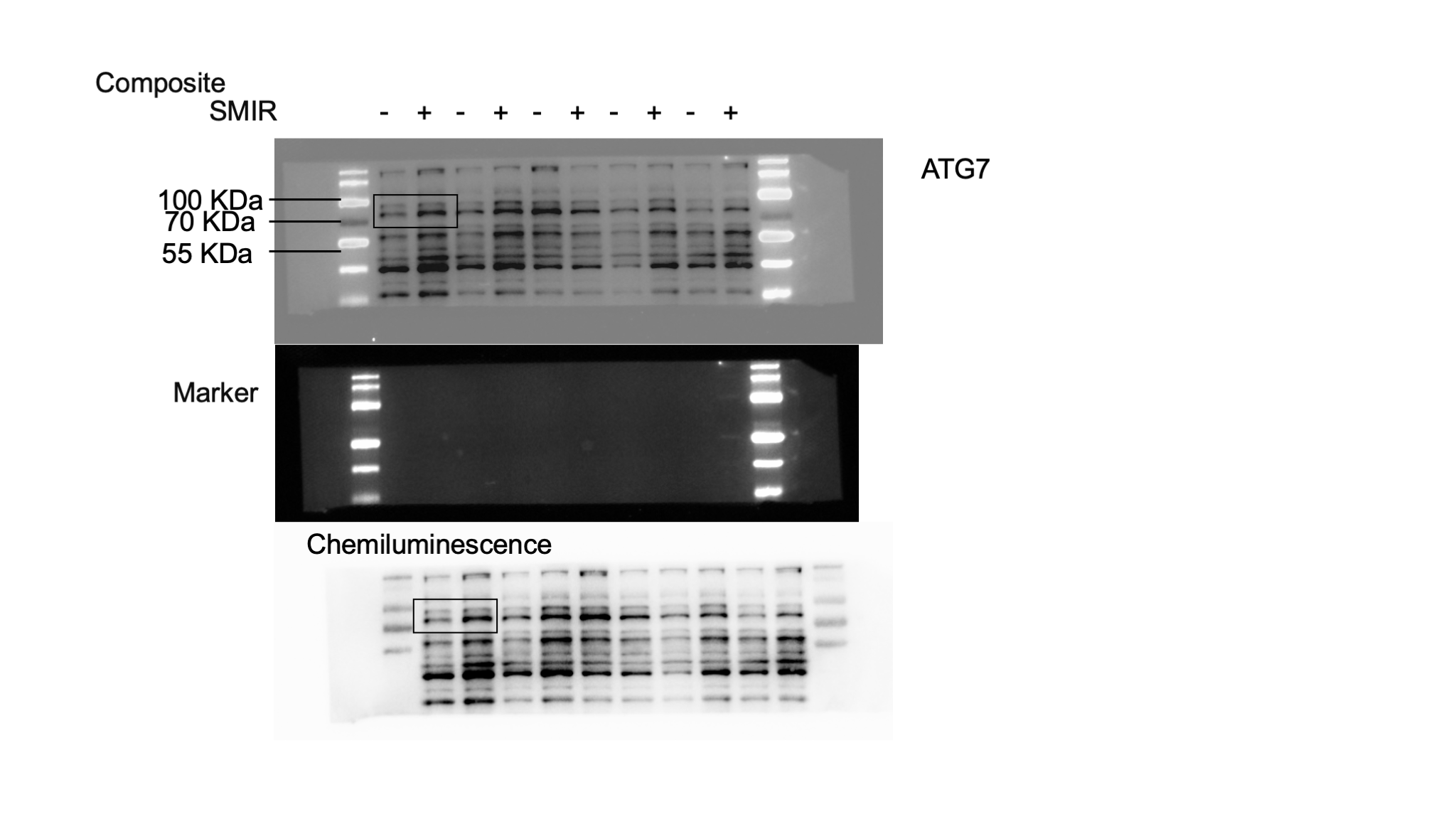

Supplement: Supplementary file 12 — Appendix Figure S2 Source Data [file 44319_2025_646_MOESM12_ESM.zip › Appendix Figure S2/S2D/S2D-ATG7.tiff]

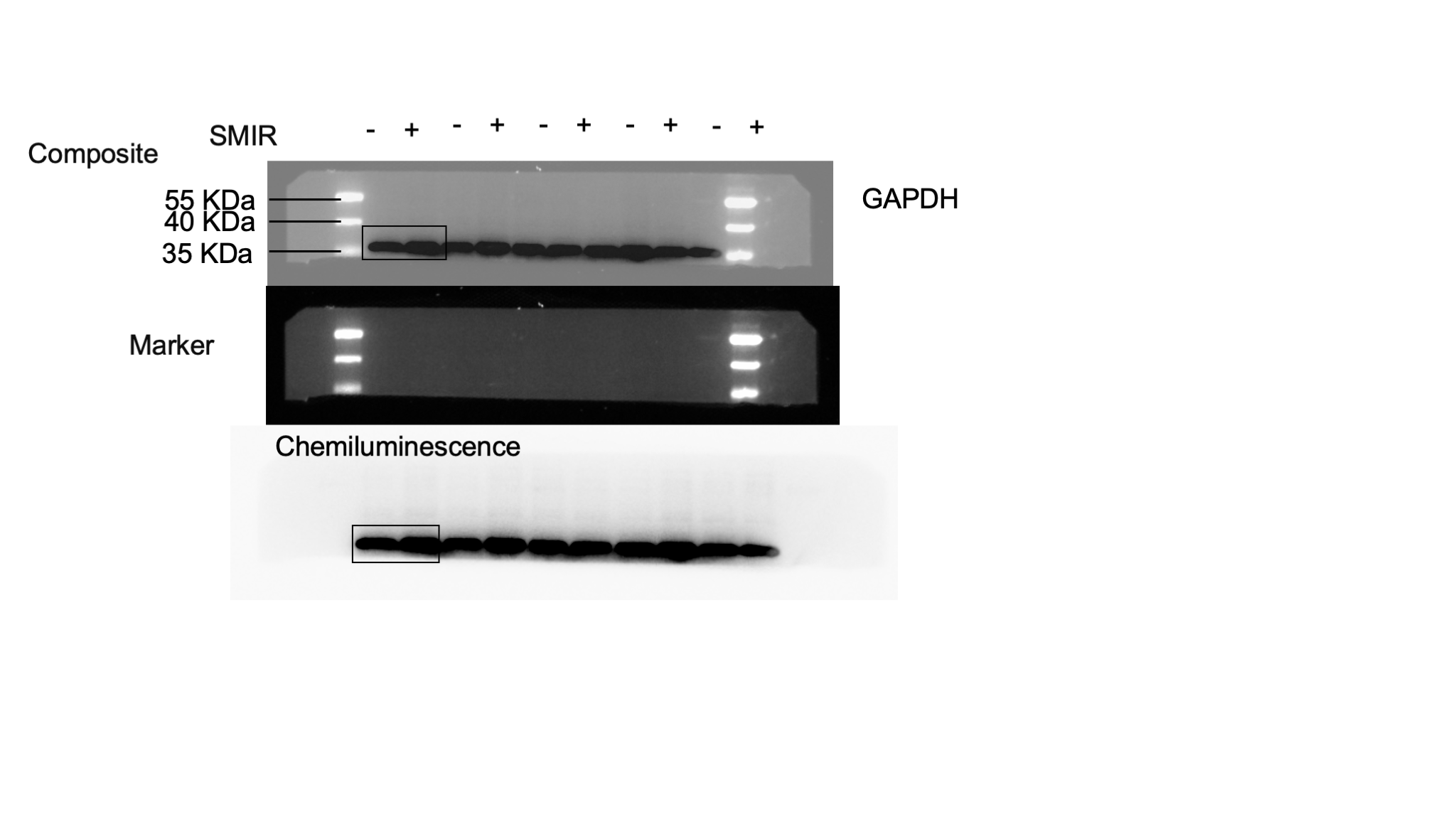

Supplement: Supplementary file 12 — Appendix Figure S2 Source Data [file 44319_2025_646_MOESM12_ESM.zip › Appendix Figure S2/S2D/S2D-GAPDH.tiff]

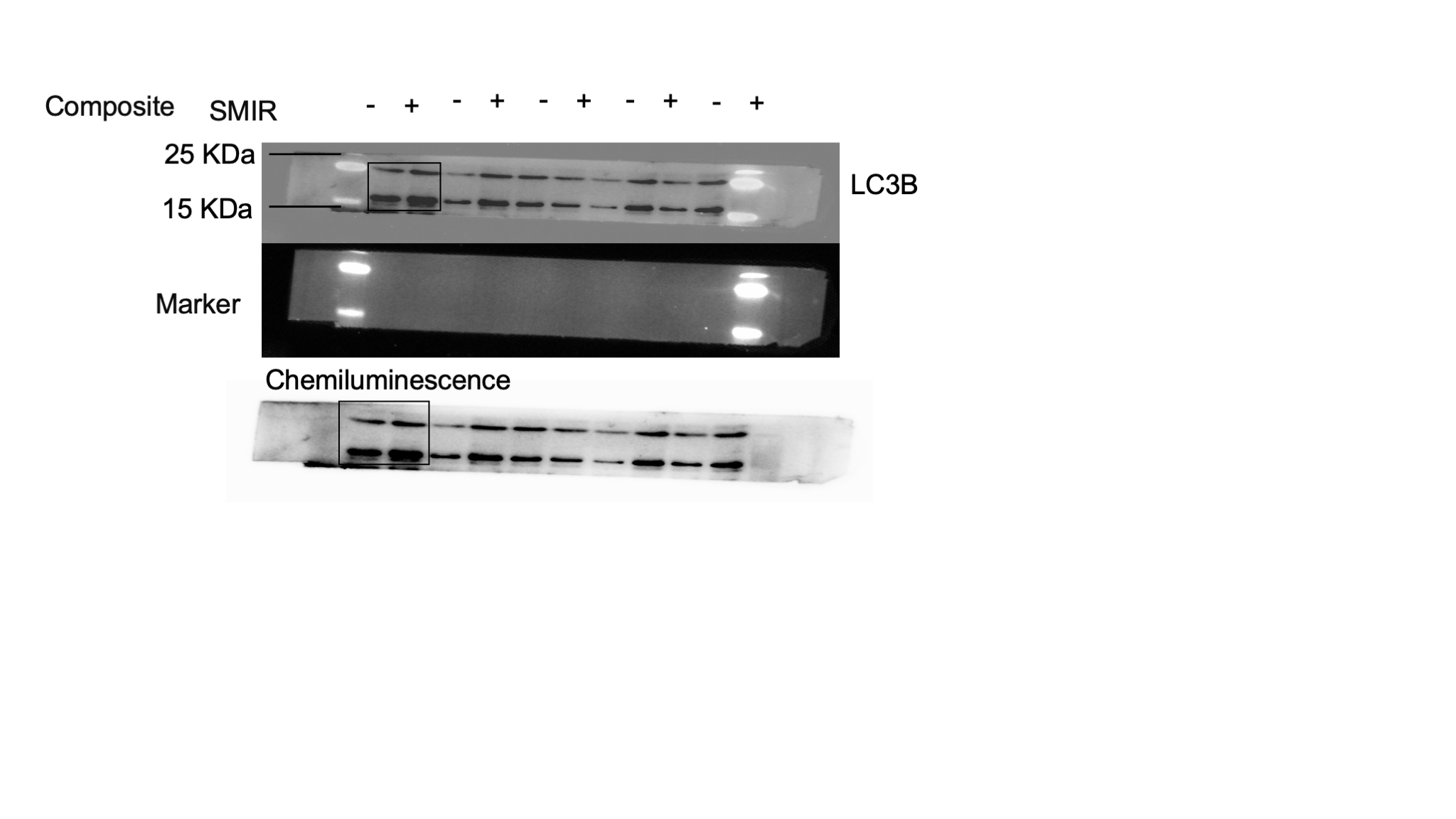

Supplement: Supplementary file 12 — Appendix Figure S2 Source Data [file 44319_2025_646_MOESM12_ESM.zip › Appendix Figure S2/S2D/S2D-LC3B.tiff]

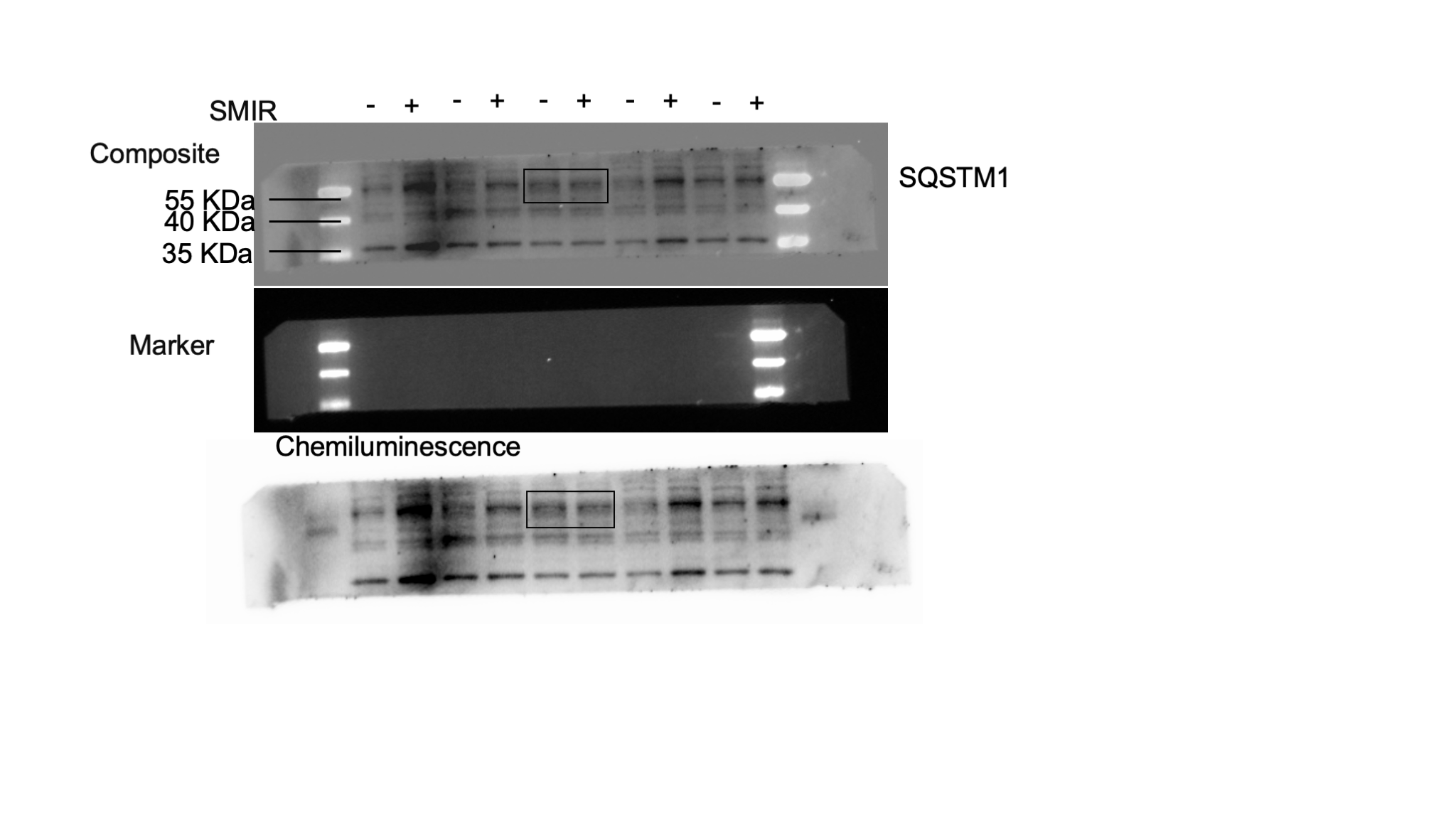

Supplement: Supplementary file 12 — Appendix Figure S2 Source Data [file 44319_2025_646_MOESM12_ESM.zip › Appendix Figure S2/S2D/S2D-SQSTM1.tiff]

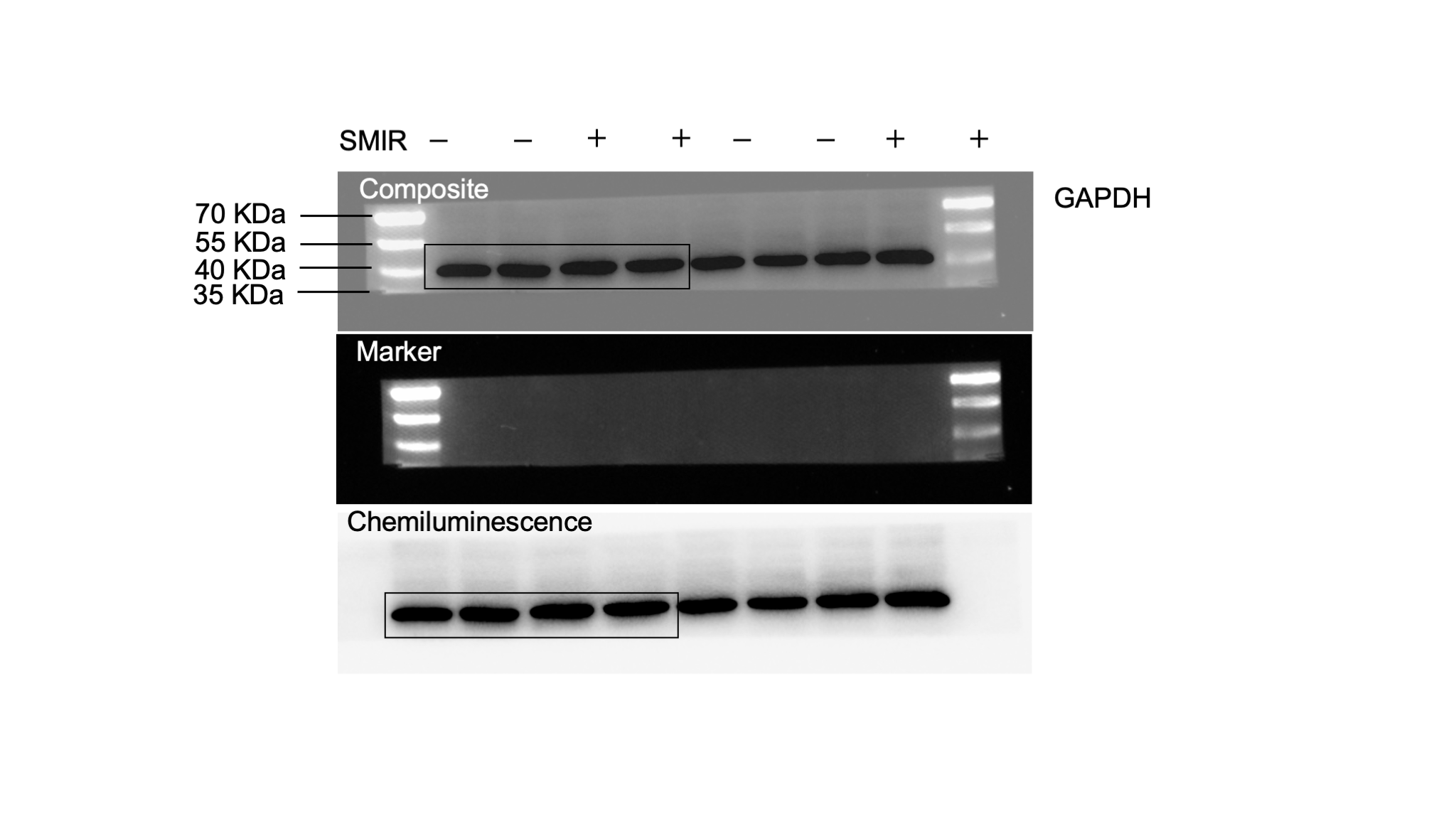

Supplement: Supplementary file 12 — Appendix Figure S2 Source Data [file 44319_2025_646_MOESM12_ESM.zip › Appendix Figure S2/S2E/S2E-GAPDH.tiff]

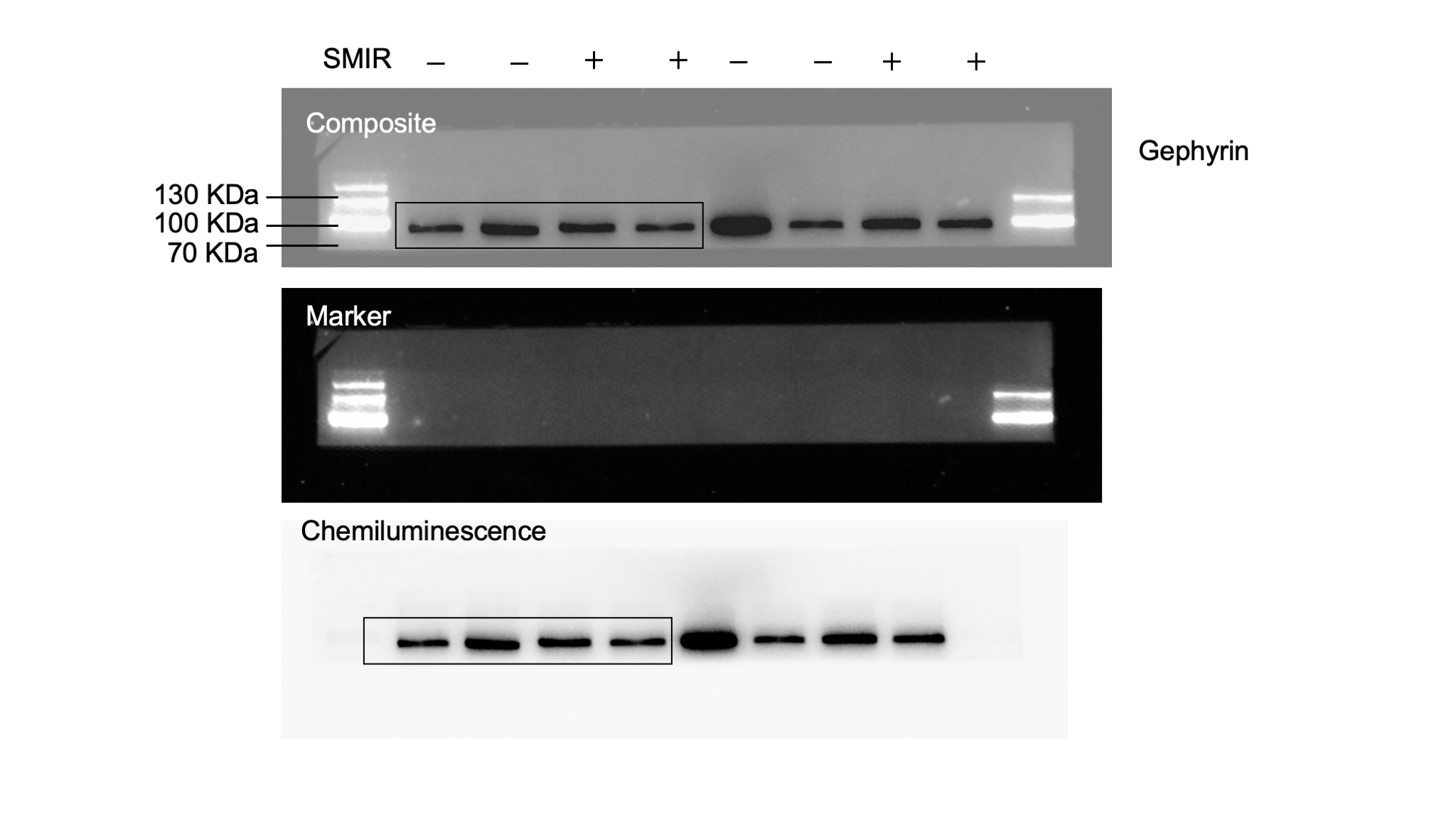

Supplement: Supplementary file 12 — Appendix Figure S2 Source Data [file 44319_2025_646_MOESM12_ESM.zip › Appendix Figure S2/S2E/S2E-Gephyrin.tiff]

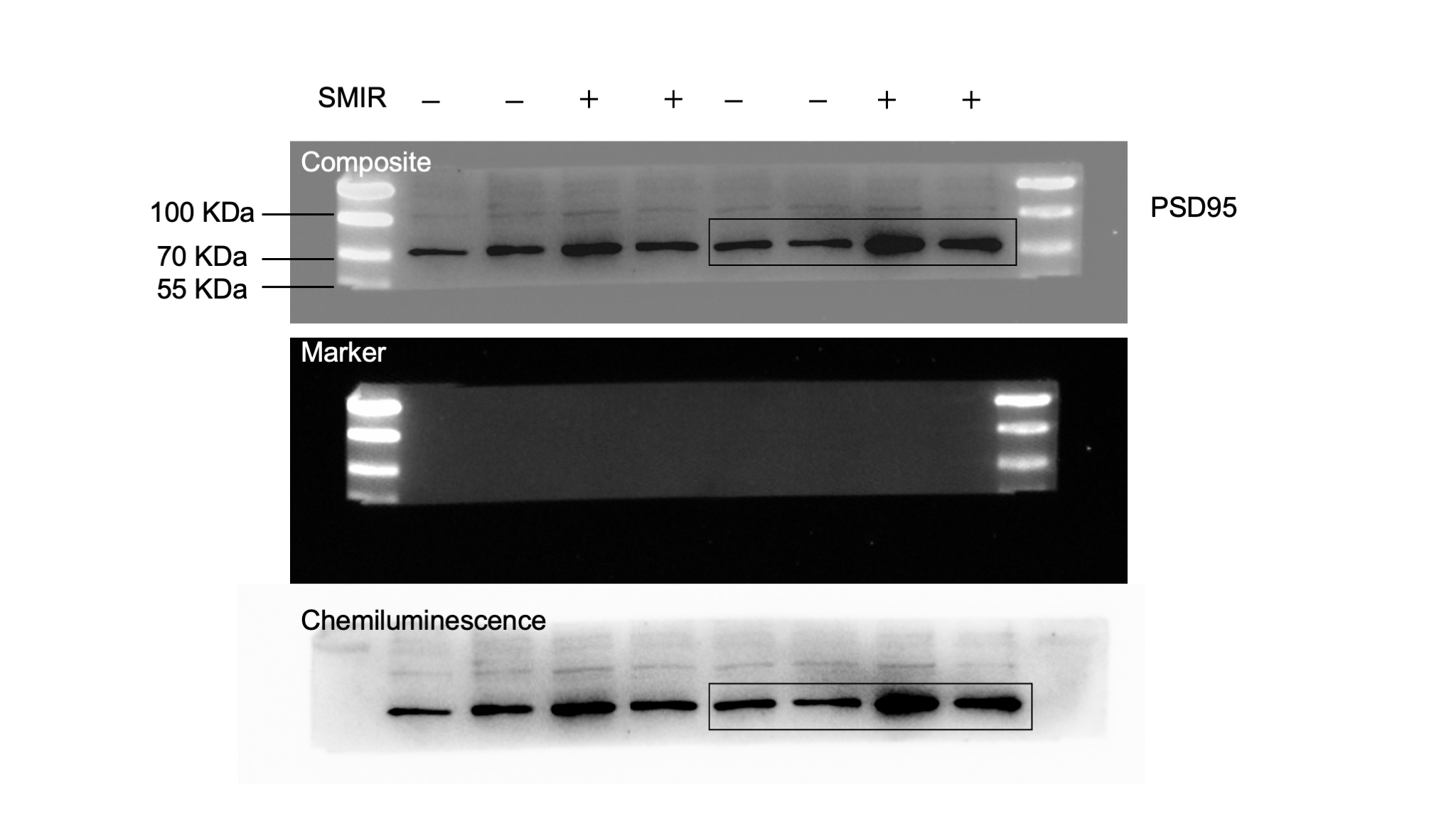

Supplement: Supplementary file 12 — Appendix Figure S2 Source Data [file 44319_2025_646_MOESM12_ESM.zip › Appendix Figure S2/S2E/S2E-PSD95.tiff]

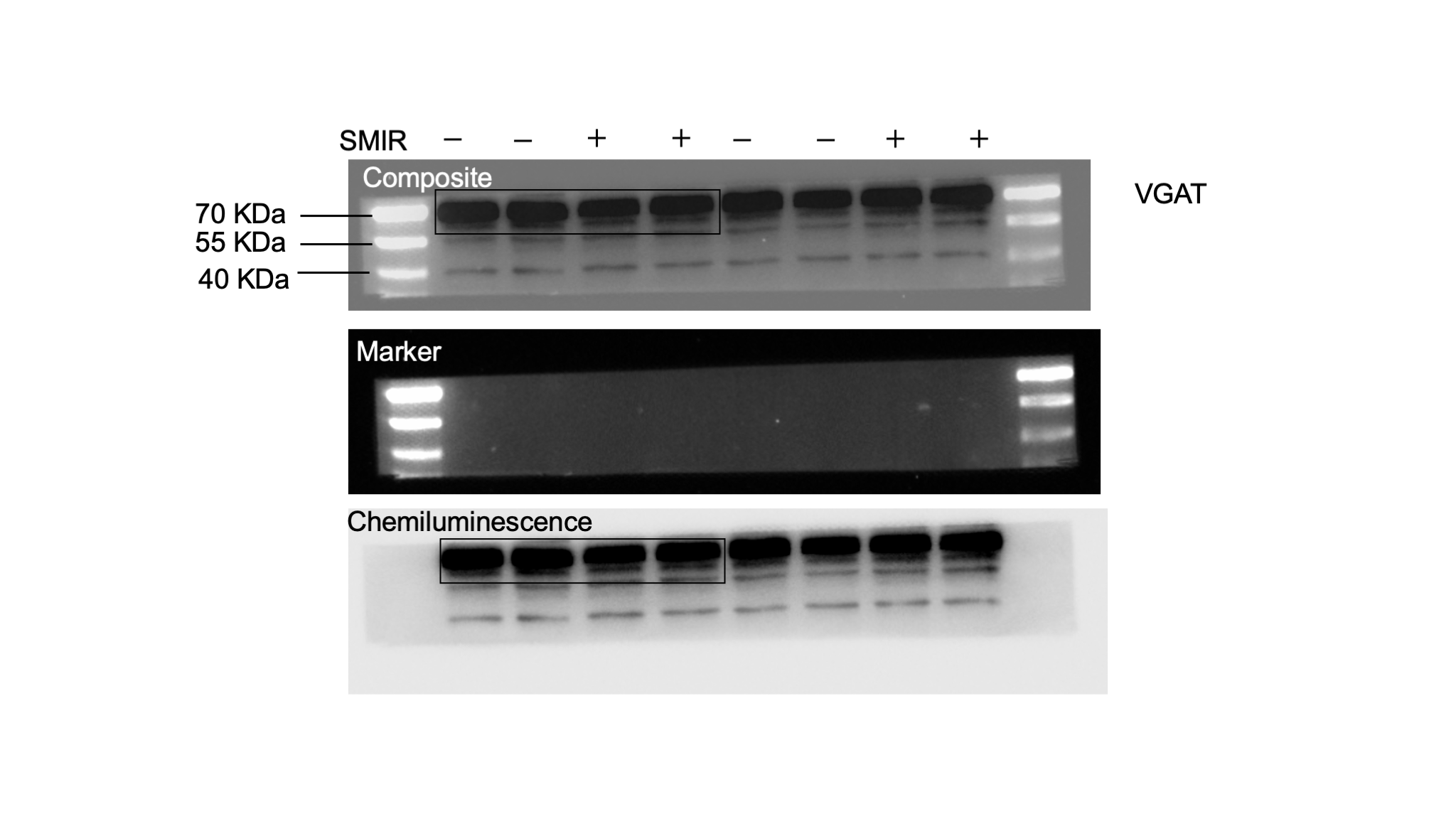

Supplement: Supplementary file 12 — Appendix Figure S2 Source Data [file 44319_2025_646_MOESM12_ESM.zip › Appendix Figure S2/S2E/S2E-VGAT.tiff]

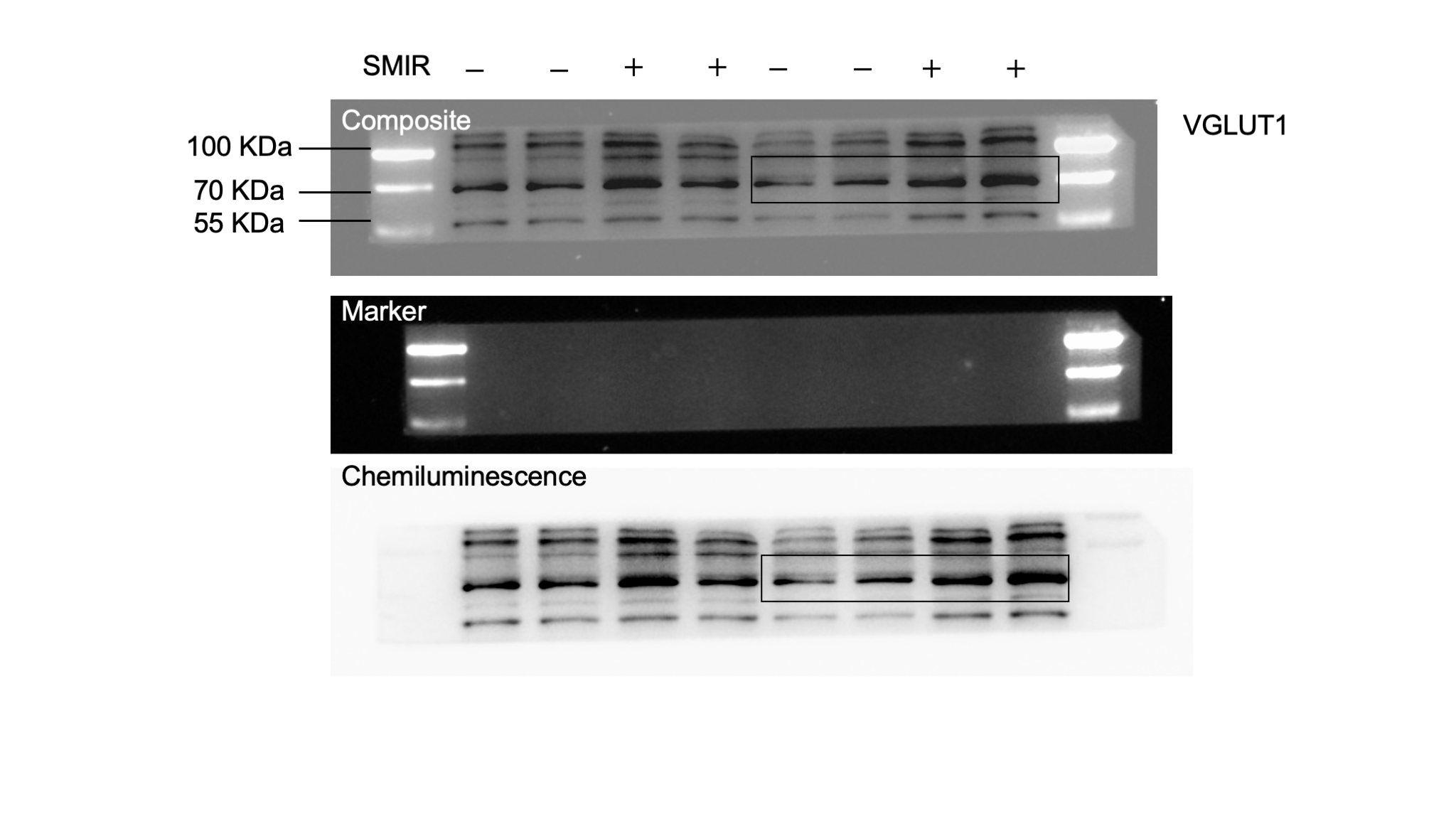

Supplement: Supplementary file 12 — Appendix Figure S2 Source Data [file 44319_2025_646_MOESM12_ESM.zip › Appendix Figure S2/S2E/S2E-VGLUT1.tiff]

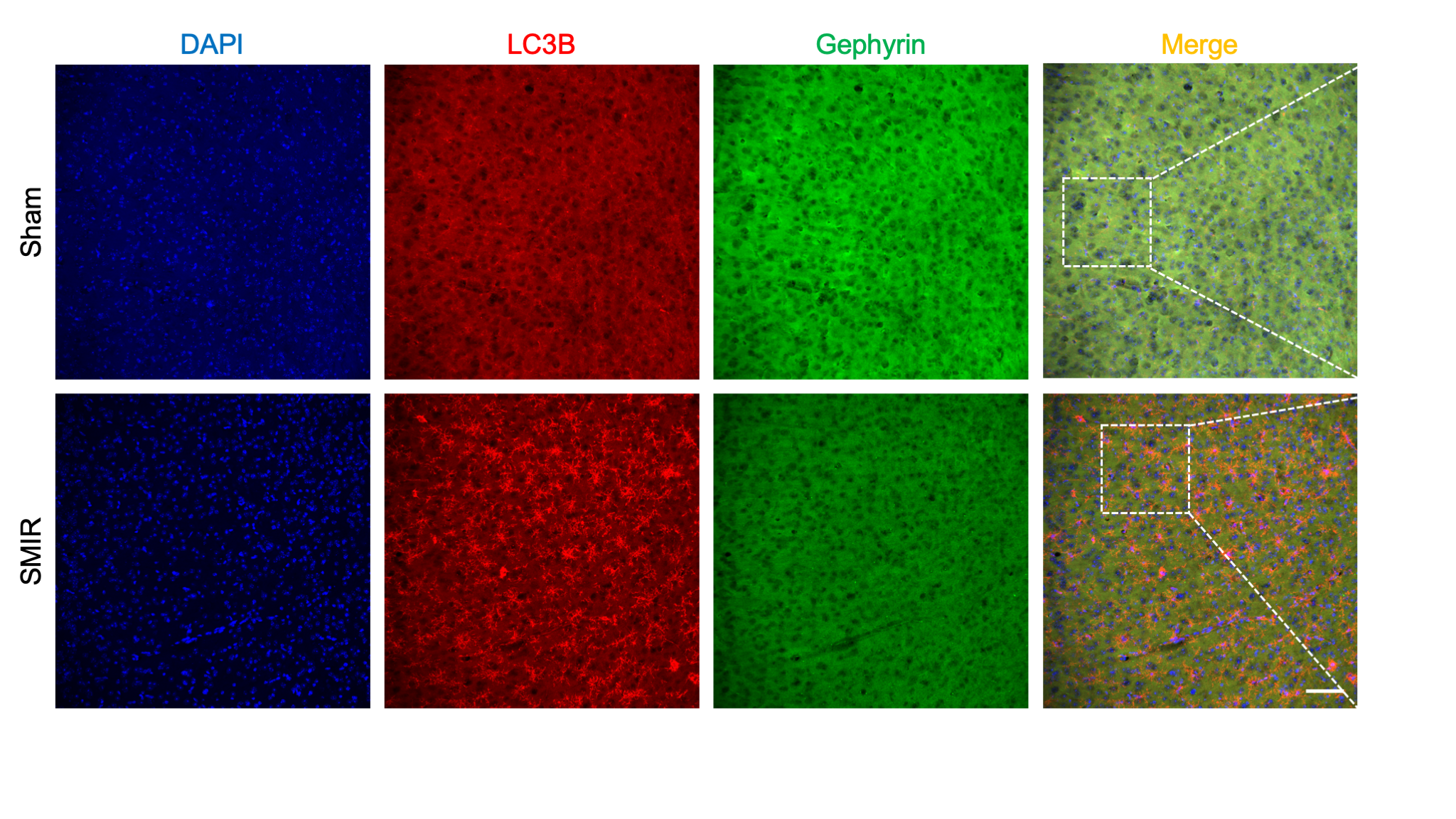

Supplement: Supplementary file 12 — Appendix Figure S2 Source Data [file 44319_2025_646_MOESM12_ESM.zip › Appendix Figure S2/S2G/S2G.tiff]

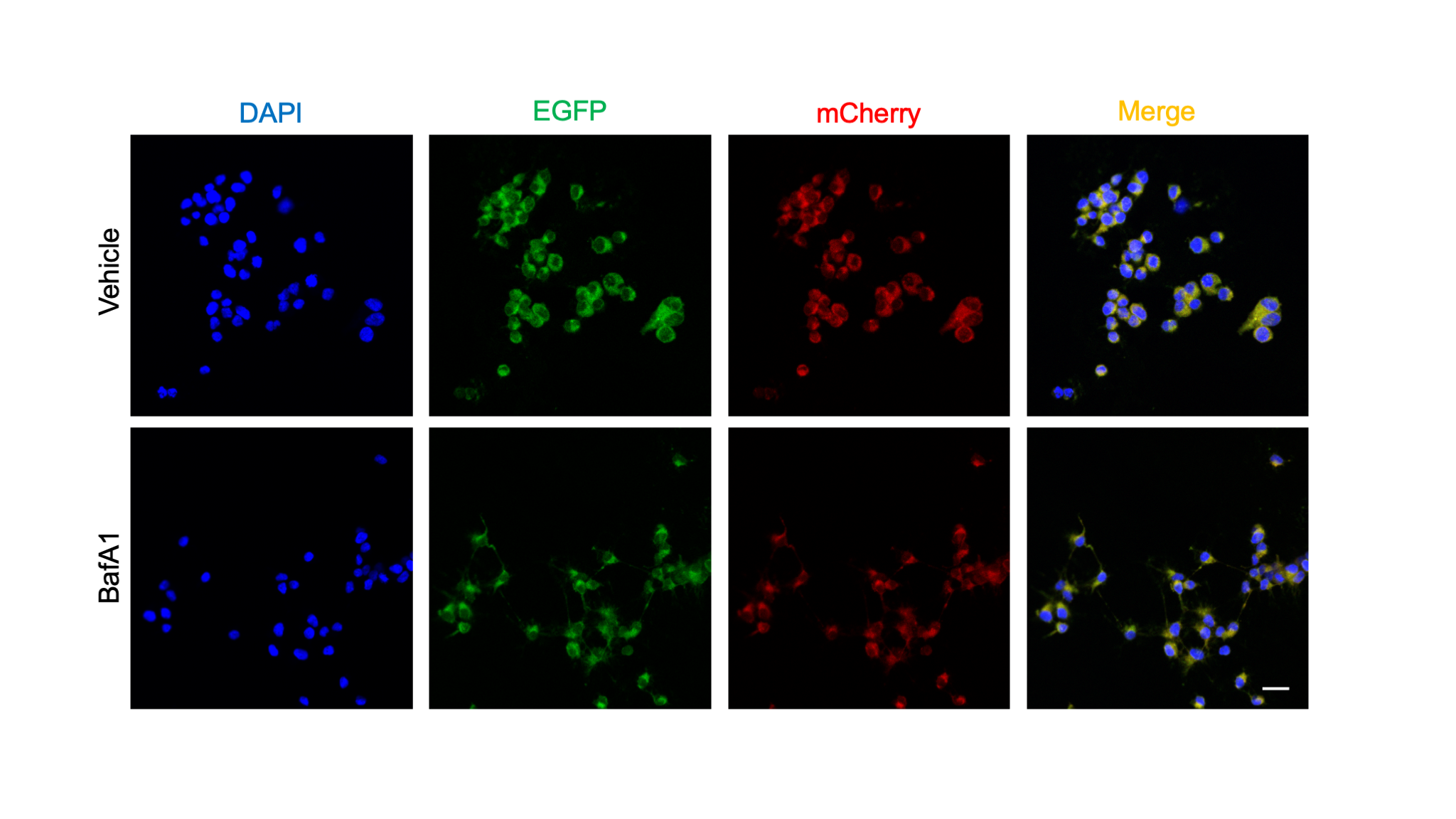

Supplement: Supplementary file 13 — Appendix Figure S3 Source Data [file 44319_2025_646_MOESM13_ESM.zip › Appendix Figure S3/S3A/S3A.tiff]

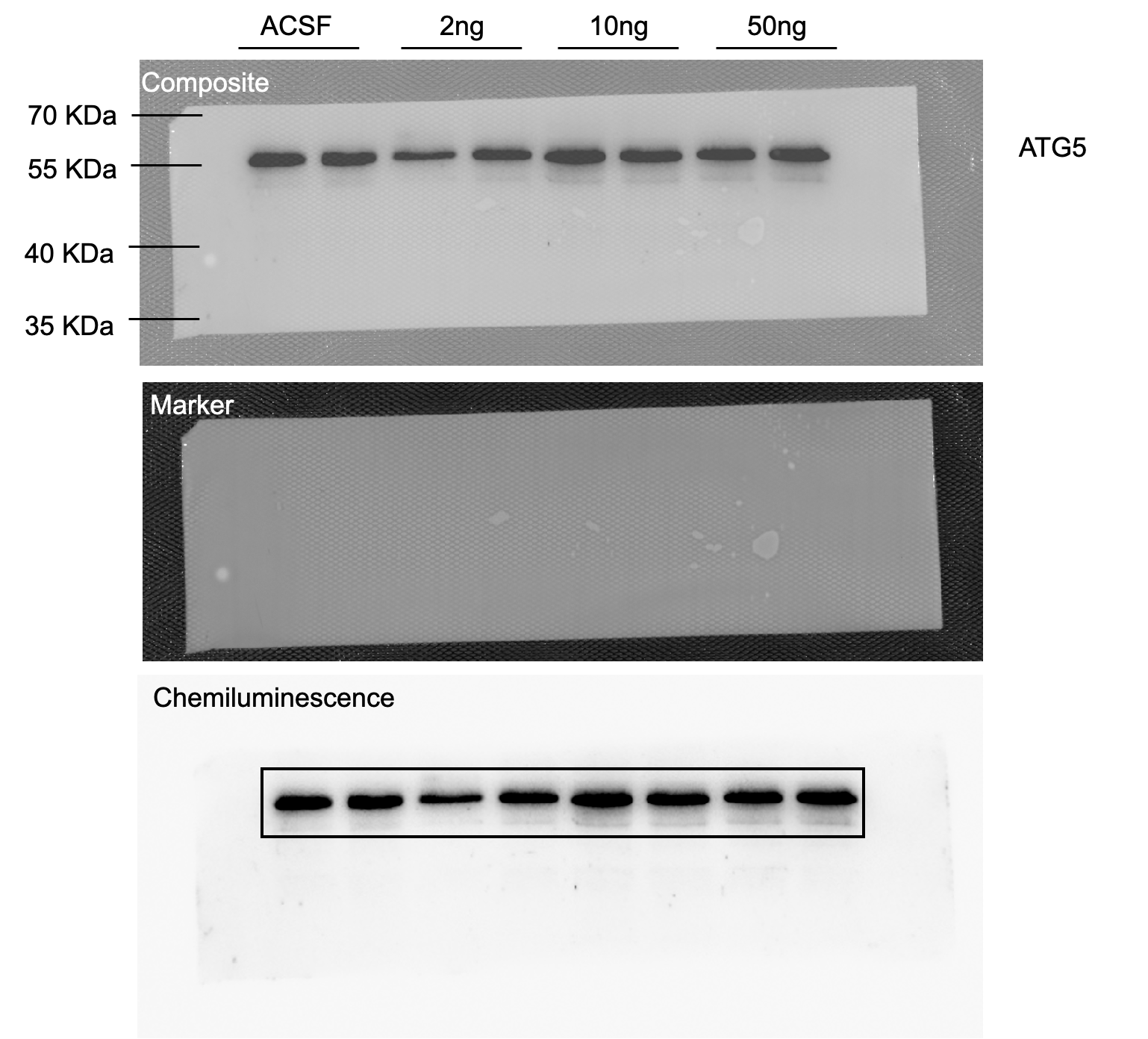

Supplement: Supplementary file 13 — Appendix Figure S3 Source Data [file 44319_2025_646_MOESM13_ESM.zip › Appendix Figure S3/S3G/S3G-ATG5.png]

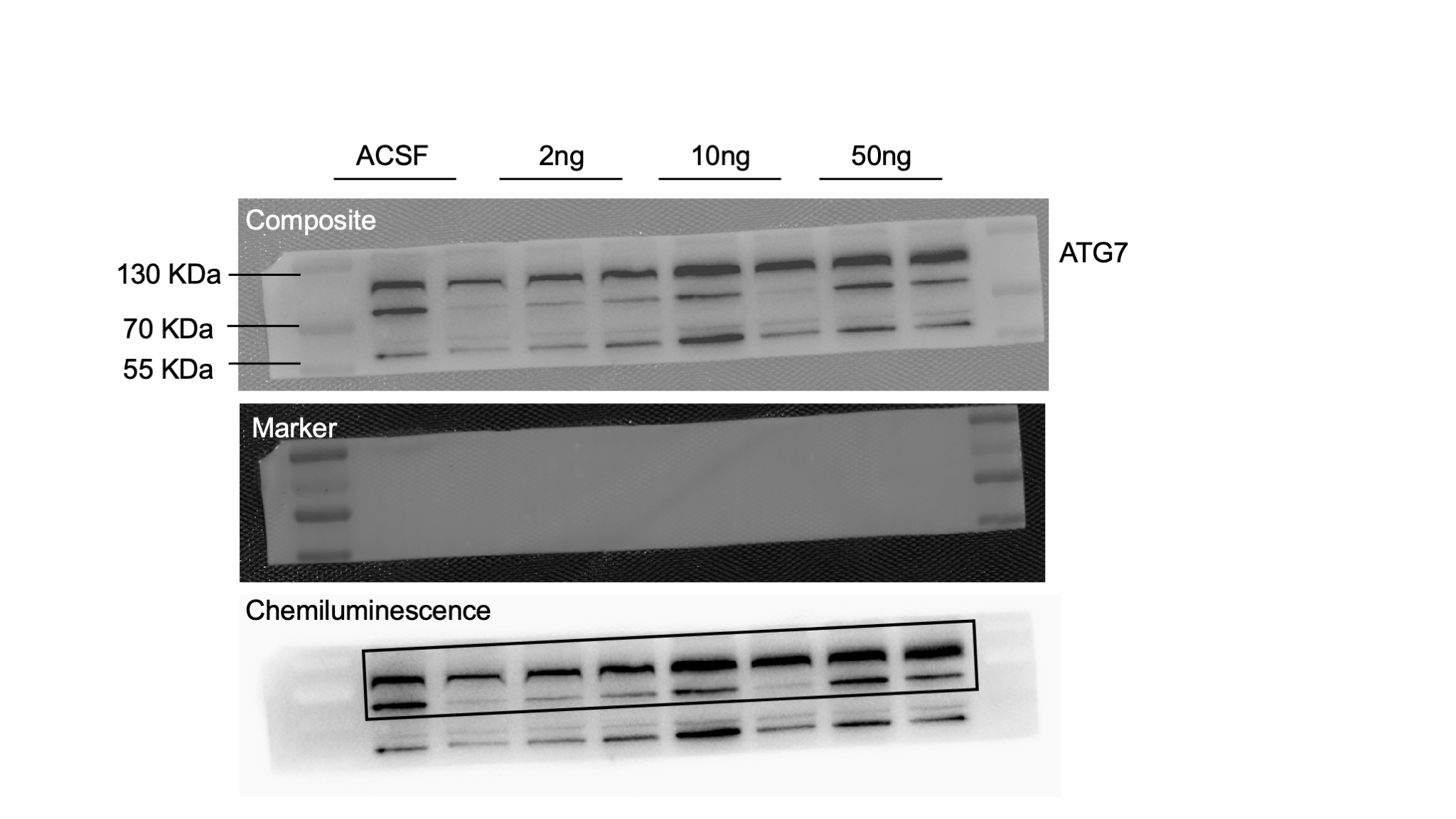

Supplement: Supplementary file 13 — Appendix Figure S3 Source Data [file 44319_2025_646_MOESM13_ESM.zip › Appendix Figure S3/S3G/S3G-ATG7.tiff]

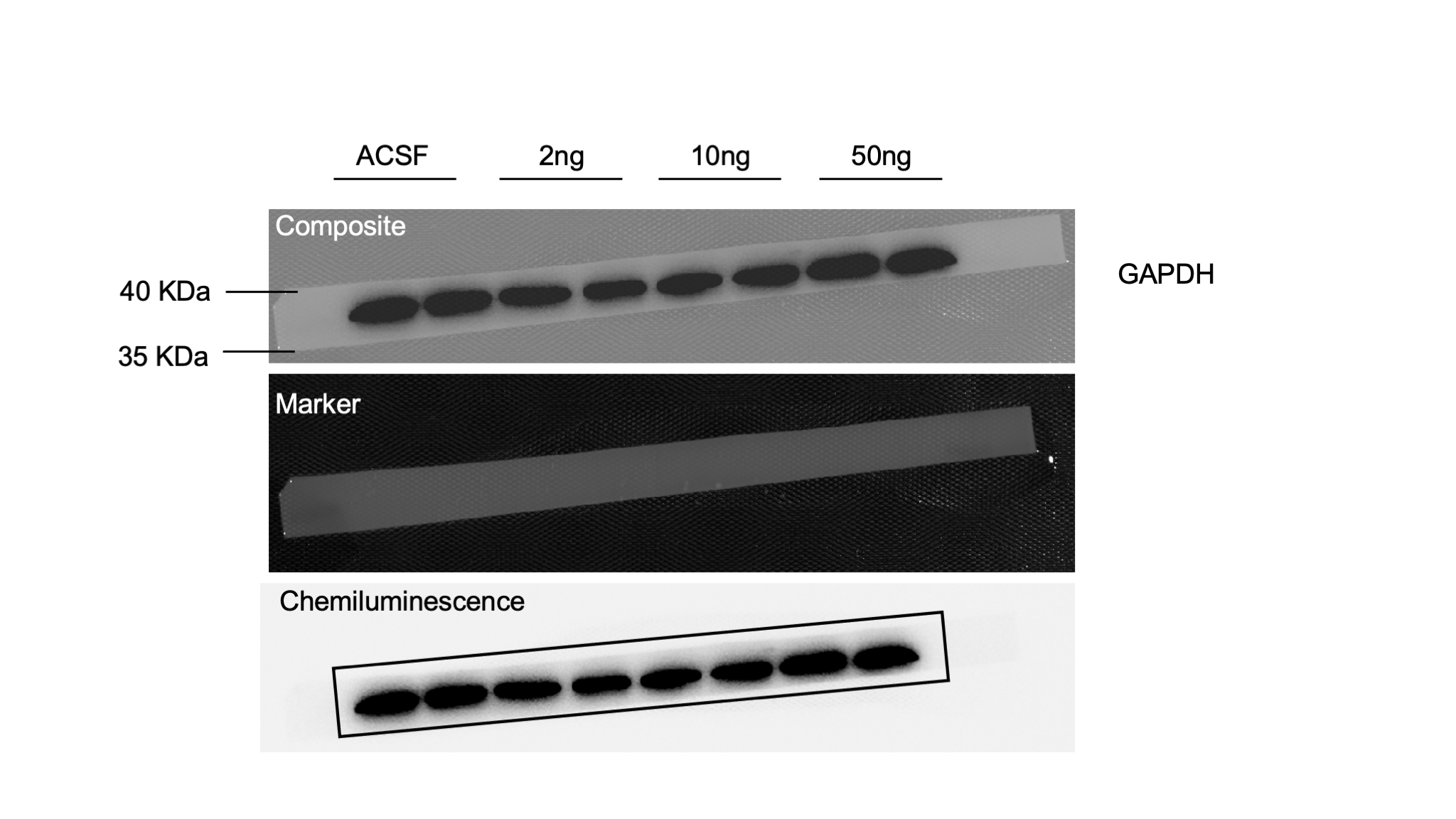

Supplement: Supplementary file 13 — Appendix Figure S3 Source Data [file 44319_2025_646_MOESM13_ESM.zip › Appendix Figure S3/S3G/S3G-GAPDH.tiff]

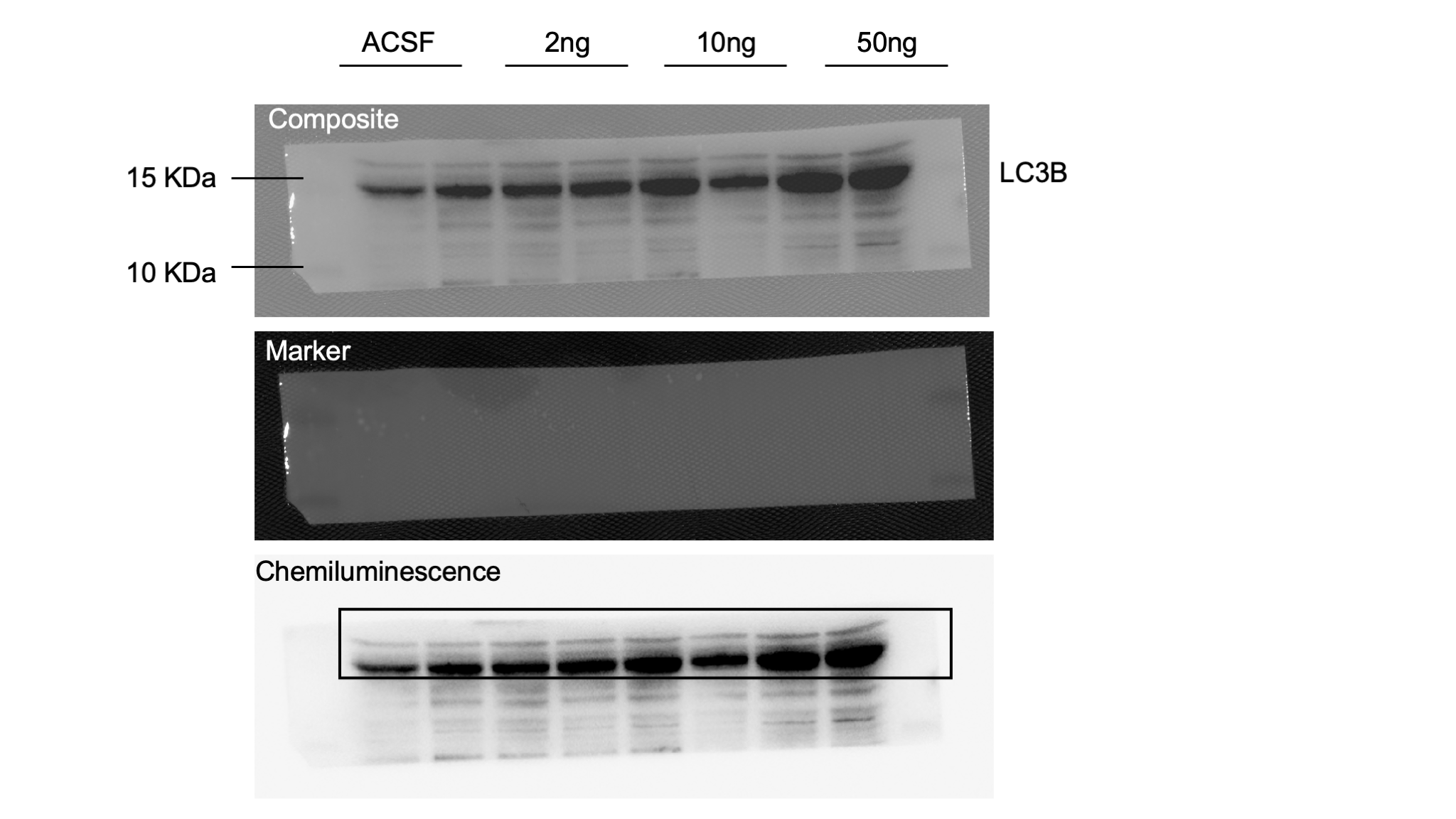

Supplement: Supplementary file 13 — Appendix Figure S3 Source Data [file 44319_2025_646_MOESM13_ESM.zip › Appendix Figure S3/S3G/S3G-LC3B.tiff]
