# Supplementary figures and images for: TMEM251 loss-induced autophagy dysfunction in the anterior cingulate cortex contributes to chronic postoperative pain (part 2 of 2)
Source: EMBO Rep. 2025 Dec 3;27(1):186–207. doi: 10.1038/s44319-025-00646-8 (PMC12796400; doi:10.1038/s44319-025-00646-8)

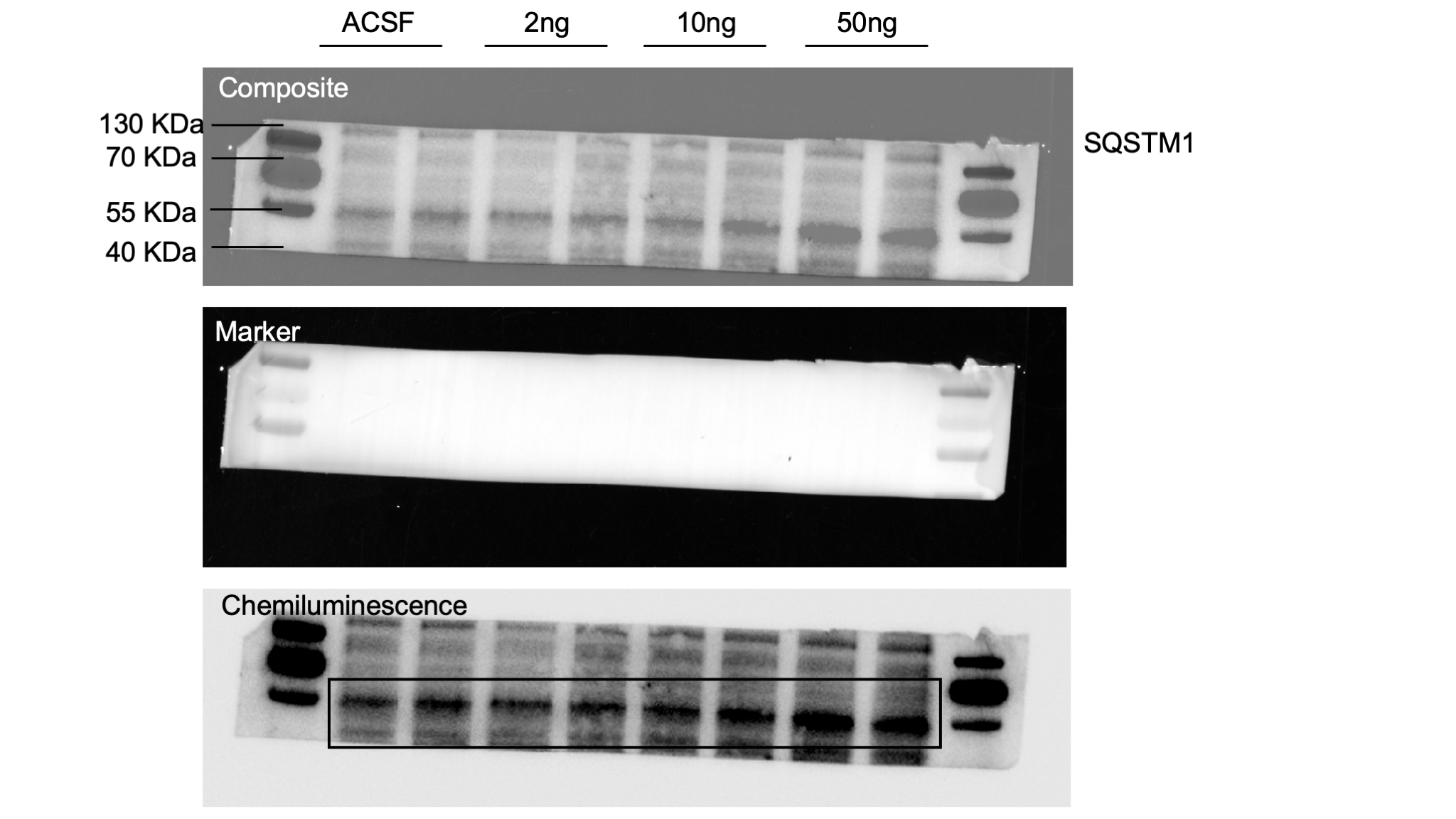

Supplement: Supplementary file 13 — Appendix Figure S3 Source Data [file 44319_2025_646_MOESM13_ESM.zip › Appendix Figure S3/S3G/S3G-SQSTM1.tiff]

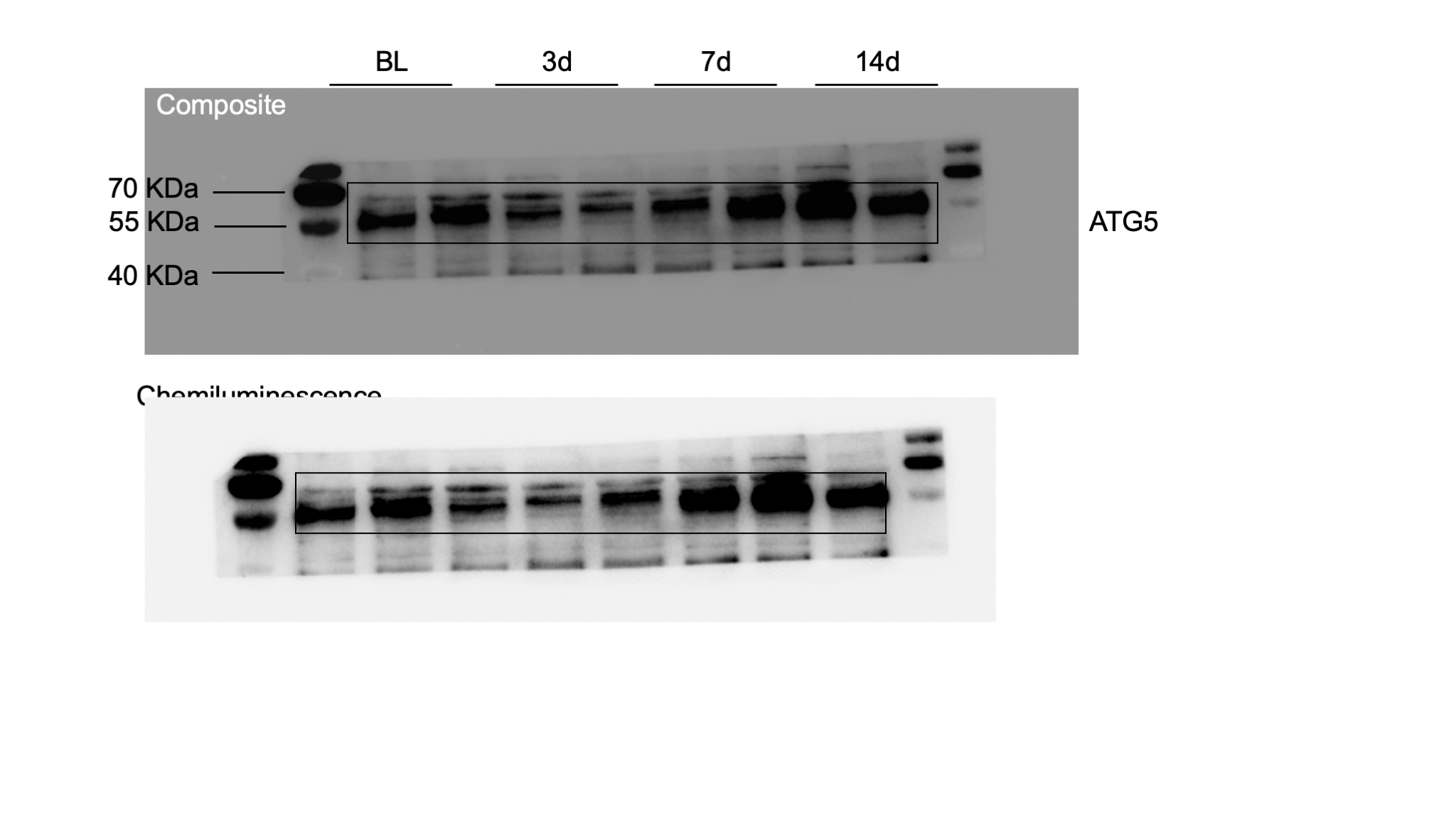

Supplement: Supplementary file 13 — Appendix Figure S3 Source Data [file 44319_2025_646_MOESM13_ESM.zip › Appendix Figure S3/S3H/S3H-ATG5.tiff]

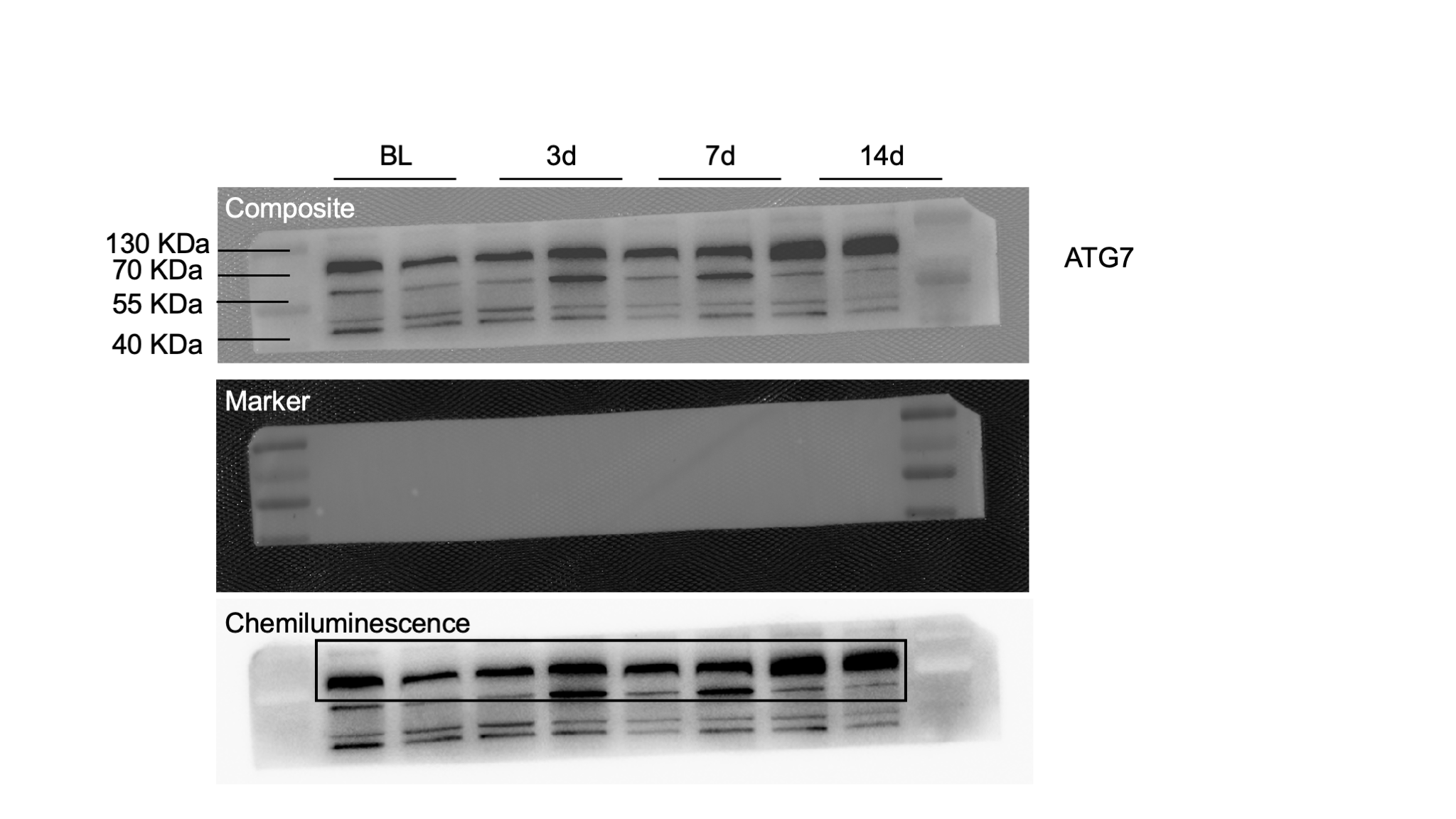

Supplement: Supplementary file 13 — Appendix Figure S3 Source Data [file 44319_2025_646_MOESM13_ESM.zip › Appendix Figure S3/S3H/S3H-ATG7.tiff]

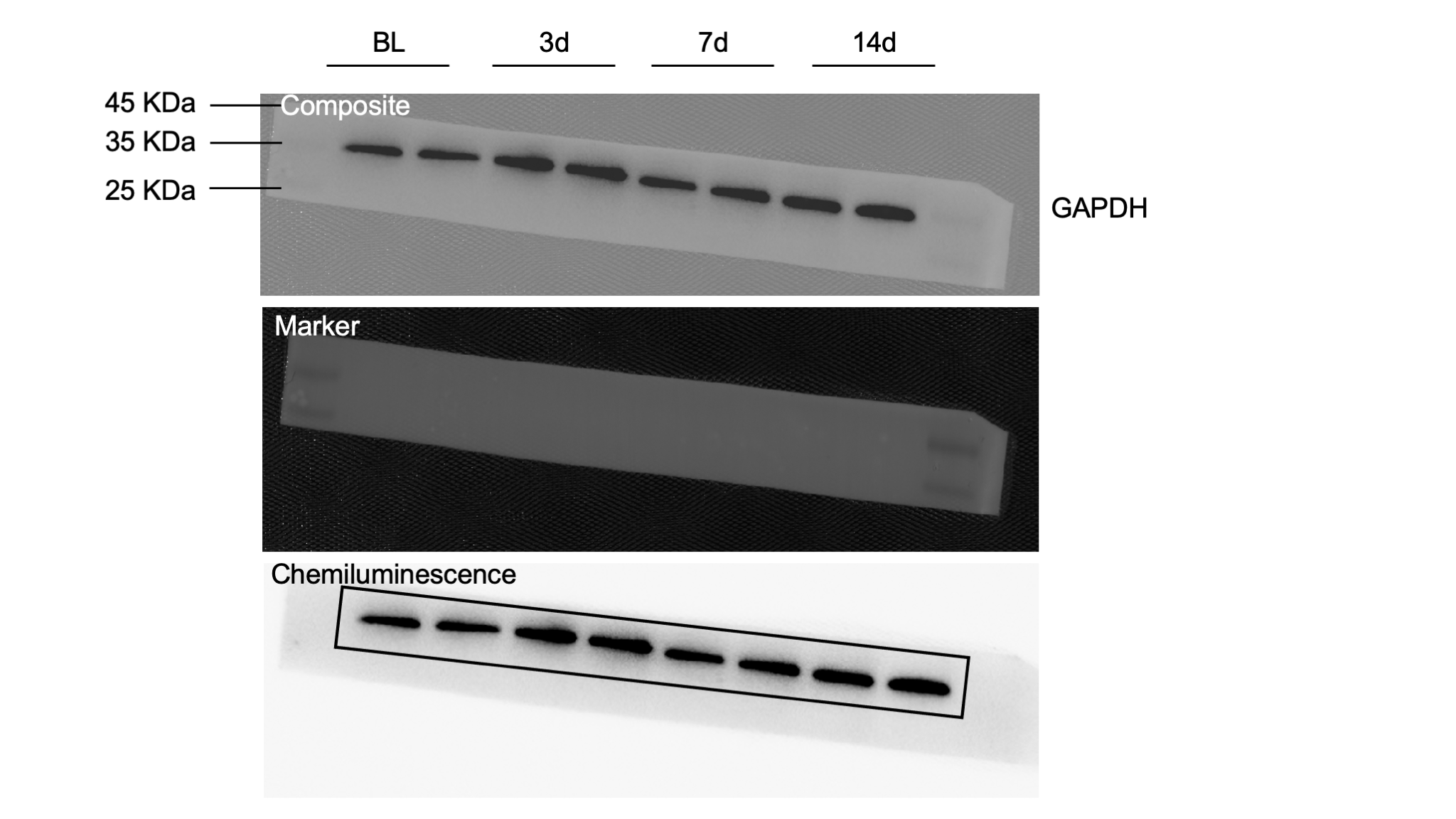

Supplement: Supplementary file 13 — Appendix Figure S3 Source Data [file 44319_2025_646_MOESM13_ESM.zip › Appendix Figure S3/S3H/S3H-GAPDH.tiff]

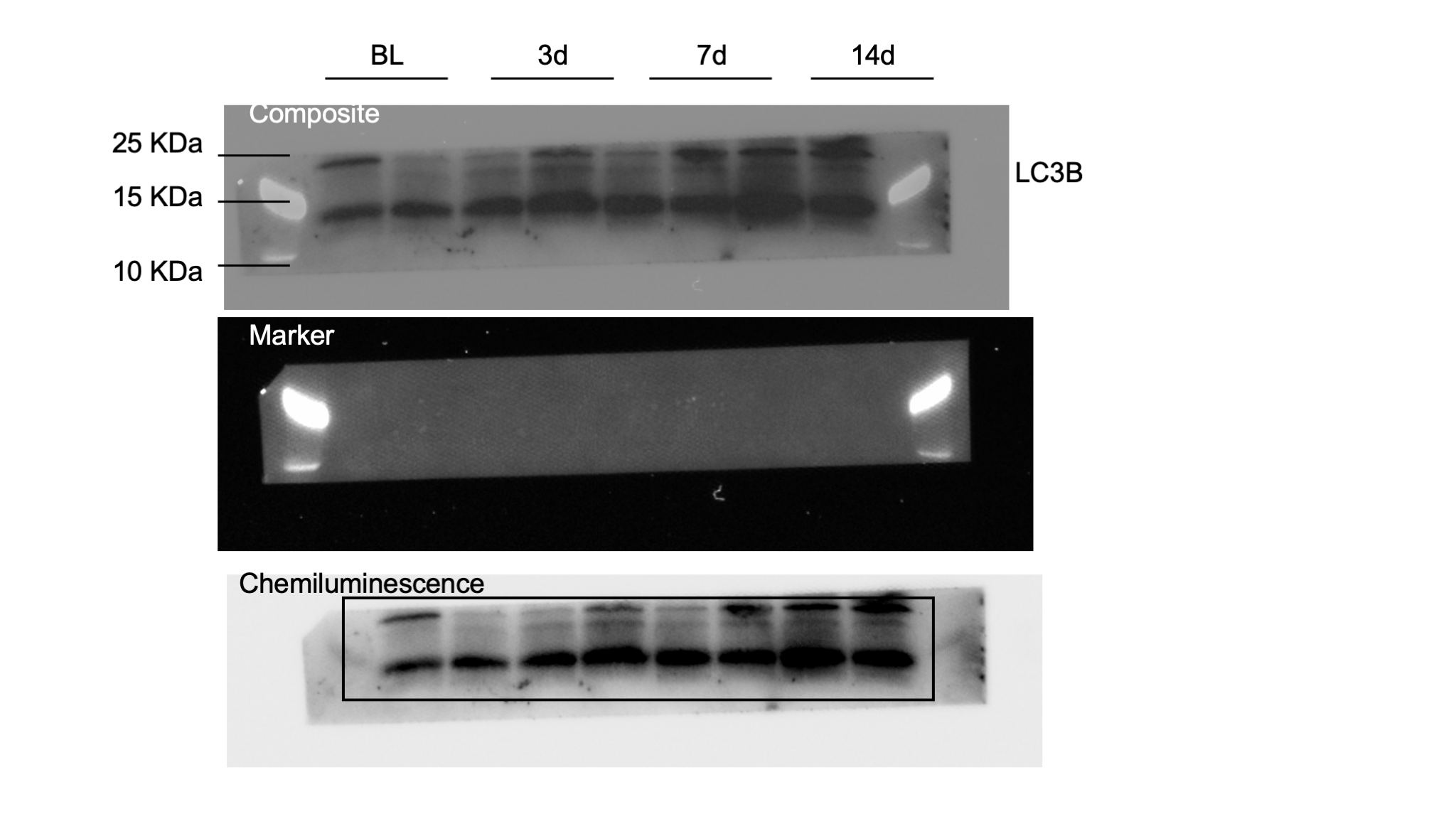

Supplement: Supplementary file 13 — Appendix Figure S3 Source Data [file 44319_2025_646_MOESM13_ESM.zip › Appendix Figure S3/S3H/S3H-LC3B.tiff]

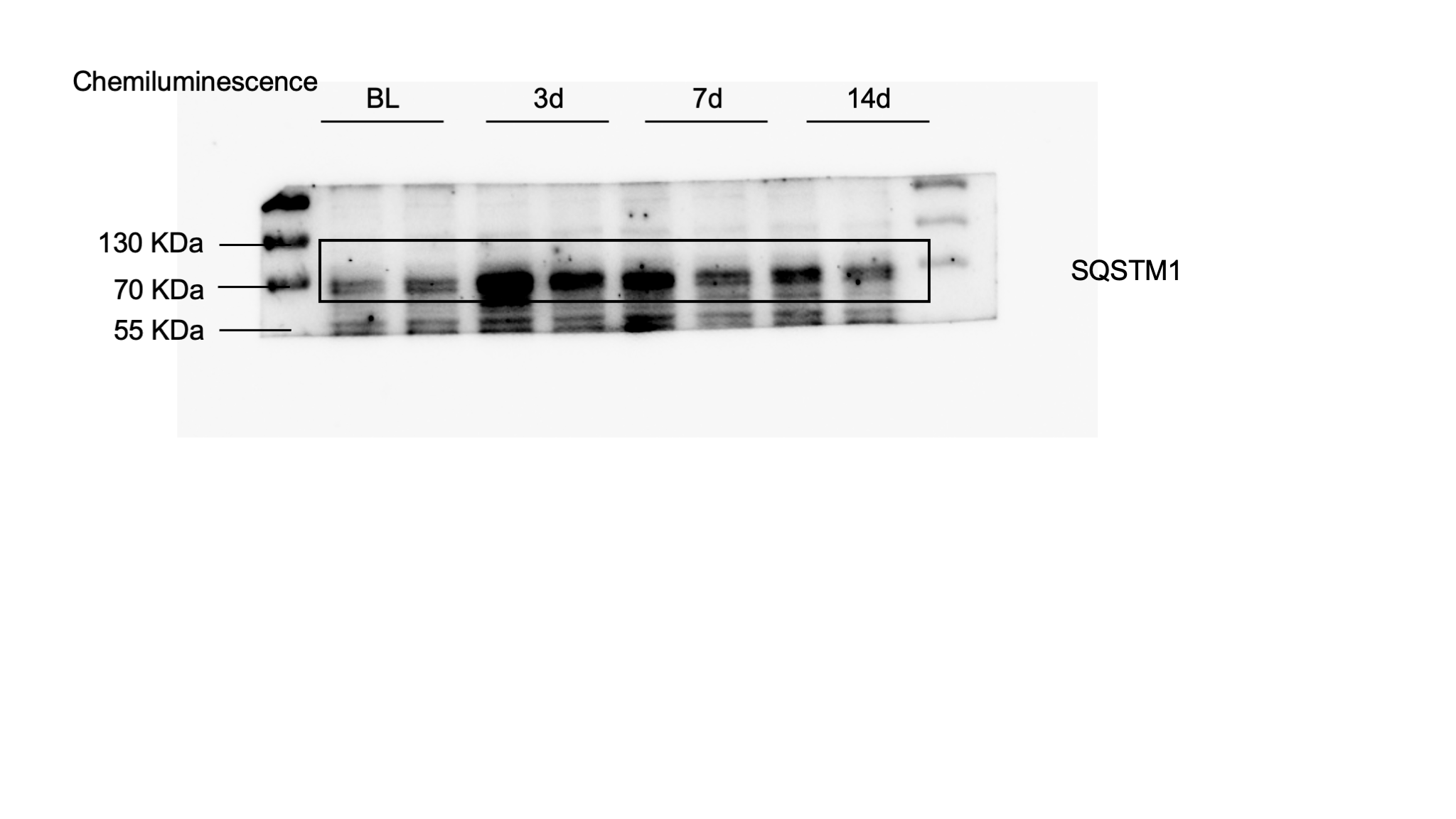

Supplement: Supplementary file 13 — Appendix Figure S3 Source Data [file 44319_2025_646_MOESM13_ESM.zip › Appendix Figure S3/S3H/S3H-SQSTM1.tiff]

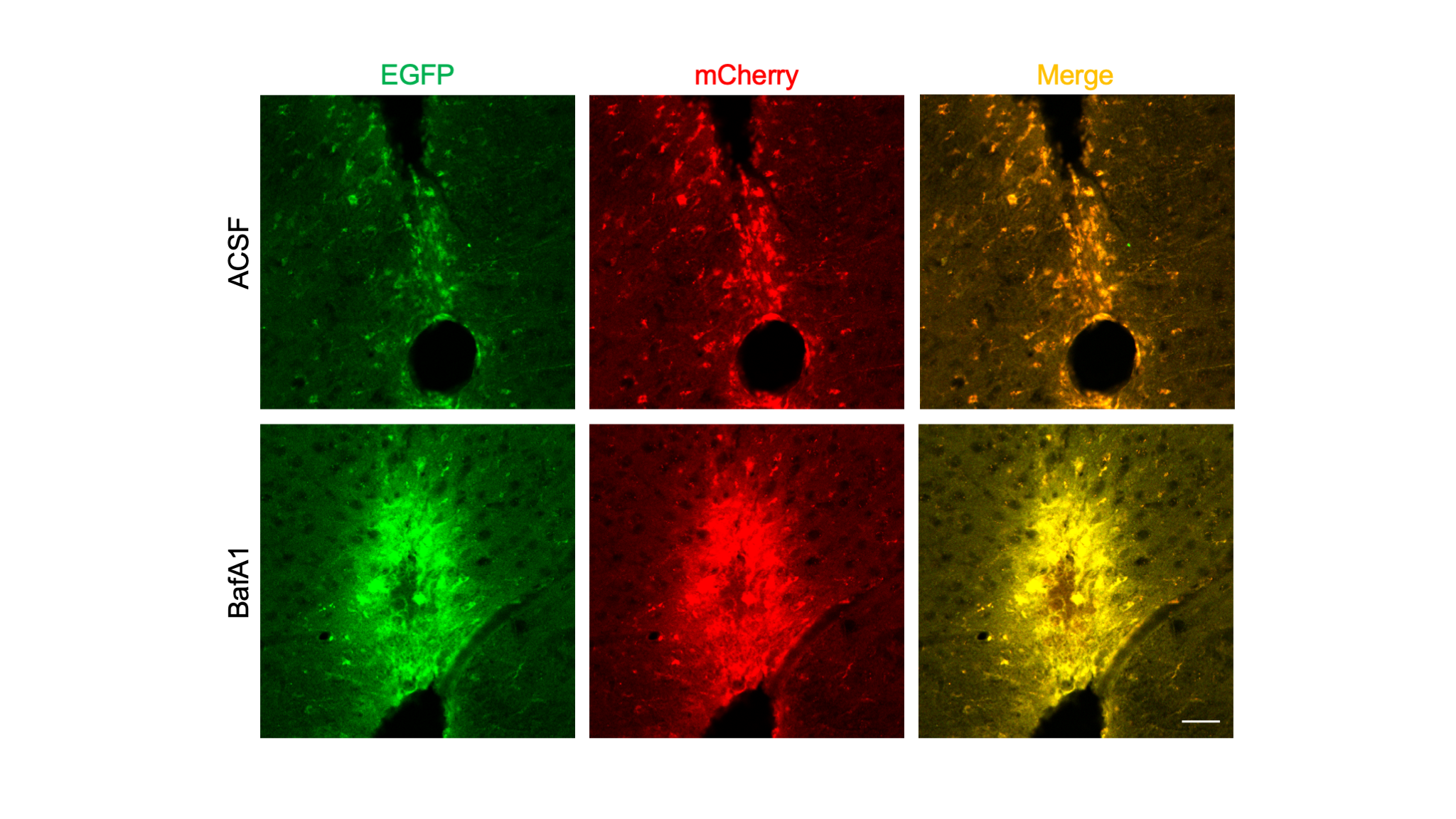

Supplement: Supplementary file 13 — Appendix Figure S3 Source Data [file 44319_2025_646_MOESM13_ESM.zip › Appendix Figure S3/S3K/S3K.tiff]

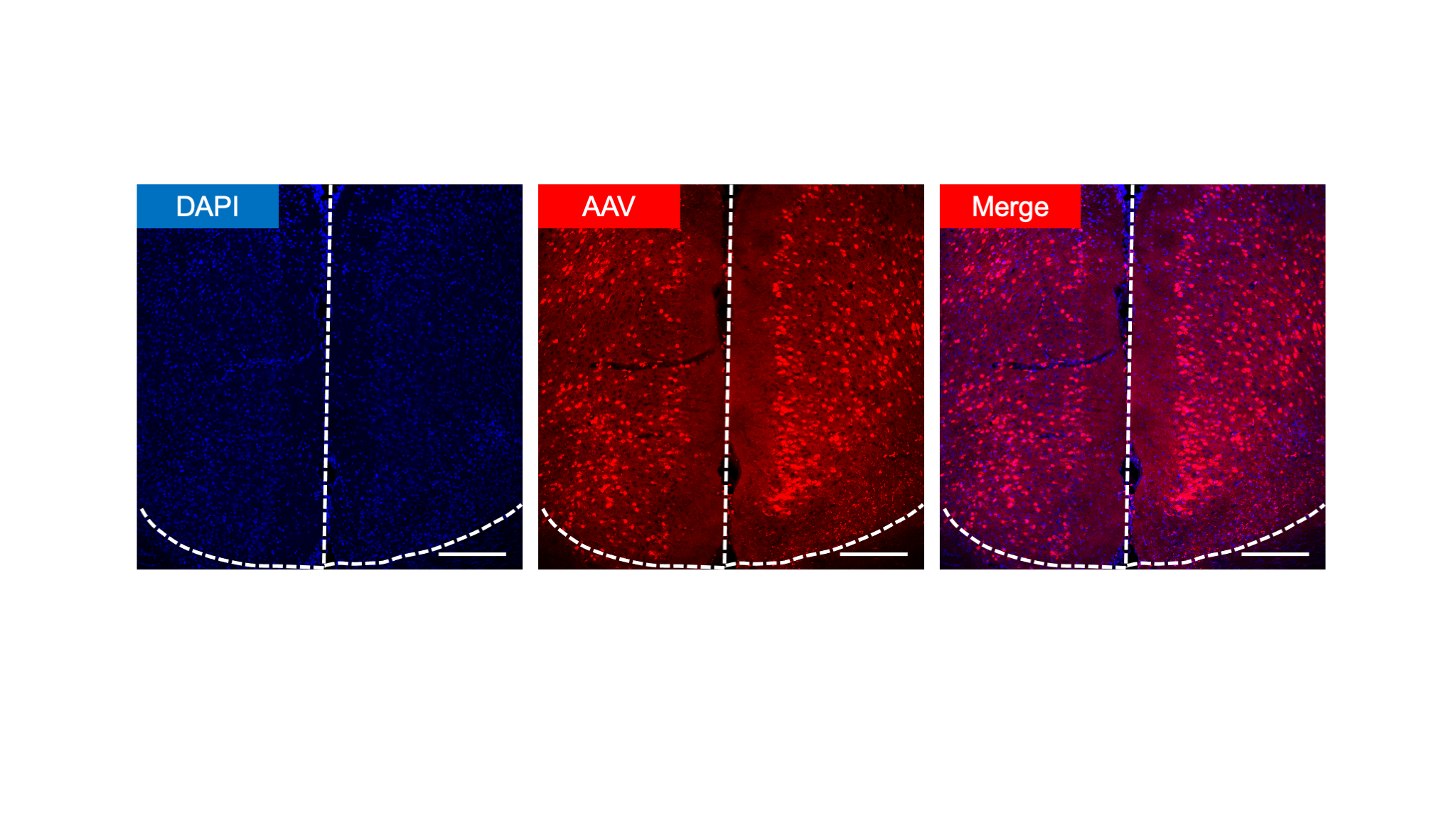

Supplement: Supplementary file 14 — Appendix Figure S4 Source Data [file 44319_2025_646_MOESM14_ESM.zip › Appendix Figure S4/S4E/S4E.tiff]

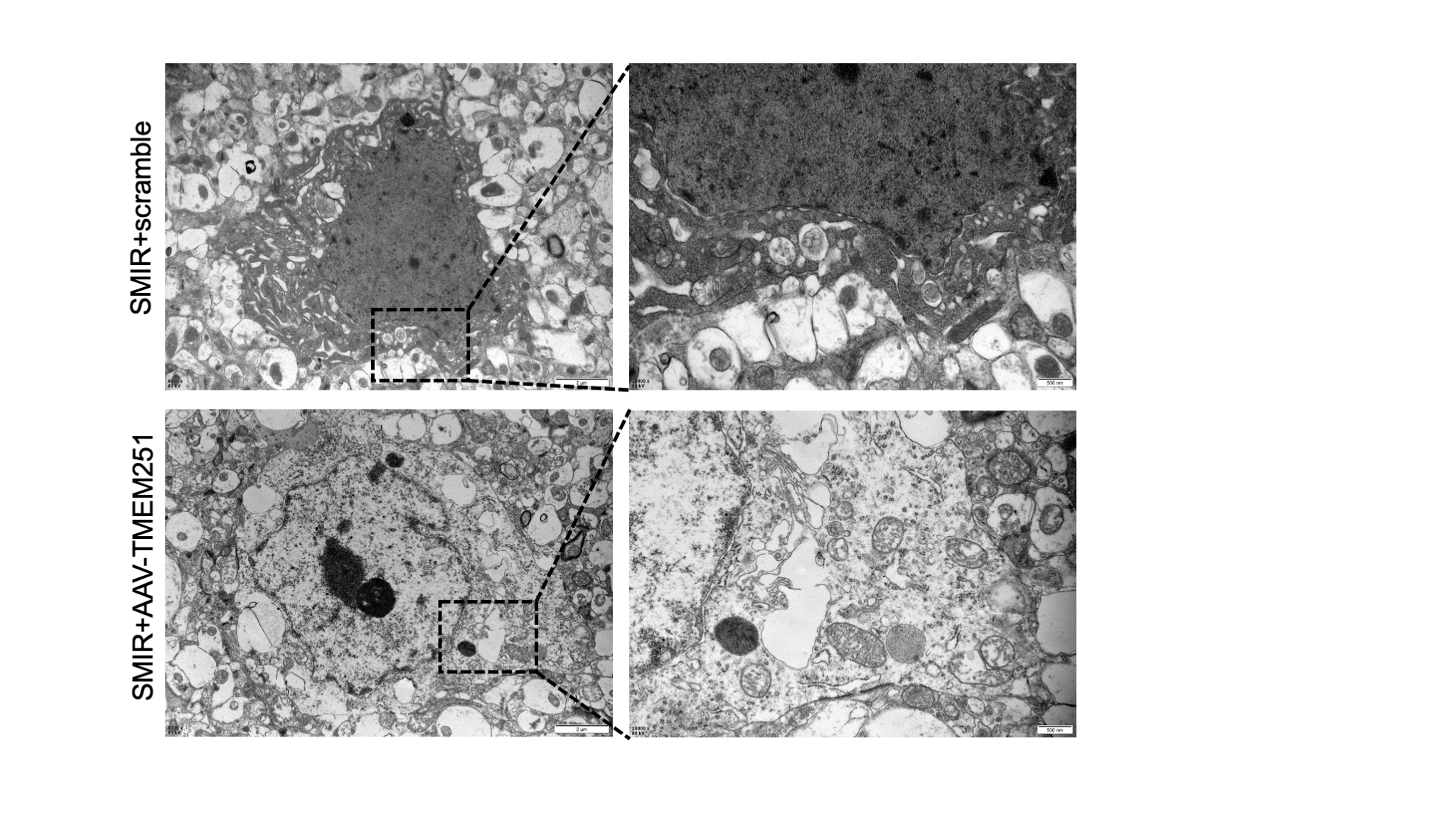

Supplement: Supplementary file 14 — Appendix Figure S4 Source Data [file 44319_2025_646_MOESM14_ESM.zip › Appendix Figure S4/S4F/S4F.tiff]

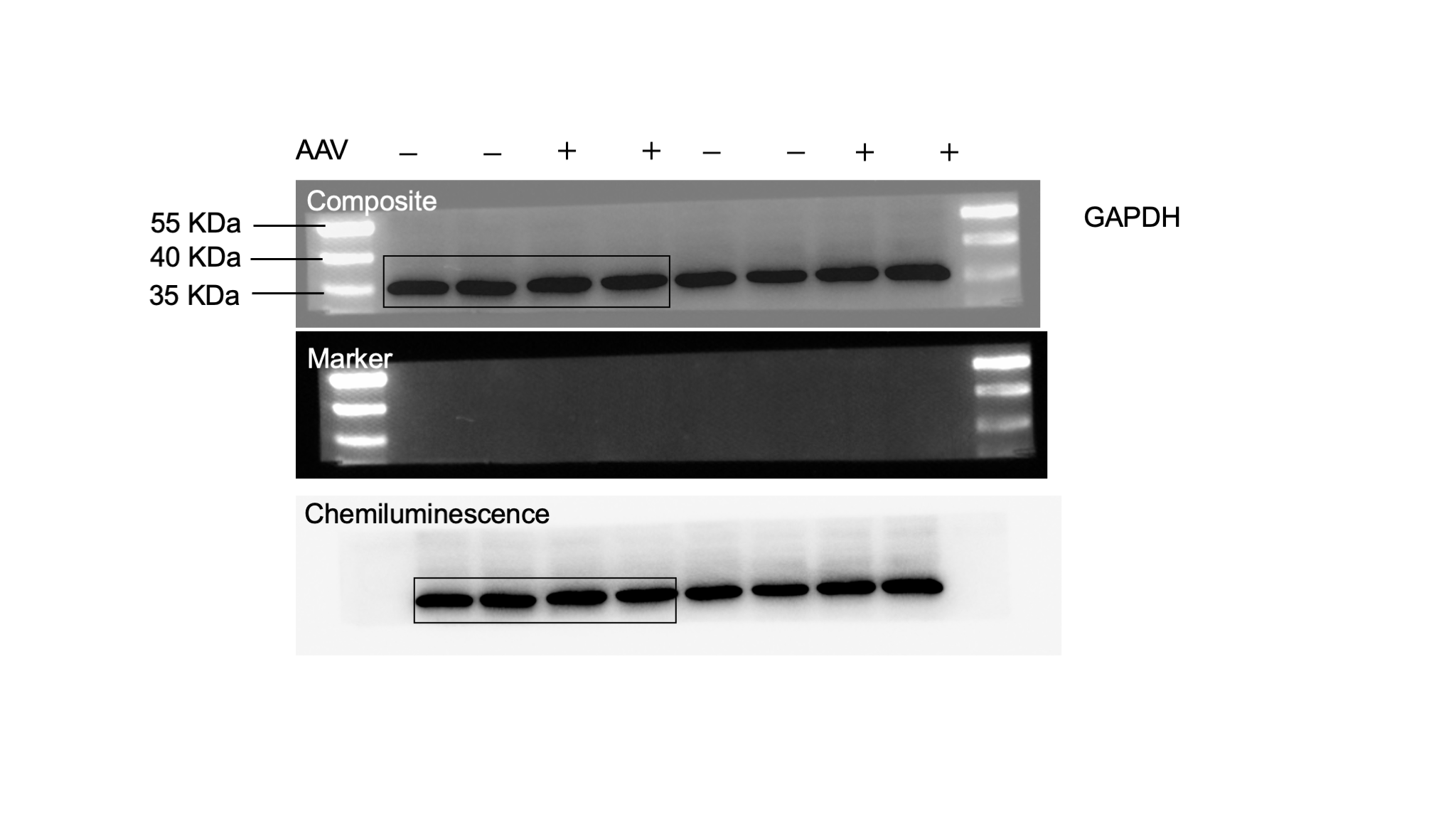

Supplement: Supplementary file 14 — Appendix Figure S4 Source Data [file 44319_2025_646_MOESM14_ESM.zip › Appendix Figure S4/S4G/S4G-GAPDH.tiff]

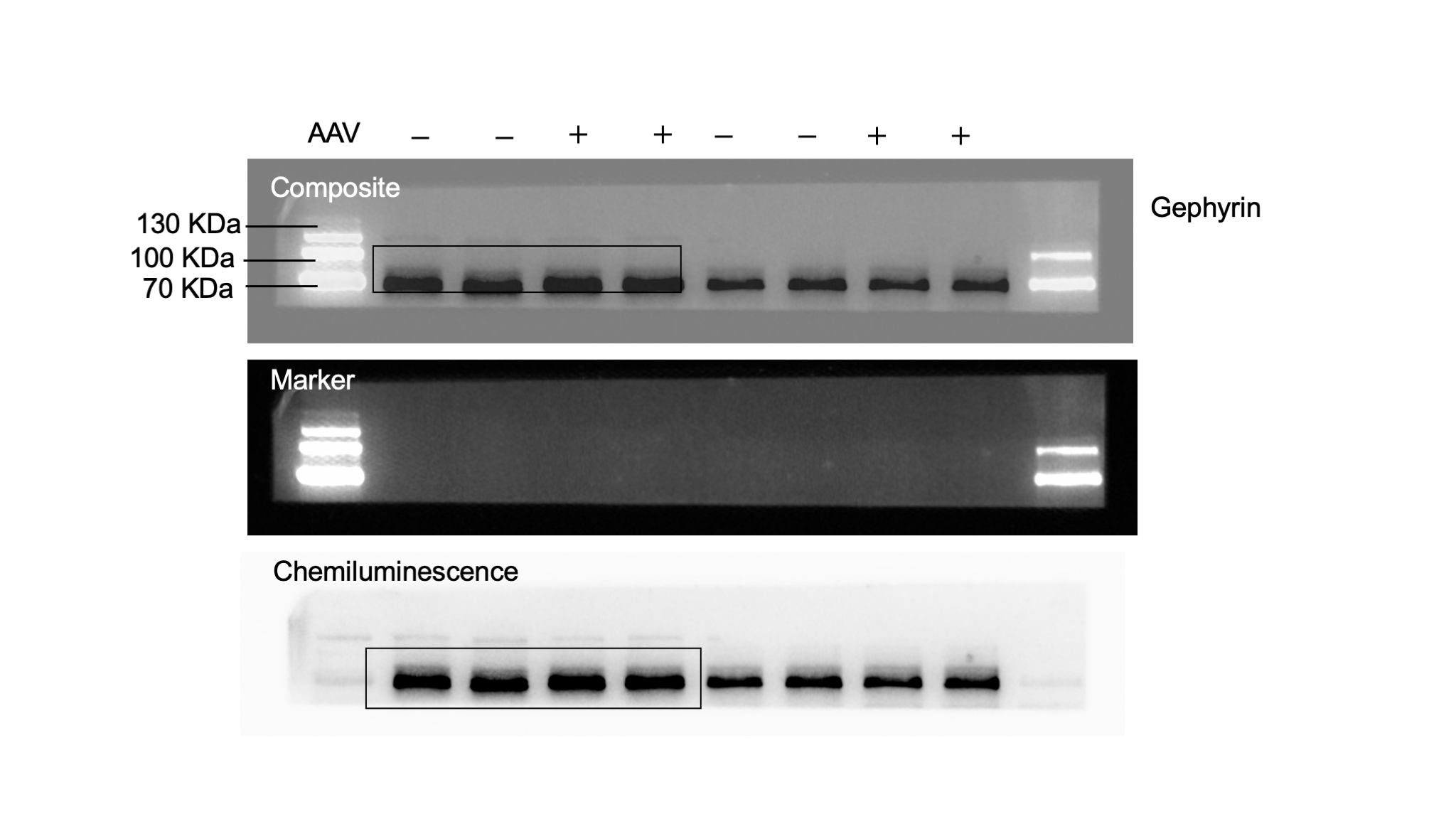

Supplement: Supplementary file 14 — Appendix Figure S4 Source Data [file 44319_2025_646_MOESM14_ESM.zip › Appendix Figure S4/S4G/S4G-Gephyrin.tiff]

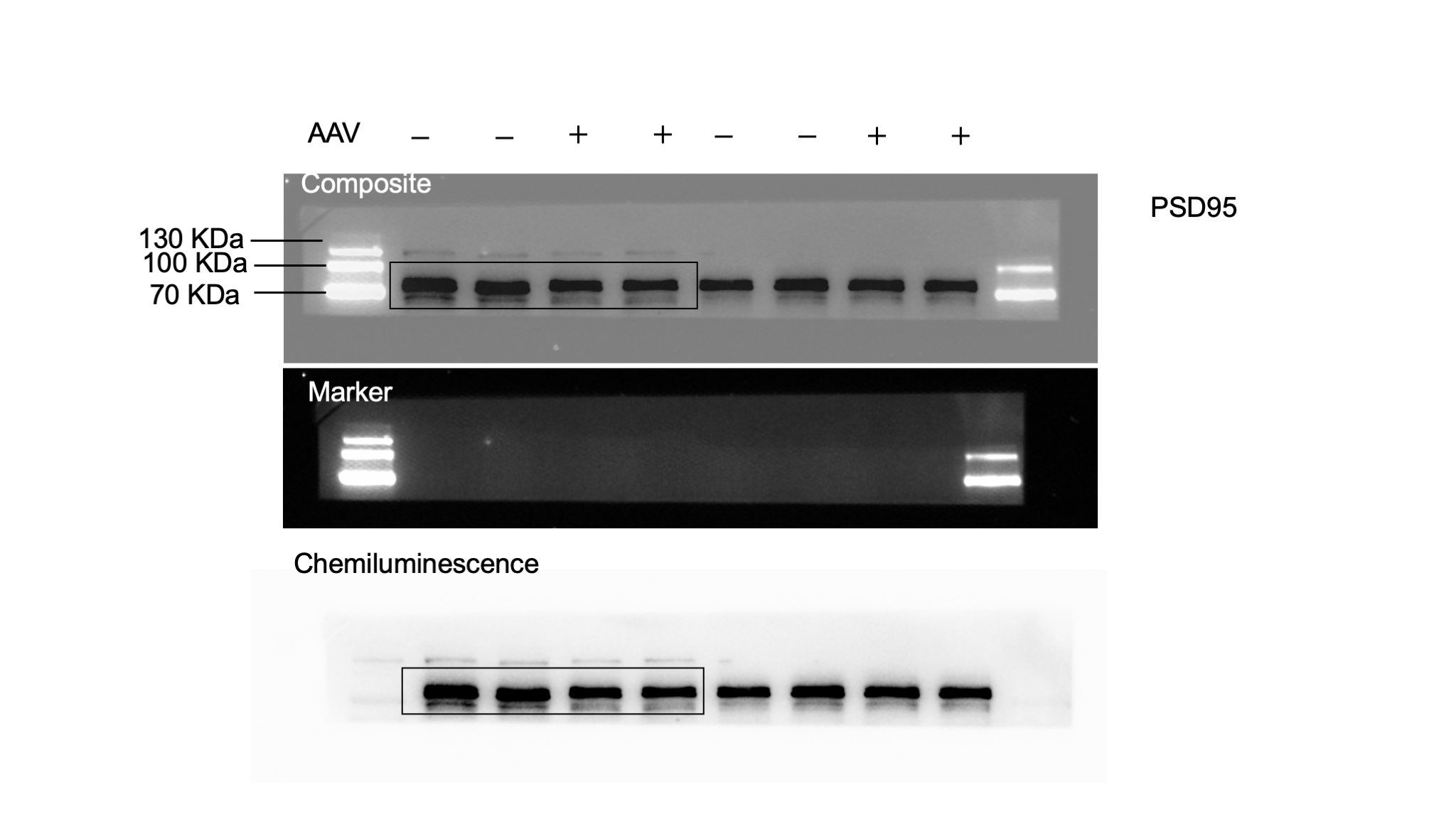

Supplement: Supplementary file 14 — Appendix Figure S4 Source Data [file 44319_2025_646_MOESM14_ESM.zip › Appendix Figure S4/S4G/S4G-PSD95.tiff]

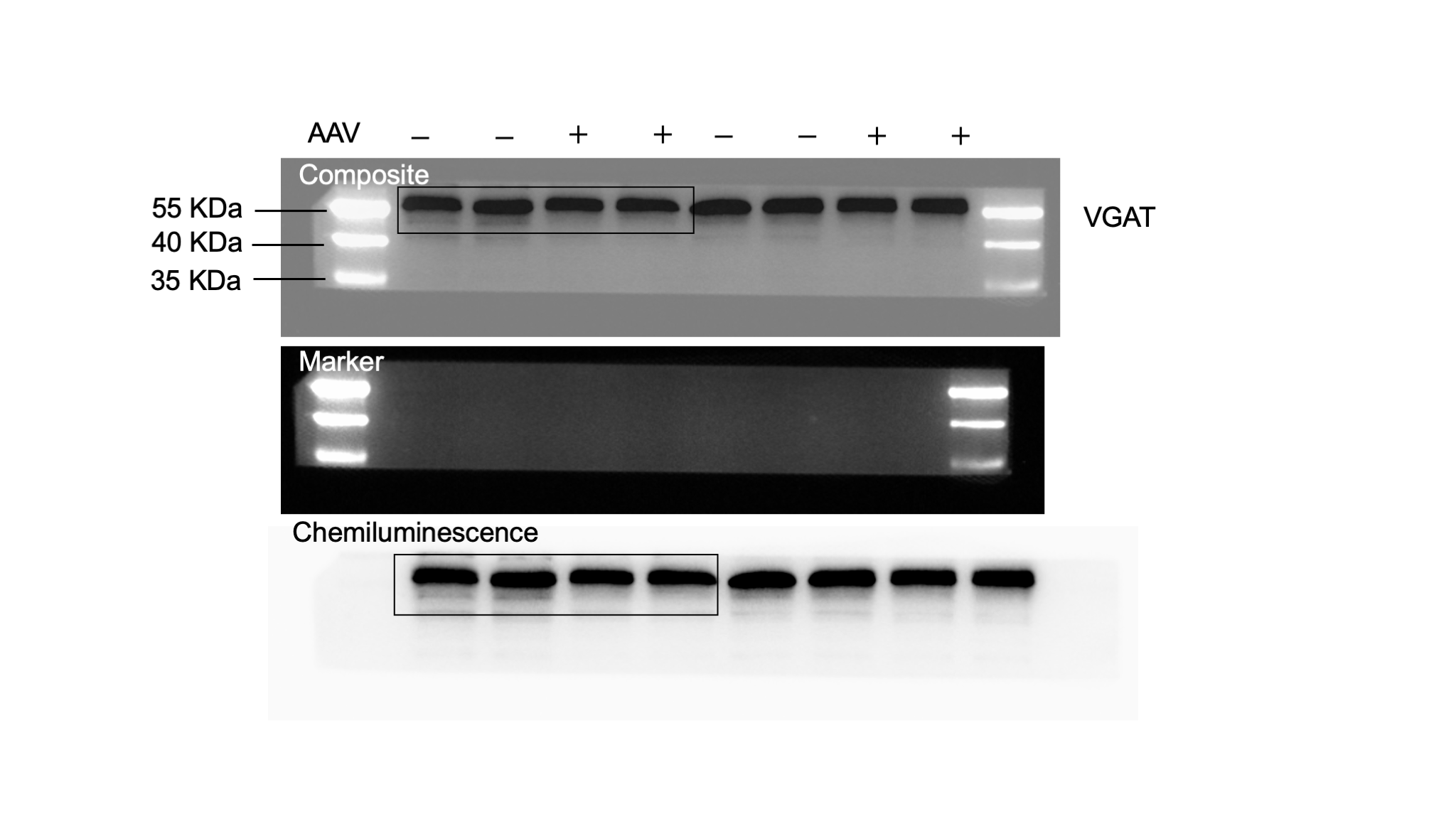

Supplement: Supplementary file 14 — Appendix Figure S4 Source Data [file 44319_2025_646_MOESM14_ESM.zip › Appendix Figure S4/S4G/S4G-VGAT.tiff]

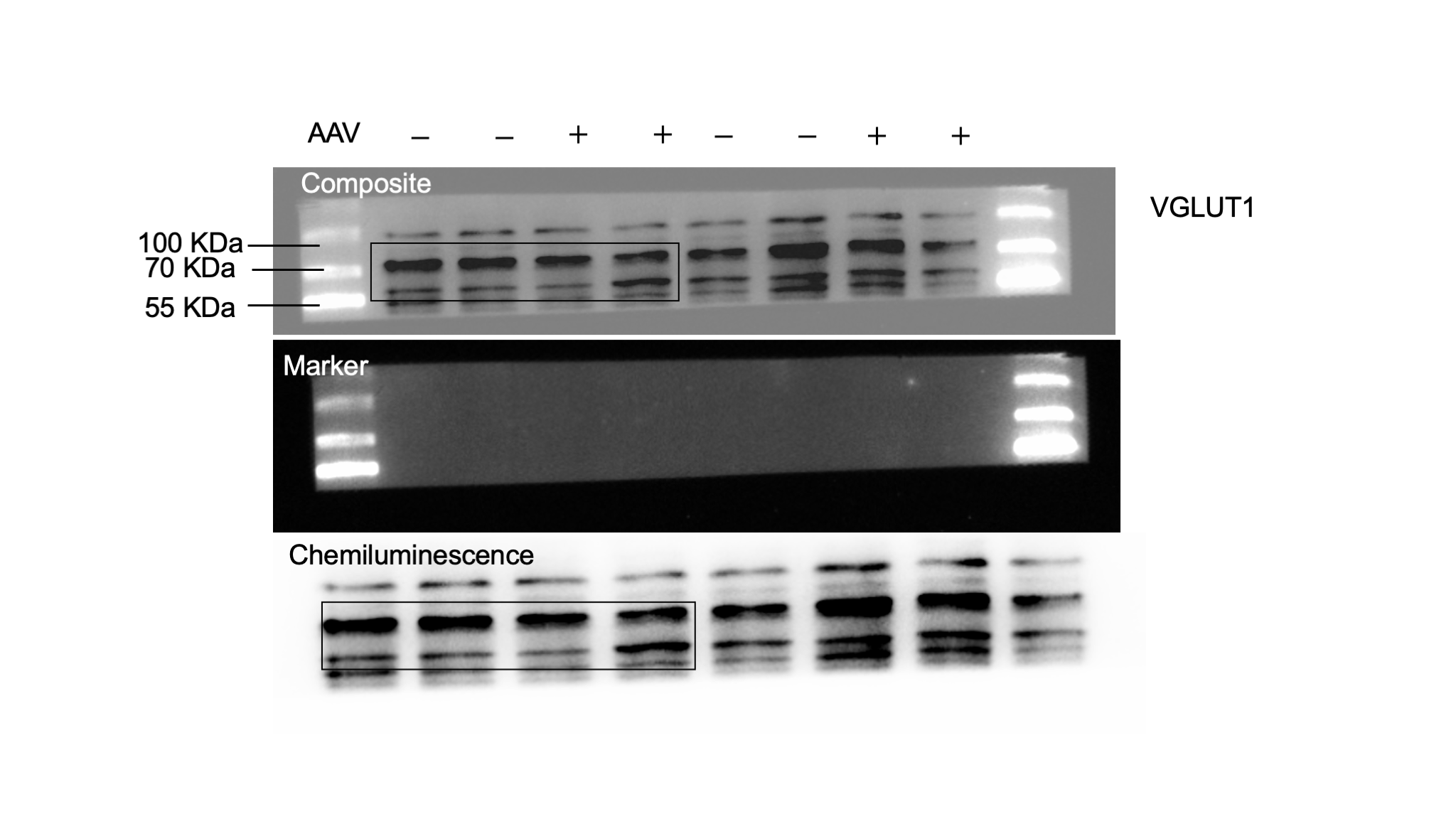

Supplement: Supplementary file 14 — Appendix Figure S4 Source Data [file 44319_2025_646_MOESM14_ESM.zip › Appendix Figure S4/S4G/S4G-VGLUT1.tiff]

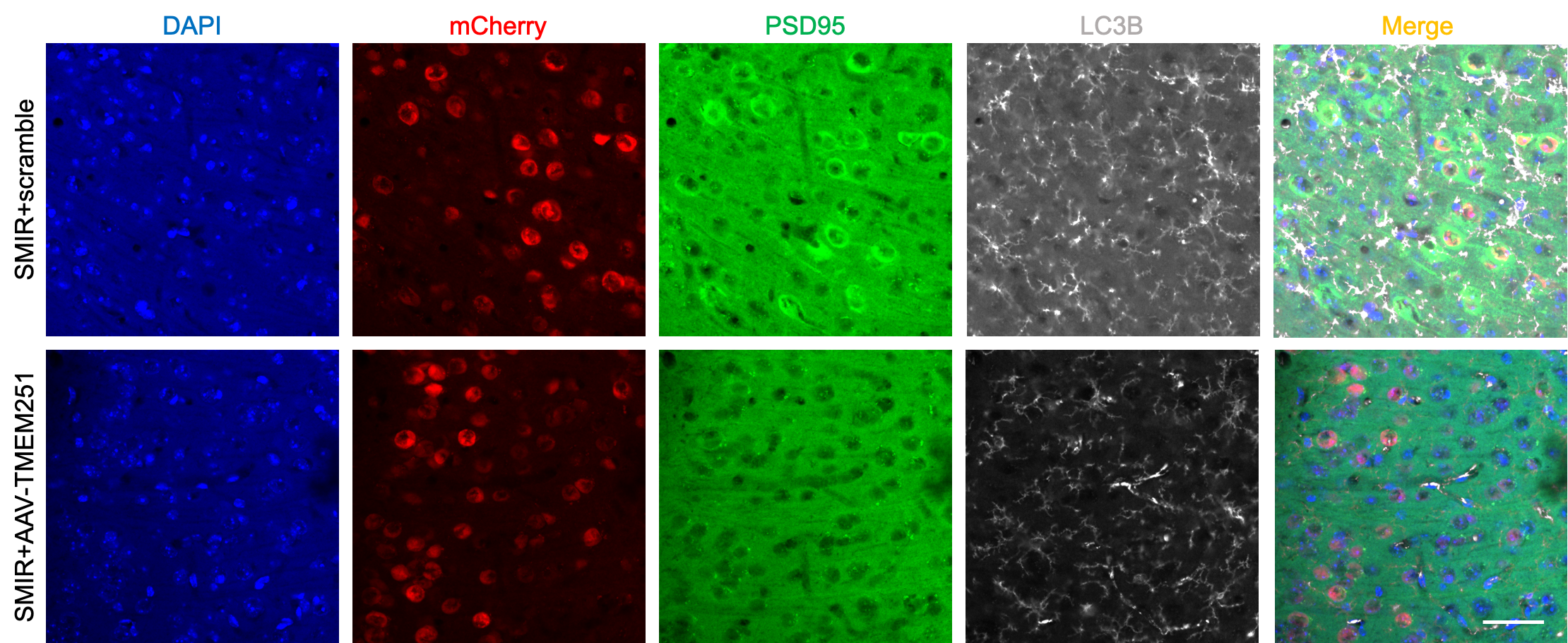

Supplement: Supplementary file 14 — Appendix Figure S4 Source Data [file 44319_2025_646_MOESM14_ESM.zip › Appendix Figure S4/S4H/S4H.png]

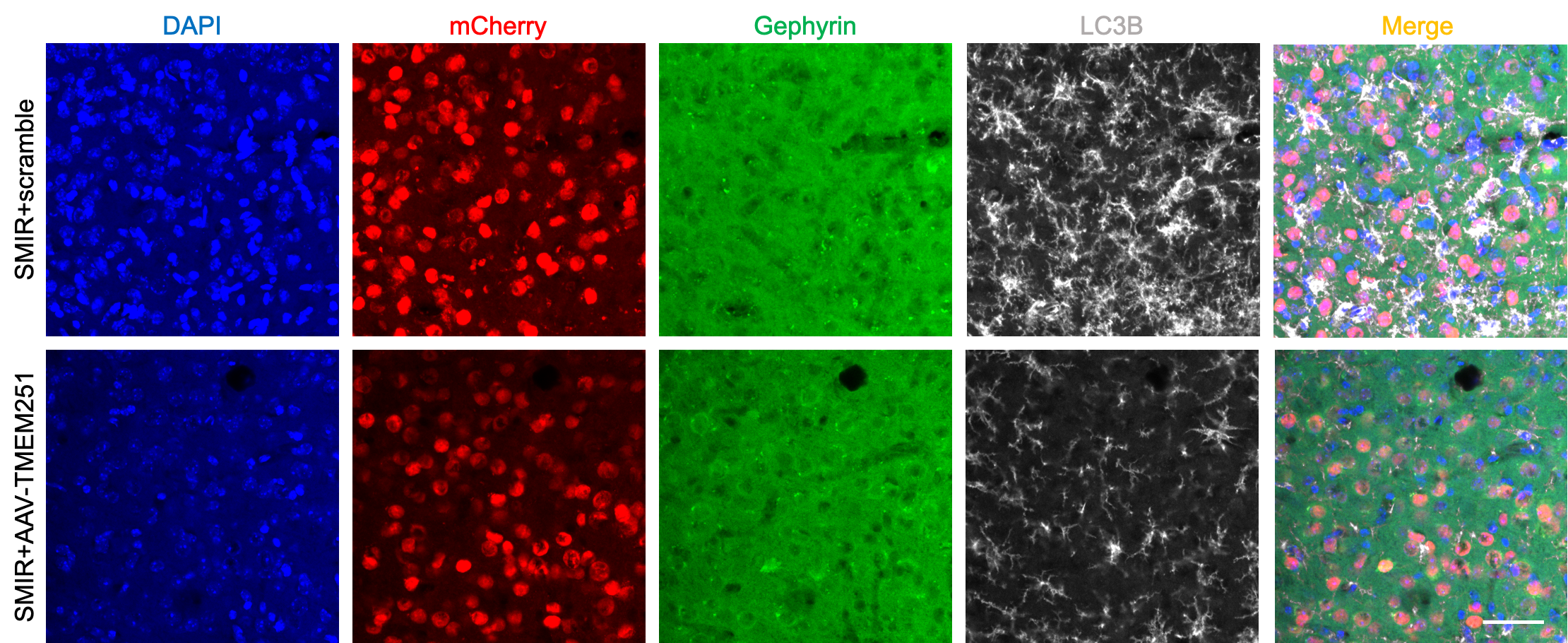

Supplement: Supplementary file 14 — Appendix Figure S4 Source Data [file 44319_2025_646_MOESM14_ESM.zip › Appendix Figure S4/S4I/S4I.png]

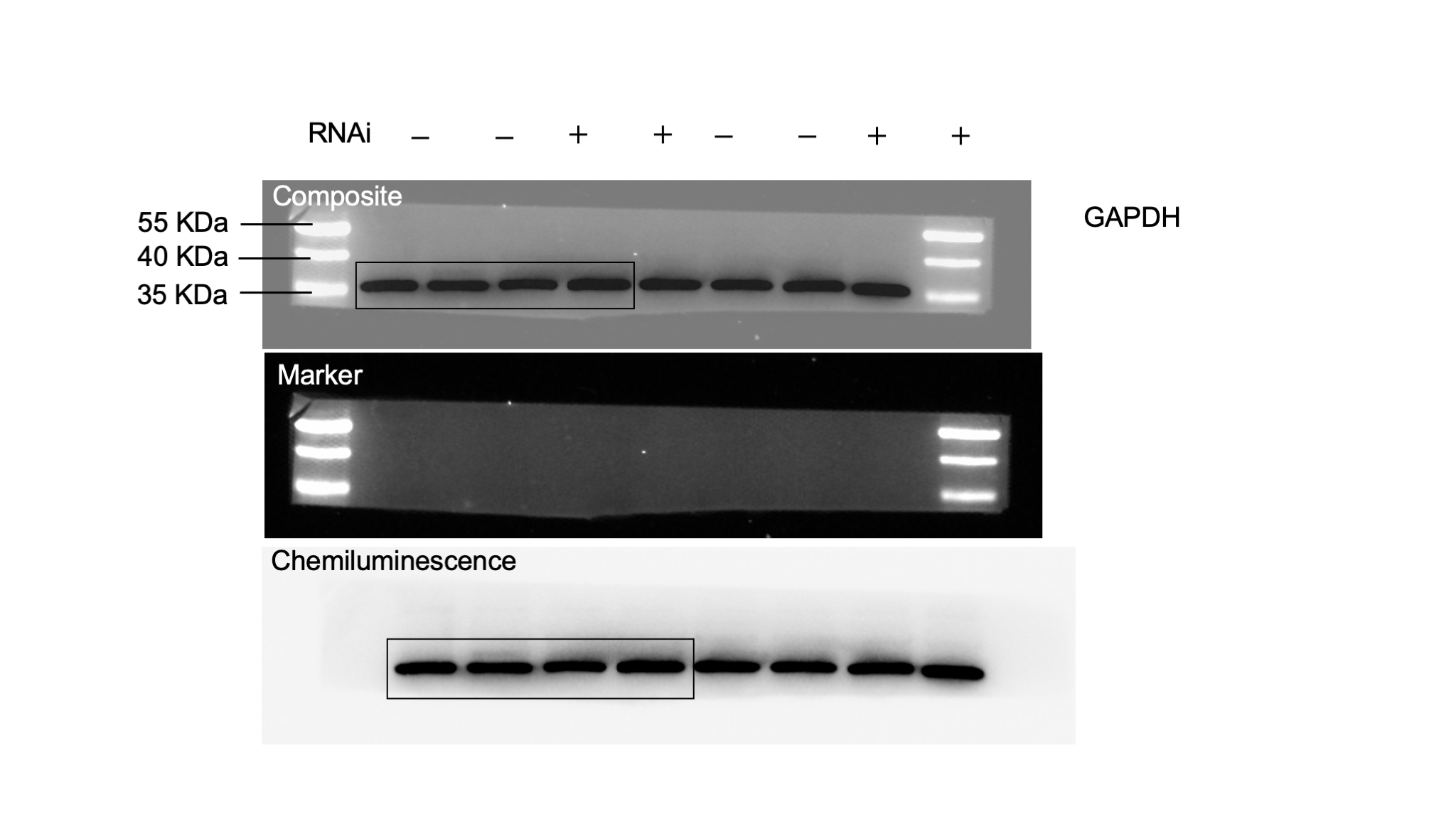

Supplement: Supplementary file 15 — Appendix Figure S5 Source Data [file 44319_2025_646_MOESM15_ESM.zip › Appendix Figure S5/S5D/S5D-GAPDH.tiff]

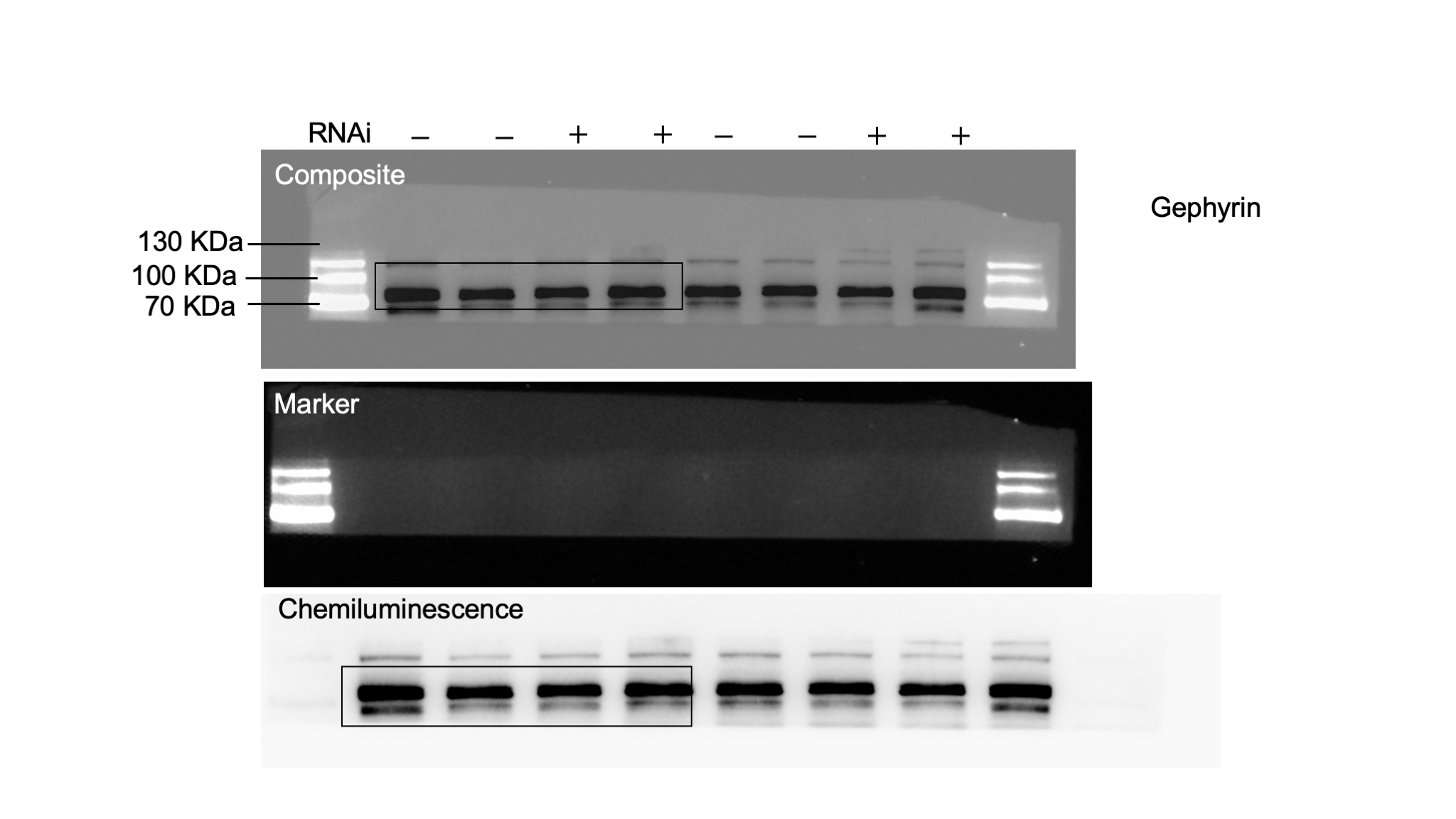

Supplement: Supplementary file 15 — Appendix Figure S5 Source Data [file 44319_2025_646_MOESM15_ESM.zip › Appendix Figure S5/S5D/S5D-Gephyrin.tiff]

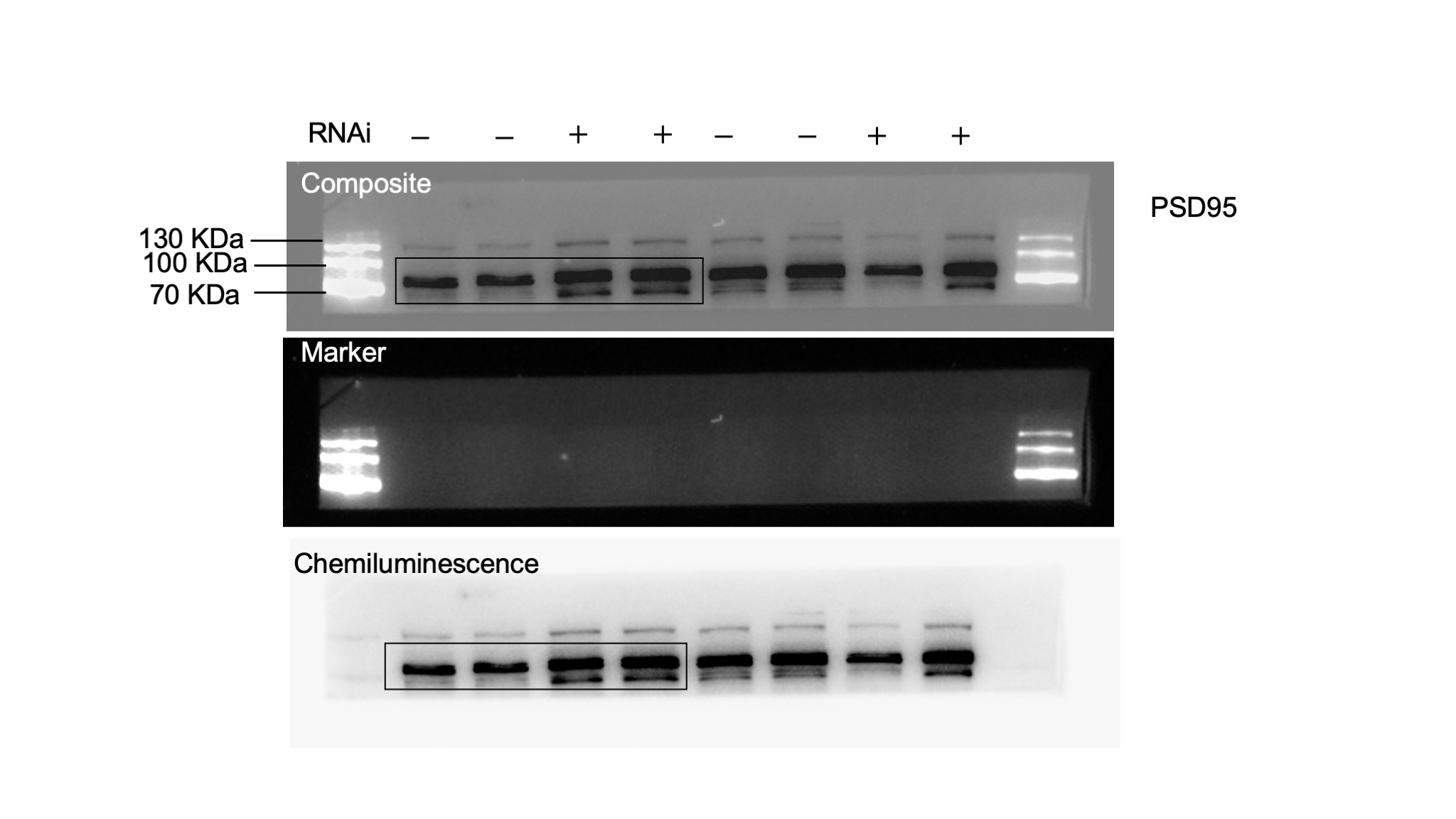

Supplement: Supplementary file 15 — Appendix Figure S5 Source Data [file 44319_2025_646_MOESM15_ESM.zip › Appendix Figure S5/S5D/S5D-PSD95.tiff]

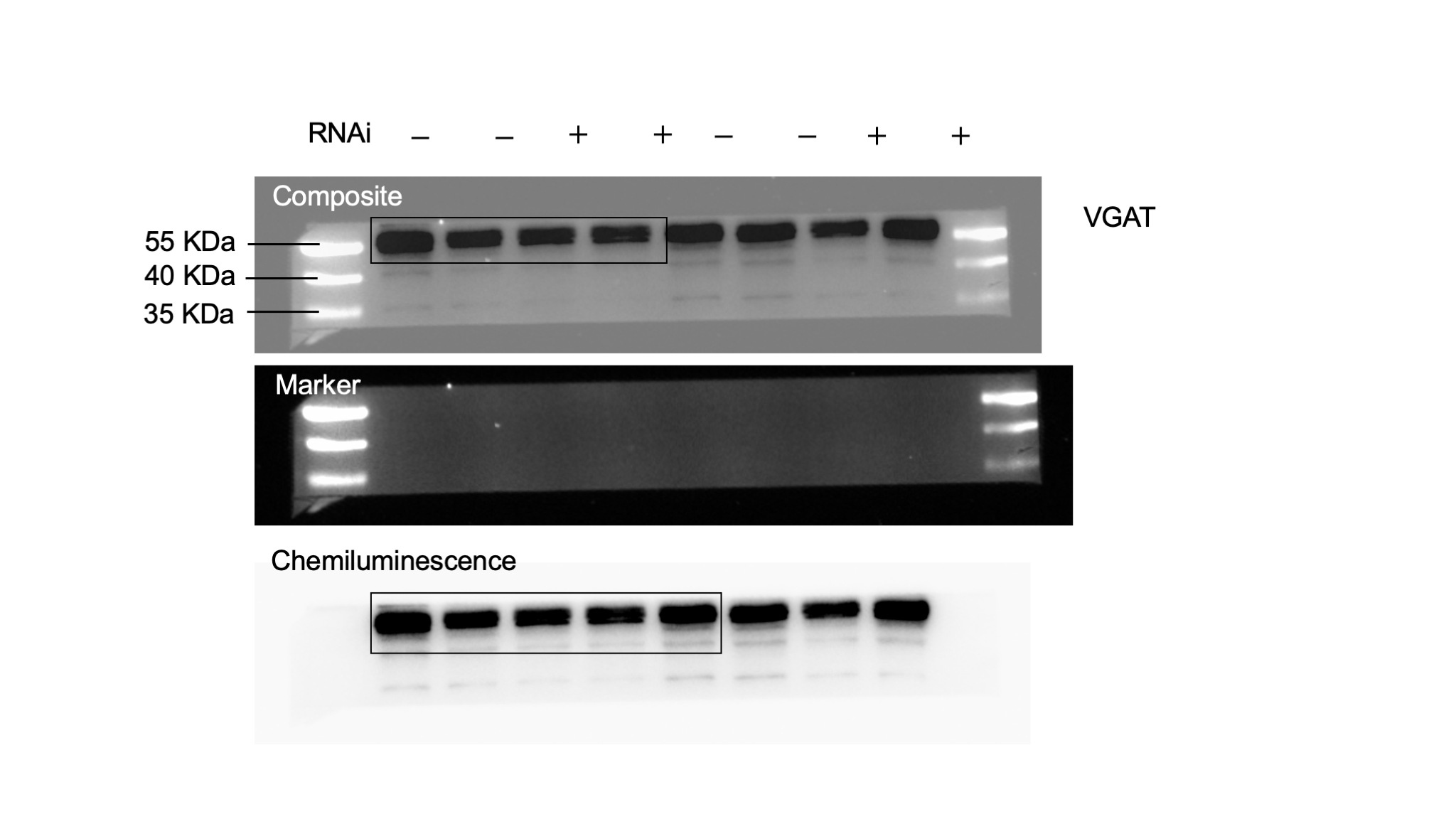

Supplement: Supplementary file 15 — Appendix Figure S5 Source Data [file 44319_2025_646_MOESM15_ESM.zip › Appendix Figure S5/S5D/S5D-VGAT.tiff]

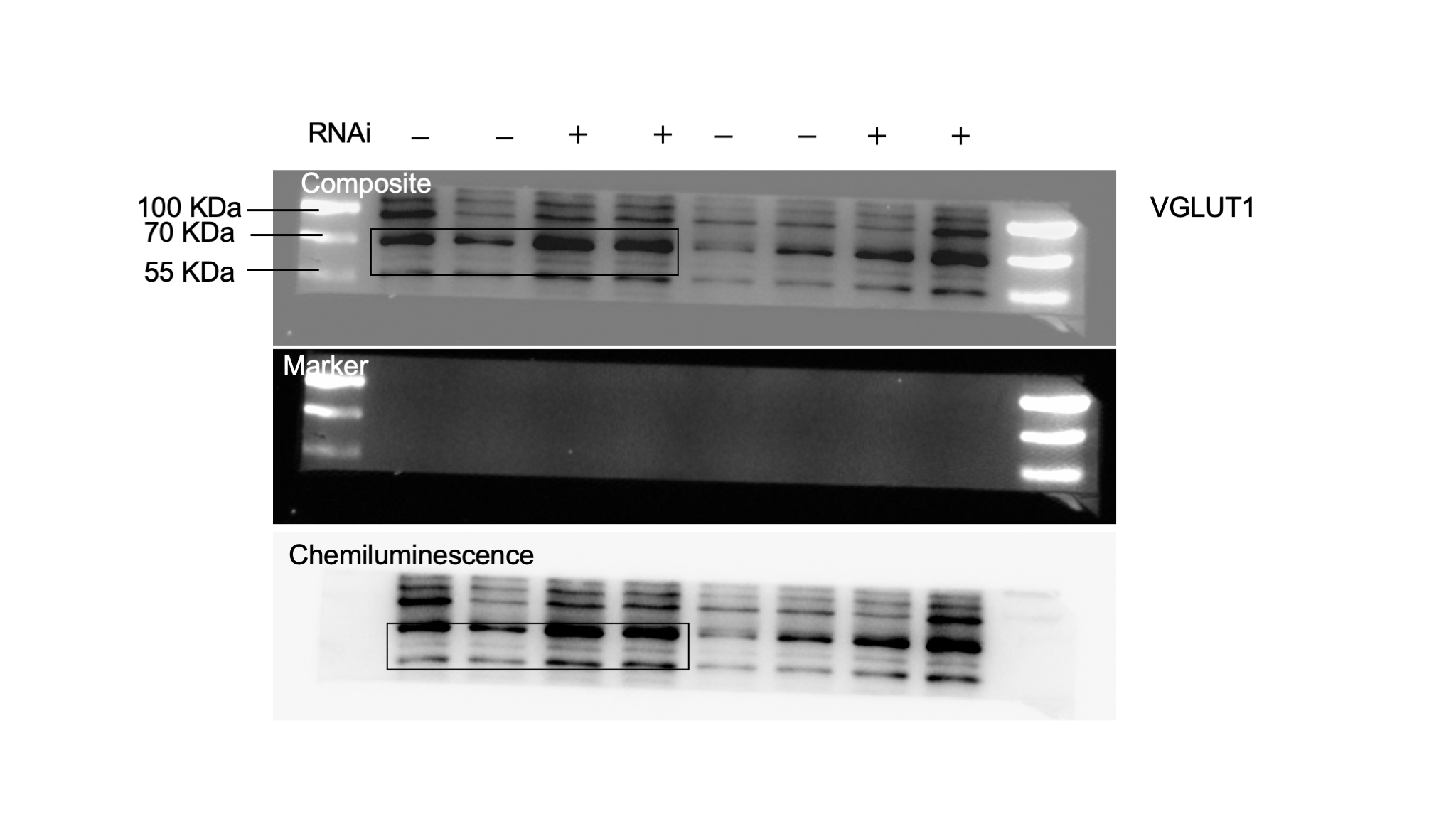

Supplement: Supplementary file 15 — Appendix Figure S5 Source Data [file 44319_2025_646_MOESM15_ESM.zip › Appendix Figure S5/S5D/S5D-VGLUT1.tiff]

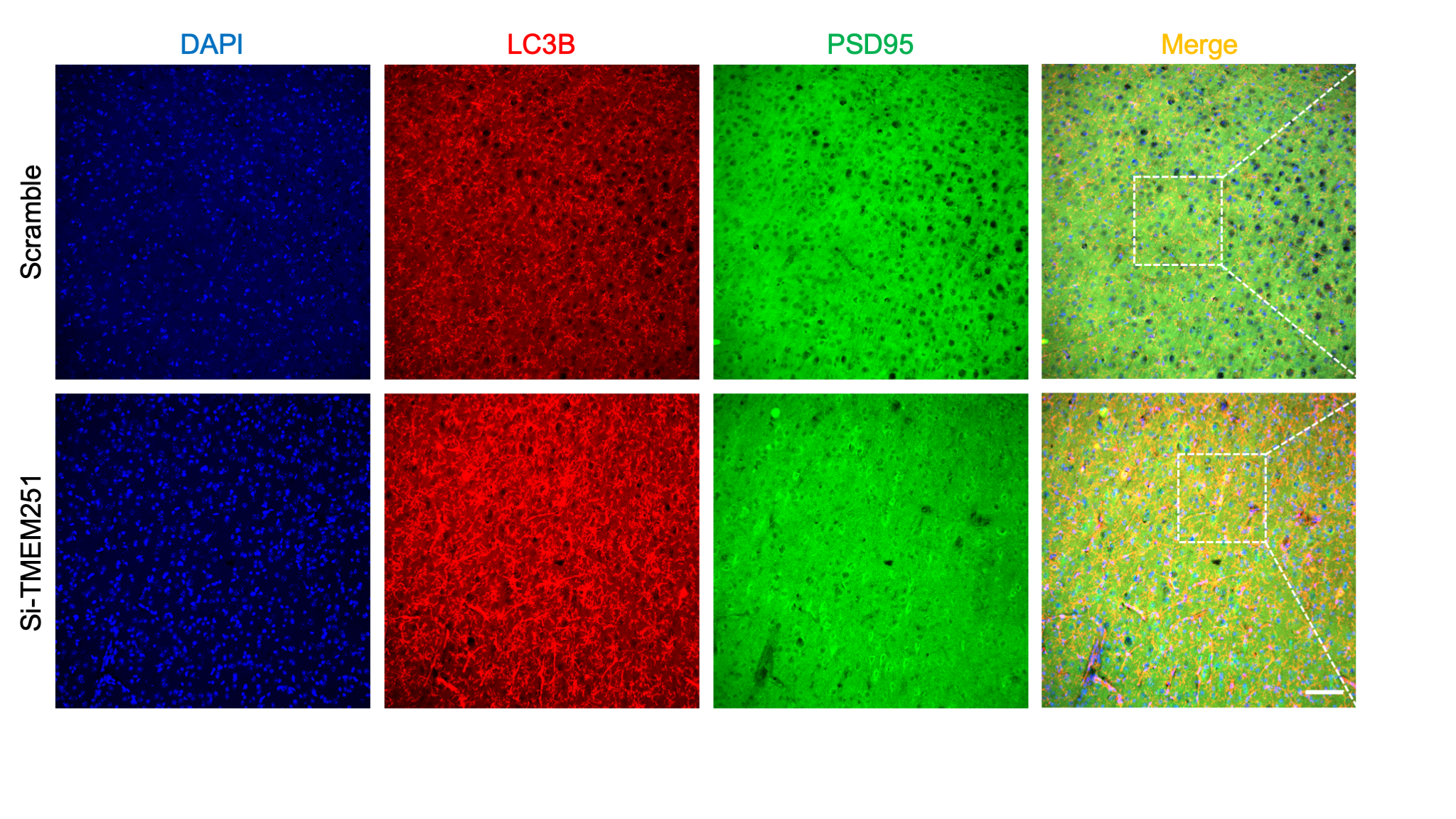

Supplement: Supplementary file 15 — Appendix Figure S5 Source Data [file 44319_2025_646_MOESM15_ESM.zip › Appendix Figure S5/S5E/S5E.tiff]

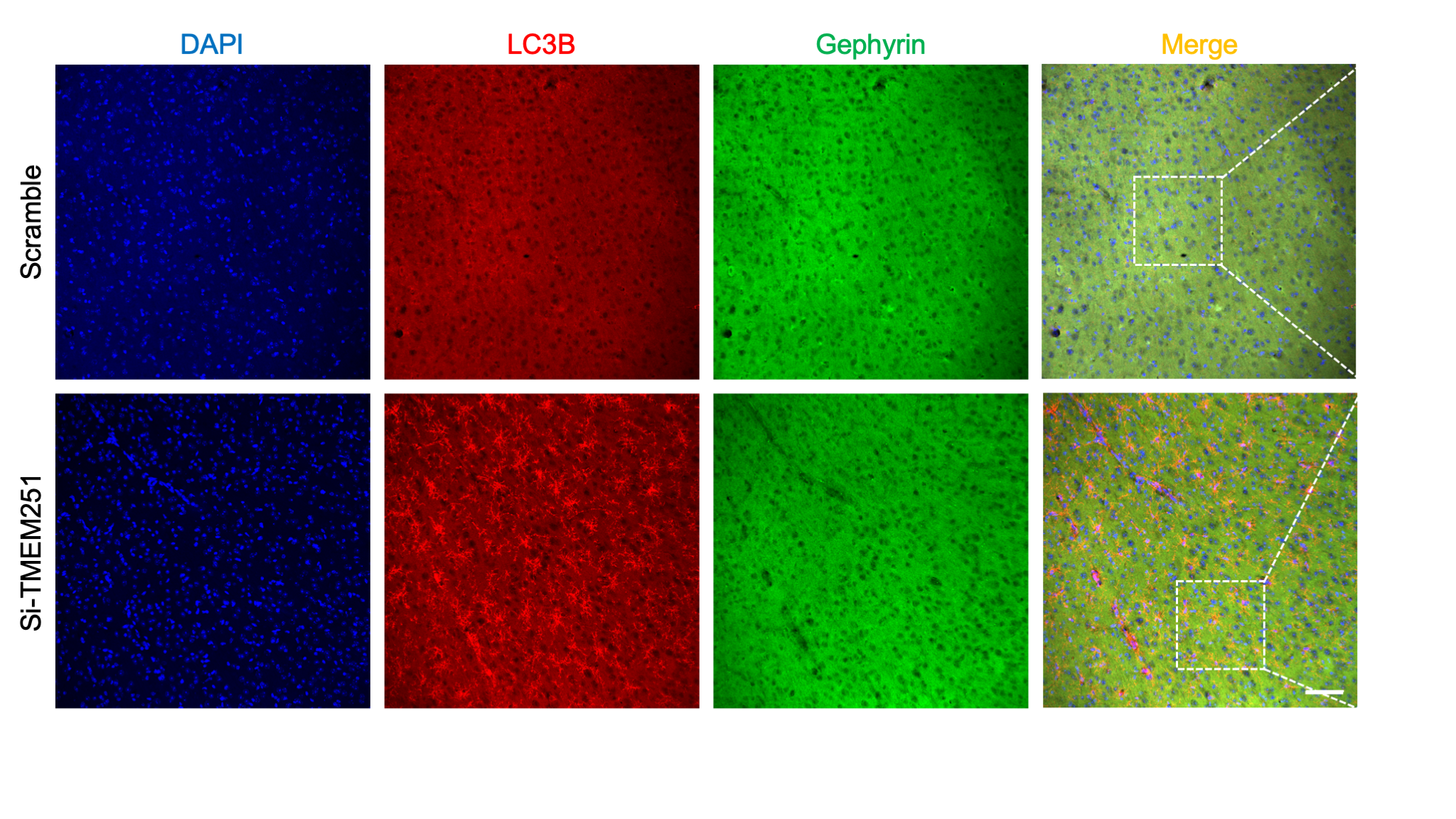

Supplement: Supplementary file 15 — Appendix Figure S5 Source Data [file 44319_2025_646_MOESM15_ESM.zip › Appendix Figure S5/S5F/S5F.tiff]

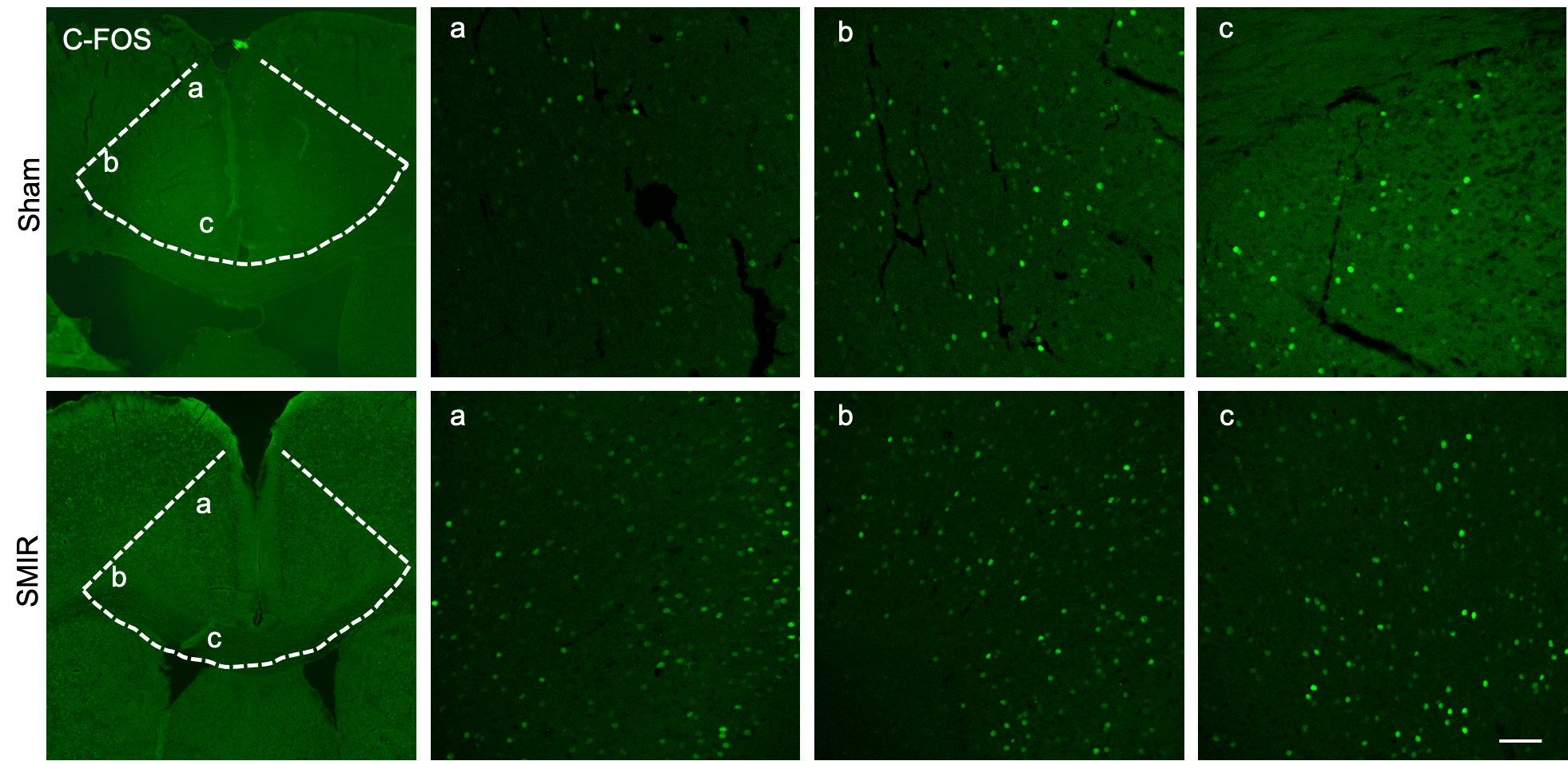

Supplement: Supplementary file 16 — Appendix Figure S6 Source Data [file 44319_2025_646_MOESM16_ESM.zip › Appendix Figure S6/S6A/S6A.png]

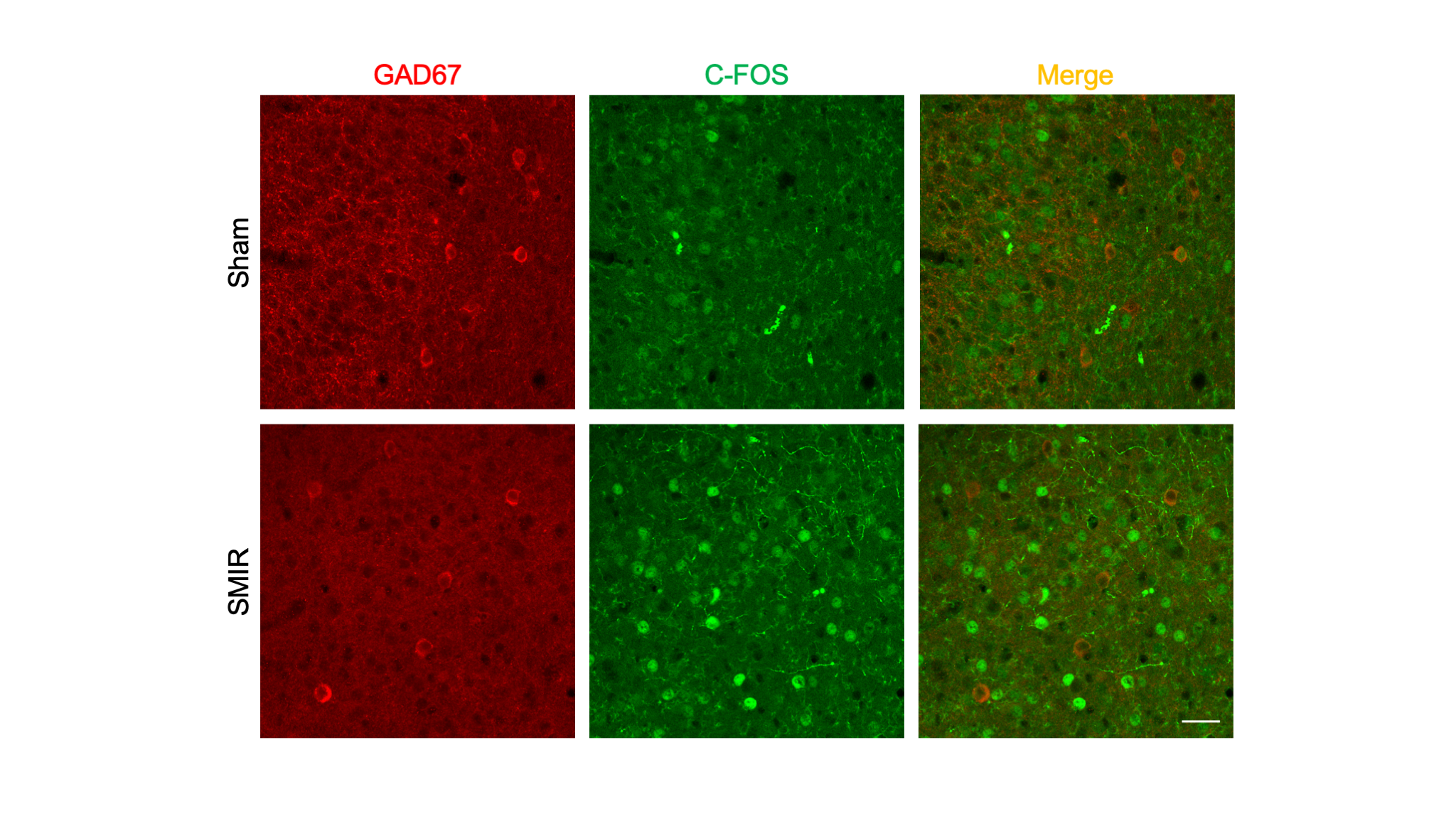

Supplement: Supplementary file 16 — Appendix Figure S6 Source Data [file 44319_2025_646_MOESM16_ESM.zip › Appendix Figure S6/S6B/S6B.tiff]

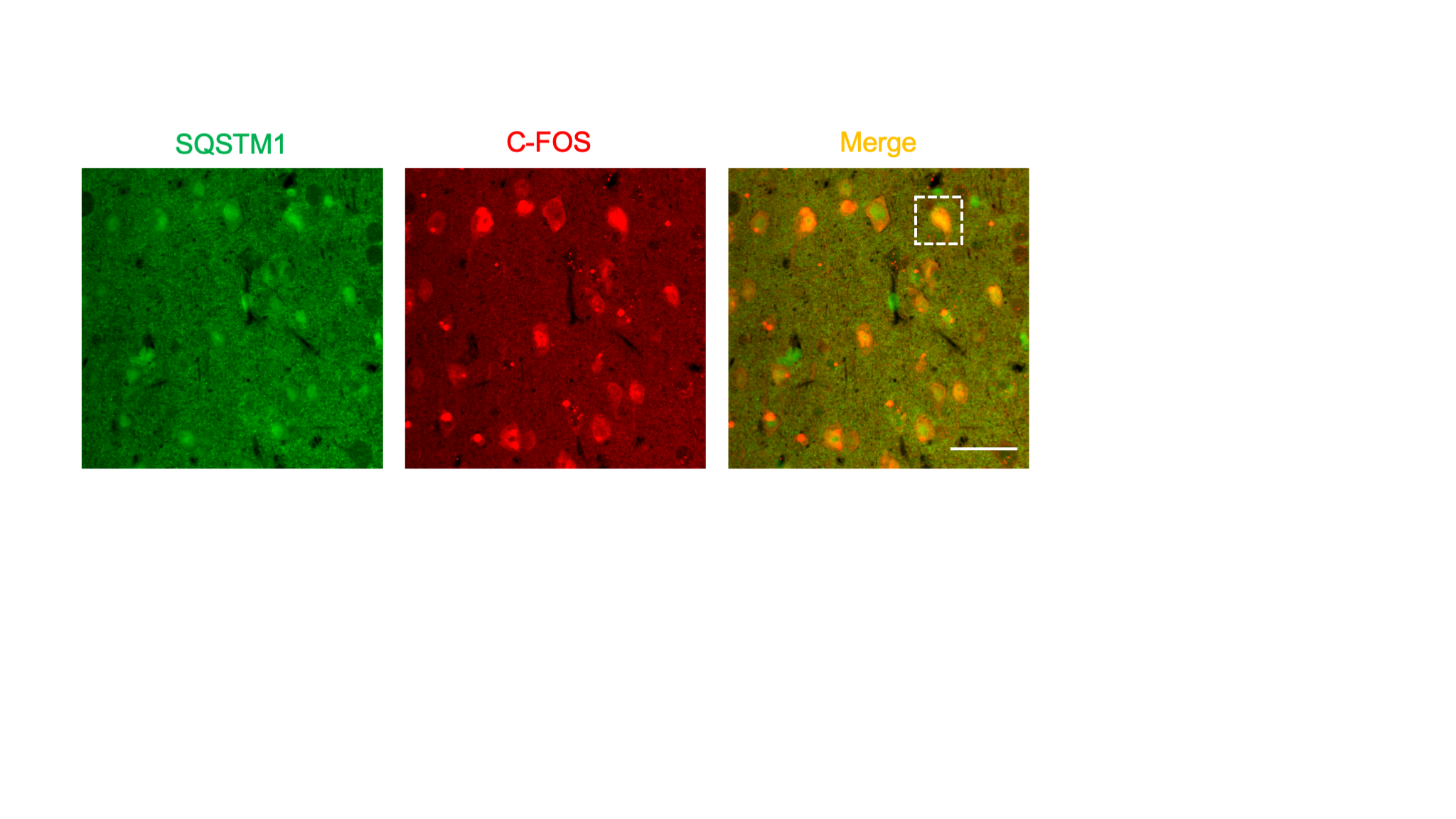

Supplement: Supplementary file 16 — Appendix Figure S6 Source Data [file 44319_2025_646_MOESM16_ESM.zip › Appendix Figure S6/S6C/S6C.tiff]

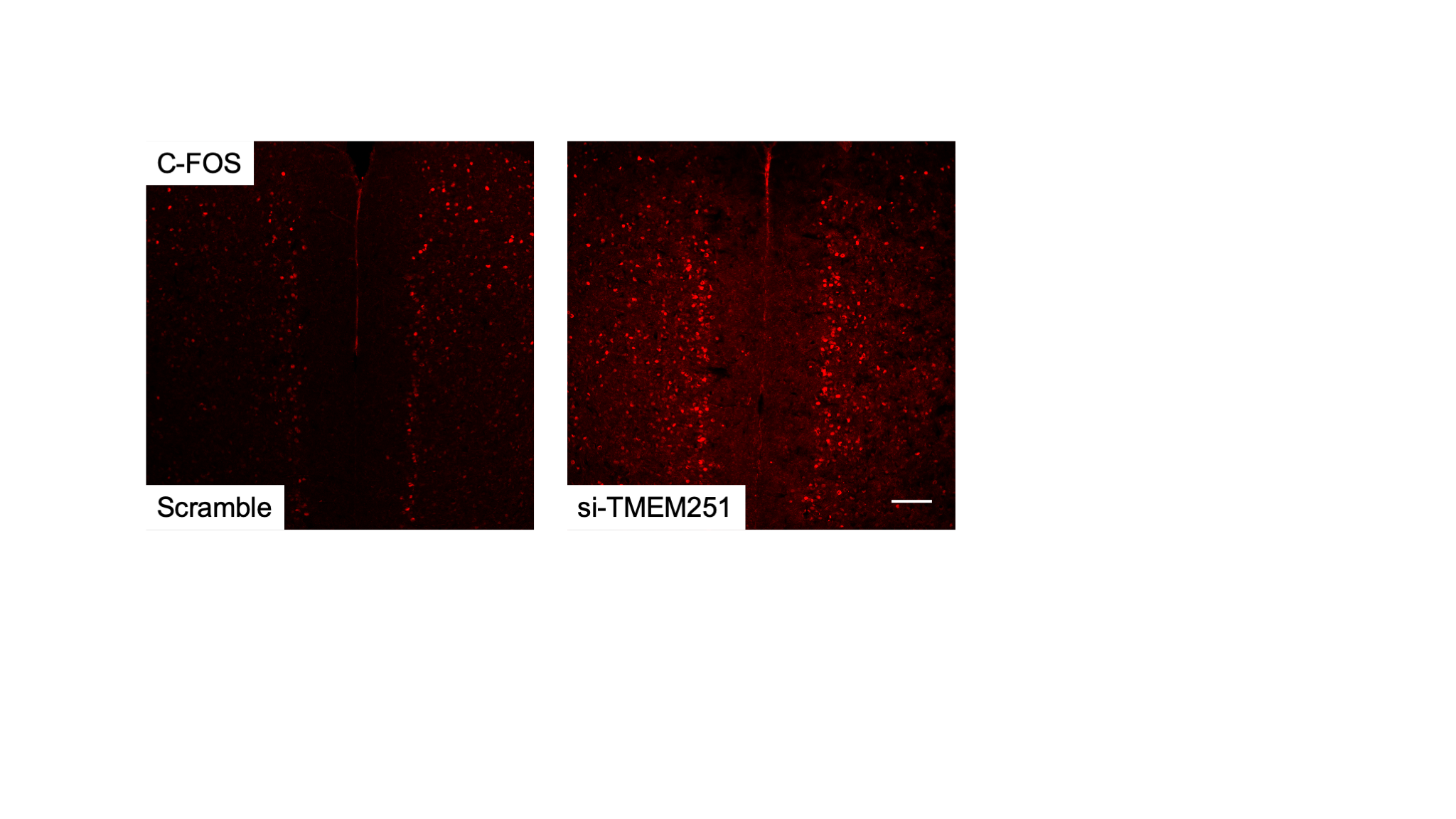

Supplement: Supplementary file 16 — Appendix Figure S6 Source Data [file 44319_2025_646_MOESM16_ESM.zip › Appendix Figure S6/S6D/S6D.tiff]

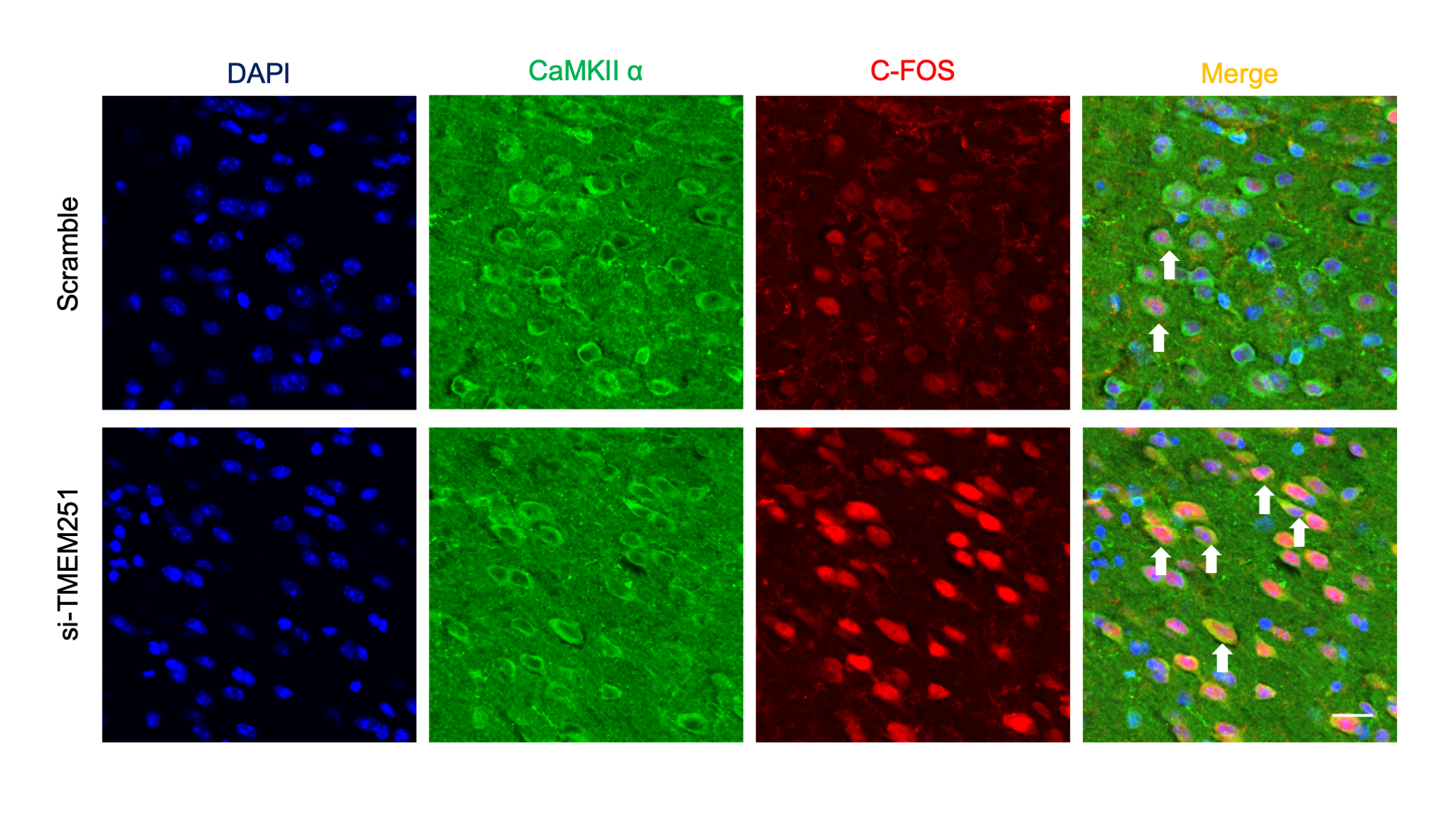

Supplement: Supplementary file 16 — Appendix Figure S6 Source Data [file 44319_2025_646_MOESM16_ESM.zip › Appendix Figure S6/S6E/S6E-CaMKII.tiff]

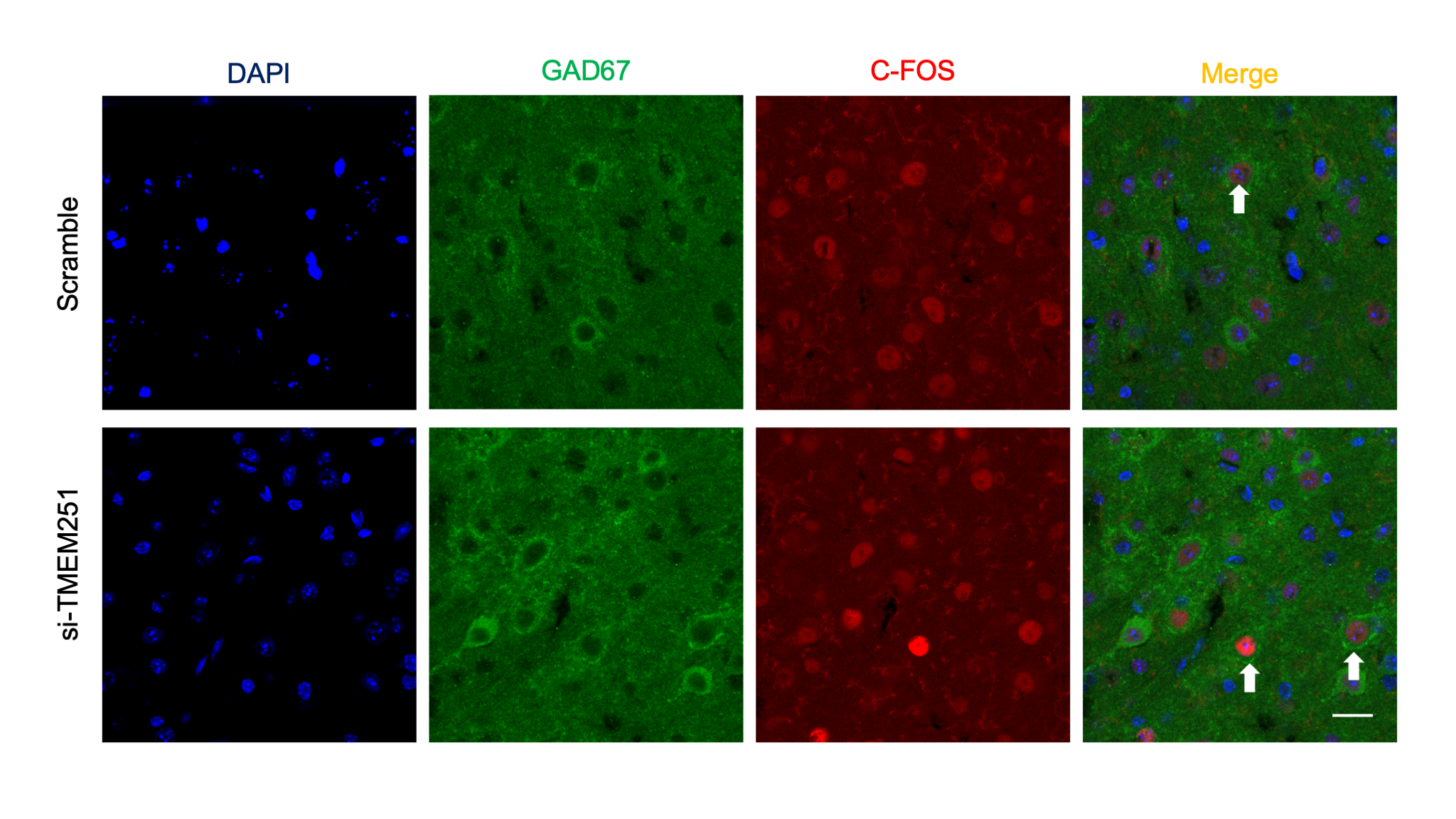

Supplement: Supplementary file 16 — Appendix Figure S6 Source Data [file 44319_2025_646_MOESM16_ESM.zip › Appendix Figure S6/S6E/S6E-GAD67.tiff]

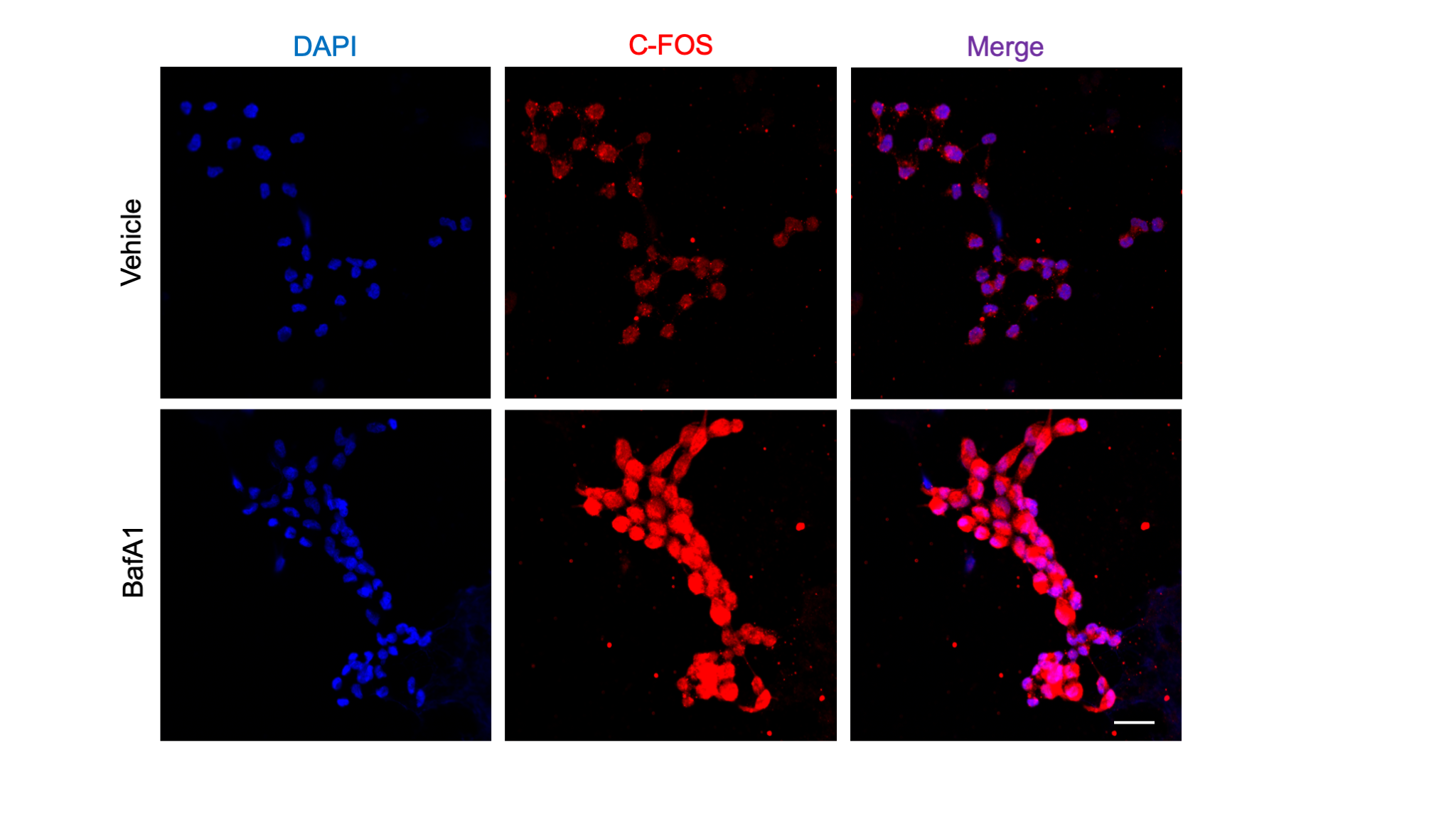

Supplement: Supplementary file 16 — Appendix Figure S6 Source Data [file 44319_2025_646_MOESM16_ESM.zip › Appendix Figure S6/S6F/S6F.tiff]

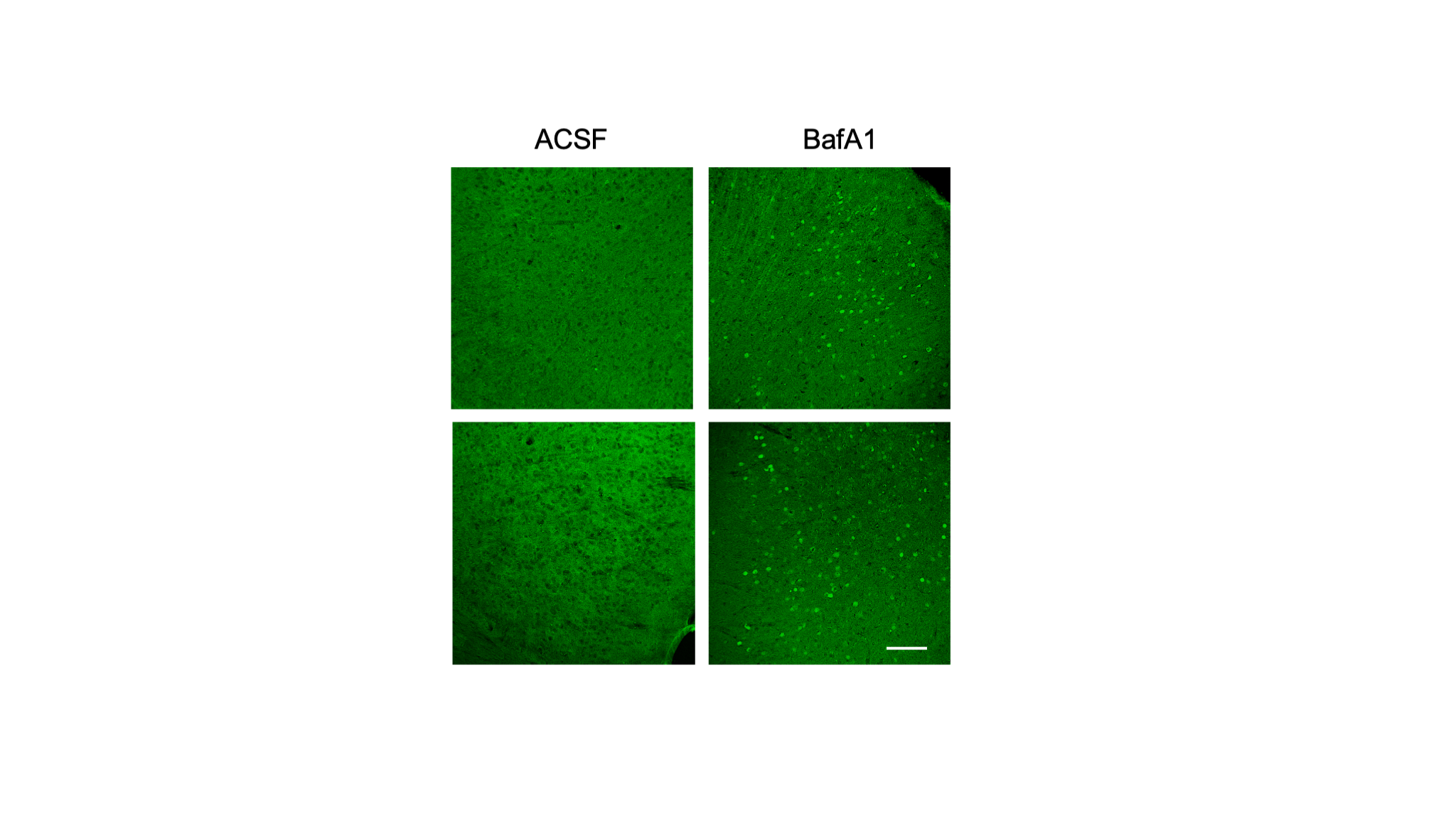

Supplement: Supplementary file 16 — Appendix Figure S6 Source Data [file 44319_2025_646_MOESM16_ESM.zip › Appendix Figure S6/S6G/S6G.tiff]

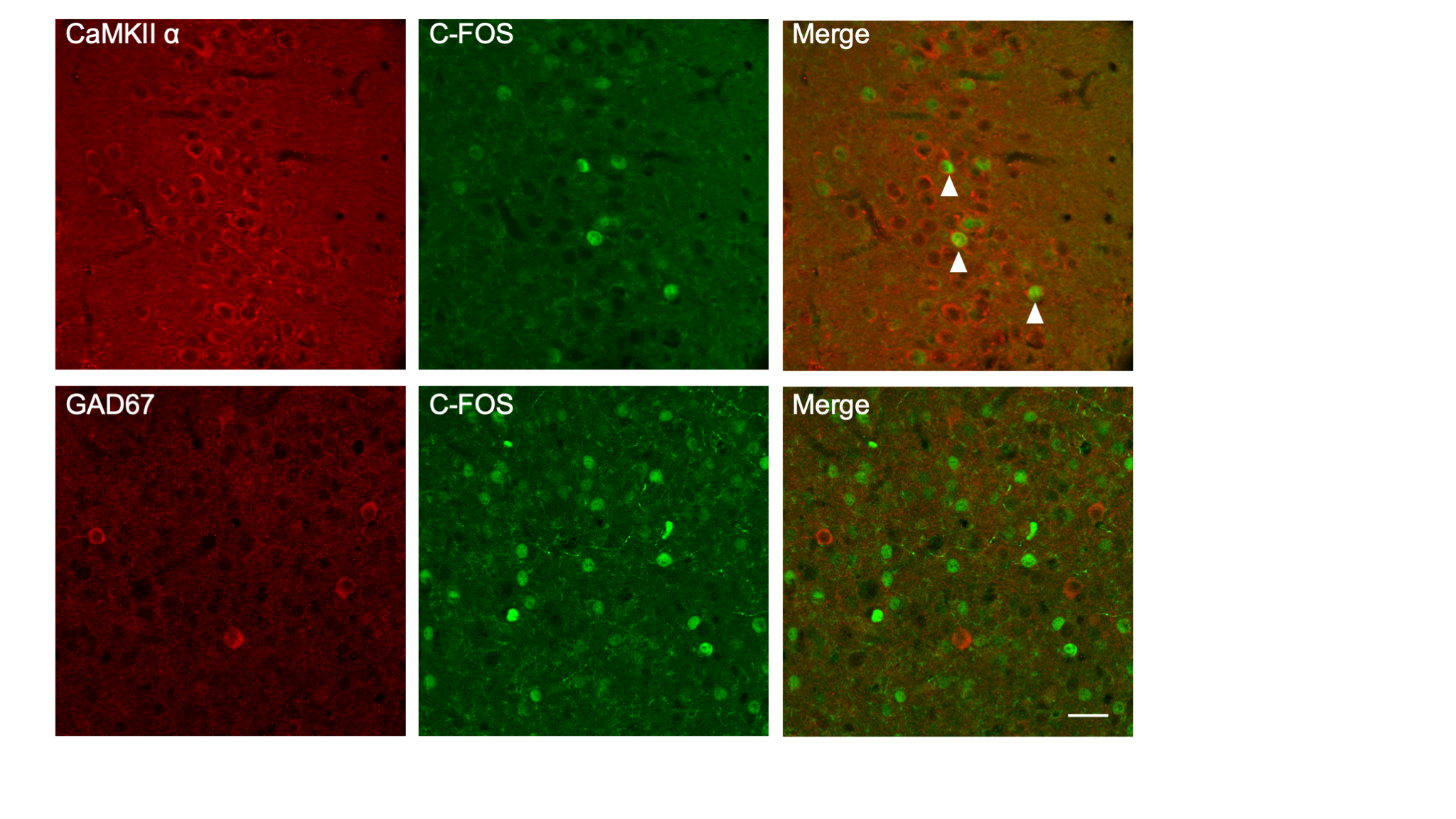

Supplement: Supplementary file 16 — Appendix Figure S6 Source Data [file 44319_2025_646_MOESM16_ESM.zip › Appendix Figure S6/S6H/S6H.tiff]

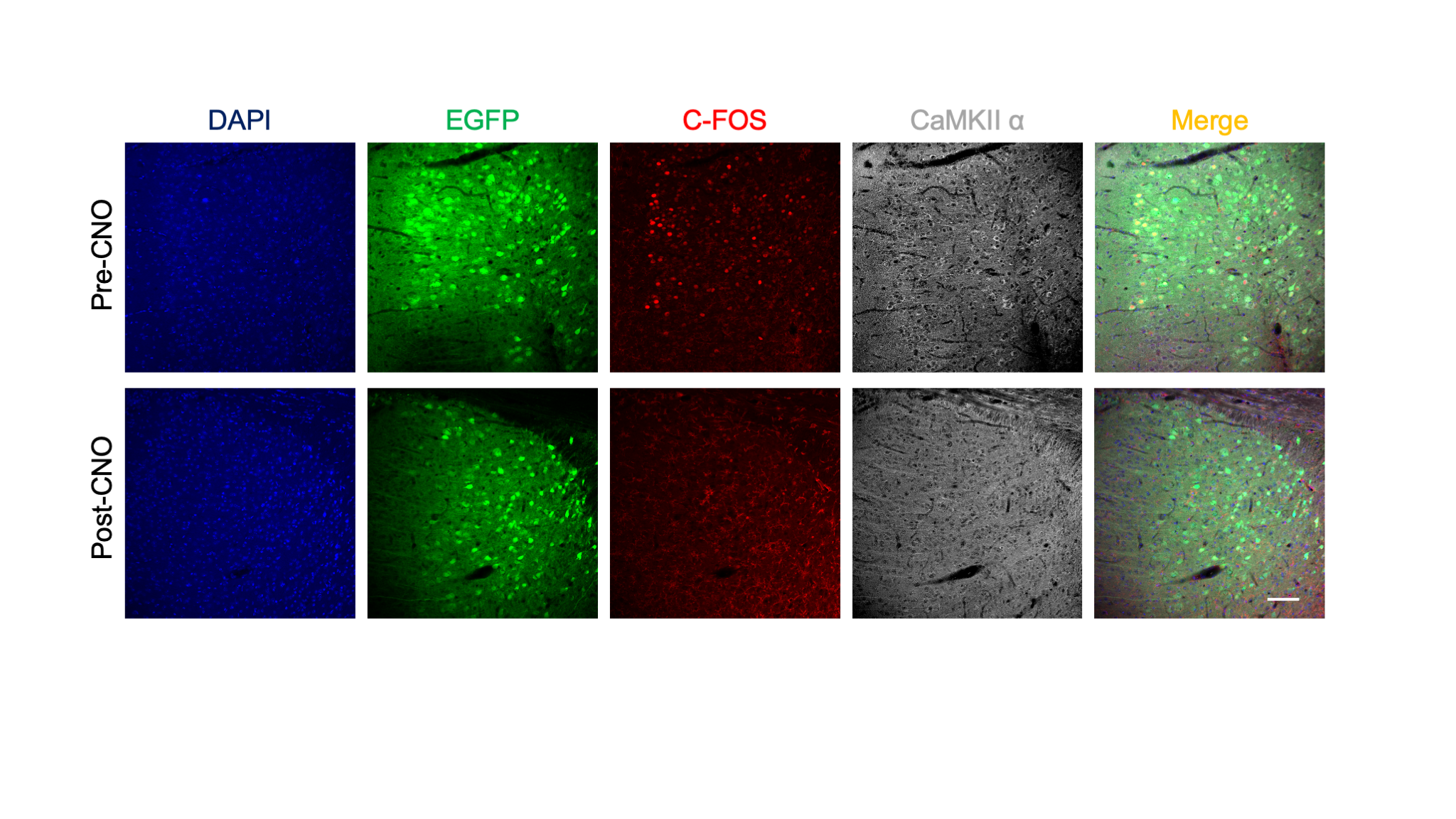

Supplement: Supplementary file 17 — Appendix Figure S7 Source Data [file 44319_2025_646_MOESM17_ESM.zip › Appendix Figure S7/S7E/S7E.tiff]

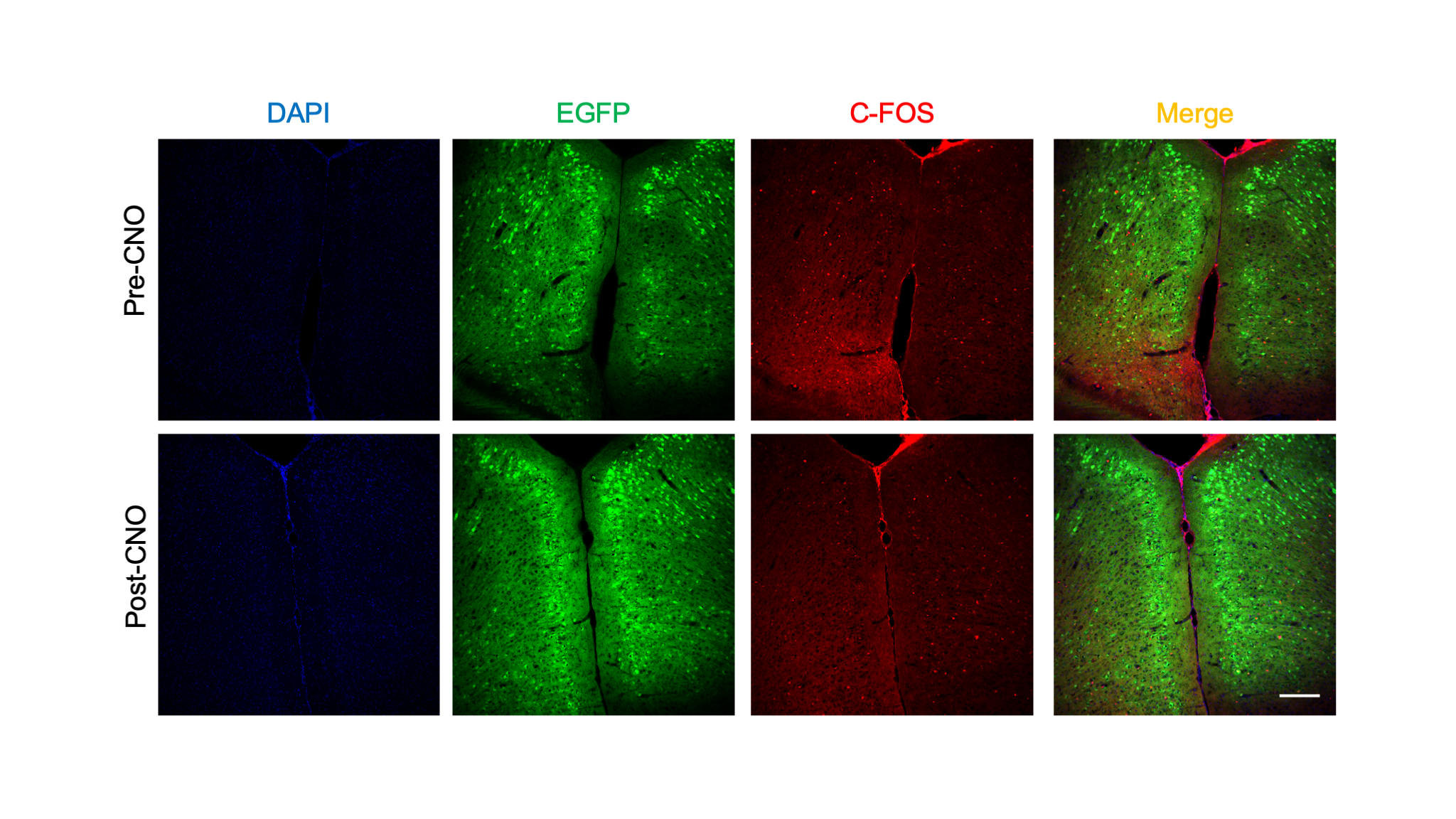

Supplement: Supplementary file 17 — Appendix Figure S7 Source Data [file 44319_2025_646_MOESM17_ESM.zip › Appendix Figure S7/S7J/S7J.tiff]

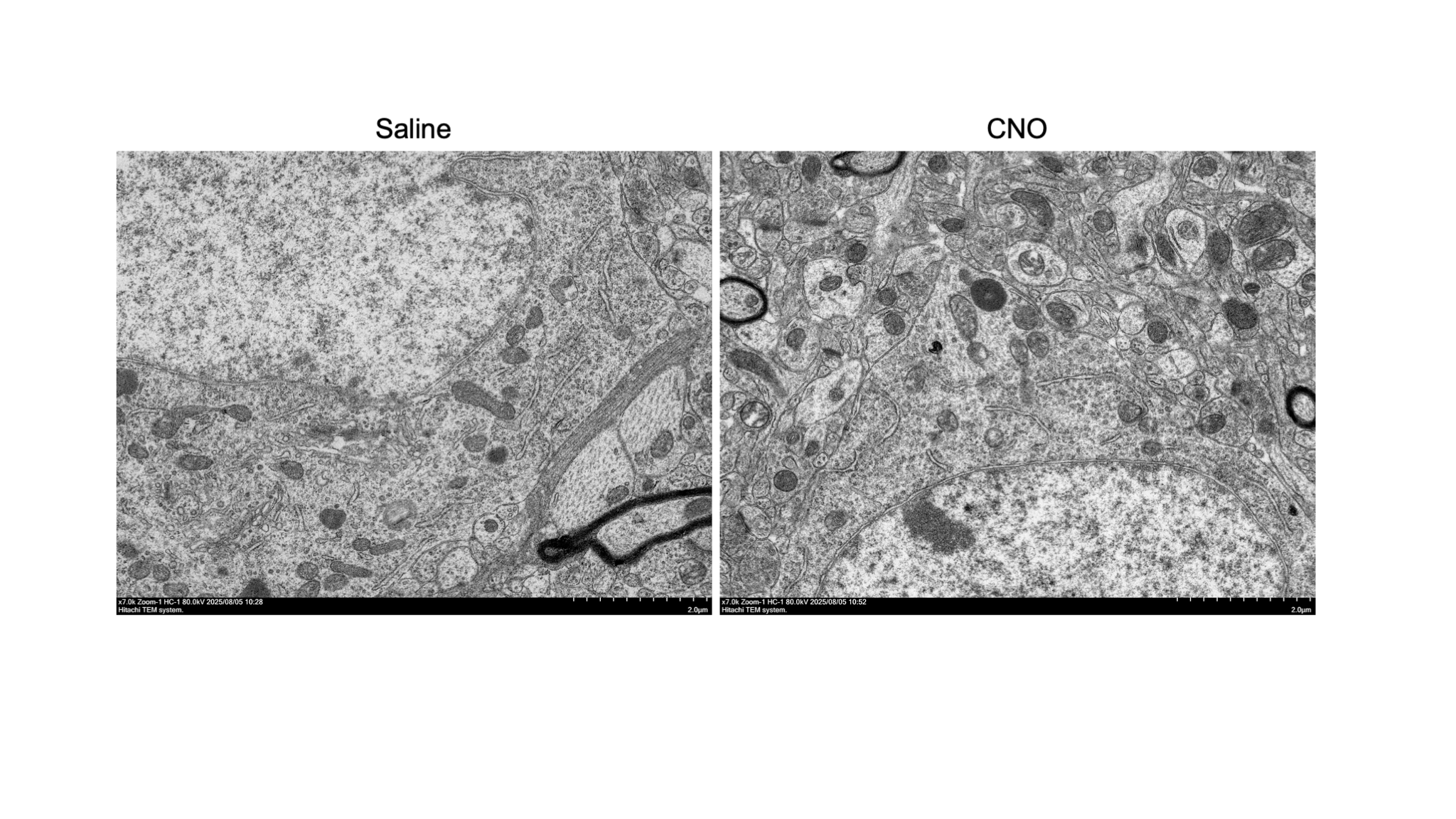

Supplement: Supplementary file 18 — Appendix Figure S8 Source Data [file 44319_2025_646_MOESM18_ESM.zip › Appendix Figure S8/S8A/S8A.tiff]

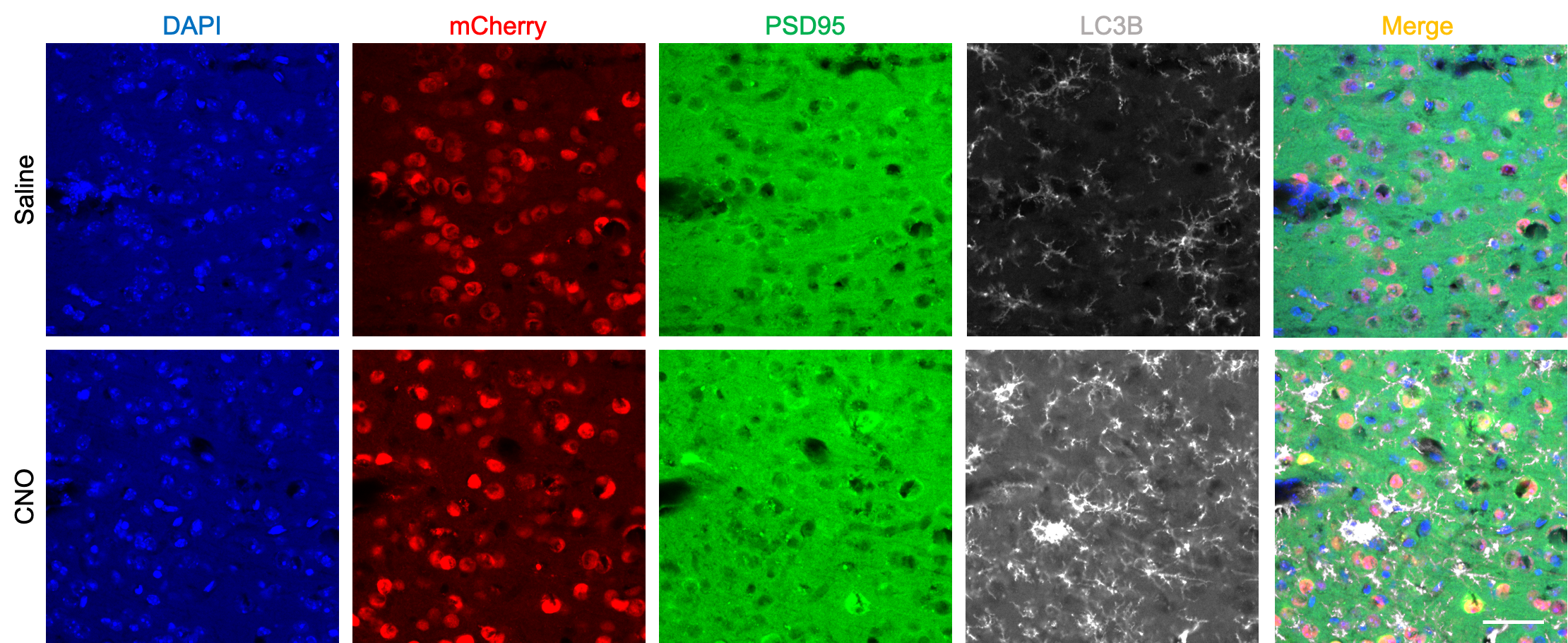

Supplement: Supplementary file 18 — Appendix Figure S8 Source Data [file 44319_2025_646_MOESM18_ESM.zip › Appendix Figure S8/S8B/S8B.png]

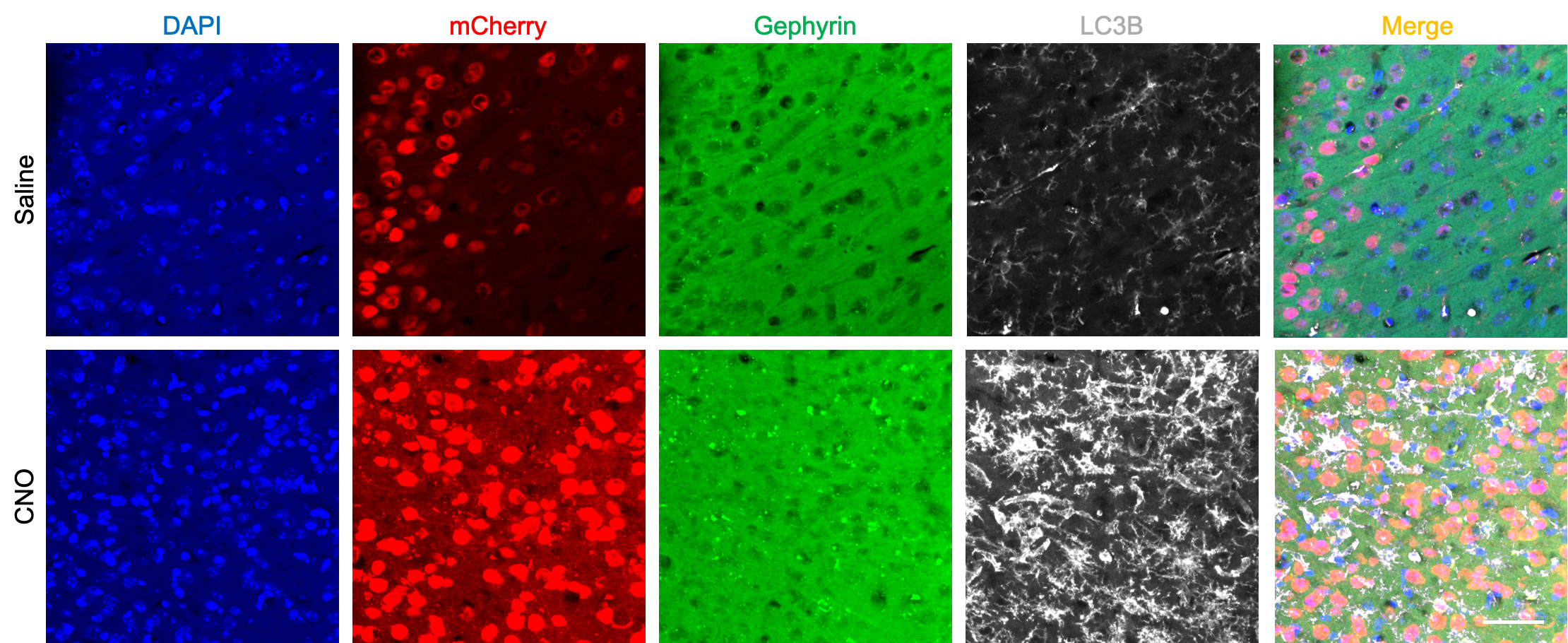

Supplement: Supplementary file 18 — Appendix Figure S8 Source Data [file 44319_2025_646_MOESM18_ESM.zip › Appendix Figure S8/S8C/S8C.png]
